# Causal Effects Between Retinal Characteristics and Cardiovascular Diseases: Insights from Genetic Correlation, Mendelian Randomization, and Cross-Sectional Study

**DOI:** 10.5334/gh.1493

**Published:** 2025-11-21

**Authors:** Xuehao Cui, Chao Sun, Dejia Wen, Jishan Xiao, Xiaorong Li

**Affiliations:** 1 John Van Geest Centre for Brain Repair and MRC Mitochondrial Biology Unit, Department of Clinical Neuroscience, University of Cambridge, Cambridge, CB2 0PY, UK; 2 Cambridge Eye Unit, Cambridge University Hospitals, Cambridge, UK; 3 Department of Ophthalmology, Zibo Central Hospital, Shandong, CN; 4 Eye Institute and School of Optometry, Tianjin Medical University Eye Hospital, Tianjin, CN; 5 Tianjin Key Laboratory of Retinal Functions and Diseases, Tianjin, CN; 6 Tianjin Branch of National Clinical Research Center for Ocular Disease, Tianjin, CN; 7 Institute of Ophthalmology, University College London, London, UK

**Keywords:** cardiovascular diseases, retina, optical coherence tomography, genetic correlation, Mendelian randomization, biomarkers, risk prediction model

## Abstract

**Background:**

Cardiovascular diseases (CVDs) are the leading global cause of mortality and disability, with prevalence increasing due to aging and risk factors like obesity and hypertension. The retina, rich in microvasculature, provides a unique opportunity to investigate microvascular dysfunction linked to CVDs and other systemic vascular diseases.

**Method:**

This study used a multifaceted approach to assess the genetic correlation and causal relationship between retinal characteristics and CVDs. Linkage disequilibrium score regression (LDSC) and Mendelian randomization (MR) analyses were conducted using genome-wide association study (GWAS) data from the UK Biobank and FinnGen datasets. A cross-sectional study was also conducted to validate the findings, collecting optical coherence tomography (OCT) images from 124 eyes (89 with CVDs and 35 healthy controls). A prediction model is based on least absolute shrinkage and selection operator (LASSO) regression to assess the risk of CVD.

**Result:**

Using LDSC and two-sample MR, we found genetic evidence consistent with a causal effect whereby genetically proxied thinner retinal nerve fiber layer (RNFL) was associated with higher risks of hypertension and myocardial infarction (MI), while genetically proxied thicker photoreceptor inner segment/outer segment (PR-IS/OS) was associated with coronary heart disease and MI (false discovery rate [FDR] thresholds as reported). Genetically proxied thinner retinal pigment epithelium (RPE) showed an inverse association with stroke risk. Several circulating biomarkers—including lipoprotein(a) [Lp(a)], low-density lipoprotein cholesterol (LDL-C), and ApoB—exhibited MR evidence of association with multiple CVDs. In a cross-sectional cohort, retinal layer differences and their relationships with lipids were directionally consistent with the genetic findings.

**Conclusion:**

Retinal structural traits measured by OCT—particularly RNFL, PR-IS/OS, and RPE thickness—are best interpreted as non-invasive markers that reflect systemic vascular biology. Our MR analyses support shared etiologic pathways between retinal microstructure and CVDs rather than implying that retinal damage clinically causes cardiovascular events. Findings warrant validation in larger and more diverse populations and should not be considered definitive proof of causality.

## Lay Summary

### Overview

This study explores how structural changes in the retina, identified through advanced imaging techniques, can serve as early indicators of cardiovascular diseases (CVDs). The findings highlight the retina’s potential as a window into cardiovascular health and disease progression by linking retinal characteristics with systemic vascular health.

### Key Findings

Key retinal layers, including the retinal nerve fiber layer (RNFL) and PR-IS/OS, exhibit significant causal relationships with primary cardiovascular conditions such as hypertension, myocardial infarction, and coronary heart disease. These findings suggest that retinal imaging could be used to monitor vascular health and identify individuals at high risk for CVDs.

Integrating retinal measurements with systemic biomarkers, such as cholesterol (CHO), triglycerides, and inflammatory factors, improves the predictive accuracy for cardiovascular risk. This study demonstrates how non-invasive retinal imaging could complement traditional diagnostic tools in assessing and managing cardiovascular health.

The research underscores the importance of further investigation into the use of retinal biomarkers for early detection and personalized treatment strategies for cardiovascular diseases. These findings offer significant implications for public health, emphasizing the potential for retina-based diagnostics to reduce the global burden of CVDs. This study suggests that retinal imaging may serve as a non-invasive biomarker for cardiovascular risk assessment. With accessible and cost-effective retinal imaging technologies, particularly in low- and middle-income countries, this approach could play a transformative role in preventive cardiovascular care.

## Introduction

Cardiovascular diseases (CVDs) constitute the primary cause of mortality and a significant source of disability globally, imposing a substantial burden on public health (1). With an estimated 523 million people affected and causing over 18.6 million deaths annually, the prevalence of CVDs is exacerbated by the aging population, highlighting the urgent need for effective prevention strategies (2). Recognized risk factors, including obesity, smoking, hyperlipidemia, hypertension, and diabetes, further underscore the complexity and multifaceted nature of this health challenge (3). CVDs encompass conditions that impair the structure and function of the heart, including high blood pressure (HBP), myocardial infarction (MI), heart failure (HF), coronary heart disease (CHD), atrial fibrillation (AF), cardiac arrhythmias (CA), and stroke. HBP is a multifaceted public health issue affecting millions globally, has the most substantial causal evidence among the risk factors for CVD, and is highly prevalent (4). HF is defined as the inability of the heart muscle to adequately supply the necessary amounts of oxygen and blood to meet the metabolic needs of the peripheral tissues (5). MI is a leading cause of global mortality and triggers inflammatory and immune responses that result in myocardial damage, with early reperfusion being key and biomedical materials offering new hope for enhancing its diagnosis and treatment. CA is an abnormal heart rhythm due to disrupted electrical activity (6). It annually claims millions of lives despite current treatments, including drug therapy, radiofrequency ablation, and cardiovascular implantable electronic devices, which have limitations like medication side effects, implantation risks, and surgical complications (7). AF is the most prevalent CA and poses significant challenges due to its complex pathophysiology and suboptimal efficacy of current treatments, with a rising incidence and research identifying defects in specific molecular pathways as fundamental to its pathogenesis (8). CHD is a clinical syndrome characterized by plaque formation in the arterial intima, leading to vessel narrowing and occlusion, and it accounts for roughly 9.1 million deaths globally each year, with a disproportionate impact on low- to middle-income regions (9). Stroke is the second leading cause of death globally, affecting up to one in five people in high-income countries and almost one in two in low-income countries, characterized by sudden neurological deficits often due to vascular causes such as extensive artery disease, cardiometabolic, and small vessel disease (10).

Recently, researchers have recognized the significant role of microvascular dysfunction in CVDs; however, there are difficulties in directly studying the microcirculation of the heart and brain (11). The retina, as the only neural tissue in the body that can be directly imaged and is rich in a microcirculatory system composed of numerous microvessels, offers an opportunity to investigate the structure and pathology of the human circulatory system (12). Retinal microvascular anomalies have been associated with systemic vascular diseases, including arterial disease, chronic renal disease, and hypertension (13). A recent study has shown that the thickness of the retinal ganglion inner plexiform layer (GCIPL) is associated with MI, HF, stroke, and CVD mortality (14). A previous study has found that macular retinal thickness is significantly reduced in patients with systemic hypertension compared to the control group (15). Another research showed that the thickness of the retinal nerve fiber layer (RNFL), ganglion cell layer(GCL), and photoreceptor outer segments (PR/OS) was inverse, and inner nuclear layer (INL) thickness was positively associated with HBP, while the thickness of the other retinal layers was not significantly correlated with blood pressure (16). Optical coherence tomography (OCT) and optical coherence tomography angiography (OCTA) are novel non-invasive methods capable of precise visualization and quantification of retinal and choroidal vascular structures and have been applied in assessing patients with CVDs (17).

Linkage disequilibrium score regression (LDSC) is a statistical method used to estimate complex traits’ heritability and distinguish between confounding and causal genetic associations (18). In addition, LDSC enables the evaluation of genetic correlations between characteristics based on summary statistics derived from GWAS data. Mendelian randomization (MR) applies instrumental variable (IV) analysis to test causal hypotheses in non-experimental data. In MR analysis, genetic variations, typically single-nucleotide polymorphisms (SNPs), are used as IVs for putative risk factors (19). The principle of MR refers to Mendel’s second law during DNA transmission, similar to random treatment assignment in a randomized controlled trial (RCT), aiming to produce comparable groups and reduce confounding risk. Well-powered genome-wide association studies (GWAS) of retinal characteristics measured by OCT from the UK Biobank dataset have identified hundreds of SNPs, which created an opportunity to test potential causal relationships between retinal characteristics and CVDs.

This study used LDSC and two-sample bidirectional MR to assess the genetic correlation and the causal relationship between retinal characteristics and CVDs. Subsequently, we collected OCT images from 124 eyes and extracted data on the thickness of various retinal layers and biomarkers, including 89 subjects with CVDs and 35 healthy controls (HC). We validated the results from LDSC and MR using a cross-sectional study and further explored the relationship between retinal characteristics and clinical biomarkers. We established a risk prediction model for CVDs using retinal characteristics and biomarkers based on LASSO regression.

## Materials and Methods

### Study design

**Figure 1:**
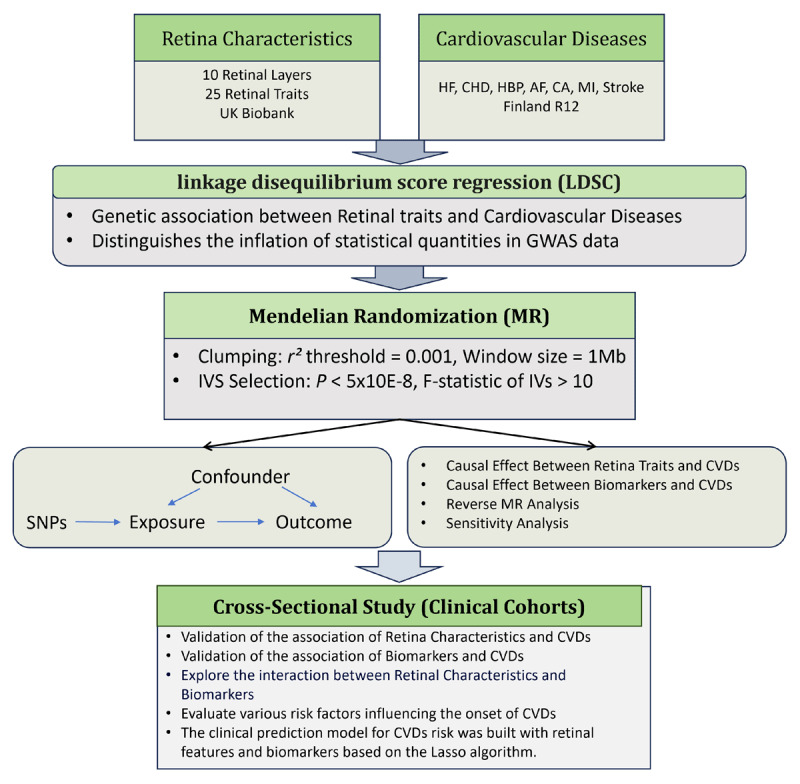
Study design and workflow. Schematic overview of the study design. Retinal traits (10 retinal layers and 25 retinal phenotypes from UK Biobank) and cardiovascular diseases (CVDs, including HF, CHD, HBP, AF, CA, MI, and stroke from the Finland R12 dataset) were analyzed. Linkage disequilibrium score regression (LDSC) was used to evaluate genetic correlations, followed by Mendelian randomization (MR) to infer causal effects, with sensitivity analyses included. Cross-sectional validation was performed in clinical cohorts to test associations between retinal features, circulating biomarkers, and CVDs.

The Institutional Review Board (IRB) approved the study and adhered to the Declaration of Helsinki. The study design is illustrated in Figure 1. We utilized LDSC and MR to investigate the genetic correlations and causal relationships between retinal traits, clinical biomarkers, and CVDs. To ensure the robustness of the MR analysis, three key criteria must be satisfied: (1) The genetic variants used must exhibit a statistically significant correlation with the exposure; (2) the chosen genetic variants acting as IVs must be unrelated to any confounding factors that concurrently affect both the exposure and the outcome; and (3) horizontal pleiotropy must be absent, implying that the IVs should exclusively exert their influence on CVDs via the exposure pathway. Subsequently, a cross-sectional study was conducted to validate the earlier causal relationships and further explore the associations between retinal traits, biomarkers, and CVDs. Finally, a risk prediction model of retinal characteristics and biomarkers for CVDs was developed based on LASSO regression.

### Data source and study population in LDSC and MR

The UK Biobank is a large-scale prospective cohort study that recruited more than half a million individuals, aged 40 to 69, from 22 different assessment centers across the United Kingdom from 2006 to 2010 (20). The GWAS of retinal characteristics is based on OCT examination results from the UK Biobank, from which we have identified 46 traits (Table S5), including 21 thickness characteristics of different retinal layers and regions (for both eyes) and four structural factors related to the optic nerve (for one eye). Our study involved a thorough examination and detailed fine mapping of loci linked to 35 biomarkers within a cohort of 363,228 participants. The GWAS of seven CVDs as the outcome data for LDSC and MR are sourced from the FinnGen Release 12 (R12) (Figure S4), a genotype dataset from Finnish biobanks including more than 500,000 individuals.

### Study population for cross-sectional study

This retrospective study collected patients from the Zibo Central Hospital and Tianjin Medical University Eye Hospital, focusing on individuals who took the OCT or OCTA test between March 2018 and March 2024. The inclusion criteria for patients were as follows: (1) The age range for participants is 40 to 90 years. (2) Patients use OCT or OCTA to measure retinal parameters. (3) The CVD group must be diagnosed with one of the seven CVDs. Exclusion criteria were in one of the following points: (1) Patients with a history of ocular trauma or severe retinal diseases. (2) Patients with coexisting cataracts and retinal or optic nerve conditions affecting visual function assessment. (3) Patients who cannot complete follow-up will be excluded from the study. (4) Patients with severe visual impairment from other causes unrelated to the retina are usually excluded, as this could affect the assessment of the study’s primary endpoints.

We identified 134 CVD participants and 60 HC groups. Subsequently, we excluded 45 CVD and 25 HC participants who lacked information on retina data or some biomarkers and suffered severe complications. After these exclusions, a final cohort of 124 participants was identified. Among them, there were 35 controls, with 25 participants diagnosed with CHD, 11 participants with HF, 21 participants with MI, 10 participants with stroke, 12 participants with CA, and 10 confirmed with AF. The Ethics Committee of Zibo Central Hospital and Tianjin Medical University Eye Hospital approved this study. This study should be considered a pilot study due to the small sample size.

### Instrument selection

We adopted a rigorous approach for selecting IVs, beginning with applying a stringent *p*-value threshold of >5E–08 to pinpoint appropriate SNVs. To ensure the exclusivity of independent variants in our MR analysis, we eliminated variants exhibiting linkage disequilibrium with the most significant SNPs, utilizing a clumping criterion of *r*^2^ < 0.001 and a clumping window spanning 10,000 kb. In the MR analysis, we primarily relied on the inverse variance weighted (IVW) method to estimate causal effects by aggregating data from genetic instruments. Furthermore, we used supplementary techniques, including the MR-Egger intercept test, to evaluate horizontal pleiotropy. We computed the *F*-statistics for each instrument, with values exceeding 10 to indicate adequate instrument strength.

### Genetic correlation analysis

LDSC regression, designed explicitly for analyzing GWAS summary data, provides a robust framework for dissecting genetic correlations among complex diseases and traits. LDSC distinguishes authentic polygenic signals from potential confounding factors, such as cryptic relatedness and population stratification. A genetic correlation that is both statistically significant and quantitatively robust indicates that the observed phenotypic correlation is not solely due to environmental confounders (21). To explore the genetic intersections between exposure and a range of outcome phenotypes, we utilized the LDSC tool publicly available at https://github.com/bulik/ldsc.

### MR analysis

In our two-sample MR study, we assessed the influence of retinal characteristics and biomarkers on CVDs by using the IVW approach with a random-effects model, as implemented in TwoSampleMR version 0.5.6 (available at https://mrcieu.github.io/TwoSampleMR/). Data harmonization and all subsequent analyses were conducted using this exact version of the TwoSampleMR package. Heterogeneity among the studies was evaluated using the ‘mr_heterogeneity()’ function, with a heterogeneity *p*-value (*Q*_pval) below 0.05 indicative of significant heterogeneity. To identify directional pleiotropy, we utilized the MR-Egger intercept test, deeming pleiotropy to be present if the MR-Egger intercept deviated significantly from zero (*p* < 0.05). The strong association of each SNP with the exposure was confirmed by calculating an *F*-statistic, with a threshold of *F* > 10 used to establish significance. In addition, reverse MR analysis was conducted on these retinal characteristics and biomarkers that exhibited causal associations to exclude the possibility of bidirectional interactions between the exposure and outcome.

### Covariates in analysis

The regression models were adjusted for a range of covariates that have previously been associated with retinal characteristics, biomarkers, and CVDs, namely age and gender. Furthermore, the models considered additional factors such as smoking history, alcohol consumption, and other pertinent indicators. The selection of covariates was based on their established relevance to retinal characteristics, biomarkers, and CVD risk, encompassing demographic, metabolic, and lifestyle factors.

### Statistical analysis

In this study, we used EmpowerStats software and logistic regression models to analyze data and evaluate clinical outcomes. The baseline characteristics of the study population were stratified by CVDs and described using appropriate statistical techniques. Continuous variables were presented as mean ±SD and analyzed using weighted linear regression models. We performed multivariate linear regression analysis to investigate the relationship between retinal characteristics, biomarkers, and CVDs, computing beta coefficients and 95% confidence intervals. Three different models were used for multivariate testing: model 1 with no adjustments, model 2 adjusted for gender and age, and model 3 for all pertinent covariates. Smoothed curve fitting was carried out with the same adjustments applied. A threshold effect analysis examined the relationship and potential inflection points between retinal characteristics and biomarkers. A *p*-value of <0.05 was considered statistically significant. We applied a weighting method during the analysis to minimize variability in the dataset.

### The predictive model

The Ethics Committee of Tianjin Medical University Eye Hospital granted research approval. As previously described, patients from various parts of China were enrolled at Tianjin Medical University Eye Hospital from March 2018 to March 2024. The cohort was randomly divided into two subsets: training and validation sets. Statistical analyses were conducted using R software (Version 4.3). To identify key predictive features among risk factors in patients with inherited retinal diseases (IRDs), we used the least absolute shrinkage and selection operator (LASSO) method, which is effective for handling high-dimensional data (22). Features with nonzero coefficients were selected, and a multivariable logistic regression model was constructed incorporating these features (23). Odds ratios, 95% confidence intervals, and *p*-values were computed. The model, designed to predict the risk of CVD, included sociodemographic variables with *p* ≤ 0.05 and all disease- and treatment-related variables. Calibration curves were utilized to assess the accuracy of the predictive model, with a significant test indicating calibration imperfections. Harrell’s C-index was used to measure the model’s discrimination performance, and bootstrapping (with 1,000 resamples) was applied to obtain a corrected C-index. Decision curve analysis evaluated the clinical utility of the nomogram by calculating net benefits across various threshold probabilities, considering the trade-off between true positives and the potential harm of unnecessary interventions. The regularization parameter in the LASSO regression model was selected via 10-fold cross-validation using the minimum mean squared error criterion. The risk prediction model was successfully validated using the validation set data. Calibration curves, decision curve analyses, and bootstrapping techniques collectively confirmed the model’s predictive accuracy.

## Results

### The genetic correlation between retinal characteristics and CVDs

**Figure 2:**
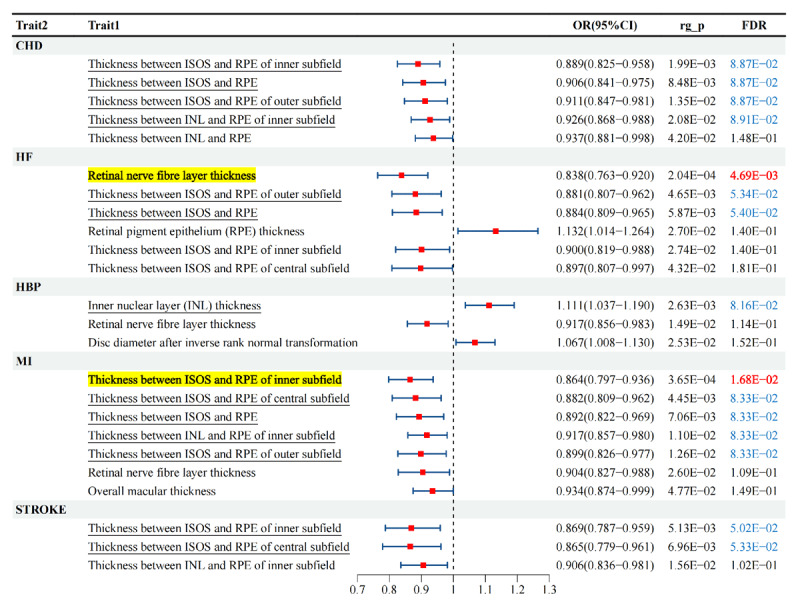
Genetic associations between retinal traits and cardiovascular diseases. Forest plots of MR estimates showing causal associations between different retinal layer thicknesses and risks of major cardiovascular diseases (CHD, HF, HBP, MI, and stroke). Odds ratios (ORs) and 95% confidence intervals (CIs) are presented. Significant associations after false discovery rate (FDR) correction are highlighted, suggesting that retinal structural alterations, particularly in photoreceptor, RPE, and nerve fiber layers, are linked with increased CVD risk.

The SNPs of each retinal characteristic were screened according to the genetic instrument selection process described above. Power calculations were performed for bidirectional MR analyses between CVDs, including HBP, CHF, stroke, AF, HF, MI, and CA. LDSC genetic correlation analysis was conducted to estimate the genetic correlation between different retinal characteristics and CVDs. Based on the results from LDSC, 24 genetic correlations were identified under *p* < 0.05 (Figure 2). In the results, we applied the Benjamini–Hochberg (BH) procedure to correct for pleiotropy. We found that, under the criterion of false discovery rate (FDR) < 0.05, there were genetic associations between RNFL thickness and HF (with OR = 0.838 (0.763–0.920)), as well as between inner segment/outer segment (IS/OS) layer thickness and MI (with OR = 0.864 (0.797–0.936)). In addition, 13 other genetic associations were identified under the criterion of FDR < 0.1. In exploratory studies, a more lenient FDR threshold of less than 0.1 can sometimes be applied (24,25).

### Causal effect of retinal characteristics with CVDs in MR

**Figure 3:**
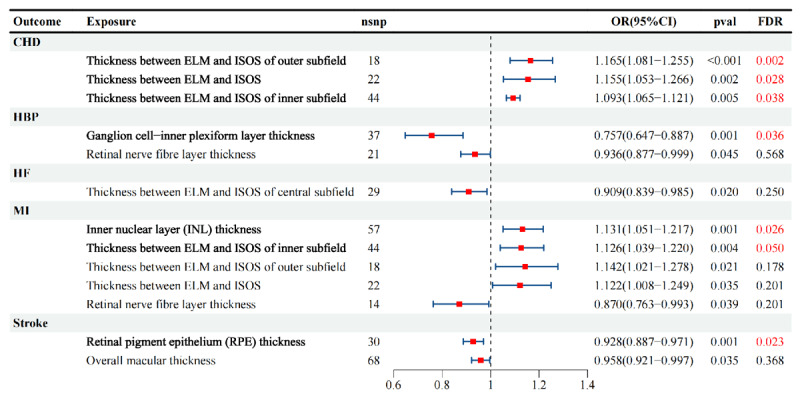
Causal associations between retinal traits and cardiovascular diseases. Forest plots of MR analyses evaluating the causal effects of retinal structural traits (e.g., ELM–ISOS thickness, GC–IPL thickness, RNFL thickness, INL thickness, RPE thickness, and overall macular thickness) on cardiometabolic outcomes. Red squares represent effect sizes (OR), horizontal bars show 95% CIs. Significant results (FDR < 0.05) are marked in red. The findings indicate specific retinal thinning patterns are causally related to CVD susceptibility.

The MR results of the retina on CVDs are listed in Figure 3. In the MR study, the IVW method revealed that genetically determined thickness between external limiting membrane (ELM) and IS/OS [OR = 1.165(1.081–1.255)] was associated with CHD with FDR < 0.05. The GCIPL [OR = 0.757(0.647–0.887)] and RNFL thickness [OR = 0.936(0.877–0.999)] have shown causal effects with HBP with a *p*-value < 0.05 but only GCIPL passed the robust test in FDR < 0.05. The thickness between the ELM and IS/OS [OR = 0.909(0.839–0.985)] has a causal relationship with HF under the threshold of *p* < 0.05; however, after FDR correction, this causal relationship is no longer significant (FDR = 0.25). In MI, the INL thickness [OR = 1.131(1.051–1.217)] and the thickness between the ELM and IS/OS [OR = 1.126(1.039–1.220)] exhibit significant causal relationships, both with FDR < 0.05. However, the causal relationship between RNFL and MI is under the *p* < 0.05 threshold but is no longer significant after FDR correction (FDR = 0.201). The RPE thickness [OR = 0.928(0.887–0.971)] and macular thickness [OR = 0.958(0.921–0.997)] exhibit significant negative causal relationships with stroke, suggesting that the thickness of these two layers may be negatively associated with the onset of stroke. However, the causal relationship between macular thickness and stroke is no longer significant after FDR correction.

### Causal effect of biomarkers with CVDs in MR

**Figure 4:**
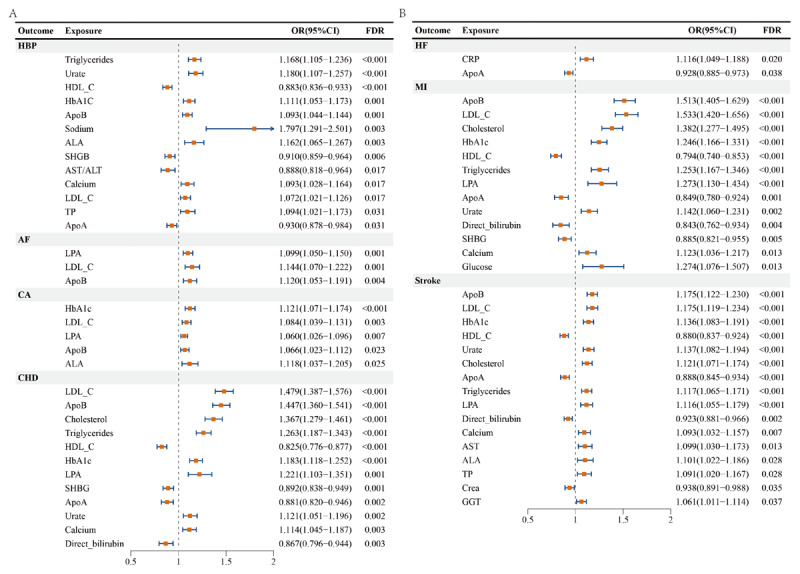
Causal associations between circulating biomarkers and cardiovascular diseases. (A) Mendelian randomization (MR) results for HBP, AF, CA, and CHD. (B) MR results for HF, MI, and stroke. Forest plots display the causal effects of circulating biomarkers—including lipids, glycemic markers, liver/kidney function indicators, and additional biochemical traits—on each cardiovascular outcome. Red squares represent OR estimates with 95% confidence intervals. Associations surviving FDR correction are highlighted. The results underscore the critical roles of lipid metabolism, glucose regulation, and hepatic/renal biomarkers in cardiovascular disease risk across different phenotypes.

We analyzed the causal relationship between 35 biomarkers and CVDs using MR analysis (Figure 4A and B). Of the 35 biomarkers, under the criterion of FDR < 0.05, the IVW method found that 13 biomarkers were significantly associated with HBP, including triglycerides (TG), urate (UA), high-density lipoprotein cholesterol (HDL-C), glycated hemoglobin (HbA1C), apolipoprotein B (ApoB), sodium, alanine aminotransferase (ALA), sex hormone-binding globulin (SHBG), ALA/aspartate aminotransferase (ALA/AST ratio), calcium, low-density lipoprotein cholesterol (LDL-C), total protein (TP), and apolipoprotein A (ApoA). Three biomarkers, lipoprotein A [Lp(a)], LDL-C, and ApoB, exhibit significant causal relationships with AF. HbA1c, LDL-C, Lp(a), ApoB, and ALA exhibit significant causal relationships with CA. In CHD, a total of 12 biomarkers exhibit significant causal relationships, including LDL-C, ApoB, CHO, TG, HDL-C, HbA1c, Lp(a), SHBG, ApoA, UA, calcium, and direct bilirubin (DB). C-reactive protein (CRP) and ApoA exhibit significant causal relationships with HF. While in MI, 13 biomarkers exhibit significant causal relationships, including ApoB, LDL-C, CHO, HbA1c, HDL-C, Lp(a), TG, ApoA, UA, DB, SHBG, calcium, and GLU. A total of 16 biomarkers have been found to exhibit significant causal relationships with stroke, namely ApoB, LDL-C, CHO, HbA1c, HDL-C, Lp(a), TG, ApoA, UA, DB, TP, calcium, GLU, AST, ALA, Crea, and gamma-glutamyl transferase (GGT).

### Baseline characteristics of population-based study

In this investigation, 124 participants were collected according to specific inclusion and exclusion criteria (Table 1, Table S7). The average age of the participants in the control group was 67.2 years, and the CVD group was 65.9 years. Of the control cohort, 42.9% were male and 57.1% were female and the gender of CVD (40.4% male and 59.6% female) cohort. We assessed the relationship between retinal thickness, biomarkers, and 7 CVDs within this demographic.

**Table 1:** The baseline information of seven cardiac vascular disorders, retina layers, and biomarkers. Bold values indicate statistically significant differences (*p* < 0.05).


PHENOTYPE	CONTROL	CHD	*p*	HF	*p*	MI	*p*	STROKE	*p*	CA	*p*	AF	*p*	CVD TOTAL	*p*

*N*	35	25		11		21		10		12		10		89	

AGE	67.2 ± 9.7	65.2 ± 8.6	0.408	70.4 ± 7.3	0.331	65.2 ± 9.6	0.450	66.4 ± 11.7	0.821	66.4 ± 8.9	0.807	63.0 ± 5.8	0.199	65.9 ± 8.9	0.541

GENDER			0.825		0.703		1.000		0.872		0.943		0.872		0.806

Male	15 (42.9%)	10 (40.0%)		4 (36.4%)		9 (42.9%)		4 (40.0%)		5 (41.7%)		4 (40.0%)		36 (40.4%)	

Female	20 (57.1%)	15 (60.0%)		7 (63.6%)		12 (57.1%)		6 (60.0%)		7 (58.3%)		6 (60.0%)		53 (59.6%)	

BP			0.452		0.179		0.143		0.648		0.107		**0.026**		**0.043**

No	16 (45.7%)	9 (36.0%)		3 (27.3%)		6 (28.6%)		4 (40.0%)		3 (25.0%)		2 (20.0%)		27 (30.3%)	

Yes	19 (54.3%)	16 (64.0%)		8 (72.7%)		15 (71.4%)		6 (60.0%)		9 (75.0%)		8 (80.0%)		62 (69.7%)	

ALCOHOL			**0.042**		**0.048**		0.338		0.186		**0.035**		0.776		0.676

No	21 (60.0%)	11 (44.0%)		5 (45.5%)		11 (52.4%)		5 (50.0%)		5 (41.7%)		6 (60.0%)		43 (48.3%)	

Yes	14 (40.0%)	14(56.0%)		6 (55.5%)		10 (47.6%)		5 (50.0%)		7(58.3%)		4(40.0%)		46(51.7%)	

SMOKE			0.759		0.786		0.862		0.315		**0.038**		0.287		0.150

No	17 (48.6%)	11 (44.0%)		5 (45.5%)		10 (47.6%)		6 (60.0%)		8 (66.7%)		5 (50.0%)		45 (50.6%)	

Yes	18 (51.4%)	14 (56.0%)		6 (18.2%)		11(52.4%)		4(40.0%)		4(33.3%)		5 (50.0%)		44(49.4%)	

RNFL	102.7 ± 7.0	90.4 ± 9.6	**<0.001**	100.6 ± 8.7	0.134	88.6 ± 12.8	**<0.001**	89.7 ± 8.0	**<0.001**	98.5 ± 5.1	0.066	100.1 ± 13.2	0.417	93.3 ± 11.1	**<0.001**

GCIPL	85.8 ± 4.7	85.3 ± 5.0	0.723	85.1 ± 8.3	0.438	85.0 ± 4.4	0.445	85.3 ± 3.6	0.511	84.5 ± 4.7	0.422	85.1 ± 5.2	0.698	85.1 ± 5.1	0.275

INL	40.4 ± 3.7	40.8 ± 3.8	0.736	40.1 ± 4.9	0.786	44.5 ± 5.0	**0.002**	41.1 ± 5.4	0.671	40.6 ± 3.8	0.902	40.7 ± 3.9	0.841	41.6 ± 4.6	0.153

OPONL	89.7 ± 6.4	89.5 ± 5.8	0.870	90.1 ± 6.3	0.908	90.1 ± 6.8	0.819	90.7 ± 6.8	0.725	89.8 ± 6.1	0.932	88.6 ± 6.0	0.615	89.8 ± 6.1	0.978

PR-IS/OS	65.2 ± 2.6	69.1 ± 3.2	**<0.001**	64.4 ± 3.0	0.288	70.0 ± 3.7	**<0.001**	65.6 ± 4.1	0.773	64.7 ± 3.3	0.263	67.8 ± 3.9	**0.017**	67.6 ± 4.1	**0.002**

RPE-BM	23.2 ± 2.3	22.2 ± 2.8	0.105	22.3 ± 2.4	0.292	20.5 ± 2.6	**<0.001**	21.2 ± 3.1	**0.015**	23.0 ± 3.1	0.883	22.5 ± 3.5	0.434	21.8 ± 2.9	**0.010**

INNER	228.9 ± 9.3	216.4 ± 10.5	**<0.001**	225.8 ± 17.9	0.131	218.1 ± 14.4	**0.006**	216.1 ± 10.1	**<0.001**	223.6 ± 7.9	0.109	225.9 ± 16.1	0.462	220.0 ± 13.2	**<0.001**

OUTER	178.2 ± 7.1	180.8 ± 6.6	0.159	176.7 ± 7.3	0.527	180.7 ± 7.6	0.189	177.5 ± 9.5	0.460	177.5 ± 7.8	0.723	178.9 ± 8.6	0.787	179.2 ± 7.6	0.526

ALT	24.7 ± 17.3	21.2 ± 7.8	0.341	22.4 ± 7.8	0.718	22.9 ± 12.3	0.624	21.9 ± 12.2	0.967	25.2 ± 13.8	0.678	23.0 ± 12.8	0.773	22.5 ± 10.7	0.872

AST	24.8 ± 13.7	21.4 ± 6.2	0.252	21.4 ± 4.2	0.877	22.2 ± 8.0	0.793	22.4 ± 12.0	0.642	23.0 ± 9.7	0.845	23.1 ± 8.1	0.711	22.1 ± 7.8	0.711

GGT	29.1 ± 10.6	30.8 ± 17.8	0.656	31.7 ± 13.3	0.827	32.5 ± 20.6	0.741	31.1 ± 49.0	**0.005**	32.1 ± 18.4	0.971	30.1 ± 15.1	0.816	31.4 ± 22.7	0.377

TBIL	9.5 ± 4.6	10.4 ± 5.0	0.474	10.4 ± 8.7	0.652	11.7 ± 6.1	0.253	9.8 ± 3.5	0.672	13.2 ± 7.1	0.133	7.9 ± 4.0	0.315	10.7 ± 5.9	0.505

ALB	44.7 ± 2.3	44.7 ± 2.5	0.962	44.5 ± 2.4	0.767	44.8 ± 2.8	0.953	44.1 ± 2.8	0.428	44.3 ± 3.3	0.971	43.6 ± 2.4	0.193	44.5 ± 2.6	0.569

CREA	75.2 ± 15.4	71.6 ± 13.7	0.365	79.1 ± 22.4	0.877	78.6 ± 19.7	0.531	67.2 ± 15.5	0.263	76.6 ± 20.1	0.591	89.6 ± 31.6	**0.050**	76.4 ± 20.3	0.918

UREA	6.0 ± 1.7	6.1 ± 1.6	0.799	6.1 ± 1.5	0.757	6.1 ± 2.1	0.859	5.9 ± 1.5	0.806	6.6 ± 2.9	0.942	7.2 ± 2.9	0.107	6.3 ± 2.1	0.665

UA	303.4 ± 63.7	315.2 ± 69.5	0.499	308.5 ± 101.7	0.908	321.9 ± 88.3	0.526	316.0 ± 45.5	0.495	307.7 ± 52.4	1.000	330.2 ± 72.8	0.261	316.7 ± 73.5	0.361

TC	5.1 ± 1.1	5.8 ± 1.4	**0.032**	5.6 ± 1.6	0.149	5.8 ± 1.4	0.077	5.8 ± 1.0	0.101	5.9 ± 1.5	0.127	5.4 ± 1.2	0.437	5.7 ± 1.3	**0.015**

TG	1.4 ± 0.7	1.6 ± 1.0	0.365	1.5 ± 0.5	0.511	1.7 ± 0.8	0.094	1.5 ± 0.5	0.287	1.7 ± 0.3	**0.019**	1.8 ± 0.8	0.147	1.6 ± 0.7	**0.047**

HDL_C	1.2 ± 0.4	1.0 ± 0.3	**0.018**	1.3 ± 0.3	0.519	1.1 ± 0.2	0.243	1.0 ± 0.3	0.275	1.1 ± 0.3	0.550	1.1 ± 0.4	0.613	1.1 ± 0.3	0.169

LDL_C	2.0 ± 0.8	3.1 ± 0.7	**<0.001**	2.5 ± 0.5	**0.039**	2.4 ± 0.6	0.074	2.7 ± 0.5	**0.006**	2.4 ± 0.5	0.097	2.5 ± 0.5	0.081	2.7 ± 0.6	**<0.001**

Lp(a)	150.2 ± 187.6	201.1 ± 257.1	0.379	308.6 ± 274.5	0.092	179.2 ± 111.2	**0.050**	177.3 ± 139.9	0.072	230.6 ± 247.4	0.414	166.5 ± 130.8	0.799	206.6 ± 206.5	0.062

APOA	1.1 ± 0.3	1.0 ± 0.3	0.068	1.0 ± 0.2	0.455	1.0 ± 0.3	0.326	0.9 ± 0.3	**0.020**	1.1 ± 0.3	0.942	1.0 ± 0.2	0.119	1.0 ± 0.3	0.055

APOB	0.8 ± 0.2	0.9 ± 0.3	0.081	0.9 ± 0.3	0.381	0.9 ± 0.3	0.101	1.1 ± 0.2	**0.002**	0.8 ± 0.1	0.200	0.8 ± 0.2	0.969	0.9 ± 0.3	**0.038**

GLU	5.6 ± 1.0	5.2 ± 0.8	0.179	5.3 ± 0.5	0.220	5.5 ± 0.9	0.846	5.7 ± 1.2	0.521	6.2 ± 2.5	1.000	5.8 ± 1.4	0.580	5.6 ± 1.3	0.507

ALP	80.7 ± 21.0	78.2 ± 17.4	0.636	82.1 ± 13.6	0.562	80.8 ± 29.9	0.594	80.4 ± 21.4	0.935	78.3 ± 13.9	0.903	79.5 ± 25.0	0.881	79.7 ± 21.0	0.879


**Figure 5:**
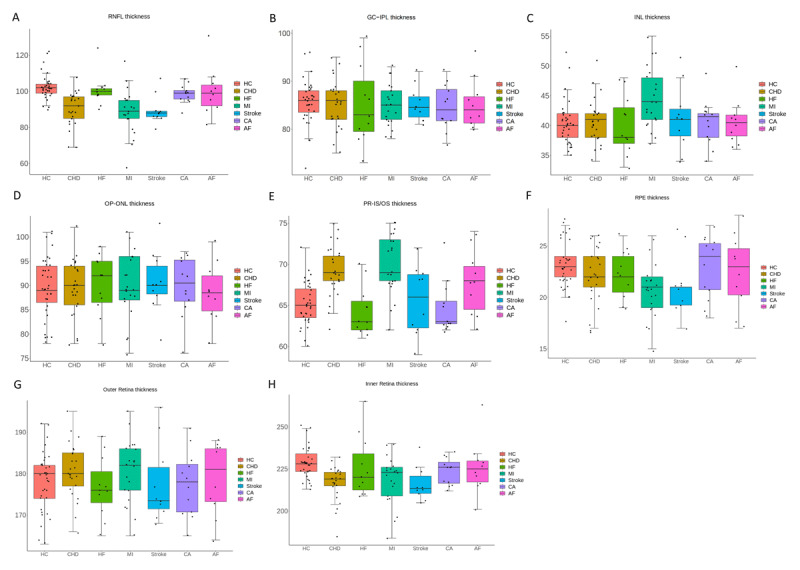
Retinal thickness differences between CVD patients and healthy controls. Box plots Box plots illustrate differences in retinal thickness among healthy controls (HC) and patients with six CVD conditions: CHD, HF, MI, stroke, CA, and AF. (A) RNFL thickness; **(B)** GC–IPL thickness; **(C)** INL thickness; **(D)** OP–ONL thickness; **(E)** PR–IS/OS thickness; **(F)** RPE thickness; **(G)** Outer retina thickness; **(H)** Inner retina thickness. Across multiple retinal layers, patients with CVDs exhibit altered thickness profiles compared with healthy controls, with notable thinning observed in structural layers such as the RNFL, GC–IPL, and RPE.

Regarding retinal characteristics (Figure 5A–H), significant differences were observed in RNFL thickness between CHD (90.4 μm), MI (88.6 μm), stroke (89.7 μm), and HC (102.7 μm). The thickness of the photoreceptor inner segment/outer segment (PR-IS/OS) layer is significantly thicker in the CHD (69.1 μm) and MI groups (70.0 μm) compared to the HC group (65.2 μm). In comparison, the thickness of the RPE-BM layer in the MI group (20.5 μm) and stroke group (21.2 μm) is significantly thinner compared to the HC group (23.2 μm). The inner retinal layers, consisting of the inner limiting membrane (ILM), RNFL, GCL, IPL, and INL, are significantly thinner in the CHD, MI, and stroke groups compared to the HC group. However, the thickness of the outer retinal layers, consisting of the OPL, ONL, ELM, PR-IS/OS, and RPE layers, does not show significant differences among the seven CVD groups and the HC group.

Regarding biomarkers, we found that CHO and TG show significant differences between the seven CVD groups and the HC group in the overall statistics, with levels significantly higher in the CVD groups compared to the HC group. LDL-C in the CVD groups is also considerably higher than in the HC group, with significant differences observed in the CHD, HF, and stroke subgroups. In addition, biomarkers such as HDL-C, ApoA, ApoB, and Lp(a) show varying degrees of significant differences between the CVD and HC groups, suggesting that these biomarkers are closely associated with CVD. In addition, alcohol use showed substantial differences in the CHD, HF, and CA groups, with a higher proportion of participants who consumed alcohol in these groups compared to the HC group. This suggests that alcohol use may increase the risk of these conditions.

### Association between retina layers thickness, biomarkers, and blood pressure

HBP is classified into three grades based on blood pressure levels: Grade 1 (mild) with systolic blood pressure ranging from 140 to 159 mmHg and diastolic blood pressure from 90 to 99 mmHg; Grade 2 (moderate) with systolic pressure from 160 to 179 mmHg and diastolic pressure from 100 to 109 mmHg; and Grade 3 (severe) with systolic pressure of 180 mmHg or higher and diastolic pressure of 110 mmHg or higher (Table 2). We analyzed the relationship between the thickness of retinal layers and the HBP subgroups (Table S2). We found significant differences in the RNFL, GCIPL layer, and inner retina layer between the groups, with the thinning of these retinal layers observed as HBP worsened. In addition, CHO showed significant differences between the subgroups, with levels significantly increasing as HBP worsened.

**Table 2:** The association between retina layers thickness and blood pressure subgroups.


PHENOTYPE	CONTROL	HBP1	HBP2	HBP3	*p*

*N*	43	43	25	13	

AGE	67.6 ± 9.7	67.2 ± 8.7	64.4 ± 8.7	62.5 ± 8.5	

GENDER					0.700

Male	20 (46.5%)	18 (41.9%)	8 (32.0%)	5 (38.5%)	

Female	23 (53.5%)	25 (58.1%)	17 (68.0%)	8 (61.5%)	

ALCOHOL					0.301

No	26 (60.5%)	20 (46.5%)	10 (40.0%)	8 (61.5%)	

Yes	17 (39.5%)	23 (53.5%)	15 (60.0%)	5 (38.5%)	

SMOKE					0.282

No	24 (55.8%)	24 (55.8%)	9 (36.0%)	5 (38.5%)	

Yes	19 (44.2%)	19 (44.2%)	16 (64.0%)	8 (61.5%)	

**RNFL**	100.1 ± 12.3	96.7 ± 9.3	90.6 ± 12.0	87.3 ± 5.1	**<0.001**

**GCIPL**	88.2 ± 4.7	84.7 ± 4.6	81.6 ± 4.0	78.2 ± 5.1	**<0.001**

INL	41.9 ± 4.8	40.9 ± 4.5	39.8 ± 3.2	43.2 ± 4.3	0.151

OPONL	89.4 ± 5.8	90.7 ± 6.3	88.8 ± 6.3	90.0 ± 6.7	0.552

PR.IS.OS	67.0 ± 4.0	66.5 ± 3.6	67.5 ± 4.2	66.8 ± 3.8	0.837

RPE.BM	22.2 ± 3.0	22.0 ± 2.5	22.0 ± 2.9	23.5 ± 2.9	0.314

**INNER**	228.7 ± 12.6	222.3 ± 10.3	213.4 ± 12.8	220.0 ± 10.6	**<0.001**

OUTER	178.6 ± 7.7	179.2 ± 7.4	178.4 ± 8.0	180.3 ± 7.1	0.817

**TC**	5.4 ± 1.3	5.4 ± 1.2	5.7 ± 1.0	6.7 ± 1.5	**0.025**

TG	1.5 ± 0.7	1.6 ± 0.8	1.4 ± 0.6	2.0 ± 0.8	0.099

HDL_C	1.1 ± 0.3	1.1 ± 0.3	1.1 ± 0.3	1.2 ± 0.4	0.827

LDL_C	2.4 ± 0.7	2.5 ± 0.8	2.6 ± 0.8	2.4 ± 0.9	0.789

Lp(a)	188.8 ± 213.7	179.5 ± 207.0	199.2 ± 199.7	217.4 ± 167.9	0.618

APOA	1.0 ± 0.2	1.1 ± 0.3	1.0 ± 0.2	0.9 ± 0.4	0.261

APOB	0.9 ± 0.3	0.8 ± 0.2	0.9 ± 0.3	0.8 ± 0.2	0.356

GLU	5.7 ± 1.4	5.4 ± 1.1	5.7 ± 1.1	5.5 ± 1.2	0.782


Grade 1 HBP: 130–139/80–89 mmHg. Grade 2 HBP: 140–159/90–99 mmHg. Grade 3 HBP: ≥160/100 mmHg. Bold values indicate statistically significant differences (*p* < 0.05).

### Association between retinal layer thickness and biomarkers

We analyzed the association between retinal layer thickness and various biomarkers, including CHO, TG, HDL-C, LDL-C, ApoA, and ApoB. We found a significant association between retinal thickness and CHO levels (Table 3, Table S3). The RNFL thickness thinned as CHO increased (101.0 μm, 94.9 μm, 90.1 μm, 86.9 μm), and the thickness of the RPE-BM layer (23.1 μm, 22.1 μm, 22.2 μm, 20.4 μm) also showed an overall thinning trend as CHO levels increased. The inner retinal layers exhibited a similar trend.

**Table 3:** The association between retina layers thickness and total cholesterol.


TC	Q1	Q2	Q3	Q4	*p*-VALUE

*N*	30	32	29	33	

AGE	68.9 ± 9.5	65.8 ± 8.8	66.7 ± 9.5	63.9 ± 8.4	0.178

GENDER					0.788

Male	11 (36.7%)	12 (37.5%)	14 (48.3%)	14 (42.4%)	

Female	19 (63.3%)	20 (62.5%)	15 (51.7%)	19 (57.6%)	

**RNFL**	101.0 ± 14.9	94.9 ± 9.1	90.1 ± 8.9	86.9 ± 7.0	**<0.001**

GCIPL	84.1 ± 5.5	83.9 ± 4.2	85.6 ± 5.9	84.8 ± 6.5	0.653

INL	40.0 ± 3.0	41.7 ± 4.5	41.9 ± 4.8	41.3 ± 5.0	0.328

OPONL	90.0 ± 6.2	89.8 ± 5.4	89.2 ± 7.0	90.1 ± 6.1	0.955

PR-IS/OS	67.4 ± 3.8	67.1 ± 3.8	66.0 ± 3.9	67.4 ± 4.0	0.484

**RPE-BM**	23.1 ± 2.0	22.1 ± 2.7	22.2 ± 2.5	20.4 ± 3.2	**0.001**

**INNER**	225.1 ± 17.6	220.5 ± 9.3	217.6 ± 10.0	213.1 ± 10.7	**0.002**

OUTER	180.5 ± 7.6	178.9 ± 7.2	177.5 ± 7.5	177.9 ± 8.4	0.448


**Figure 6:**
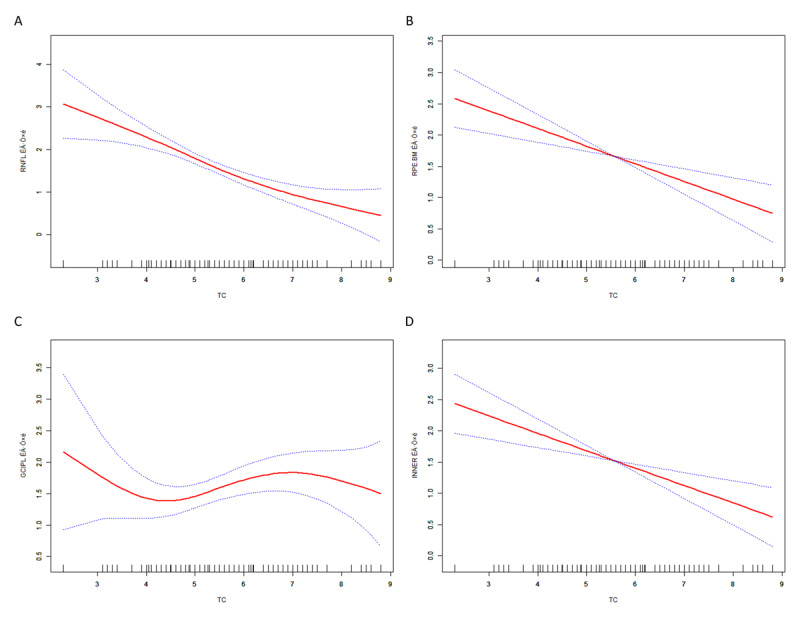
Nonlinear relationships between serum cholesterol and retinal layer thickness. Restricted cubic spline (RCS) models were used to explore the dose–response relationship between TC levels and the thickness of four retinal layers. **(A)** RNFL thickness; **(B)** RPE thickness; **(C)** GC–IPL thickness; **(D)** Inner retina thickness. Red curves represent the fitted nonlinear associations, and blue dashed lines denote 95% confidence intervals. The results reveal inverse or nonlinear patterns linking higher cholesterol levels with retinal neurodegeneration across multiple retinal layers.

We used smooth curves to demonstrate the relationship between the thickness of different retinal layers and CHO levels (Figure 6A–D). We found that RNFL, RPE-BM, and inner retina all exhibited a linear negative correlation with CHO levels, with the thickness of these layers thinning as CHO levels increased. However, the relationship between the GCIPL layer and CHO levels displayed an S-shaped curve, where the thickness initially decreased, then increased, and ultimately reduced again as CHO levels rose.

### Development of a prediction model for CVDs

**Figure 7:**
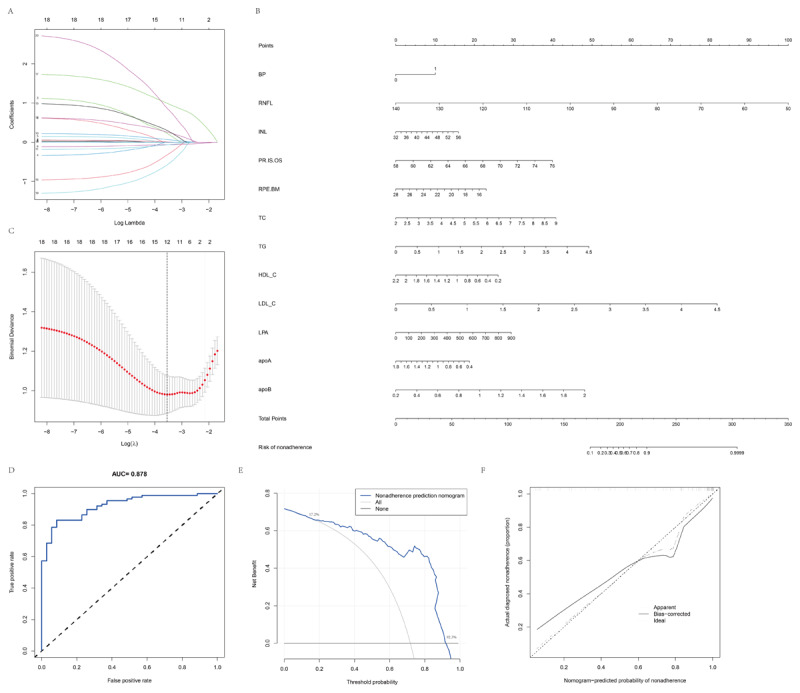
Prediction model for cardiovascular disease risk using retinal traits and biomarkers. **(A)** LASSO coefficient profiles of all candidate features. **(B)** Ten-fold cross-validation for optimal parameter (lambda) selection in the LASSO model. **(C)** Nomogram developed from the selected predictors, incorporating retinal layer thickness and circulating biomarkers. **(D)** Receiver operating characteristic (ROC) curve for the nomogram, showing discrimination performance (AUC = 0.878). **(E)** Decision curve analysis (DCA) evaluating the net clinical benefit across threshold probabilities. **(F)** Calibration plot comparing predicted vs. observed risk, demonstrating good model calibration. Overall, the LASSO-based nomogram shows strong discrimination, clinical utility, and calibration, supporting the predictive value of combining retinal imaging features with systemic biomarkers in assessing cardiovascular disease risk.

This study divided the training cohort into CVD and HC groups. Among the demographic, disease, retinal characteristics, and biomarkers, 20 features were identified as potential predictors (Figure 7A and C) with nonzero coefficients in the LASSO regression model. Logistic regression analysis used the variables BP, RNFL, INL, PR-IS/OS, RPE-BM, CHO, TG, HDL-C, LDL-C, Lp(a), ApoA, and ApoB. A prediction model incorporating these independent predictors was developed and visualized as a nomogram (Figure 7B). The calibration curve for the risk nomogram demonstrated strong agreement within the cohort (Figure 7F). The C-index for the prediction model was 0.924, and this was validated with a bootstrapping cohort, which produced a value of 0.842, indicating excellent discriminatory ability. The risk nomogram showed robust predictive performance, confirming its reliability in disease risk. Furthermore, the receiver operating characteristic (ROC) curve was used to calculate the model’s True Positive Rate and False Positive Rate, with a final area under the curve(AUC) of 0.878, highlighting the model’s high clinical value (Figure 7D). The decision curve analysis for the nomogram is shown in Figure 7E. The decision curve revealed that when the threshold probability is between 17.2% and 92.3%, the nomogram provides more significant benefits than other schemes. Within this range, the net benefit was comparable to other approaches based on the risk nomogram.

## Discussion

This study investigated the complex relationship between retinal characteristics, biomarkers, and CVD. The results indicated that changes in the thickness of different retinal layers, such as RNFL, GCIPL, and ELM-IS/OS, have significant causal relationships with CVD and are closely associated with the risk of CVD onset. Many biomarkers, such as CHO, TG, and LDL-C, also exhibit significant associations with CVD. In addition, significant correlations are found between retinal layer thickness and factors like hypertension and CHO, which are similarly significantly associated with CVD.

We observed that in LDSC and MR analyses, the RNFL thickness was associated with CHD. In the clinical retrospective study, the RNFL thickness was thinner in the CHD group than in the HC group. Some researchers suggest that changes in cardiac function in patients with CHD may affect microcirculation, particularly reducing retinal blood flow perfusion, which could lead to thinning of the RNFL (26). The RNFL has been observed to have a negative causal relationship with disease risk in HF, MI, and HBP, and significant thinning of the RNFL layer has been confirmed in both the MI and stroke cohorts. This may also be related to retinal microcirculation ischemia, which leads to reduced blood flow, further reducing the blood supply to the RNFL and ultimately resulting in atrophy (27). However, the thickness between ELM and IS/OS showed a significant genetic association and a positive causal relationship with CHD and MI, suggesting that the thickness of the PR-IS/OS layer may increase the risk of CHD and MI. In the clinical cohort, we observed that the thickness of the PR-IS/OS layer was significantly thicker in both the MI and CHD groups compared to the HC group. The mechanism underlying the thickening of the PR-IS/OS layer is not yet precise, and no previous studies have reported this finding. We speculate that it may be related to retinal microcirculation dysfunction, which leads to ischemia, resulting in retinal hypoxia and inadequate nutrient supply. This could cause compensatory proliferation or exudation of photoreceptor cells, leading to thickening of this layer. The RPE-BM layer was observed to have a significant genetic association with MI and stroke in LDSC, while in MR, RPE-BM was found to have a significant negative causal relationship with stroke. In the clinical cohort, we observed that the RPE thickness in the MI and stroke groups was significantly thinner compared to the HC group, which is consistent with the results from LDSC and MR. This may be related to retinal blood flow insufficiency leading to hypoxia, which causes degeneration and damage to RPE cells (28). Systemic inflammatory responses lead to apoptosis and functional decline of RPE cells, resulting in reduced thickness (29,30). In addition, oxidative stress caused by hypoxia and inflammation further damages the structure and function of RPE cells, leading to apoptosis and decreased thickness (31). While MR is a powerful strategy to probe causality, it estimates the effect of lifelong, genetically proxied differences in traits and does not by itself prove clinical causation. In the vascular context, it is more clinically plausible that retinal structural changes reflect systemic microvascular and atherosclerotic processes occurring in medium and large arteries, rather than retinal damage directly causing cardiovascular events. Our findings therefore support shared etiologic pathways between retinal layers and CVD risk and motivate the use of retinal metrics as accessible markers of systemic vascular health. We explicitly state the three MR assumptions (relevance, independence, and exclusion restriction) report instrument strength, heterogeneity, and MR-Egger intercept tests, and emphasize that residual horizontal pleiotropy and unmeasured confounding cannot be entirely excluded. Triangulation across LDSC, MR, and cross-sectional evidence strengthens the inference, but the results are not definitive.

We found that CVDs are closely associated with a range of biomarkers, especially those related to lipid metabolism, such as CHO, TG, HDL-C, LDL-C, ApoA, ApoB, Lp(a), and other biomarkers strongly correlated with CVD. The standard structure and function of the retina are also closely related to lipid metabolism (32). Our study found a significant correlation between the thickness of specific retinal layers and CHO levels. The thickness of the RNFL and RPE layers decreases as CHO levels increase. Several previous studies have shown that an increase in CHO is associated with thinning of the RNFL (33). CHO has also been associated with the structure, function, and metabolism of the RPE (34). Our study comprehensively analyzed the relationship between the thickness of various retinal layers and CHO levels. The RNFL, RPE, and inner retina thinned linearly with increasing CHO levels. However, the relationship between GCIPL and CHO levels was more complex, exhibiting an S-shaped curve. Specifically, at low CHO levels, GCIPL thickness decreased as CHO levels increased, but as CHO levels continued to rise, GCIPL thickness increased. However, at high CHO levels, GCIPL thickness began to thin again. The complex relationship between GCIPL and CHO suggests that CHO plays multiple biological roles in the retina, and further experiments are needed to validate this.

We also studied the relationship between retinal thickness and HBP. HBP affects the eye by causing hypertensive retinopathy, which includes retinal arteriolar narrowing, arteriovenous nicking, retinal hemorrhages, microaneurysms, and, in severe cases, optic disc and macular edema (35). Previous research found that RNFL, GCL, and PR-OS thickness were inversely, and INL thickness was positively, associated with higher blood pressure. In contrast, the thickness of the other retinal layers was not significantly correlated with blood pressure (36). In this study, we found that as blood pressure increased, the thickness of the RNFL, GCIPL, and inner retinal layers gradually decreased in the clinical cohort. This is entirely consistent with the MR analysis, where the thickness of the GCIPL and RNFL layers showed a negative causal relationship with HBP, strongly supporting the reliability of the results and suggesting that the thickness of the RNFL, GCIPL, and inner retinal layers is significantly associated with hypertension.

Nomograms are currently extensively utilized as prognostic instruments in medicine, owing to their user-friendly interfaces, enhanced precision, and explicit prognostic results facilitating clinical decision-making (37). Our study represents the pioneering effort to apply a nomogram for predicting CVD risk based on retinal layer thickness and biomarkers. By incorporating demographic and disease risk factors into a user-friendly nomogram, this tool facilitates personalized predictions of CVD risk. Internal cohort validation has demonstrated robust discrimination and calibration capabilities, with a high C-index obtained from interval validation, attesting to the tool’s broad applicability and accuracy, thanks to its large sample size (38). In our model, factors such as BP, RNFL thickness, and various pertinent biomarkers are assigned specific scores. By computing each patient’s score, we can accurately predict their likelihood of developing CVD. Patients with a score exceeding 225 face over a 90% risk of developing CVD, while those with a score above 305 have over a 99% risk. Consequently, this model is well-suited for routine check-ups and assessments in the context of CVD, providing timely guidance for predicting CVD risk. The predictive model demonstrated high accuracy in differentiating cases, as reflected by the ROC curves and AUC values. These findings suggest that retinal imaging could be a potential non-invasive tool for early cardiovascular risk stratification. Given the accessibility of retinal imaging in routine ophthalmic examinations, integrating such predictive models into clinical practice may provide additional insights for early detection and intervention. However, further validation in larger, multi-center cohorts must confirm its generalizability and real-world applicability.

### Study Limitations

The current study has several limitations: (1) A notable limitation is the exclusive use of GWAS data sourced from the European population, which may not adequately represent the genetic diversity across other ethnicities and races globally. (2) Our cross-sectional study included 89 patients in the CVD group, constituting a mere fraction of the total CVD population. This issue is particularly pronounced in the HF, AF, CA, and stroke subgroups, each comprising only 10–12 participants, thereby increasing the risk of data bias. Future clinical studies must enroll significantly larger sample sizes to unravel the intricate relationship between retinal thickness and CVDs. (3) Some relevant covariates may have been overlooked in our multiple regression analysis. (4) Although we endeavored to identify and exclude outlier variants, the possibility of horizontal pleiotropy influencing our findings cannot be discounted entirely. (5) Another significant limitation stems from our reliance on GWAS data primarily from European populations, supplemented by a Chinese cohort with limited sample sizes for specific CVD categories, especially HF, AF, CA, and stroke. This ethnic-specific focus restricts the generalizability of our results to broader populations. Furthermore, the small sample size may diminish statistical power and elevate variability. To address these concerns, future studies should prioritize the utilization of larger, more diverse datasets to validate our findings. Consequently, our results should be interpreted with prudence, and future research endeavors should strive to confirm these associations in larger, more heterogeneous populations. This strategy will facilitate the applicability of our findings across different ethnic groups and enhance the overall credibility of the study. Therefore, multi-center epidemiological investigations and genetic engineering experiments involving larger sample sizes and diverse populations are imperative to substantiate our findings further.

## Conclusion

This study used LDSC, MR, and cross-sectional studies to assess the relationships between the retina and CVDs. It was found that RNFL thickness is causally linked to HBP, MI, and possibly stroke. PR-IS/OS layer thickness is associated with CHD, HF, and MI and may relate to AF. RPE thickness has a causal link with stroke and may correlate with MI. RNFL and GCIPL thickness are causally related to HBP grading, while RNFL and RPE thickness are causally linked to CHO levels. Retinal layer thicknesses derived from OCT are promising non-invasive markers of systemic vascular health. Our genetic analyses support shared etiologic pathways connecting retinal microstructure and CVD risk, but do not imply that retinal damage clinically causes cardiovascular events. Broader, multi-ethnic replication and prospective validation are needed prior to clinical translation.

## Data Accessibility Statement

The data presented in this study are available in this article and supplementary materials.

## Additional File

The additional file for this article can be found as follows:

10.5334/gh.1493.s1 Supplementary File. Tables S1–S7.
