# Supplementary material for: Causal Effects Between Retinal Characteristics and Cardiovascular Diseases: Insights from Genetic Correlation, Mendelian Randomization, and Cross-Sectional Study
Source: Glob Heart. 2025 Nov 21;20(1):104. doi: 10.5334/gh.1493 (PMC12636282; doi:10.5334/gh.1493)
Supplement: Supplementary File. — Tables S1–S7. [file gh-20-1-1493-s1.pdf]

Table S1. The STROBE-MR checklist table.
Table S2. The information for GWAS summary data for eye OCT phenotypes.
Table S3. The instrumental variables for eye OCT phenotypes.
Table S4. The information for GWAS summary data for cardiovascular disorders.
Table S5. The instrumental variables for cardiovascular disorders.
Table S6. The Reverse MR result Between Retina and cardiovascular disorders.
Table S7. The Clinical Cohort of cardiovascular disorders.

Table S1. The STROBE-MR checklist table.

Item No.	Section
1	TITLE and ABSTRACT
	INTRODUCTION
2	Background
3	Objectives
	METHODS
4	Study design and data sources
	a)
	b)
	c)
	d)

	e)
5	Assumptions
6	Statistical methods: main analysis
	a)
	b)
	c)
	d)
	e)
7	Assessment of assumptions
8	Sensitivity analyses and additional analyses
9	Software and pre-registration
	a)
	b)
	RESULTS
10	Descriptive data

	a)
	b)
	c)
	d)
11	Main results
	a)
	b)
	c)
	d)
12	Assessment of assumptions
	a)
	b)
13	Sensitivity analyses and additional analyses

	a)
	b)
	c)
	d)
	DISCUSSION
14	Key results
15	Limitations
16	Interpretation
	a)
	b)
	c)
17	Generalizability
	OTHER INFORMATION

18	Funding
19	Data and data sharing
20	Conflicts of Interest

STROBE-MR checklist table

Checklist item
Indicate Mendelian randomization (MR) as the study’s design in the title and/or the abstract if that is a main purpose of the study
Explain the scientific background and rationale for the reported study. What is the exposure? Is a potential causal relationship between exposure and outcome plausible? Justify why MR is a helpful method to address the study question
State specific objectives clearly, including pre-specified causal hypotheses (if any). State that MR is a method that, under specific assumptions, intends to estimate causal effects
Present key elements of the study design early in the article. Consider including a table listing sources of data for all phases of the study. For each data source contributing to the analysis, describe the following:
Setting: Describe the study design and the underlying population, if possible. Describe the setting, locations, and relevant dates, including periods of recruitment, exposure, follow-up, and data collection, when available.
Participants: Give the eligibility criteria, and the sources and methods of selection of participants. Report the sample size, and whether any power or sample size calculations were carried out prior to the main analysis
Describe measurement, quality control and selection of genetic variants
For each exposure, outcome, and other relevant variables, describe methods of assessment and diagnostic criteria for diseases

Provide details of ethics committee approval and participant informed consent, if relevant

Explicitly state the three core IV assumptions for the main analysis (relevance, independence and exclusion restriction) as well assumptions for any additional or sensitivity analysis

Describe statistical methods and statistics used

Describe how quantitative variables were handled in the analyses (i.e., scale, units, model)

Describe how genetic variants were handled in the analyses and, if applicable, how their weights were selected

Describe the MR estimator (e.g. two-stage least squares, Wald ratio) and related statistics. Detail the included covariates and, in case of two-sample MR, whether the same covariate set was used for adjustment in the two samples

Explain how missing data were addressed

If applicable, indicate how multiple testing was addressed

Describe any methods or prior knowledge used to assess the assumptions or justify their validity

Describe any sensitivity analyses or additional analyses performed (e.g. comparison of effect estimates from different approaches, independent replication, bias analytic techniques, validation of instruments, simulations)

Name statistical software and package(s), including version and settings used

State whether the study protocol and details were pre-registered (as well as when and where)

Report the numbers of individuals at each stage of included studies and reasons for exclusion. Consider use of a flow diagram

Report summary statistics for phenotypic exposure(s), outcome(s), and other relevant variables (e.g. means, SDs, proportions)

If the data sources include meta-analyses of previous studies, provide the assessments of heterogeneity across these studies

For two-sample MR:

i. Provide justification of the similarity of the genetic variant-exposure associations between the exposure and outcome samples

ii. Provide information on the number of individuals who overlap between the exposure and outcome studies

Report the associations between genetic variant and exposure, and between genetic variant and outcome, preferably on an interpretable scale

Report MR estimates of the relationship between exposure and outcome, and the measures of uncertainty from the MR analysis, on an interpretable scale, such as odds ratio or relative risk per SD difference

If relevant, consider translating estimates of relative risk into absolute risk for a meaningful time period

Consider plots to visualize results (e.g. forest plot, scatterplot of associations between genetic variants and outcome versus between genetic variants and exposure)

Report the assessment of the validity of the assumptions

Report any additional statistics (e.g., assessments of heterogeneity across genetic variants, such as I^2, Q statistic or E-value)

Report any sensitivity analyses to assess the robustness of the main results to violations of the assumptions

Report results from other sensitivity analyses or additional analyses

Report any assessment of direction of causal relationship (e.g., bidirectional MR)

When relevant, report and compare with estimates from non-MR analyses

Summarize key results with reference to study objectives

Discuss limitations of the study, taking into account the validity of the IV assumptions, other sources of potential bias, and imprecision. Discuss both direction and magnitude of any potential bias and any efforts to address them

Meaning: Give a cautious overall interpretation of results in the context of their limitations and in comparison with other studies

Mechanism: Discuss underlying biological mechanisms that could drive a potential causal relationship between the investigated exposure and the outcome, and whether the gene-environment equivalence assumption is reasonable. Use causal language carefully, clarifying that IV estimates may provide causal effects only under certain assumptions

Clinical relevance: Discuss whether the results have clinical or public policy relevance, and to what extent they inform effect sizes of possible interventions

Discuss the generalizability of the study results (a) to other populations, (b) across other exposure periods/timings, and (c) across other levels of exposure

Describe sources of funding and the role of funders in the present study and, if applicable, sources of funding for the databases and original study or studies on which the present study is based
Provide the data used to perform all analyses or report where and how the data can be accessed, and reference these sources in the article. Provide the statistical code needed to reproduce the results in the article, or report whether the code is publicly accessible and if so, where
All authors should declare all potential conflicts of interest

Relevant text from manuscript
Detailed in the Title and Abstract sections
Detailed in the Introduction section
Detailed in the Introduction section
Detailed in the Methods: Study Design, Data sources, TableS2-TableS4
Detailed in the Methods: Study Design, Data sources, TableS2-TableS4

Detailed in the Methods

Detailed in the Methods: Statistical analysis and Study Design

Detailed in the Methods: Study Design, Data sources, TableS2-TableS4

Detailed in the Study design and Discusion sections

Detailed in the Methods: MR statistical analysis

Detailed in the Methods: MR statistical analysis

Detailed in the Methods: Ethics

Detailed in the Methods: Study Design, Data sources, TableS2-TableS4

Detailed in the Results, Supplementary tables sections.

Detailed in the Results and Supplementary tables sections.

Detailed in the Methods, Results, and Supplementary tables

Detailed in the Discussion section

Detailed in the Discussion section

Detailed in the Abstract section

Detailed in the Methods: Study Design, Data sources, TableS2-TableS4

Detailed in the Declaration of interests section

Table S2. The information for GWAS summary data for eye OCT phenotypes.

ID	UKB Data-Field	Description	File_name	Description
ELM_ISOS_thickness_left	28520	Thickness between ELM a	ukbiobank	Thickness between ELM and ISOS
ELM_ISOS_thickness_right	28521	Thickness between ELM a	ukbiobank	Thickness between ELM and ISOS
INL_ELM_thickness_left	28512	Thickness between INL ar	ukbiobank	Thickness between INL and ELM
INL_ELM_thickness_right	28513	Thickness between INL ar	ukbiobank	Thickness between INL and ELM
INL_RPE_thickness_left	28536	Thickness between INL ar	ukbiobank	Thickness between INL and RPE
INL_RPE_thickness_right	28537	Thickness between INL ar	ukbiobank	Thickness between INL and RPE
ISOS_RPE_thickness_left	28528	Thickness between ISOS a	ukbiobank	Thickness between ISOS and RPE
ISOS_RPE_thickness_right	28529	Thickness between ISOS a	ukbiobank	Thickness between ISOS and RPE
GCIPL_thickness_left	28504	Ganglion cell-inner plexifo	ukbiobank	Ganglion cell-inner plexiform laye
GCIPL_thickness_right	28505	Ganglion cell-inner plexifo	ukbiobank	Ganglion cell-inner plexiform laye
INL_thickness_left	28502	Inner nuclear layer (INL) t	ukbiobank	Inner nuclear layer (INL) thicknes
INL_thickness_right	28503	Inner nuclear layer (INL) t	ukbiobank	Inner nuclear layer (INL) thicknes
RNFL_thickness_left	28500	Retinal nerve fibre layer t	ukbiobank	Retinal nerve fibre layer thicknes
RNFL_thickness_right	28501	Retinal nerve fibre layer t	ukbiobank	Retinal nerve fibre layer thicknes
Disc_diameter_trans_left	27851	Disc diameter after invers	ukbiobank	Disc diameter after inverse rank
ELM_ISOS_thickness_central_s	28514	Thickness between ELM a	ukbiobank	Thickness between ELM and ISOS
ELM_ISOS_thickness_central_s	28515	Thickness between ELM a	ukbiobank	Thickness between ELM and ISOS
ELM_ISOS_thickness_inner_su	28516	Thickness between ELM a	ukbiobank	Thickness between ELM and ISOS
ELM_ISOS_thickness_inner_su	28517	Thickness between ELM a	ukbiobank	Thickness between ELM and ISOS
ELM_ISOS_thickness_outer_su	28518	Thickness between ELM a	ukbiobank	Thickness between ELM and ISOS
ELM_ISOS_thickness_outer_su	28519	Thickness between ELM a	ukbiobank	Thickness between ELM and ISOS
INL_ELM_thickness_central_su	28506	Thickness between INL ar	ukbiobank	Thickness between INL and ELM
INL_ELM_thickness_central_su	28507	Thickness between INL ar	ukbiobank	Thickness between INL and ELM
INL_ELM_thickness_inner_sub	28508	Thickness between INL ar	ukbiobank	Thickness between INL and ELM
INL_ELM_thickness_inner_sub	28509	Thickness between INL ar	ukbiobank	Thickness between INL and ELM
INL_ELM_thickness_outer_sub	28510	Thickness between INL ar	ukbiobank	Thickness between INL and ELM
INL_ELM_thickness_outer_sub	28511	Thickness between INL ar	ukbiobank	Thickness between INL and ELM
INL_RPE_thickness_central_su	28530	Thickness between INL ar	ukbiobank	Thickness between INL and RPE c
INL_RPE_thickness_central_su	28531	Thickness between INL ar	ukbiobank	Thickness between INL and RPE c
INL_RPE_thickness_inner_sub	28532	Thickness between INL ar	ukbiobank	Thickness between INL and RPE c
INL_RPE_thickness_inner_sub	28533	Thickness between INL ar	ukbiobank	Thickness between INL and RPE c
INL_RPE_thickness_outer_sub	28534	Thickness between INL ar	ukbiobank	Thickness between INL and RPE c
INL_RPE_thickness_outer_sub	28535	Thickness between INL ar	ukbiobank	Thickness between INL and RPE c
ISOS_RPE_thickness_central_s	28522	Thickness between ISOS a	ukbiobank	Thickness between ISOS and RPE
ISOS_RPE_thickness_central_s	28523	Thickness between ISOS a	ukbiobank	Thickness between ISOS and RPE
ISOS_RPE_thickness_inner_sul	28524	Thickness between ISOS a	ukbiobank	Thickness between ISOS and RPE
ISOS_RPE_thickness_inner_sul	28525	Thickness between ISOS a	ukbiobank	Thickness between ISOS and RPE
ISOS_RPE_thickness_outer_su	28526	Thickness between ISOS a	ukbiobank	Thickness between ISOS and RPE
ISOS_RPE_thickness_outer_su	28527	Thickness between ISOS a	ukbiobank	Thickness between ISOS and RPE
mean_of_vertical_disc_diame	27853	Mean of vertical disc diar	ukbiobank	Mean of vertical disc diameter
overall_average_retinal_pigm	27822	Retinal pigment epitheliu	ukbiobank	Retinal pigment epithelium (RPE)

overall_average_retinal_pigm	27823	Retinal pigment epitheliu	ukbiobank	Retinal pigment epithelium (RPE)
overall_macular_thickness_lef	27800	Overall macular thickness	ukbiobank	Overall macular thickness
overall_macular_thickness_rig	27801	Overall macular thickness	ukbiobank	Overall macular thickness
VCDR_left	27857	Vertical cup to disc ratio (	ukbiobank	Vertical cup to disc ratio
VCDR_regressed_left	27855	Vertical cup to disc ratio r	ukbiobank	Vertical cup to disc ratio regresse

S
S

.
.
.
er thickness
er thickness
ss
ss
ss
ss
normal transformation
S of central subfield
S of central subfield
S of inner subfield
S of inner subfield
S of outer subfield
S of outer subfield
of central subfield
of central subfield
of inner subfield
of inner subfield
of outer subfield
of outer subfield
of central subfield
of central subfield
of inner subfield
of inner subfield
of outer subfield
of outer subfield
of central subfield
of central subfield
of inner subfield
of inner subfield
of outer subfield
of outer subfield

) thickness

) thickness

ed and transformed

Table S3. The instrumental variables for eye OCT phenotypes.

chr.exposure	SNP	pos.exposure	effect_allele.e	other_all	samplesize	eaf.exposure	beta.expo
1	rs12747978	3733391	A	G	50182	0.131202	-0.07793
3	rs4647260	37054601	C	T	50154	0.487877	-0.03411
3	rs12497826	47669549	A	T	48159	0.327094	0.04149
4	rs375259	48343550	T	C	49466	0.314297	-0.03986
5	rs10941417	39080660	C	G	49667	0.442024	0.03475
5	rs3828616	71496419	G	A	50345	0.329099	0.05231
6	rs35440822	56665804	T	C	50061	0.184495	-0.05426
7	rs113319029	917352	A	C	49917	0.175171	0.05395
8	rs12544796	10473180	A	G	47423	0.248751	0.05036
10	rs11008690	32208769	A	G	50280	0.48924	-0.04494
10	rs12265809	62653374	C	T	49740	0.231313	0.04798
11	rs78658393	1791414	A	G	50205	0.124828	0.05302
11	rs7116940	66303895	C	T	49721	0.239627	0.05081
12	rs80247972	96270298	T	C	50273	0.192181	-0.0449
14	rs1555211	61025791	T	C	50135	0.292859	-0.04957
14	rs10147158	74465741	A	T	49923	0.460028	0.037
14	rs887595	74666641	A	G	49958	0.17827	-0.11119
14	rs8023039	103777606	A	G	49707	0.489026	-0.05008
16	rs2012649	5064917	T	C	50345	0.259132	-0.04555
16	rs8063535	71754711	C	T	50267	0.471174	0.05723
17	rs55978930	47299789	G	A	49191	0.308979	0.04544
22	rs11913168	30606564	A	G	50345	0.109197	-0.06401
1	rs55768060	113541081	T	C	51114	0.229272	-0.04007
2	rs6710698	172596078	C	G	51342	0.284348	0.04294
2	rs2229814	227954599	G	A	51428	0.500535	0.03403
3	rs6766253	3269481	T	C	50852	0.13379	-0.05806
3	rs17279437	45814094	A	G	51428	0.106693	-0.12516
4	rs2166181	82422327	G	A	50747	0.468097	-0.03721
4	rs11100197	159685743	T	C	51409	0.4269	0.03869
4	rs10212888	184924281	G	A	50447	0.342637	0.05507
5	rs17219543	11543668	G	C	49270	0.449614	0.03532
5	rs1070768	87135896	T	C	50654	0.0676452	-0.08673
5	rs75048985	87847992	A	T	51303	0.0719256	0.16824
6	rs115531193	34651311	A	T	51420	0.113049	0.05514
6	rs1536057	108885623	T	C	51161	0.278493	-0.05766
6	rs12193446	129820038	G	A	51428	0.0956677	0.07102
7	rs11762530	46630602	G	C	50709	0.406673	-0.09368
7	rs13309489	51011324	A	C	50666	0.252586	0.06972
8	rs10955947	121041396	T	C	50836	0.411588	-0.03519
9	rs11145415	71754153	T	C	51262	0.208546	-0.04862
9	rs10760712	102787364	A	C	51355	0.438458	-0.04266

11	rs7478986	17131249	C	T	51381	0.502316	0.03653
11	rs3862368	88529474	A	C	50484	0.325598	-0.04139
11	rs10750291	124706625	A	G	48853	0.321751	-0.03945
12	rs56108400	56213297	T	G	51428	0.239218	0.04865
12	rs56882977	118472388	C	G	50658	0.174918	0.05452
14	rs887595	74666641	A	G	51036	0.178041	0.06278
14	rs8021126	104546296	G	A	46661	0.148218	0.05635
15	rs1800407	28230318	T	C	51428	0.0833106	-0.10674
15	rs524952	35005886	A	T	51396	0.48888	-0.03774
15	rs17197194	89111610	A	G	50694	0.32059	0.03923
17	rs4627402	43932741	T	C	50624	0.219994	0.04996
17	rs12940987	59269257	G	A	51315	0.217734	0.04296
17	rs28855509	65998167	C	T	51256	0.219116	0.04248
17	rs9905786	79602063	G	T	51364	0.356222	-0.09893
18	rs11876004	57038761	G	A	51264	0.181336	-0.05269
19	rs10417071	10872431	G	T	51156	0.471372	0.03432
19	rs55924780	37542693	G	A	51406	0.371426	0.03786
19	rs4803297	40310084	T	C	50846	0.431027	-0.04224
21	rs28582765	41418714	T	G	51140	0.106512	0.05615
21	rs1625244	47364499	T	C	51407	0.278114	-0.0643
1	rs61779064	21351885	T	C	50885	0.0809964	0.06694
1	rs16828071	41783603	A	G	50883	0.229291	0.07374
1	rs1779861	61491275	T	G	50222	0.391303	-0.03529
1	rs4839276	113524639	G	T	50548	0.228535	-0.04428
1	rs35661734	150551295	C	T	50885	0.0390783	-0.17176
1	rs61820950	202709410	C	A	49726	0.455375	-0.03782
1	rs74454622	214107008	T	C	50885	0.0766729	-0.14052
2	rs759460	37548136	T	C	50861	0.081998	-0.0729
2	rs9967780	56234942	T	G	50130	0.212298	0.04853
2	rs2028900	85767735	T	C	50862	0.449255	0.03735
2	rs6727356	111912729	G	A	48194	0.306366	0.04796
2	rs17581439	172737267	C	T	50134	0.263823	0.04718
2	rs2115597	227791218	T	C	49914	0.499369	0.03701
3	rs2118752	45700125	A	G	50821	0.270469	-0.03959
3	rs71330995	123124513	A	G	50464	0.270757	0.04381
3	rs28454055	150181177	G	T	50221	0.168316	-0.05132
3	rs902415	183976108	C	T	50821	0.252238	0.04144
4	rs10019492	32395413	A	G	50467	0.412042	-0.03605
4	rs1838492	82396010	C	G	49780	0.457885	-0.03575
4	rs3811773	184942220	C	T	50313	0.319619	0.04829
5	rs2190989	11584342	C	T	50885	0.452324	0.03776
5	rs10942927	71524163	T	A	50455	0.368051	-0.03686
5	rs1070768	87135896	T	C	50113	0.0676172	-0.12311
5	rs181433574	87402754	T	A	50778	0.0152133	0.16876

5	rs17421627	87847586	G	T	50885	0.0732043	0.37273
5	rs13156539	148606052	C	A	50002	0.467181	0.04981
6	rs17507554	11394287	A	G	50885	0.0498477	0.09959
6	rs217343	84336742	T	C	50567	0.437904	0.08179
6	rs12193446	129820038	G	A	50885	0.0955291	0.09308
6	rs9767105	149980727	G	A	50885	0.352176	0.04665
7	rs7806594	8103283	G	C	49528	0.124828	0.05962
7	rs187136683	27084648	G	A	50424	0.371143	0.04216
7	rs3823729	42198474	T	C	49750	0.341005	-0.03777
7	rs7807296	155617259	C	T	50093	0.429232	-0.06081
7	rs10255584	156237926	G	C	49551	0.318651	0.03882
8	rs1877119	8707197	G	C	50244	0.411651	0.04603
8	rs4449834	61755868	G	T	50425	0.269955	0.04185
8	rs2340768	105153786	G	A	50504	0.194777	-0.0456
9	rs1983810	21552654	C	T	48978	0.3082	0.04336
9	rs28446321	98266855	A	T	50601	0.0894251	0.08053
10	rs4838417	49742205	C	T	50146	0.353029	0.03751
11	rs12575487	17019317	T	C	50792	0.289051	0.04068
11	rs3026390	31813509	T	C	50865	0.484144	-0.04483
11	rs17149785	68933041	T	C	50537	0.0396046	-0.09579
12	rs3138142	56115585	T	C	50537	0.240002	0.06728
12	rs4767656	118495118	C	T	50707	0.234139	0.04549
14	rs28711886	61072658	G	A	48847	0.318464	-0.0813
14	rs887595	74666641	A	G	50494	0.178031	0.09482
14	rs2070598	75360906	A	G	50833	0.45917	-0.03452
15	rs7168800	28341575	A	G	50104	0.0799038	-0.06545
15	rs75638145	31453147	A	G	49274	0.148111	-0.07026
15	rs524952	35005886	A	T	50853	0.489145	-0.03957
15	rs548932774	41438153	A	G	46690	0.336068	-0.04141
15	rs62023489	53076118	C	T	50883	0.112366	-0.05458
15	rs4776131	53610353	A	G	49412	0.42091	0.04086
15	rs690526	54007118	G	T	50742	0.482579	-0.0494
15	rs72763825	89149872	G	A	50579	0.341426	0.04601
16	rs11642954	24824248	A	G	50695	0.194654	-0.04759
16	rs4491516	80548671	A	C	50480	0.260658	0.04215
17	rs4790941	65977132	A	G	50686	0.202729	0.04742
17	rs9905786	79602063	G	T	50820	0.355441	-0.0744
18	rs9952482	6730716	A	C	50254	0.477395	0.03576
18	rs2305041	57054087	T	C	50561	0.175175	-0.05689
19	rs4803297	40310084	T	C	50306	0.431092	-0.04381
22	rs11704728	18130209	T	C	50180	0.193972	-0.06299
1	rs3131705	40417621	C	G	50960	0.325687	-0.03772
1	rs12032906	41769158	C	T	50675	0.219033	0.07515
1	rs72683468	113564178	G	T	51290	0.230503	-0.04856

1	rs35661734	150551295	C	T	51290	0.0390135	-0.19464
1	rs61820950	202709410	C	A	50116	0.455593	-0.03714
1	rs80256218	214110713	T	C	51263	0.0763416	-0.14207
2	rs13399945	56239897	G	A	50585	0.213107	0.05501
2	rs2028900	85767735	T	C	51268	0.44913	0.03979
2	rs6727356	111912729	G	A	48556	0.306255	0.05011
2	rs114352177	143919827	A	C	51290	0.0302593	-0.1
2	rs62182392	172721762	A	G	51100	0.259511	0.05024
2	rs9288464	213929086	A	G	47520	0.244918	-0.04171
2	rs13382950	227956067	C	T	51290	0.494726	-0.03602
3	rs28454055	150181177	G	T	50612	0.168428	-0.04777
3	rs73886573	187500118	G	A	51283	0.0437572	0.08508
4	rs6847123	32446372	C	G	50334	0.433594	-0.03759
4	rs17499741	82421848	A	G	50617	0.467965	-0.04149
4	rs13118227	154759949	G	T	51230	0.497375	-0.03483
4	rs1970896	184943118	C	T	50946	0.320064	0.04903
5	rs2190989	11584342	C	T	51290	0.452047	0.03749
5	rs4704538	71394472	A	G	51109	0.173825	-0.04968
5	rs1070768	87135896	T	C	50517	0.0676406	-0.12279
5	rs181433574	87402754	T	A	51185	0.0151705	0.17692
5	rs17421627	87847586	G	T	51290	0.0731429	0.36943
5	rs4490572	148612322	A	G	48264	0.46053	0.05279
6	rs17507554	11394287	A	G	51290	0.049805	0.11464
6	rs4706108	80697318	G	A	51198	0.335462	-0.03795
6	rs7752421	84317533	T	A	51005	0.435663	0.07849
6	rs12193446	129820038	G	A	51290	0.0956229	0.10013
6	rs2275045	150163911	C	T	50876	0.35046	0.04368
7	rs73674757	8018413	G	C	50824	0.135763	0.05639
7	rs10259123	27083853	A	C	49255	0.343346	0.03965
7	rs3801209	42198515	T	C	50122	0.341407	-0.03822
7	rs7807296	155617259	C	T	50481	0.428785	-0.05613
8	rs907180	8702827	G	A	51290	0.433184	0.04751
8	rs1865304	26233374	G	A	50762	0.355601	0.03671
8	rs140727637	61684249	G	A	51106	0.245275	0.04436
9	rs4141713	21556881	T	C	50073	0.310666	0.04251
9	rs11145124	71702999	T	C	50767	0.319922	-0.03798
9	rs28446321	98266855	A	T	51005	0.0894716	0.06666
10	rs4838417	49742205	C	T	50549	0.353489	0.04012
11	rs7127818	17004090	C	G	51251	0.289536	0.04376
11	rs2239789	31815896	T	A	51290	0.484597	-0.04149
11	rs116233906	68968271	A	C	50869	0.0407124	-0.12148
11	rs4121427	128818599	T	C	51135	0.40088	0.03657
12	rs3138142	56115585	T	C	50939	0.240238	0.06273
12	rs4767656	118495118	C	T	51113	0.234314	0.04301

14	rs2753462	60850703	C	G	51156	0.287806	-0.08058
14	rs113145157	68926765	T	C	50601	0.0879825	-0.06475
14	rs887595	74666641	A	G	50898	0.17819	0.08103
15	rs7168800	28341575	A	G	50495	0.0798594	-0.06514
15	rs75638145	31453147	A	G	49673	0.148149	-0.06715
15	rs524952	35005886	A	T	51258	0.488977	-0.03832
15	rs6492978	41317702	C	T	51136	0.469278	-0.04227
15	rs12900646	53611011	A	G	50028	0.418066	0.03762
15	rs690383	54002267	G	A	51147	0.492072	-0.05155
15	rs72763822	89143949	T	C	51034	0.341949	0.0407
16	rs11642954	24824248	A	G	51099	0.194632	-0.04803
16	rs6564760	80485098	C	G	50872	0.244132	0.04833
17	rs4790941	65977132	A	G	51097	0.202654	0.04653
17	rs9905786	79602063	G	T	51226	0.356186	-0.07455
18	rs9952482	6730716	A	C	50644	0.477115	0.0389
18	rs11876937	57032800	G	A	50975	0.180441	-0.05623
19	rs4803297	40310084	T	C	50707	0.431114	-0.042
22	rs11704728	18130209	T	C	50579	0.194191	-0.05585
22	rs9330813	46364161	A	G	51290	0.312975	0.04505
1	rs34085461	182581157	T	G	48562	0.44436	-0.03647
2	rs62135230	48802819	G	A	46376	0.352984	0.04014
2	rs12104619	145413855	C	A	50602	0.31228	0.03786
2	rs7598246	172866359	T	C	50357	0.414282	0.04242
3	rs17279437	45814094	A	G	50871	0.106554	-0.06312
3	rs62252351	69567372	G	A	50730	0.199517	-0.0516
3	rs149831820	77192591	C	T	50821	0.0623364	-0.07785
5	rs377614	2613654	G	A	50349	0.419055	-0.04792
7	rs12718990	50973786	G	A	50673	0.255965	0.04296
8	rs13261390	74225058	C	G	50602	0.32561	0.03795
8	rs111714439	109122444	C	A	50570	0.256051	-0.0843
10	rs1900003	70004551	C	A	50630	0.234821	-0.08676
11	rs7950415	88899605	C	T	50757	0.47752	0.05019
12	rs1561091	96197201	T	G	50814	0.224849	-0.04899
14	rs7148979	74547318	A	T	50838	0.119507	0.10807
14	rs118186707	74686575	A	G	50394	0.0285848	-0.11329
17	rs112364254	79578287	A	G	50761	0.355657	-0.0724
1	rs12132529	182580329	C	G	49798	0.406643	-0.03986
1	rs502937	183335495	C	T	50982	0.422875	-0.03501
1	rs143289658	214233838	G	A	51295	0.0209475	-0.12136
2	rs2271758	172701157	T	G	50980	0.41172	0.04271
3	rs4327428	45809329	A	C	51240	0.120248	-0.06604
3	rs62252351	69567372	G	A	51167	0.200002	-0.06231
5	rs377614	2613654	G	A	50797	0.418834	-0.04503
7	rs10269822	17696399	C	A	50956	0.271322	0.03906

7	rs71540282	51008649	T	C	51002	0.246951	0.04425
8	rs617117	109114464	C	T	49921	0.246099	-0.08667
9	rs490333	128354569	T	C	51169	0.421925	0.03469
10	rs61854803	70014638	C	T	51245	0.259752	-0.07533
11	rs12574166	69291285	T	C	50973	0.152993	0.05558
11	rs7949891	88907162	G	A	50868	0.423803	0.04214
12	rs11561397	46428974	A	G	50949	0.377878	-0.03625
14	rs1010053	61005625	G	A	51139	0.293015	-0.04265
14	rs11844841	61050397	C	A	50779	0.127651	-0.05379
14	rs887595	74666641	A	G	50917	0.177946	0.1027
14	rs118186707	74686575	A	G	50829	0.0286156	-0.10254
15	rs1348002	89113138	C	G	50538	0.325626	0.04099
17	rs1518774	61287445	T	G	51222	0.496798	0.03458
17	rs112364254	79578287	A	G	51202	0.356363	-0.05916
1	rs12024620	3049362	T	C	45894	0.0640716	0.08394
1	rs1192415	92077097	G	A	45923	0.187814	0.1633
1	rs12137699	116209762	T	C	45817	0.236386	0.06727
1	rs11589479	155033308	A	G	45923	0.16287	-0.04916
1	rs114537871	227560794	A	G	45801	0.0758062	-0.1318
2	rs142164320	19432726	C	G	45878	0.0134269	-0.19343
2	rs4832012	86000500	C	G	45205	0.512377	0.0453
2	rs980772	145442190	G	T	45923	0.328147	-0.04103
3	rs4858678	25047446	G	A	45840	0.284599	0.04117
3	rs6787363	25388569	A	G	45923	0.375803	0.05597
3	rs17398137	100625703	A	G	45923	0.182066	-0.07135
4	rs74764079	81952637	A	T	45701	0.0263123	-0.14027
4	rs1566795	112375172	C	T	45422	0.356512	-0.04595
5	rs72759609	31952051	C	T	45662	0.100861	-0.09315
5	rs251526	52586585	G	A	45819	0.0790284	0.06861
5	rs77443179	87826858	G	A	45635	0.0855812	0.07555
6	rs1520	39526867	G	A	45746	0.37506	0.04233
6	rs7744813	73643289	C	A	44364	0.410908	0.04711
6	rs117131686	122680085	G	A	45624	0.296467	-0.04937
6	rs1313696	127052113	G	A	45401	0.503414	-0.0452
8	rs4876271	1901930	A	G	45923	0.126647	0.06048
8	rs1905014	13407297	C	T	45443	0.428053	0.03825
8	rs10957177	61911070	G	A	44593	0.247864	-0.04262
8	rs9297976	143752235	C	T	44870	0.43207	0.04243
9	rs7866671	89275519	A	G	45899	0.336935	0.0397
9	rs76709848	98313349	G	A	45100	0.102871	0.06569
9	rs56072648	134560083	G	A	45871	0.268557	-0.04697
10	rs952917	25055291	T	G	45383	0.329903	-0.04243
10	rs3858146	70013250	T	C	45885	0.258919	-0.17263
10	rs7893954	104318966	G	A	45923	0.289713	-0.04468

11	rs1448941	30848413	C	T	45923	0.42114	-0.03749
11	rs2180950	31540767	G	A	45856	0.229774	0.05597
12	rs11051145	31050201	T	C	44971	0.159114	-0.07
12	rs61952220	83959234	C	A	45110	0.450111	-0.0506
13	rs9534439	47192049	T	C	45745	0.190425	0.05422
14	rs2761882	54420309	T	C	44362	0.496416	-0.03967
14	rs12147505	60660734	C	T	45822	0.0120139	-0.31334
14	rs12436074	85727904	G	A	45704	0.443101	0.04938
16	rs7196852	51462823	G	A	45691	0.30239	0.06226
17	rs11079418	59239754	T	G	45869	0.186608	0.07666
17	rs9905786	79602063	G	T	45865	0.356819	-0.0578
18	rs11083244	25643844	C	T	45211	0.273739	-0.04421
22	rs1972202	29100564	A	G	45896	0.358855	-0.06326
22	rs6000765	37920993	T	C	45782	0.246156	0.061
22	rs9330814	46364191	T	C	45620	0.31005	-0.04181
1	rs4507956	3769262	T	C	51561	0.268401	-0.04053
1	rs2235547	23406485	C	G	51626	0.483836	0.04923
1	rs16825939	39662508	A	G	51463	0.210093	0.04696
1	rs4587551	90289861	A	G	51626	0.0632336	-0.09464
1	rs1513617	227447213	C	T	51527	0.336115	0.04343
2	rs6749990	77836998	C	T	50806	0.426879	-0.03448
3	rs6763422	150170180	T	C	51187	0.239807	0.08931
3	rs6790999	160386249	G	T	51626	0.028445	0.10797
4	rs34417820	48460975	T	C	51428	0.255872	-0.03974
4	rs6851970	76819129	C	A	51492	0.19537	0.04333
5	rs10940160	67563227	A	T	51522	0.470838	0.03677
6	rs9267577	31813774	T	C	51535	0.18664	0.04808
6	rs1132635	123046922	G	A	51516	0.442018	-0.03729
7	rs6460896	12252659	A	G	51410	0.419656	-0.08433
7	rs2693730	129421674	C	G	50814	0.439682	-0.0493
9	rs141629142	77230421	T	G	51421	0.0154606	0.15031
9	rs10751482	134372232	C	T	51533	0.190266	0.04754
10	rs1409396	22879032	G	A	50792	0.444283	-0.05446
10	rs11817531	85981351	A	G	51606	0.231359	0.04223
10	rs36098736	124849178	G	A	51512	0.030391	-0.10003
15	rs890506	42146712	C	T	49654	0.405939	-0.03897
15	rs7179450	53978353	T	C	50918	0.493342	-0.03612
15	rs4775191	59926946	A	C	50856	0.335988	0.03781
16	rs7191593	71713943	A	G	51540	0.471226	0.04626
17	rs55978930	47299789	G	A	50438	0.308944	0.08295
18	rs8098032	53239302	C	T	51500	0.174893	0.04893
19	rs76076446	3771586	A	G	51626	0.0224596	0.11877
19	rs231222	36274488	C	T	51458	0.387811	-0.03793
21	rs9974393	43434303	A	G	50775	0.432644	-0.05184

21	rs2250773	45087786	T	C	51559	0.406689	-0.05611
22	rs9606708	30646126	T	G	49537	0.29379	0.03885
1	rs2235547	23406485	C	G	51520	0.483589	0.04209
1	rs4660475	39612240	G	A	51466	0.214948	0.05079
1	rs4587551	90289861	A	G	51520	0.0631794	-0.09733
1	rs143278123	151691309	T	C	51002	0.0205972	-0.12392
1	rs6664829	200366695	T	C	46690	0.338488	-0.03822
1	rs6426584	227374949	T	A	51444	0.335647	0.04873
3	rs1264098	53522314	G	T	51128	0.365465	-0.03767
3	rs1463229	150167912	A	G	50937	0.238255	0.09532
3	rs6790999	160386249	G	T	51520	0.0283773	0.10614
4	rs111394384	71528566	T	C	51494	0.0420826	0.09646
5	rs17421627	87847586	G	T	51520	0.0733307	0.06937
5	rs4701143	179045142	A	G	51418	0.220127	-0.04529
6	rs2227956	31778272	G	A	51520	0.189363	0.05003
7	rs2356065	12276885	C	T	51485	0.415169	-0.08827
7	rs10251172	129422494	A	T	50857	0.439605	-0.05194
9	rs139514242	77269429	A	C	51207	0.0200559	0.15966
9	rs2994040	134290898	A	G	51462	0.179054	0.05274
10	rs12253847	22859656	C	T	51520	0.409695	-0.05422
10	rs11595440	32215656	C	G	51485	0.212538	0.0446
10	rs12218853	85981889	A	G	51513	0.231398	0.04205
14	rs1255910	64461068	A	G	49408	0.362239	0.03755
14	rs887595	74666641	A	G	51129	0.178284	-0.04473
14	rs79165758	103773191	G	A	50993	0.397849	0.03627
15	rs1197674	42149952	C	T	50565	0.350667	-0.04417
15	rs556217	53999768	T	C	51312	0.486602	-0.03587
15	rs6151448	59980064	C	T	50904	0.413386	0.03736
16	rs2010428	71772115	T	G	51480	0.468162	0.04045
17	rs411305	43687393	T	C	49739	0.204568	0.04445
17	rs55634267	47283815	T	C	51520	0.313907	0.09549
18	rs34733940	53303684	C	A	51343	0.177054	0.05013
21	rs9974393	43434303	A	G	50679	0.432507	-0.05768
21	rs969060	45080852	T	G	51152	0.419651	-0.05856
22	rs73166584	30649229	T	C	50218	0.171771	-0.04558
1	rs4307512	3762747	C	T	50220	0.254699	-0.06943
1	rs1757046	23511821	C	T	50798	0.345279	0.04371
1	rs590214	39612493	G	A	50857	0.259256	0.04209
1	rs1801466	94476467	A	T	50932	0.0680221	-0.0693
1	rs10737680	196679455	C	A	50932	0.40399	-0.03571
1	rs71633898	200310898	G	C	49915	0.385495	-0.03854
1	rs1513617	227447213	C	T	50834	0.336005	0.04046
2	rs10186426	169012427	G	C	49449	0.263302	-0.04808
2	rs57268345	182412968	A	G	50647	0.108575	-0.06018

3	rs9837054	27711706	G	A	50838	0.150262	0.05465
3	rs115080299	46011023	T	C	50925	0.0118311	-0.16175
3	rs3732530	47618953	C	A	50932	0.332905	0.04515
3	rs348874	100930681	T	A	50525	0.481762	0.03527
3	rs6763422	150170180	T	C	50494	0.239464	0.0909
3	rs11915030	188245035	A	G	50797	0.500955	0.03757
4	rs2970871	23890582	T	C	50547	0.440115	-0.03615
5	rs264521	61632175	T	A	50865	0.415689	-0.03862
5	rs63338061	71486228	T	C	49465	0.361852	0.03577
5	rs17421627	87847586	G	T	50932	0.0732644	0.08532
6	rs11753226	20253392	C	A	50715	0.269486	0.04335
6	rs574389	53377834	T	C	50891	0.477491	-0.03759
6	rs71550518	56708365	C	T	50846	0.182217	-0.04789
6	rs1326170	76760305	T	C	50932	0.319161	-0.05025
7	rs79674914	917184	T	C	50046	0.176767	0.05166
7	rs1990621	12283873	G	C	50706	0.414142	-0.05498
7	rs10251172	129422494	A	T	50280	0.439578	-0.03932
7	rs354088	149338263	C	G	49931	0.0568885	-0.07935
8	rs7816990	10463944	A	C	50932	0.255252	0.06344
8	rs3134331	110701843	G	A	50815	0.221106	0.05742
9	rs2966333	134276649	A	G	50844	0.17417	0.06618
10	rs2799023	32163668	G	A	50831	0.489229	-0.05494
10	rs12265809	62653374	C	T	50323	0.231296	0.05718
11	rs78658393	1791414	A	G	50789	0.124791	0.05691
11	rs7116940	66303895	C	T	50300	0.239682	0.04857
12	rs3138142	56115585	T	C	50582	0.239819	0.05892
13	rs2121036	44555182	C	G	50824	0.407062	0.03782
14	rs6571751	21770730	A	G	50932	0.497781	-0.03865
14	rs1555211	61025791	T	C	50715	0.292744	-0.0502
14	rs7148979	74547318	A	T	50899	0.119541	-0.09809
14	rs17782472	74732757	A	G	50932	0.0486433	-0.08128
14	rs11621828	103778643	A	G	49731	0.486005	-0.05492
15	rs72724186	42145420	T	C	48785	0.109675	-0.05703
15	rs2414183	53100845	C	G	50562	0.132066	-0.05244
15	rs6493642	53990075	C	G	50594	0.489267	-0.04052
16	rs2012649	5064917	T	C	50932	0.258943	-0.04055
16	rs3730393	67226405	A	G	50747	0.0397462	-0.10087
16	rs8053070	71732345	A	G	50765	0.470423	0.06541
17	rs55978930	47299789	G	A	49763	0.308985	0.07587
18	rs525475	595498	T	G	50618	0.162976	-0.05077
18	rs8098032	53239302	C	T	50807	0.174927	0.06825
18	rs4940460	57045134	A	G	49946	0.49996	-0.03803
20	rs449703	25458559	T	C	50786	0.436134	-0.035
21	rs59332489	43378964	G	A	50932	0.180073	0.0446

21	rs11702544	45091861	C	T	50867	0.421953	-0.07125
22	rs11704655	30305898	T	A	50898	0.251513	-0.06134
22	rs2267153	30643609	G	C	48884	0.295649	0.06396
1	rs12727043	3744306	C	T	51189	0.251744	-0.06564
1	rs10218778	21474082	C	T	50796	0.455695	-0.03912
1	rs1757046	23511821	C	T	51062	0.345061	0.03717
1	rs596991	39834692	T	A	50320	0.256538	0.04147
1	rs1801466	94476467	A	T	51198	0.0677468	-0.07775
1	rs36003362	200367088	G	A	51198	0.373579	-0.03863
1	rs1513617	227447213	C	T	51100	0.335939	0.04936
2	rs3769391	169013688	T	G	50726	0.156941	-0.06687
2	rs1441147	182511856	C	T	51120	0.120775	0.06081
3	rs17644807	22019860	A	T	50908	0.158217	0.04676
3	rs9837054	27711706	G	A	51103	0.150314	0.05191
3	rs3772403	47616155	C	T	50891	0.329056	0.04218
3	rs11705918	53789698	G	C	50887	0.211527	-0.04696
3	rs1463229	150167912	A	G	50624	0.238474	0.09927
4	rs7657071	23896474	A	C	50298	0.328363	0.03758
5	rs2112884	61551997	C	A	50550	0.33543	-0.04016
5	rs63338061	71486228	T	C	49731	0.361917	0.04488
5	rs17421627	87847586	G	T	51198	0.0731669	0.07389
5	rs67432087	139689454	C	T	50996	0.205938	-0.04185
6	rs11753226	20253392	C	A	50978	0.270067	0.05209
6	rs600033	53382370	C	G	51125	0.477936	-0.04262
6	rs6928477	76801228	T	C	50869	0.350882	-0.05022
6	rs761779	109011327	A	G	49677	0.153119	-0.05576
6	rs12213228	109369169	G	A	50188	0.109688	-0.06684
7	rs5011434	12268717	T	C	50926	0.413158	-0.05446
7	rs10251172	129422494	A	T	50535	0.439784	-0.04091
7	rs4727085	149230632	A	G	50707	0.0758672	-0.06994
8	rs7816990	10463944	A	C	51198	0.255	0.06986
8	rs3779791	87676710	T	C	51000	0.237706	-0.04215
8	rs3133934	110701012	A	G	51095	0.220834	0.04245
9	rs9802491	134306708	G	A	50793	0.193393	0.0667
10	rs2808093	32141931	T	C	51117	0.489397	-0.05247
10	rs35314358	62647787	A	T	50396	0.218767	0.0536
10	rs372959000	86041869	T	C	49792	0.11069	0.05709
11	rs78658393	1791414	A	G	51055	0.125002	0.05373
11	rs7116940	66303895	C	T	50560	0.239547	0.04379
12	rs3138142	56115585	T	C	50843	0.240141	0.05609
13	rs9513105	28988795	C	T	50955	0.297046	-0.04094
13	rs9567338	44550238	G	A	51124	0.30915	0.04534
13	rs12560780	114324984	T	C	50693	0.482236	-0.0346
14	rs10146342	61007104	G	A	51024	0.292784	-0.04591

14	rs10147158	74465741	A	T	50782	0.460084	0.03694
14	rs887595	74666641	A	G	50806	0.178237	-0.09498
14	rs9630408	103779365	G	A	50038	0.365142	0.05189
15	rs7179450	53978353	T	C	50497	0.493138	-0.03902
16	rs2012649	5064917	T	C	51198	0.259102	-0.04962
16	rs80190634	67660902	A	G	51198	0.0450604	-0.08723
16	rs2010428	71772115	T	G	51157	0.467795	0.05907
17	rs55634267	47283815	T	C	51198	0.314114	0.07594
18	rs485741	594505	C	A	51198	0.164557	-0.05028
18	rs34733940	53303684	C	A	51019	0.177022	0.07134
19	rs58914460	48350847	T	C	48826	0.201573	0.04747
21	rs59332489	43378964	G	A	51198	0.179861	0.05255
21	rs9977076	45085829	G	A	51198	0.420905	-0.06576
22	rs11704655	30305898	T	A	51163	0.251637	-0.05804
22	rs9606708	30646126	T	G	49128	0.293651	0.05946
1	rs12723501	3723990	A	G	49455	0.235285	-0.05289
1	rs651538	21097866	G	A	50398	0.448718	-0.03508
1	rs6426584	227374949	T	A	50580	0.33536	0.03716
2	rs74175027	23998092	A	T	49621	0.419963	-0.04154
2	rs11890527	169031488	C	T	50529	0.174622	-0.04587
3	rs12497826	47669549	A	T	48456	0.327194	0.04211
5	rs63338061	71486228	T	C	49201	0.362005	0.05766
5	rs1438692	148659664	A	G	50653	0.420548	0.03669
6	rs13210876	56604223	T	C	50610	0.17865	-0.04772
6	rs6928477	76801228	T	C	50330	0.350815	-0.03849
7	rs7791653	896293	C	T	50378	0.178868	0.04761
7	rs7806555	50707083	C	G	50161	0.45018	0.03612
7	rs855913	149203662	A	C	50653	0.0648629	-0.06892
8	rs7816990	10463944	A	C	50653	0.254891	0.05614
8	rs3779791	87676710	T	C	50457	0.237608	-0.04082
10	rs2808079	32167385	C	T	50581	0.489878	-0.03579
10	rs12242633	62656355	C	T	50215	0.265498	0.04576
10	rs3781280	104863843	G	T	50493	0.31642	-0.03764
11	rs78658393	1791414	A	G	50511	0.125062	0.05584
11	rs4576	66331000	G	A	50573	0.24433	0.0457
12	rs80247972	96270298	T	C	50581	0.192019	-0.0489
14	rs61991661	61139117	G	A	49988	0.237107	-0.04747
14	rs887595	74666641	A	G	50264	0.178259	-0.11346
14	rs118186707	74686575	A	G	50168	0.0285541	0.13192
14	rs9630408	103779365	G	A	49507	0.365019	0.03818
15	rs3825991	89761664	A	C	48965	0.475166	-0.03467
16	rs2012649	5064917	T	C	50653	0.259126	-0.04479
16	rs2010428	71772115	T	G	50615	0.467559	0.05254
17	rs55978930	47299789	G	A	49497	0.30913	0.04172

22	rs9614123	30371350	G	A	50642	0.251511	-0.04477
22	rs11089441	30654464	T	G	49439	0.294403	0.047
1	rs12747978	3733391	A	G	50194	0.131161	-0.06859
3	rs12497826	47669549	A	T	48168	0.327136	0.03933
4	rs375259	48343550	T	C	49478	0.314281	-0.03753
5	rs116407384	38949632	T	C	50125	0.0260449	0.11478
5	rs3828616	71496419	G	A	50357	0.3292	0.05508
6	rs35440822	56665804	T	C	50071	0.184488	-0.05187
7	rs113319029	917352	A	C	49929	0.175159	0.048
7	rs76929182	98220936	T	C	50357	0.0961832	0.05965
8	rs12544796	10473180	A	G	47436	0.248735	0.04284
8	rs617117	109114464	C	T	48985	0.245953	0.04193
10	rs11008690	32208769	A	G	50292	0.489193	-0.03944
10	rs16915675	62647306	T	G	49566	0.219102	0.04779
10	rs4933314	85980728	T	C	50187	0.469115	-0.03763
11	rs7116940	66303895	C	T	49733	0.23964	0.04483
14	rs1555211	61025791	T	C	50147	0.292809	-0.04445
14	rs887595	74666641	A	G	49971	0.178293	-0.11032
14	rs10145258	74693465	G	A	49309	0.401042	-0.03776
14	rs11623891	103755226	A	C	48926	0.486653	-0.04381
16	rs2012649	5064917	T	C	50357	0.258981	-0.04536
16	rs8063535	71754711	C	T	50279	0.471101	0.04678
1	rs12723501	3723990	A	G	49501	0.235238	-0.04613
2	rs74175027	23998092	A	T	49664	0.419892	-0.0398
3	rs73060324	45785915	G	T	50510	0.0995644	-0.05676
4	rs1021678	186678768	A	G	50320	0.229114	-0.04496
5	rs63338061	71486228	T	C	49247	0.3619	0.05664
5	rs1438692	148659664	A	G	50699	0.420521	0.04372
6	rs3002006	56615714	T	G	50620	0.178605	-0.0489
7	rs7791653	896293	C	T	50423	0.178787	0.04779
7	rs2876869	50706178	T	C	50203	0.450282	0.03554
8	rs7816990	10463944	A	C	50699	0.254857	0.04672
10	rs10829208	27479322	T	C	50507	0.368187	-0.03528
10	rs7089016	62645182	C	T	49768	0.252642	0.04042
10	rs6585847	85981801	G	T	50526	0.471698	-0.03745
11	rs78658393	1791414	A	G	50557	0.125156	0.05385
11	rs10896129	66338300	C	T	50529	0.244295	0.04361
12	rs80247972	96270298	T	C	50627	0.192131	-0.04732
14	rs61991661	61139117	G	A	50032	0.237118	-0.04462
14	rs887595	74666641	A	G	50312	0.178317	-0.10908
14	rs118186707	74686575	A	G	50215	0.0285472	0.12514
16	rs2012649	5064917	T	C	50699	0.25892	-0.04198
16	rs34158217	71834953	C	T	50478	0.486925	0.04761
1	rs2235547	23406485	C	G	51926	0.483688	0.03756

1	rs6427972	202820815	C	G	47892	0.259365	-0.04315
1	rs11590672	212555263	G	C	51630	0.214013	0.04966
1	rs1513617	227447213	C	T	51826	0.336144	0.03718
2	rs11689553	170067947	G	C	51926	0.243086	-0.04489
2	rs17468821	202529514	C	T	51926	0.133247	-0.05645
2	rs3770526	216824967	C	T	51761	0.0929947	-0.05882
3	rs9837054	27711706	G	A	51829	0.150495	0.05996
3	rs73857644	114440375	C	T	51843	0.0702216	-0.07029
3	rs11717195	123082398	C	T	51735	0.247163	0.05459
3	rs729257	156828823	A	G	51439	0.397208	0.03862
5	rs2190989	11584342	C	T	51926	0.452336	0.03554
5	rs17421627	87847586	G	T	51926	0.073027	0.12937
5	rs1438689	148631433	C	T	50839	0.476996	0.04226
5	rs72822609	167736865	G	C	51229	0.100919	0.05808
5	rs166303	173038580	A	C	51832	0.374064	-0.04142
6	rs75757892	7232389	T	C	51393	0.182544	-0.0771
6	rs17507554	11394287	A	G	51926	0.049715	0.09063
6	rs7752728	35477152	C	T	51684	0.210684	-0.06011
6	rs4896369	138814417	T	C	49345	0.458273	-0.03913
6	rs12523793	149973764	A	G	51718	0.332408	0.04615
7	rs187136683	27084648	G	A	51453	0.370921	0.04126
9	rs9657572	21553876	A	T	50461	0.319326	0.04751
9	rs10869425	77213266	C	G	51714	0.471236	0.03574
10	rs17010513	49403140	C	T	51926	0.268112	0.04124
11	rs17705366	31434854	T	A	51184	0.298072	-0.04191
11	rs41313171	129154777	T	C	51752	0.321137	0.03747
12	rs7960781	19089150	C	G	51642	0.3106	-0.04014
12	rs10843854	31055144	T	A	50850	0.235516	0.04354
12	rs3138142	56115585	T	C	51566	0.240236	0.10957
12	rs12308509	96179655	C	T	51759	0.198458	-0.04795
13	rs11069798	110360444	C	T	51809	0.269712	-0.04571
14	rs4981371	21781775	T	C	51813	0.485235	-0.04725
14	rs72683982	21987151	A	G	49495	0.276119	-0.04246
14	rs10135303	74484310	A	G	51799	0.113651	-0.12778
14	rs4903292	75656245	C	T	51335	0.483871	0.03791
15	rs3087328	59981515	G	A	51926	0.416333	0.05193
16	rs9934555	18945075	A	G	48986	0.459305	-0.03618
17	rs1991556	44083402	A	G	51013	0.226001	0.06533
17	rs112364254	79578287	A	G	51816	0.356241	-0.03983
18	rs12458899	57125321	C	T	51876	0.0813286	0.06235
22	rs9606708	30646126	T	G	49823	0.29376	0.0506
1	rs7532266	23551623	A	C	51186	0.305982	0.03846
1	rs11206097	53568101	A	G	51628	0.208501	0.04602
1	rs71633898	200310898	G	C	50964	0.385282	-0.04736

1	rs1470390	212510700	T	C	51926	0.214498	0.05018
1	rs7549464	227214398	G	A	50707	0.464167	0.03833
2	rs11680719	48657843	A	G	51708	0.319747	0.0397
2	rs11689553	170067947	G	C	52005	0.24314	-0.04067
2	rs17384293	202529479	C	T	51972	0.13261	-0.05112
2	rs2177599	227893561	T	G	51925	0.479624	0.03863
3	rs9837054	27711706	G	A	51910	0.150385	0.0689
3	rs7614016	123070426	A	G	51987	0.24761	0.05427
3	rs73865467	129241216	T	C	51950	0.049846	0.07898
3	rs729257	156828823	A	G	51517	0.397238	0.03982
4	rs11734772	163095431	G	T	51394	0.500253	0.03974
5	rs79327504	87847988	A	G	51879	0.0721005	0.13141
5	rs4490572	148612322	A	G	48939	0.460727	0.04739
5	rs6876370	167850039	T	C	51820	0.436559	0.03928
5	rs258874	173045961	G	A	51824	0.376804	-0.04436
6	rs75757892	7232389	T	C	51472	0.18242	-0.0816
6	rs7764472	35479574	G	C	51721	0.146208	-0.06362
6	rs56207206	138820725	T	A	47934	0.456461	-0.04602
6	rs12523793	149973764	A	G	51793	0.332381	0.04923
7	rs73170760	100254991	T	C	52005	0.17432	-0.04883
9	rs9298816	21555283	T	C	50661	0.311384	0.04726
9	rs7862283	77164322	C	T	51667	0.45261	0.03467
10	rs11101258	49410358	T	C	51906	0.267445	0.04537
11	rs11024110	17028390	A	T	51861	0.326247	0.03647
11	rs17705366	31434854	T	A	51262	0.297999	-0.04389
12	rs7960781	19089150	C	G	51722	0.310603	-0.04379
12	rs3138142	56115585	T	C	51646	0.24028	0.11789
12	rs112432639	96167976	G	A	51998	0.183132	-0.04767
13	rs12874510	111878531	G	A	51945	0.39384	-0.03968
14	rs6571751	21770730	A	G	52005	0.497971	-0.04902
14	rs79010241	61627526	G	A	51940	0.0394012	0.08749
14	rs10130013	69563295	G	A	51993	0.327102	0.04478
14	rs887595	74666641	A	G	51609	0.178167	-0.12625
14	rs10145258	74693465	G	A	50933	0.401184	-0.04738
14	rs11622320	103912194	T	A	51870	0.3425	0.03857
15	rs7183831	59984779	A	C	51812	0.415164	0.05351
16	rs4780768	18939081	C	A	50476	0.44534	-0.03479
17	rs17769552	43927290	A	G	51729	0.223366	0.07478
22	rs9606708	30646126	T	G	49898	0.293769	0.05274
1	rs2235547	23406485	C	G	51332	0.483675	0.05563
1	rs6677968	65776702	G	A	51108	0.3917	-0.0396
1	rs112806902	113508391	A	G	51113	0.221137	-0.04504
1	rs17551016	170671962	T	G	51004	0.254715	-0.04091
1	rs71633898	200310898	G	C	50306	0.385709	-0.07346

1	rs12032598	222148552	C	A	48131	0.199684	0.05168
1	rs1513617	227447213	C	T	51233	0.335858	0.05462
2	rs116350483	145338686	T	C	51332	0.0165491	-0.23263
2	rs3820860	169016460	T	C	50804	0.156198	-0.06849
2	rs11689553	170067947	G	C	51332	0.243172	-0.05229
2	rs17384293	202529479	C	T	51301	0.132795	-0.06596
2	rs13030243	216807227	T	C	51132	0.208304	0.04237
2	rs148388367	216850944	A	T	50447	0.033669	-0.13782
3	rs74471895	25478606	G	A	50501	0.212709	-0.05377
3	rs9837054	27711706	G	A	51237	0.15036	0.08597
3	rs4563439	49383779	T	C	51028	0.4256	-0.03602
3	rs73857644	114440375	C	T	51250	0.0702049	-0.07913
3	rs3806709	129109764	A	C	50728	0.146941	0.05095
3	rs12639090	150114504	T	C	51003	0.286513	0.0412
3	rs729257	156828823	A	G	50849	0.397097	0.05519
4	rs139516306	25393598	G	A	50831	0.0309949	0.099
4	rs6838613	48476539	A	G	50065	0.482133	-0.03465
5	rs116513572	87819493	T	C	51321	0.0734982	0.136
5	rs1438689	148631433	C	T	50256	0.477266	0.04178
5	rs258877	173047897	A	G	51132	0.376643	-0.0394
6	rs75757892	7232389	T	C	50804	0.182545	-0.05847
6	rs17507554	11394287	A	G	51332	0.0497838	0.08658
6	rs7752728	35477152	C	T	51090	0.210599	-0.07315
6	rs377749063	79684445	C	G	51298	0.110531	-0.06158
6	rs9398063	106528340	A	G	51104	0.0346842	-0.15861
6	rs4896370	138818837	A	G	49492	0.405156	-0.03964
6	rs9322194	149920249	T	C	51083	0.32781	0.04213
7	rs9639276	867033	T	C	51332	0.173829	0.05545
7	rs187136683	27084648	G	A	50865	0.371179	0.04018
7	rs2237477	50745307	T	C	51058	0.309334	0.03894
7	rs2240093	51102820	A	C	50256	0.279738	-0.04446
7	rs77711029	99507354	T	G	50577	0.420705	0.04205
7	rs12532878	100212254	A	G	50401	0.196256	-0.0489
7	rs13229095	101513672	C	G	50871	0.136787	-0.05199
8	rs113958504	61591436	T	C	50671	0.136705	-0.05207
8	rs71506350	101962901	A	G	51155	0.38614	-0.03599
8	rs13263941	109121945	C	T	50954	0.255603	0.08671
9	rs9657572	21553876	A	T	49887	0.319262	0.06465
9	rs6560395	77164177	C	T	51250	0.454176	0.04499
10	rs4838417	49742205	C	T	50584	0.352987	0.04426
10	rs56238729	70001640	C	T	51318	0.236184	0.04316
10	rs12253241	102733779	C	T	51214	0.206203	-0.04238
11	rs17705366	31434854	T	A	50596	0.298294	-0.06035
11	rs7128814	68953054	G	A	50792	0.422458	-0.03867

11	rs631695	69283303	T	G	50963	0.413182	0.04254
11	rs41313171	129154777	T	C	51160	0.321286	0.04433
12	rs3138142	56115585	T	C	50977	0.239902	0.18274
12	rs17288982	96164706	C	A	50448	0.17414	-0.06544
12	rs920221	109732523	G	T	50928	0.49621	0.03943
13	rs2093472	50145394	A	T	51285	0.286312	0.04343
13	rs7328625	100244963	C	T	47699	0.503983	-0.04021
14	rs8019326	21773023	T	C	51219	0.498428	-0.05038
14	rs79459464	21988688	A	T	50390	0.284828	-0.04042
14	rs12436579	60983087	C	A	51285	0.28951	0.04613
14	rs112109204	74101756	T	C	50203	0.0402366	0.12638
14	rs887595	74666641	A	G	50940	0.178092	-0.24146
14	rs118186707	74686575	A	G	50850	0.0286136	0.23754
14	rs12891131	75240090	A	G	51323	0.462882	-0.06251
15	rs12437603	45498365	T	C	51124	0.298451	0.03901
15	rs8038719	53995635	C	T	51116	0.490355	-0.03781
15	rs28754340	60016301	A	G	47338	0.450748	0.05178
16	rs9934555	18945075	A	G	48422	0.459306	-0.0372
16	rs4889149	80494374	G	A	50554	0.433685	0.05153
16	rs142963458	84561361	T	C	50159	0.0309914	0.11649
17	rs8068292	26321436	T	C	51249	0.279859	0.04415
17	rs1991556	44083402	A	G	50428	0.226333	0.08162
17	rs57043009	59023998	T	C	51056	0.147534	0.07913
17	rs9905786	79602063	G	T	51268	0.355758	-0.06156
18	rs9964861	56937136	A	G	50908	0.304608	0.06123
18	rs11662962	57052843	A	G	51174	0.127653	0.08159
21	rs8132685	34220618	C	T	51164	0.476644	0.04049
22	rs929272	30638292	G	C	49438	0.304846	0.07879
22	rs75159625	46377008	G	T	50910	0.313543	0.03851
1	rs2235547	23406485	C	G	51774	0.483582	0.0544
1	rs71633898	200310898	G	C	50737	0.385301	-0.08091
1	rs12032598	222148552	C	A	48544	0.199726	0.06145
1	rs1513617	227447213	C	T	51676	0.335843	0.05865
2	rs74175027	23998092	A	T	50712	0.419851	-0.03928
2	rs1010659	25595397	C	T	51340	0.311716	-0.03783
2	rs116350483	145338686	T	C	51774	0.016601	-0.27137
2	rs3820860	169016460	T	C	51239	0.156141	-0.06367
2	rs11689553	170067947	G	C	51774	0.243047	-0.04023
2	rs17384293	202529479	C	T	51741	0.132564	-0.05188
2	rs148388367	216850944	A	T	50886	0.0337028	-0.14653
2	rs7599260	218504136	A	G	51544	0.0728019	-0.06565
3	rs74471895	25478606	G	A	50937	0.21291	-0.0596
3	rs9837054	27711706	G	A	51679	0.150254	0.08358
3	rs73857644	114440375	C	T	51692	0.0703591	-0.06736

3	rs73865445	129187713	G	A	51752	0.0499691	0.08471
3	rs58628422	150110582	C	T	51400	0.28644	0.04104
3	rs729257	156828823	A	G	51288	0.397354	0.04819
4	rs1982865	163102673	A	G	51056	0.500676	0.04085
5	rs79327504	87847988	A	G	51648	0.0719583	0.13526
5	rs2400826	148615479	G	A	51487	0.485899	0.04592
5	rs258877	173047897	A	G	51572	0.376765	-0.04567
6	rs75757892	7232389	T	C	51244	0.182431	-0.06145
6	rs17507554	11394287	A	G	51774	0.0497547	0.09063
6	rs7752728	35477152	C	T	51527	0.210734	-0.07112
6	rs9398063	106528340	A	G	51546	0.0347651	-0.14128
6	rs4896370	138818837	A	G	49932	0.404921	-0.0456
6	rs12530220	149934176	C	T	50360	0.338374	0.04272
7	rs75523003	865089	A	G	51585	0.172938	0.04579
7	rs10259123	27083853	A	C	49731	0.342935	0.03787
7	rs2240093	51102820	A	C	50697	0.279445	-0.04574
7	rs2572019	99478804	T	C	51764	0.414796	0.04459
7	rs10953299	100243411	C	T	51119	0.211086	-0.04711
7	rs13229095	101513672	C	G	51313	0.136983	-0.05252
8	rs12547986	61986668	A	G	51244	0.294698	0.04119
8	rs13263941	109121945	C	T	51391	0.255648	0.07794
9	rs9298816	21555283	T	C	50436	0.311414	0.0653
9	rs10869410	77129844	G	A	51335	0.378192	0.04788
10	rs10795055	3581221	A	G	51506	0.396332	-0.03745
10	rs1947075	49741135	C	T	50884	0.35237	0.04787
10	rs1900003	70004551	C	A	51532	0.235155	0.04897
11	rs17705366	31434854	T	A	51033	0.297993	-0.05821
11	rs11606813	69018588	T	C	51365	0.0428794	-0.13457
11	rs2930972	69286205	C	T	51206	0.182664	0.05344
11	rs7122154	128922626	A	C	51111	0.311146	0.04202
12	rs3138142	56115585	T	C	51415	0.240183	0.17716
12	rs35053512	96223776	C	A	51388	0.214593	-0.05394
12	rs920221	109732523	G	T	51371	0.49637	0.03686
13	rs9508029	28991988	T	C	51466	0.297157	-0.03812
13	rs2274278	50126382	G	A	51705	0.15131	0.0484
14	rs8019326	21773023	T	C	51658	0.4982	-0.05144
14	rs28730443	36154749	A	G	51702	0.121301	0.05523
14	rs1254316	60900113	T	C	51716	0.289582	0.04398
14	rs10130013	69563295	G	A	51762	0.32718	0.06401
14	rs113860908	74070803	G	A	51189	0.0419621	0.11804
14	rs887595	74666641	A	G	51378	0.178092	-0.25961
14	rs10145258	74693465	G	A	50710	0.401025	-0.07727
15	rs634990	35006073	C	T	51774	0.488595	-0.0352
15	rs556217	53999768	T	C	51561	0.486909	-0.0378

15	rs7183832	59984780	A	C	49621	0.405171	0.05696
15	rs1372613	101204835	T	C	51467	0.301018	0.03782
16	rs9932878	18932038	G	C	51304	0.449965	-0.03564
16	rs4889149	80494374	G	A	51003	0.433524	0.05107
16	rs35953313	85710476	A	T	50354	0.29515	-0.0446
17	rs62054372	43798775	C	G	51100	0.22137	0.08961
17	rs57043009	59023998	T	C	51494	0.14758	0.07746
17	rs28855509	65998167	C	T	51599	0.218996	0.04568
17	rs138055235	79502316	A	C	48301	0.223422	0.06266
18	rs8098032	53239302	C	T	51647	0.175054	0.04601
18	rs13381549	56899666	A	C	51774	0.0550083	0.10415
18	rs536113	56967796	A	C	50957	0.222962	-0.07058
19	rs76076446	3771586	A	G	51774	0.0223858	-0.1213
21	rs8132685	34220618	C	T	51607	0.476486	0.04261
22	rs2032576	27089655	G	T	51690	0.440037	0.0359
22	rs13054238	30620891	A	G	49690	0.304317	0.08035
22	rs75159625	46377008	G	T	51343	0.313451	0.04236
1	rs6426786	23470460	T	C	50974	0.403068	0.03584
1	rs61817379	170644498	G	A	47182	0.281739	-0.04404
1	rs71633898	200310898	G	C	49960	0.385719	-0.05728
1	rs1513617	227447213	C	T	50876	0.335925	0.04899
2	rs4555304	23910155	G	T	50184	0.489588	-0.04005
2	rs116350483	145338686	T	C	50974	0.0165575	-0.28875
2	rs4668035	169019319	C	T	50588	0.174122	-0.09404
2	rs148388367	216850944	A	T	50097	0.0337445	-0.17972
2	rs62175360	218520035	A	C	50524	0.0689573	-0.11514
2	rs2177599	227893561	T	G	50898	0.479292	0.04165
3	rs372236001	22026823	G	A	50441	0.157897	0.04878
3	rs11129176	25049310	A	G	50905	0.282733	0.05471
3	rs2370990	27690531	T	C	50495	0.242351	0.0509
3	rs4855836	49590256	T	C	50954	0.431222	-0.03633
3	rs35667547	64547477	C	G	50974	0.125142	0.05275
3	rs58628422	150110582	C	T	50607	0.28664	0.04521
3	rs4680287	156329180	G	A	50801	0.273804	-0.04162
3	rs729257	156828823	A	G	50492	0.397281	0.03534
5	rs13162470	38645365	C	T	50231	0.496028	-0.03614
5	rs30373	55745334	G	C	48854	0.365344	0.05718
5	rs63338061	71486228	T	C	49510	0.362159	0.04187
5	rs17421627	87847586	G	T	50974	0.0732825	0.17898
5	rs28712135	88084016	A	T	49048	0.464545	0.04466
5	rs12523362	126095011	A	G	50760	0.199448	-0.07796
5	rs4490572	148612322	A	G	47967	0.460848	0.04061
6	rs9379084	7231843	A	G	48517	0.104829	0.06007
6	rs7752728	35477152	C	T	50735	0.210535	-0.04695

6	rs7742431	79679577	A	G	50826	0.498515	-0.04299
6	rs9398063	106528340	A	G	50746	0.0345939	-0.1826
6	rs12193446	129820038	G	A	50974	0.0954604	0.08039
7	rs9639276	867033	T	C	50974	0.173765	0.06931
7	rs12531825	8005174	A	G	50354	0.120408	0.0713
7	rs2240093	51102820	A	C	49902	0.279798	-0.05746
7	rs4134898	99711614	T	C	50506	0.128965	-0.06186
7	rs34926272	129591807	C	G	49717	0.0237042	-0.15514
8	rs113958504	61591436	T	C	50318	0.13687	-0.07586
8	rs12547986	61986668	A	G	50452	0.294904	0.03865
8	rs375646	109136090	C	T	50689	0.255262	0.13561
9	rs10810150	14452729	T	C	50445	0.167479	0.04834
9	rs9298816	21555283	T	C	49655	0.311248	0.06109
9	rs10869406	77114274	A	G	50520	0.377286	0.04353
10	rs1947075	49741135	C	T	50092	0.351883	0.04417
10	rs1900003	70004551	C	A	50734	0.234882	0.07585
10	rs1696837	123427642	G	A	50974	0.325627	0.04486
11	rs1232180	31640411	A	G	50947	0.30093	-0.05106
11	rs11606813	69018588	T	C	50566	0.0427362	-0.13193
11	rs2930972	69286205	C	T	50416	0.182432	0.05962
11	rs7125694	117670657	C	T	50596	0.49074	0.03646
11	rs12792820	120055403	A	T	50759	0.304104	0.0452
11	rs11218350	121452651	A	T	50681	0.223368	0.04209
12	rs3138142	56115585	T	C	50625	0.239822	0.16527
12	rs17370487	96272463	A	G	50974	0.19253	-0.06986
12	rs920221	109732523	G	T	50574	0.49649	0.03749
13	rs9319429	28973703	T	C	50971	0.296983	-0.04537
13	rs7325207	50112843	C	G	50811	0.159936	0.05685
13	rs9585141	100246997	G	A	49506	0.479306	-0.04373
14	rs112109204	74101756	T	C	49854	0.0401272	0.12663
14	rs887595	74666641	A	G	50583	0.178044	-0.29158
14	rs118186707	74686575	A	G	50496	0.0285864	0.29056
14	rs12891131	75240090	A	G	50965	0.462984	-0.05283
15	rs524952	35005886	A	T	50942	0.489174	-0.03895
15	rs12437603	45498365	T	C	50768	0.298338	0.04374
15	rs2470069	53988114	G	A	50952	0.490118	-0.04801
15	rs28754340	60016301	A	G	47003	0.450546	0.0369
15	rs16956241	72111688	T	G	50825	0.211176	0.05035
15	rs1372613	101204835	T	C	50672	0.301488	0.03885
16	rs5923	67973953	A	G	50974	0.0482697	-0.08298
16	rs62048558	80430160	C	G	48435	0.264901	0.05935
16	rs142963458	84561361	T	C	49812	0.0310467	0.11913
17	rs8068292	26321436	T	C	50892	0.279887	0.03956
17	rs62054372	43798775	C	G	50323	0.221589	0.07519

17	rs9914370	59022395	G	A	50741	0.14711	0.05708
17	rs9905786	79602063	G	T	50910	0.355559	-0.07014
18	rs2271733	56940307	T	G	50974	0.303812	0.06947
18	rs11662962	57052843	A	G	50820	0.127686	0.06909
19	rs76076446	3771586	A	G	50974	0.022433	-0.15078
20	rs575770	62785115	G	A	50553	0.158695	-0.05187
21	rs8132685	34220618	C	T	50807	0.476381	0.0515
21	rs2838337	45072621	A	C	50720	0.420682	-0.04068
22	rs15888	30426335	A	T	49732	0.168956	-0.04745
22	rs929271	30638226	G	T	49126	0.306498	0.06443
22	rs75159625	46377008	G	T	50557	0.313636	0.03819
1	rs2806558	23503456	C	T	51262	0.443847	0.03631
1	rs71633898	200310898	G	C	50343	0.385277	-0.05772
1	rs4950784	202625113	A	G	51344	0.420458	0.03568
1	rs7526703	214126621	A	C	51203	0.33418	-0.03932
1	rs1513617	227447213	C	T	51274	0.335901	0.05244
2	rs7569226	23992582	A	G	49404	0.445096	0.04355
2	rs11892740	145222171	G	A	51372	0.360342	-0.03659
2	rs116350483	145338686	T	C	51372	0.0165654	-0.3109
2	rs3820860	169016460	T	C	50837	0.156077	-0.09076
2	rs17384293	202529479	C	T	51339	0.132755	-0.05075
2	rs148388367	216850944	A	T	50495	0.0336766	-0.18274
2	rs62175360	218520035	A	C	50919	0.0685893	-0.11142
2	rs2177599	227893561	T	G	51292	0.479997	0.03745
3	rs12487626	25043829	G	C	50885	0.28698	0.05557
3	rs2370990	27690531	T	C	50891	0.242115	0.04799
3	rs35667547	64547477	C	G	51372	0.125058	0.05487
3	rs58628422	150110582	C	T	50997	0.286458	0.04625
3	rs12630138	156291765	A	T	49773	0.487051	0.03839
5	rs13162470	38645365	C	T	50618	0.495891	-0.03817
5	rs30373	55745334	G	C	49243	0.365199	0.05423
5	rs63338061	71486228	T	C	49895	0.362191	0.04463
5	rs79327504	87847988	A	G	51247	0.072014	0.17852
5	rs28712135	88084016	A	T	49446	0.464122	0.04105
5	rs12523362	126095011	A	G	51157	0.199416	-0.07179
6	rs7752728	35477152	C	T	51129	0.210771	-0.04957
6	rs13195825	56734711	C	T	51072	0.173344	0.05312
6	rs7742431	79679577	A	G	51221	0.498243	-0.03902
6	rs9398063	106528340	A	G	51147	0.0347723	-0.17258
6	rs12193446	129820038	G	A	51372	0.0956066	0.08054
7	rs75523003	865089	A	G	51186	0.172938	0.06656
7	rs12531825	8005174	A	G	50750	0.120631	0.07086
7	rs2237477	50745307	T	C	51098	0.309405	0.03751
7	rs2285843	51104236	A	G	50720	0.202376	-0.07178

7	rs2572019	99478804	T	C	51362	0.41445	0.04256
7	rs62482495	106965009	G	A	51182	0.237193	0.04112
7	rs34926272	129591807	C	G	50116	0.0236052	-0.14113
8	rs72650452	61617849	T	C	51372	0.137536	-0.06849
8	rs12547986	61986668	A	G	50842	0.29451	0.04097
8	rs13263941	109121945	C	T	50996	0.255481	0.13433
9	rs9298816	21555283	T	C	50039	0.311397	0.06302
9	rs10869406	77114274	A	G	50909	0.377546	0.04578
10	rs1947075	49741135	C	T	50488	0.352618	0.04157
10	rs1900003	70004551	C	A	51134	0.235098	0.07463
10	rs1696837	123427642	G	A	51372	0.325605	0.04458
11	rs10488688	31789114	C	G	50620	0.28951	-0.05383
11	rs11606813	69018588	T	C	50963	0.0429429	-0.15412
11	rs2930972	69286205	C	T	50808	0.182658	0.06362
11	rs7125694	117670657	C	T	50992	0.490646	0.04156
11	rs2076765	120053941	G	A	51122	0.303998	0.04142
11	rs11221653	129177537	C	T	51372	0.391536	0.03987
12	rs3138142	56115585	T	C	51019	0.240126	0.15521
12	rs12827484	96244473	G	A	50964	0.214426	-0.06641
13	rs9508029	28991988	T	C	51064	0.29699	-0.04417
13	rs9535259	50110486	C	G	51320	0.157697	0.05267
13	rs61972411	100602630	G	A	49602	0.450708	-0.03845
14	rs34723417	36292323	C	T	51077	0.155775	0.04779
14	rs151154986	74040136	A	G	51372	0.0316223	0.13705
14	rs887595	74666641	A	G	50979	0.178083	-0.29235
14	rs118186707	74686575	A	G	50885	0.0286332	0.26982
14	rs12891131	75240090	A	G	51363	0.462444	-0.05462
15	rs7161746	34987432	A	T	50096	0.305284	-0.04341
15	rs556217	53999768	T	C	51163	0.486484	-0.04763
15	rs28754340	60016301	A	G	47381	0.450655	0.03995
15	rs4270127	70754781	A	G	51250	0.0894634	-0.0612
15	rs1372613	101204835	T	C	51069	0.301093	0.04715
16	rs80190634	67660902	A	G	51372	0.0449661	-0.08678
16	rs7206532	80490131	T	C	51228	0.48373	-0.05397
16	rs12149202	85700360	A	G	51232	0.276556	-0.04654
17	rs4527069	26324978	G	C	51205	0.279621	0.04068
17	rs62054372	43798775	C	G	50704	0.221274	0.06746
17	rs7502307	65989961	G	C	51035	0.195101	0.04449
17	rs6565592	79505883	T	G	49950	0.314454	0.0665
18	rs34733940	53303684	C	A	51194	0.177101	0.04768
18	rs189921298	56922264	G	A	49508	0.294094	0.07003
18	rs11662962	57052843	A	G	51217	0.127887	0.06173
18	rs62112952	74302423	C	T	51314	0.433176	0.03473
19	rs76076446	3771586	A	G	51372	0.0224636	-0.15405

21	rs8132685	34220618	C	T	51205	0.47635	0.04715
21	rs6518302	45018478	A	G	51312	0.405812	-0.04152
22	rs13054238	30620891	A	G	49308	0.304555	0.06591
1	rs2235547	23406485	C	G	51894	0.483698	0.039
1	rs72683434	113445614	T	C	51789	0.223764	-0.05642
1	rs2019724	196674917	T	C	51872	0.426656	-0.04436
2	rs34326859	24126812	T	G	51212	0.448635	0.03476
2	rs11689553	170067947	G	C	51894	0.243053	-0.04124
3	rs11717195	123082398	C	T	51704	0.247205	0.04836
5	rs17421627	87847586	G	T	51894	0.0730817	0.11821
6	rs75757892	7232389	T	C	51362	0.182547	-0.06321
6	rs7752728	35477152	C	T	51651	0.210683	-0.06224
6	rs12523793	149973764	A	G	51685	0.332495	0.04411
7	rs6463086	42041665	G	A	50626	0.435349	-0.03705
7	rs62482222	100197866	A	G	51000	0.131304	-0.05369
9	rs9657572	21553876	A	T	50431	0.319218	0.03978
10	rs11200922	85961758	G	A	51681	0.459724	0.03819
10	rs60401382	124227624	T	C	51330	0.227596	-0.04271
11	rs17705366	31434854	T	A	51152	0.298072	-0.03761
11	rs10894020	129160328	C	T	51823	0.325531	0.04309
12	rs3138142	56115585	T	C	51535	0.240312	0.12924
12	rs12308509	96179655	C	T	51727	0.198436	-0.04295
14	rs10142653	21782241	G	A	51787	0.485267	-0.04202
14	rs10135303	74484310	A	G	51767	0.113596	-0.09144
15	rs6151442	59980389	T	A	51850	0.415497	0.04261
17	rs62065453	43573419	T	C	51486	0.225382	0.05861
22	rs9606708	30646126	T	G	49793	0.293796	0.05005
1	rs7532266	23551623	A	C	51166	0.305994	0.04448
1	rs76661687	113445001	C	T	51860	0.223776	-0.05212
1	rs1329428	196702810	T	C	51984	0.403807	0.0387
1	rs12139944	202794787	C	T	51984	0.259532	-0.03976
1	rs7549464	227214398	G	A	50686	0.464162	0.03969
2	rs6751857	23973799	C	T	51984	0.445791	0.04215
2	rs13386914	112712498	T	A	51754	0.379546	-0.03605
2	rs11689553	170067947	G	C	51984	0.243075	-0.03964
2	rs2115597	227791218	T	C	50991	0.499686	0.03808
3	rs9837054	27711706	G	A	51889	0.150485	0.0499
3	rs7614016	123070426	A	G	51966	0.247546	0.04672
3	rs9843097	150197676	T	C	51195	0.234789	0.04129
4	rs11734772	163095431	G	T	51373	0.500204	0.0353
5	rs75048985	87847992	A	T	51858	0.0720718	0.12011
5	rs4490572	148612322	A	G	48918	0.4607	0.0422
5	rs72828890	167806836	T	C	49368	0.120118	0.06046
6	rs75757892	7232389	T	C	51453	0.182438	-0.0698

6	rs2064318	35477025	C	G	51984	0.181633	-0.06598
6	rs56207206	138820725	T	A	47912	0.456399	-0.03907
6	rs12523793	149973764	A	G	51771	0.332358	0.04561
7	rs2231172	100252918	G	A	51229	0.207617	-0.04994
10	rs61871747	124213046	T	C	51955	0.214484	-0.05116
11	rs17705366	31434854	T	A	51242	0.297998	-0.04468
11	rs967257	129079118	G	A	51404	0.309344	0.03897
12	rs3138142	56115585	T	C	51625	0.240213	0.1456
12	rs17024669	96164160	A	G	51636	0.180601	-0.04472
14	rs6571751	21770730	A	G	51984	0.497951	-0.04455
14	rs887595	74666641	A	G	51589	0.178119	-0.09495
14	rs973879	75297727	T	C	51918	0.462749	-0.03746
15	rs3936513	60002601	C	T	51493	0.452314	0.04992
17	rs62063300	44048350	C	T	51628	0.223512	0.07367
18	rs11662962	57052843	A	G	51825	0.127902	0.05219
22	rs9606708	30646126	T	G	49880	0.293835	0.0519
22	rs3026641	38364205	A	G	51967	0.31114	-0.03831
1	rs2235547	23406485	C	G	50950	0.483641	0.04205
1	rs61817379	170644498	G	A	47161	0.281705	-0.04477
1	rs71633898	200310898	G	C	49937	0.385686	-0.06489
1	rs919655	214157972	A	G	50403	0.120786	0.05717
1	rs1513617	227447213	C	T	50852	0.335916	0.05375
2	rs6751857	23973799	C	T	50950	0.445486	0.03938
2	rs116350483	145338686	T	C	50950	0.0165653	-0.28642
2	rs4668035	169019319	C	T	50564	0.174116	-0.08835
2	rs11689553	170067947	G	C	50950	0.243003	-0.044
2	rs17384293	202529479	C	T	50919	0.13277	-0.05832
2	rs148388367	216850944	A	T	50075	0.0337294	-0.17634
2	rs7589104	218517174	C	T	50512	0.0689341	-0.10349
2	rs2177599	227893561	T	G	50874	0.479312	0.04179
3	rs4298013	25053643	C	A	50388	0.428495	0.04109
3	rs74471895	25478606	G	A	50125	0.212569	-0.04643
3	rs9837054	27711706	G	A	50857	0.150461	0.07136
3	rs4855836	49590256	T	C	50930	0.431151	-0.03748
3	rs58628422	150110582	C	T	50583	0.286687	0.0453
3	rs4680287	156329180	G	A	50778	0.273859	-0.03992
3	rs729257	156828823	A	G	50468	0.397262	0.04343
5	rs13162470	38645365	C	T	50208	0.495997	-0.03666
5	rs30373	55745334	G	C	48832	0.365385	0.05092
5	rs63338061	71486228	T	C	49486	0.362173	0.03864
5	rs17421627	87847586	G	T	50950	0.0732777	0.17697
5	rs28712135	88084016	A	T	49025	0.464528	0.0422
5	rs62391698	126092596	G	A	50894	0.199984	-0.0629
5	rs4490572	148612322	A	G	47945	0.460809	0.04576

6	rs9379084	7231843	A	G	48497	0.104872	0.06344
6	rs7752728	35477152	C	T	50711	0.210546	-0.05853
6	rs7742431	79679577	A	G	50802	0.498553	-0.04237
6	rs9398063	106528340	A	G	50722	0.0345905	-0.18305
6	rs12193446	129820038	G	A	50950	0.0954858	0.06872
7	rs9639276	867033	T	C	50950	0.173778	0.06819
7	rs12531825	8005174	A	G	50330	0.120465	0.06031
7	rs2240093	51102820	A	C	49876	0.279804	-0.05685
7	rs4134898	99711614	T	C	50482	0.128996	-0.06358
7	rs17407001	101562674	T	C	50215	0.138883	-0.05038
7	rs34926272	129591807	C	G	49692	0.023696	-0.14036
7	rs1225379	155608923	T	C	48304	0.48939	0.03552
8	rs113958504	61591436	T	C	50295	0.136872	-0.07337
8	rs12547986	61986668	A	G	50428	0.294916	0.03879
8	rs375646	109136090	C	T	50664	0.25524	0.12702
9	rs9298816	21555283	T	C	49633	0.311245	0.06555
9	rs10869406	77114274	A	G	50497	0.37726	0.04576
10	rs1947075	49741135	C	T	50068	0.351901	0.04503
10	rs56238729	70001640	C	T	50936	0.23612	0.06918
10	rs4752581	123441729	G	A	49678	0.321782	0.04285
11	rs1232180	31640411	A	G	50923	0.300964	-0.05618
11	rs11606813	69018588	T	C	50542	0.0427268	-0.12816
11	rs2930972	69286205	C	T	50393	0.182466	0.05812
11	rs2076765	120053941	G	A	50706	0.304165	0.04161
11	rs11218350	121452651	A	T	50657	0.223385	0.04188
11	rs11221653	129177537	C	T	50950	0.391698	0.0366
12	rs3138142	56115585	T	C	50601	0.239847	0.17959
12	rs17370487	96272463	A	G	50950	0.192542	-0.0708
12	rs920221	109732523	G	T	50550	0.496469	0.03798
13	rs9513095	28967660	C	T	50913	0.305649	-0.04299
13	rs7325207	50112843	C	G	50787	0.159962	0.05848
13	rs9585141	100246997	G	A	49484	0.479296	-0.045
14	rs11620670	21785133	T	G	50936	0.416964	-0.03897
14	rs112109204	74101756	T	C	49831	0.0401357	0.13505
14	rs887595	74666641	A	G	50559	0.17798	-0.28938
14	rs118186707	74686575	A	G	50473	0.0285994	0.28745
14	rs12891131	75240090	A	G	50941	0.462987	-0.05948
15	rs524952	35005886	A	T	50918	0.48909	-0.03748
15	rs11070445	45513163	A	T	50766	0.300024	0.04462
15	rs7179450	53978353	T	C	50245	0.493144	-0.0469
15	rs7183832	59984780	A	C	48826	0.405276	0.04464
15	rs16956241	72111688	T	G	50801	0.211216	0.04962
16	rs6564760	80485098	C	G	50526	0.243934	0.06192
16	rs142963458	84561361	T	C	49789	0.0310611	0.12174

17	rs8068292	26321436	T	C	50868	0.279891	0.04354
17	rs62054372	43798775	C	G	50298	0.22156	0.08209
17	rs28896238	59021599	C	G	50720	0.147131	0.06868
17	rs9905786	79602063	G	T	50886	0.35552	-0.07042
18	rs34733940	53303684	C	A	50771	0.176951	0.04576
18	rs9964861	56937136	A	G	50528	0.304415	0.07031
18	rs11662962	57052843	A	G	50796	0.127697	0.0765
19	rs76076446	3771586	A	G	50950	0.0224436	-0.14804
20	rs575770	62785115	G	A	50529	0.158741	-0.05051
21	rs8132685	34220618	C	T	50783	0.47639	0.05253
22	rs15888	30426335	A	T	49710	0.16889	-0.04795
22	rs929271	30638226	G	T	49103	0.306468	0.07205
22	rs75159625	46377008	G	T	50533	0.313597	0.04029
1	rs16824124	3741985	C	T	51264	0.131437	-0.05652
1	rs2235547	23406485	C	G	51319	0.48371	0.04958
1	rs7519368	113150644	A	T	51013	0.274126	-0.03857
1	rs72683434	113445614	T	C	51215	0.22388	-0.08292
1	rs12409048	170699149	A	G	51319	0.25513	-0.04255
1	rs1329428	196702810	T	C	51319	0.403934	0.05354
1	rs71633898	200310898	G	C	50294	0.385662	-0.05427
1	rs12032598	222148552	C	A	48120	0.199647	0.05452
1	rs1513617	227447213	C	T	51220	0.335914	0.04771
2	rs6751857	23973799	C	T	51319	0.445644	0.05536
2	rs11892740	145222171	G	A	51319	0.360383	-0.03647
2	rs116350483	145338686	T	C	51319	0.0165631	-0.21626
2	rs10432464	169024826	A	G	50925	0.174158	-0.06928
2	rs11689553	170067947	G	C	51319	0.243195	-0.05184
2	rs7594781	234227113	C	G	51310	0.0497466	-0.08752
3	rs9837054	27711706	G	A	51224	0.150291	0.08061
3	rs12639090	150114504	T	C	50990	0.286448	0.03912
3	rs729257	156828823	A	G	50836	0.39711	0.04463
4	rs6446731	3284751	A	G	51118	0.282777	-0.04024
5	rs2914245	38655281	C	T	51319	0.494437	0.03583
5	rs10940160	67563227	A	T	51215	0.471112	0.03628
5	rs17421627	87847586	G	T	51319	0.0731211	0.1484
5	rs28712135	88084016	A	T	49387	0.464242	0.04093
6	rs7743323	7195684	T	C	51103	0.189539	-0.05746
6	rs7752728	35477152	C	T	51077	0.210633	-0.08606
6	rs9398063	106528340	A	G	51090	0.0346839	-0.12664
6	rs10872647	150079823	C	T	51197	0.337119	0.04745
7	rs10237857	867879	A	G	47247	0.185356	0.05063
7	rs2240093	51102820	A	C	50243	0.279721	-0.03966
7	rs62482222	100197866	A	G	50433	0.130996	-0.06458
7	rs13229095	101513672	C	G	50859	0.13681	-0.06286

8	rs113958504	61591436	T	C	50659	0.136639	-0.0509
8	rs12547986	61986668	A	G	50792	0.295066	0.0406
8	rs13263941	109121945	C	T	50941	0.255629	0.08918
9	rs9657572	21553876	A	T	49874	0.319345	0.05425
9	rs6560395	77164177	C	T	51237	0.454086	0.04374
10	rs4838417	49742205	C	T	50571	0.353009	0.0442
10	rs56238729	70001640	C	T	51305	0.236137	0.04893
10	rs10788333	85960149	A	C	51250	0.458732	0.05224
10	rs2672587	124235355	G	C	51319	0.236082	-0.05081
11	rs61879160	622044	T	C	51005	0.147074	0.05808
11	rs17705366	31434854	T	A	50583	0.298163	-0.05675
11	rs78977588	68968149	A	C	50517	0.11989	0.06528
11	rs2930972	69286205	C	T	50760	0.182329	0.05324
11	rs2155394	117378116	A	G	51129	0.451368	0.03428
11	rs7127011	129099686	T	C	51064	0.318306	0.04902
12	rs3138142	56115585	T	C	50964	0.239905	0.23318
12	rs17288982	96164706	C	A	50434	0.174	-0.0592
12	rs920221	109732523	G	T	50916	0.496347	0.03509
13	rs9508029	28991988	T	C	51010	0.297177	-0.04341
13	rs7325207	50112843	C	G	51154	0.160232	0.05401
13	rs11839787	114318239	G	A	50188	0.440185	-0.04313
14	rs10142653	21782241	G	A	51213	0.485385	-0.04892
14	rs112109204	74101756	T	C	50190	0.0402172	0.10686
14	rs887595	74666641	A	G	50927	0.178059	-0.20593
14	rs118186707	74686575	A	G	50837	0.0286111	0.21018
14	rs12891131	75240090	A	G	51310	0.462902	-0.05794
15	rs6416452	53994493	A	G	51118	0.490356	-0.04743
15	rs28754340	60016301	A	G	47325	0.450766	0.04334
15	rs8023681	89148093	G	A	50985	0.408542	0.03505
15	rs7175019	89732471	C	T	50888	0.502054	0.04277
16	rs28738814	80489238	A	T	51167	0.499287	-0.04035
17	rs1991556	44083402	A	G	50414	0.226346	0.06968
17	rs9303435	59013670	C	T	51001	0.147232	0.07001
17	rs138055235	79502316	A	C	47899	0.223355	0.04382
18	rs55847427	6716098	G	T	51281	0.475946	0.03527
18	rs28774981	56936000	A	G	49386	0.277326	0.06202
18	rs11664409	57026673	G	T	50463	0.0898381	0.07664
21	rs17696057	34164832	A	G	51166	0.0700563	-0.06954
22	rs9614155	30581998	C	G	51103	0.158533	-0.04973
22	rs929271	30638226	G	T	49457	0.306559	0.08055
22	rs9330814	46364191	T	C	50987	0.312256	0.03916
1	rs12728401	3731965	A	G	51604	0.130145	-0.053
1	rs2235547	23406485	C	G	51772	0.483572	0.05591
1	rs72683434	113445614	T	C	51668	0.223833	-0.07839

1	rs1329428	196702810	T	C	51772	0.403954	0.05161
1	rs71633898	200310898	G	C	50736	0.385279	-0.0624
1	rs12032598	222148552	C	A	48543	0.199761	0.06234
1	rs1513617	227447213	C	T	51674	0.335836	0.05801
2	rs6751857	23973799	C	T	51772	0.445946	0.0577
2	rs116350483	145338686	T	C	51772	0.016592	-0.24765
2	rs10497336	169013165	T	C	51210	0.160125	-0.06955
2	rs11689553	170067947	G	C	51772	0.242988	-0.04323
2	rs7583380	234223886	A	G	51686	0.048785	-0.09321
3	rs74471895	25478606	G	A	50936	0.212796	-0.04609
3	rs9837054	27711706	G	A	51677	0.15027	0.07626
3	rs6784048	150117934	G	A	51451	0.286243	0.03819
3	rs729257	156828823	A	G	51286	0.397389	0.04054
3	rs56351888	183904226	T	C	51767	0.167887	-0.04494
4	rs11100415	163070497	G	A	51355	0.458037	-0.03754
5	rs2914245	38655281	C	T	51772	0.494485	0.03477
5	rs79327504	87847988	A	G	51646	0.0719514	0.14193
5	rs28712135	88084016	A	T	49831	0.464089	0.03926
5	rs2400826	148615479	G	A	51484	0.485957	0.03738
5	rs394248	173042832	C	A	51637	0.374877	-0.03809
6	rs75757892	7232389	T	C	51241	0.182413	-0.06051
6	rs7752728	35477152	C	T	51525	0.210655	-0.0831
6	rs9398059	106514039	A	G	51458	0.0341541	-0.12043
6	rs4896370	138818837	A	G	49932	0.404991	-0.03878
6	rs12530220	149934176	C	T	50363	0.338433	0.04551
7	rs10259123	27083853	A	C	49729	0.342959	0.03725
7	rs62482222	100197866	A	G	50876	0.1313	-0.07418
7	rs13229095	101513672	C	G	51311	0.136978	-0.06344
8	rs12547986	61986668	A	G	51242	0.294729	0.04814
8	rs13263941	109121945	C	T	51389	0.255599	0.08304
9	rs1377193	21566440	G	A	51595	0.314255	0.05256
9	rs11144015	77200750	A	G	51293	0.466955	0.04199
10	rs1947075	49741135	C	T	50882	0.352472	0.0447
10	rs56238729	70001640	C	T	51758	0.236398	0.05419
10	rs11200922	85961758	G	A	51562	0.459398	0.04499
10	rs10490924	124214448	T	G	51772	0.214353	-0.04966
11	rs28499086	635240	T	A	49826	0.250592	0.04841
11	rs17705366	31434854	T	A	51031	0.297986	-0.0585
11	rs67808136	69055051	A	G	51660	0.053194	-0.11355
11	rs2930972	69286205	C	T	51203	0.182694	0.04997
11	rs967257	129079118	G	A	51195	0.309317	0.05217
12	rs3138142	56115585	T	C	51413	0.240173	0.23473
12	rs17288982	96164706	C	A	50883	0.174007	-0.05744
12	rs4766590	109744235	T	C	51772	0.479313	0.03528

13	rs2296284	28963676	A	G	51412	0.294523	-0.04756
13	rs6561539	50112129	C	G	51696	0.157275	0.04943
13	rs9577895	114498034	A	G	50009	0.497131	0.04046
14	rs8019326	21773023	T	C	51657	0.49819	-0.0494
14	rs10134646	36124335	G	A	51718	0.121534	0.0646
14	rs112109204	74101756	T	C	50629	0.0401549	0.09773
14	rs887595	74666641	A	G	51376	0.17805	-0.2208
14	rs118186707	74686575	A	G	51282	0.0286845	0.21007
14	rs973879	75297727	T	C	51706	0.462577	-0.05976
15	rs634990	35006073	C	T	51772	0.488585	-0.03854
15	rs556217	53999768	T	C	51559	0.486918	-0.04982
15	rs3936513	60002601	C	T	51287	0.452317	0.04713
15	rs12898755	63574641	A	G	51687	0.206986	0.04263
15	rs4932481	89723999	C	G	49870	0.484039	0.03738
16	rs28559271	80489223	G	A	51604	0.484633	-0.047
16	rs1974868	85710983	T	C	50901	0.294199	-0.04191
17	rs62054372	43798775	C	G	51099	0.221443	0.08023
17	rs9303435	59013670	C	T	51449	0.147223	0.06675
17	rs28855509	65998167	C	T	51598	0.219059	0.04204
17	rs138055235	79502316	A	C	48298	0.223446	0.04249
18	rs12605508	56917562	C	T	51369	0.29729	0.06038
18	rs11662962	57052843	A	G	51613	0.127952	0.07422
20	rs1327226	10920872	C	T	51346	0.078789	-0.07001
21	rs12627346	34249135	G	A	51285	0.432592	-0.03565
22	rs4443100	23372864	G	C	50912	0.306971	-0.0385
22	rs13054238	30620891	A	G	49687	0.304265	0.08458
22	rs9330813	46364161	A	G	51772	0.312795	0.04463
1	rs11102510	113144012	T	C	50867	0.255234	-0.04109
1	rs72683434	113445614	T	C	50809	0.223641	-0.06607
1	rs61817379	170644498	G	A	47121	0.281711	-0.04179
1	rs4915434	200365963	T	C	50596	0.216045	0.04379
1	rs10800867	202792179	A	G	50658	0.354623	-0.05053
1	rs919655	214157972	A	G	50365	0.120719	0.05948
1	rs16846872	227236016	C	T	50779	0.144046	0.05898
2	rs7560892	23928637	A	G	50882	0.453422	-0.06355
2	rs2959258	45285120	G	C	50646	0.474845	0.03912
2	rs11892740	145222171	G	A	50913	0.360154	-0.04204
2	rs116350483	145338686	T	C	50913	0.0165675	-0.29733
2	rs10221681	169035382	T	C	50821	0.174121	-0.11294
2	rs11689553	170067947	G	C	50913	0.243003	-0.04248
2	rs7589104	218517174	C	T	50474	0.0689761	-0.10833
2	rs10203008	227956622	G	A	50376	0.486492	0.03568
2	rs55895356	234224681	T	C	50871	0.0500875	-0.13378
3	rs11129176	25049310	A	G	50844	0.282747	0.05156

3	rs35667547	64547477	C	G	50913	0.125145	0.05442
3	rs58628422	150110582	C	T	50546	0.28664	0.0464
3	rs4680287	156329180	G	A	50740	0.273837	-0.04493
4	rs77766742	72543037	G	T	50553	0.097917	-0.06184
5	rs13162470	38645365	C	T	50171	0.496093	-0.04092
5	rs30373	55745334	G	C	48798	0.365374	0.06343
5	rs63338061	71486228	T	C	49450	0.362083	0.0426
5	rs71580755	87002295	G	A	49791	0.0469964	-0.08621
5	rs114622514	87827065	G	A	50902	0.0736022	0.18135
5	rs28712135	88084016	A	T	48990	0.464503	0.05505
5	rs12523362	126095011	A	G	50699	0.199442	-0.07386
5	rs3797315	148620224	G	T	50777	0.486441	0.04134
6	rs2326838	6901663	A	G	50856	0.363231	-0.04803
6	rs7752728	35477152	C	T	50674	0.210621	-0.06116
6	rs519458	84271264	A	T	50676	0.278347	-0.03862
6	rs9398063	106528340	A	G	50685	0.0346059	-0.17306
6	rs12193446	129820038	G	A	50913	0.095457	0.06315
6	rs9366187	170552900	C	G	50197	0.218051	-0.04178
7	rs9639276	867033	T	C	50913	0.173767	0.06014
7	rs12531825	8005174	A	G	50295	0.120449	0.07436
7	rs2240093	51102820	A	C	49842	0.279744	-0.05635
7	rs35111986	100000274	A	C	50891	0.19359	0.07015
7	rs13229095	101513672	C	G	50457	0.136978	-0.063
7	rs34926272	129591807	C	G	49656	0.0237031	-0.15231
8	rs113958504	61591436	T	C	50260	0.136858	-0.08189
8	rs12547986	61986668	A	G	50392	0.294878	0.04523
8	rs375646	109136090	C	T	50627	0.2552	0.14385
9	rs10810150	14452729	T	C	50384	0.167444	0.05818
9	rs9657572	21553876	A	T	49477	0.319502	0.0537
9	rs6560395	77164177	C	T	50833	0.454282	0.03872
10	rs1947075	49741135	C	T	50032	0.351905	0.047
10	rs1900003	70004551	C	A	50673	0.23479	0.0834
10	rs4562752	85978809	G	T	50746	0.47252	0.05797
10	rs1696837	123427642	G	A	50913	0.325673	0.04442
11	rs61879160	622044	T	C	50599	0.14692	0.05481
11	rs10488688	31789114	C	G	50166	0.289599	-0.04192
11	rs67808136	69055051	A	G	50801	0.0530108	-0.11082
11	rs2930972	69286205	C	T	50357	0.182408	0.06493
11	rs149950515	83873446	G	A	49876	0.0113381	0.16385
11	rs10892132	117375951	A	G	50714	0.45098	0.04228
11	rs3819132	117671489	A	G	50309	0.443569	0.04597
11	rs10790981	128821108	T	C	50733	0.330593	0.0421
12	rs1391789	24183284	A	G	50685	0.234488	-0.0406
12	rs3138142	56115585	T	C	50565	0.239909	0.18643

12	rs17368197	96196672	A	C	50856	0.192377	-0.0764
12	rs59966711	109729141	T	G	48679	0.323507	-0.03966
12	rs7296418	123457619	C	T	50913	0.285703	0.03949
13	rs9319429	28973703	T	C	50910	0.296955	-0.06402
13	rs7325207	50112843	C	G	50750	0.15999	0.0545
13	rs9585141	100246997	G	A	49447	0.47924	-0.04153
13	rs9669831	114313349	G	A	49893	0.484226	-0.05532
14	rs28730443	36154749	A	G	50846	0.121543	0.05616
14	rs112109204	74101756	T	C	49794	0.0401655	0.11783
14	rs887595	74666641	A	G	50522	0.178021	-0.28531
14	rs118186707	74686575	A	G	50436	0.0285808	0.27978
14	rs12891131	75240090	A	G	50904	0.463019	-0.05381
15	rs7161746	34987432	A	T	49654	0.305756	-0.04367
15	rs556217	53999768	T	C	50698	0.486804	-0.05397
15	rs16956273	72117102	A	G	50913	0.235323	0.04926
15	rs2070780	89760997	T	C	49317	0.469209	0.04234
15	rs1372613	101204835	T	C	50612	0.30145	0.03869
16	rs79437914	67578143	A	C	50887	0.0447364	-0.12167
16	rs4888087	80490858	G	T	50913	0.500452	-0.04153
16	rs3815797	85720955	A	G	50259	0.288595	-0.04897
17	rs12449582	6337965	T	G	50824	0.190432	-0.06024
17	rs35524223	44192590	A	T	50072	0.219584	0.06795
17	rs9303434	59013669	G	A	50386	0.144832	0.05658
17	rs6565592	79505883	T	G	49505	0.314493	0.05313
18	rs17696543	56971398	T	C	49595	0.179645	-0.07531
19	rs76076446	3771586	A	G	50913	0.0224599	-0.12557
21	rs8132685	34220618	C	T	50746	0.476363	0.04908
21	rs2838338	45075582	T	C	50654	0.420855	-0.05935
22	rs5996460	23393090	A	G	50328	0.291051	-0.03803
22	rs11913168	30606564	A	G	50913	0.109226	-0.09248
22	rs9330814	46364191	T	C	50586	0.312379	0.0394
1	rs7519368	113150644	A	T	51017	0.274203	-0.04083
1	rs112806902	113508391	A	G	51107	0.221026	-0.06508
1	rs12409048	170699149	A	G	51322	0.255193	-0.03976
1	rs12134598	196681001	G	T	51292	0.173936	0.04959
1	rs71633898	200310898	G	C	50293	0.385372	-0.05291
1	rs1513617	227447213	C	T	51224	0.335878	0.05225
2	rs4665251	24190263	C	A	51110	0.449276	0.06727
2	rs2959258	45285120	G	C	51056	0.474861	0.03417
2	rs11892740	145222171	G	A	51322	0.360265	-0.04138
2	rs116350483	145338686	T	C	51322	0.0165524	-0.31136
2	rs12613885	169032900	T	G	51220	0.157282	-0.10963
2	rs7589104	218517174	C	T	50880	0.0685731	-0.10553
2	rs10203008	227956622	G	A	50792	0.486583	0.03748

2	rs55895356	234224681	T	C	51277	0.0500224	-0.12897
3	rs11129176	25049310	A	G	51253	0.282715	0.04937
3	rs9845475	32842101	G	T	51322	0.136287	0.05311
3	rs35667547	64547477	C	G	51322	0.125093	0.05253
3	rs58628422	150110582	C	T	50947	0.286386	0.04934
3	rs4680287	156329180	G	A	51146	0.273746	-0.04125
5	rs327264	38653601	T	G	50917	0.494324	0.04228
5	rs30373	55745334	G	C	49196	0.365162	0.05866
5	rs63338061	71486228	T	C	49845	0.362233	0.0475
5	rs79327504	87847988	A	G	51198	0.0720145	0.18174
5	rs28712135	88084016	A	T	49398	0.464199	0.04908
5	rs12523362	126095011	A	G	51107	0.199425	-0.07035
5	rs2400826	148615479	G	A	51035	0.486	0.03858
6	rs2326838	6901663	A	G	51267	0.363431	-0.04034
6	rs7752728	35477152	C	T	51079	0.210742	-0.06309
6	rs74526772	106515218	A	T	51064	0.0345743	-0.17622
6	rs12193446	129820038	G	A	51322	0.0956023	0.06303
7	rs10950673	874639	G	A	51264	0.17763	0.05854
7	rs12531825	8005174	A	G	50701	0.120668	0.07454
7	rs2876869	50706178	T	C	50824	0.450152	0.03608
7	rs62448278	51098849	A	G	50214	0.281246	-0.05662
7	rs35111986	100000274	A	C	51300	0.193635	0.0665
7	rs13229095	101513672	C	G	50865	0.13698	-0.06067
7	rs34926272	129591807	C	G	50066	0.0236188	-0.15817
8	rs7816990	10463944	A	C	51322	0.254803	0.04099
8	rs113958504	61591436	T	C	50668	0.13699	-0.07669
8	rs12547986	61986668	A	G	50792	0.294466	0.05071
8	rs13263941	109121945	C	T	50946	0.255467	0.14118
9	rs1377193	21566440	G	A	51149	0.31419	0.05615
9	rs10781236	77181631	G	T	51273	0.455259	0.04216
10	rs7067692	45430699	A	G	51089	0.219392	-0.04345
10	rs1947075	49741135	C	T	50438	0.352571	0.04455
10	rs1900003	70004551	C	A	51084	0.235142	0.08123
10	rs11200922	85961758	G	A	51112	0.459315	0.05729
10	rs1696837	123427642	G	A	51322	0.325679	0.04043
11	rs61879160	622044	T	C	51006	0.146973	0.0548
11	rs10488688	31789114	C	G	50570	0.289351	-0.04138
11	rs116233906	68968271	A	C	50900	0.040609	-0.13159
11	rs2930972	69286205	C	T	50759	0.182628	0.06294
11	rs2155394	117378116	A	G	51132	0.451899	0.04509
11	rs7125694	117670657	C	T	50942	0.490636	0.05345
11	rs11221551	128920332	T	C	51097	0.316878	0.04259
12	rs4280086	24130571	A	G	51004	0.377706	-0.03589
12	rs3138142	56115585	T	C	50969	0.240156	0.18085

12	rs17370487	96272463	A	G	51322	0.192335	-0.07478
12	rs59966711	109729141	T	G	49095	0.323638	-0.03837
13	rs9319429	28973703	T	C	51318	0.296689	-0.06237
13	rs9535259	50110486	C	G	51270	0.157714	0.05146
13	rs9585141	100246997	G	A	49847	0.479337	-0.03798
13	rs7399672	114467309	C	A	50731	0.457925	-0.0593
14	rs28730443	36154749	A	G	51250	0.121415	0.06489
14	rs17825846	65598752	G	C	51247	0.349962	-0.03722
14	rs112109204	74101756	T	C	50192	0.0400462	0.1247
14	rs887595	74666641	A	G	50930	0.178019	-0.2889
14	rs118186707	74686575	A	G	50835	0.0286318	0.26783
14	rs973879	75297727	T	C	51257	0.462259	-0.05478
15	rs7161746	34987432	A	T	50046	0.305219	-0.04371
15	rs556217	53999768	T	C	51113	0.486461	-0.05442
15	rs16956273	72117102	A	G	51322	0.235299	0.04487
15	rs4932480	89723858	T	C	49990	0.486517	0.03903
15	rs1372613	101204835	T	C	51019	0.301133	0.0438
16	rs140837060	67662815	A	T	51182	0.0443222	-0.11833
16	rs9921725	80489718	T	A	51165	0.483885	-0.04998
16	rs9930110	85720780	C	T	50552	0.291897	-0.05362
17	rs12449582	6337965	T	G	51229	0.190615	-0.06854
17	rs8068292	26321436	T	C	51238	0.279773	0.04206
17	rs62054372	43798775	C	G	50655	0.221291	0.06746
17	rs8077430	79511135	A	G	51041	0.227209	0.05896
18	rs17696543	56971398	T	C	50001	0.179746	-0.08253
19	rs76076446	3771586	A	G	51322	0.0224563	-0.12797
21	rs8132685	34220618	C	T	51155	0.476386	0.04331
21	rs2838338	45075582	T	C	51064	0.42106	-0.05769
22	rs4443100	23372864	G	C	50462	0.306983	-0.03901
22	rs13054238	30620891	A	G	49259	0.304513	0.06412
1	rs72637739	9165685	A	G	51583	0.215565	-0.04253
1	rs6668533	113064468	A	G	51514	0.259861	-0.06045
1	rs72683432	113442300	C	T	51170	0.22266	-0.08947
1	rs1329428	196702810	T	C	51583	0.404154	0.09661
1	rs173273	212446689	G	T	51242	0.406327	-0.03598
2	rs12470949	23934816	T	C	51567	0.287335	-0.04454
2	rs2871922	112719431	A	G	51560	0.374922	-0.04555
2	rs3769876	165569373	T	C	50378	0.414844	0.03708
2	rs288337	183545110	G	A	48967	0.349133	0.04071
2	rs1864251	216829638	G	A	51327	0.0930797	0.1081
2	rs7564805	234228946	G	A	51583	0.0496578	-0.12732
3	rs114582462	48517349	G	A	51248	0.0205471	-0.12304
3	rs6771073	58458043	A	G	51334	0.0885476	-0.06959
3	rs62266133	93561499	T	C	51557	0.260547	0.04396

3	rs511575	100942033	A	T	51459	0.209604	-0.0466
3	rs3806709	129109764	A	C	50977	0.14687	-0.05412
3	rs149973742	196385773	G	A	49642	0.0267616	0.10787
5	rs11133859	16783993	G	A	51223	0.260205	-0.04482
5	rs186024631	17154247	T	C	51340	0.0245812	-0.13784
5	rs115237855	17186336	A	G	51583	0.0243879	-0.12642
6	rs2326838	6901663	A	G	51525	0.363076	-0.04307
6	rs556679	31894355	T	C	51447	0.118539	0.08753
6	rs1581836	76796339	C	T	51318	0.311382	0.04176
6	rs9320282	109619377	A	G	51492	0.481366	-0.03792
8	rs57819090	10469340	A	G	51056	0.126195	0.09499
10	rs11101268	49445540	A	G	51293	0.0351997	-0.10043
10	rs4562752	85978809	G	T	51411	0.472078	0.04689
10	rs10490924	124214448	T	G	51583	0.214266	-0.11759
11	rs11230527	60720893	T	G	50192	0.181752	-0.04865
11	rs61629638	76939409	T	C	51255	0.21196	-0.05615
11	rs12289724	87915956	G	A	51414	0.0958202	0.05921
11	rs1847142	89021574	A	G	51277	0.327944	-0.05716
12	rs7969761	355842	C	T	51208	0.447557	-0.04815
12	rs3138142	56115585	T	C	51225	0.240303	0.10994
13	rs9556964	99159306	T	C	51172	0.323829	0.03819
14	rs7155448	60937851	T	C	51357	0.302666	-0.05488
15	rs1800407	28230318	T	C	51583	0.0832542	0.08265
15	rs12898755	63574641	A	G	51498	0.207474	0.04724
15	rs3825991	89761664	A	C	49857	0.475249	0.05321
16	rs142963458	84561361	T	C	50403	0.0309109	-0.19143
17	rs9889642	79591762	C	T	51564	0.451303	-0.04999
19	rs11085197	6713175	C	G	51531	0.21066	-0.04719
19	rs56248314	17566634	A	T	50968	0.16066	0.05645
20	rs6077977	10930708	G	A	51551	0.483987	0.06299
20	rs11697947	32613494	A	T	50121	0.355739	0.04446
1	rs72637739	9165685	A	G	51322	0.215512	-0.04412
1	rs12125361	113144779	T	C	51272	0.256885	-0.05781
1	rs72683432	113442300	C	T	50913	0.222674	-0.07918
1	rs1329428	196702810	T	C	51322	0.403648	0.10272
2	rs4848901	112710828	T	C	51242	0.448577	-0.04694
2	rs34360304	203294455	A	G	51204	0.035798	0.09753
2	rs148388367	216850944	A	T	50445	0.0336802	0.17747
2	rs7583380	234223886	A	G	51237	0.0488221	-0.12718
3	rs11706087	48539536	C	T	51138	0.024033	-0.11493
3	rs6441594	100960895	A	C	51263	0.209225	-0.04362
5	rs250347	16789844	C	T	50798	0.449821	-0.03423
5	rs115836805	17129578	T	C	51186	0.0258958	-0.13048
5	rs115237855	17186336	A	G	51322	0.0242391	-0.14874

6	rs2326838	6901663	A	G	51266	0.363165	-0.04534
6	rs556679	31894355	T	C	51189	0.118717	0.09953
6	rs418532	42663072	G	A	50904	0.426568	0.03456
6	rs1952134	76786554	T	A	51083	0.311522	0.04263
6	rs1546723	109625879	A	G	51212	0.443968	0.03662
8	rs57819090	10469340	A	G	50795	0.126006	0.08661
10	rs11101268	49445540	A	G	51037	0.0352097	-0.09566
10	rs4562752	85978809	G	T	51149	0.47216	0.0424
10	rs11200633	124211596	T	C	51049	0.213716	-0.12112
11	rs11235950	73633622	A	G	51282	0.130757	-0.05595
11	rs12577658	76929337	A	G	50832	0.189654	-0.06015
11	rs3900053	89021065	C	T	51012	0.328089	-0.0564
12	rs7969761	355842	C	T	50951	0.447145	-0.0495
12	rs3138142	56115585	T	C	50964	0.240591	0.11513
13	rs9556964	99159306	T	C	50912	0.323627	0.0368
14	rs1254260	60835737	A	G	51243	0.288059	-0.05399
15	rs1800407	28230318	T	C	51322	0.0833366	0.08037
15	rs12898755	63574641	A	G	51237	0.207038	0.05854
15	rs3825991	89761664	A	C	49611	0.475691	0.0488
16	rs142963458	84561361	T	C	50146	0.0309995	-0.19533
17	rs12948698	10089663	A	G	48953	0.214481	-0.04302
17	rs111812808	79585834	A	G	51276	0.342139	-0.06163
19	rs11569415	6716279	A	G	49768	0.216163	-0.04304
19	rs62125245	17570005	G	A	50836	0.16208	0.07509
20	rs6077979	10933773	A	G	50422	0.471263	0.06818
20	rs11697947	32613494	A	T	49877	0.356226	0.0414
1	rs72637739	9165685	A	G	51216	0.215665	-0.0489
1	rs6668533	113064468	A	G	51147	0.259722	-0.07199
1	rs72683442	113468825	C	T	51192	0.222447	-0.0909
1	rs1329428	196702810	T	C	51216	0.40418	0.08464
2	rs12470949	23934816	T	C	51200	0.287412	-0.05398
2	rs61457616	165817419	A	G	50563	0.304215	0.04213
2	rs1864251	216829638	G	A	50962	0.0928437	0.12038
2	rs7564805	234228946	G	A	51216	0.0496427	-0.16081
3	rs10865725	14328227	C	T	50551	0.424898	0.0364
3	rs6771073	58458043	A	G	50969	0.0886421	-0.07843
3	rs348882	100971181	C	G	51072	0.210272	-0.06259
3	rs3806709	129109764	A	C	50611	0.146925	-0.07173
3	rs9844184	150132039	G	A	51216	0.243137	-0.04641
3	rs115483925	183925356	A	G	50851	0.182317	-0.05142
5	rs11133859	16783993	G	A	50857	0.260387	-0.03967
5	rs186024631	17154247	T	C	50973	0.0244541	-0.13634
5	rs115237855	17186336	A	G	51216	0.0243088	-0.13069
6	rs2326838	6901663	A	G	51158	0.363198	-0.06062

6	rs556679	31894355	T	C	51082	0.118652	0.07333
6	rs9381202	42580545	G	T	51196	0.31397	-0.04263
6	rs1581836	76796339	C	T	50951	0.311397	0.05942
7	rs111963714	99948655	G	T	50422	0.210285	0.05661
8	rs57819090	10469340	A	G	50690	0.126208	0.09073
10	rs10793567	45415207	T	C	50329	0.285025	-0.046
10	rs35314358	62647787	A	T	50408	0.218834	0.05368
10	rs4562752	85978809	G	T	51049	0.472193	0.06537
10	rs200227426	124213671	A	C	50086	0.203191	-0.10283
11	rs11230527	60720893	T	G	49830	0.181597	-0.0547
11	rs11237155	76968775	G	C	51216	0.20516	-0.06431
11	rs1847142	89021574	A	G	50912	0.327516	-0.07241
12	rs11613331	351467	G	A	51036	0.448125	-0.0573
12	rs3138142	56115585	T	C	50861	0.240007	0.14302
13	rs9556964	99159306	T	C	50803	0.324193	0.04581
13	rs9669831	114313349	G	A	50198	0.484422	-0.04386
14	rs7155448	60937851	T	C	50996	0.302661	-0.04698
15	rs1800407	28230318	T	C	51216	0.0832552	0.10827
15	rs12898755	63574641	A	G	51131	0.207252	0.04265
15	rs3825991	89761664	A	C	49510	0.475278	0.07964
15	rs11433	101716583	A	G	51025	0.437736	-0.03809
16	rs142963458	84561361	T	C	50044	0.0309628	-0.20472
17	rs12948698	10089663	A	G	48862	0.214584	-0.04425
17	rs4794029	47280301	T	C	50682	0.317371	-0.04891
17	rs112364254	79578287	A	G	51107	0.356057	0.07716
19	rs11085197	6713175	C	G	51165	0.210662	-0.04767
20	rs6077977	10930708	G	A	51185	0.483481	0.06857
20	rs11697947	32613494	A	T	49761	0.356122	0.0407
1	rs78870840	9169131	A	G	51023	0.214599	-0.04277
1	rs12125361	113144779	T	C	51348	0.257147	-0.07282
1	rs72683432	113442300	C	T	50987	0.222812	-0.08967
1	rs1329428	196702810	T	C	51398	0.403741	0.08431
2	rs4233703	24066193	G	A	51268	0.445736	0.03952
2	rs61457616	165817419	A	G	50746	0.304113	0.04063
2	rs148388367	216850944	A	T	50521	0.0337187	0.16996
2	rs7583380	234223886	A	G	51314	0.048895	-0.15791
3	rs6771073	58458043	A	G	51153	0.088597	-0.07627
3	rs521661	100946742	T	G	51321	0.208842	-0.05282
3	rs68155216	129217968	C	T	51021	0.112904	-0.07852
3	rs1463229	150167912	A	G	50820	0.238489	-0.05232
5	rs186024631	17154247	T	C	51153	0.0245049	-0.13042
5	rs115237855	17186336	A	G	51398	0.0242811	-0.15971
6	rs2326838	6901663	A	G	51340	0.363177	-0.05762
6	rs556679	31894355	T	C	51265	0.118716	0.08415

6	rs418532	42663072	G	A	50981	0.426551	0.04592
6	rs1952134	76786554	T	A	51158	0.31174	0.05257
7	rs111963714	99948655	G	T	50612	0.210276	0.04533
8	rs57819090	10469340	A	G	50871	0.125985	0.08086
10	rs10793567	45415207	T	C	50516	0.284989	-0.04421
10	rs11101268	49445540	A	G	51112	0.0353048	-0.09915
10	rs16915682	62650959	G	A	50601	0.246833	0.04003
10	rs4562752	85978809	G	T	51226	0.472026	0.0586
10	rs10490924	124214448	T	G	51398	0.214357	-0.10032
11	rs11230527	60720893	T	G	50012	0.181796	-0.06037
11	rs11235841	73367122	C	T	51178	0.128952	-0.05871
11	rs61629638	76939409	T	C	51068	0.211923	-0.06054
11	rs3900053	89021065	C	T	51088	0.328208	-0.07676
12	rs7969761	355842	C	T	51023	0.447494	-0.06215
12	rs3138142	56115585	T	C	51040	0.240439	0.149
13	rs9556964	99159306	T	C	50988	0.323792	0.04129
13	rs9669831	114313349	G	A	50377	0.484566	-0.0453
14	rs1254260	60835737	A	G	51318	0.287959	-0.04966
15	rs1800407	28230318	T	C	51398	0.0832912	0.12364
15	rs12898755	63574641	A	G	51313	0.206955	0.05766
15	rs3825991	89761664	A	C	49688	0.475266	0.07417
15	rs7171515	101769618	T	C	51097	0.366626	0.0419
16	rs142963458	84561361	T	C	50220	0.0310335	-0.19528
17	rs12948698	10089663	A	G	49027	0.214494	-0.04565
17	rs7222840	47280915	T	C	50883	0.317582	-0.04559
17	rs11150803	79621160	A	C	50745	0.475466	0.08848
19	rs7246201	8235311	T	C	51371	0.452025	0.0397
20	rs1232603	10612963	T	C	50719	0.324188	-0.03684
20	rs6077977	10930708	G	A	51368	0.483326	0.07266
20	rs6142055	32539806	T	C	51126	0.413205	0.04305
1	rs72637739	9165685	A	G	50508	0.215738	-0.04912
1	rs11587687	110147801	A	G	50412	0.442682	0.04999
1	rs7519368	113150644	A	T	50207	0.273657	-0.0693
1	rs2149036	113479886	A	T	50486	0.222309	-0.07536
1	rs6677089	196684313	C	A	50485	0.404199	0.06686
2	rs7581989	23933132	T	C	50468	0.453793	-0.04779
2	rs72886128	165822558	G	A	49649	0.302614	0.04199
2	rs6728947	169059511	C	G	49726	0.389937	0.05389
2	rs6715936	198148570	T	C	50126	0.244554	0.04198
2	rs1864251	216829638	G	A	50257	0.0928726	0.09194
2	rs7564805	234228946	G	A	50508	0.0496555	-0.20778
3	rs7632128	14330340	A	G	50477	0.365572	0.03717
3	rs348882	100971181	C	G	50365	0.210483	-0.07064
3	rs3806709	129109764	A	C	49915	0.14687	-0.09738

3	rs2903834	150167144	G	A	50330	0.460074	-0.03594
3	rs2231224	183901281	A	G	50508	0.167944	-0.05171
5	rs113100410	17168087	G	A	50508	0.0713748	-0.07908
5	rs201456755	88043466	C	T	50226	0.284633	-0.04248
6	rs2326838	6901663	A	G	50451	0.363016	-0.05921
6	rs488755	31904306	A	G	50376	0.0745196	0.06616
6	rs2273001	35478612	G	A	49938	0.299391	-0.03861
6	rs376389	42662574	A	C	50327	0.286129	-0.07666
6	rs111791023	56714017	C	A	50319	0.180876	-0.05646
6	rs1581836	76796339	C	T	50244	0.311649	0.06065
7	rs1964242	99976703	A	G	50420	0.186484	0.06655
8	rs57819090	10469340	A	G	49995	0.126143	0.09722
10	rs10793570	45442793	G	A	49144	0.214116	-0.04287
10	rs7073076	85962650	G	T	50290	0.460201	0.10314
10	rs60401382	124227624	T	C	49954	0.227319	-0.05546
11	rs11230527	60720893	T	G	49138	0.18154	-0.05209
11	rs12574286	76937602	C	G	50156	0.211361	-0.05926
11	rs10765199	88971638	A	T	49680	0.306421	-0.06197
12	rs11613331	351467	G	A	50330	0.448242	-0.05639
12	rs3138142	56115585	T	C	50161	0.239878	0.09112
13	rs17086617	28962686	C	T	49809	0.299876	-0.04385
13	rs9556964	99159306	T	C	50100	0.324371	0.03861
13	rs9669831	114313349	G	A	49499	0.484171	-0.06507
14	rs28711886	61072658	G	A	48486	0.318185	-0.04414
15	rs1800407	28230318	T	C	50508	0.0831056	0.11502
15	rs111466394	52742888	G	A	50062	0.165854	0.06571
15	rs3825991	89761664	A	C	48824	0.475125	0.09042
15	rs11433	101716583	A	G	50318	0.437885	-0.04137
16	rs142963458	84561361	T	C	49356	0.0310499	-0.18714
17	rs12449582	6337965	T	G	50420	0.190579	-0.05319
17	rs4794029	47280301	T	C	49979	0.317373	-0.04877
17	rs111812808	79585834	A	G	50463	0.342746	-0.07772
19	rs1379868	5827097	G	A	50206	0.177539	-0.04502
20	rs7828	10619014	C	A	50053	0.328372	-0.05289
20	rs6077977	10930708	G	A	50480	0.48333	0.07101
21	rs2251253	45092257	T	A	50449	0.422209	-0.04756
1	rs6669802	110163076	A	G	49491	0.474197	-0.04436
1	rs6668533	113064468	A	G	50613	0.259884	-0.0686
1	rs72683432	113442300	C	T	50272	0.222838	-0.07023
1	rs1329428	196702810	T	C	50679	0.404102	0.06162
1	rs6663745	220097906	T	G	50526	0.21291	-0.04365
2	rs4665244	24102691	A	G	50671	0.383987	-0.04499
2	rs72886118	165819021	T	A	50028	0.30307	0.0422
2	rs10930308	169017436	A	G	50450	0.388989	0.05024

2	rs1864251	216829638	G	A	50430	0.0928812	0.07644
2	rs7583380	234223886	A	G	50596	0.0488181	-0.20411
3	rs4684200	14338285	C	T	50106	0.390472	0.03781
3	rs13094898	58290143	A	G	50627	0.0821597	-0.0673
3	rs2660806	100926254	A	C	50670	0.209453	-0.06506
3	rs68155216	129217968	C	T	50305	0.112762	-0.10154
3	rs6778948	150134304	G	A	50667	0.243107	-0.04299
5	rs186024631	17154247	T	C	50439	0.0245643	-0.13701
5	rs115237855	17186336	A	G	50679	0.0241816	-0.13732
5	rs304152	88124123	G	T	50496	0.304064	-0.04088
6	rs2326838	6901663	A	G	50623	0.363195	-0.05989
6	rs556679	31894355	T	C	50548	0.118699	0.06366
6	rs649472	42673015	C	T	50376	0.288153	-0.0751
6	rs13194625	56685244	G	A	50611	0.177481	-0.06532
6	rs1952134	76786554	T	A	50441	0.31176	0.05229
6	rs11153159	109376647	G	C	50332	0.113993	-0.06088
7	rs111963714	99948655	G	T	49910	0.210268	0.06126
8	rs57819090	10469340	A	G	50155	0.1259	0.07494
8	rs13261873	109121977	T	C	49067	0.243667	0.04179
10	rs7067692	45430699	A	G	50446	0.219413	-0.05253
10	rs16915682	62650959	G	A	49889	0.246587	0.04144
10	rs4562752	85978809	G	T	50508	0.471945	0.09947
10	rs200227426	124213671	A	C	49556	0.202851	-0.06453
11	rs4073591	674094	G	T	50486	0.448728	0.03715
11	rs11230527	60720893	T	G	49310	0.181829	-0.05299
11	rs61629638	76939409	T	C	50353	0.211874	-0.06905
11	rs1847142	89021574	A	G	50378	0.328	-0.07069
12	rs7969761	355842	C	T	50307	0.447443	-0.059
12	rs3138142	56115585	T	C	50327	0.240269	0.09342
13	rs17086617	28962686	C	T	49979	0.299916	-0.04824
13	rs9669831	114313349	G	A	49676	0.484077	-0.06386
14	rs1254260	60835737	A	G	50600	0.288123	-0.04702
15	rs1800407	28230318	T	C	50679	0.0831607	0.11718
15	rs117089270	52804249	A	G	50357	0.167405	0.06982
15	rs12898755	63574641	A	G	50595	0.207145	0.04952
15	rs3825991	89761664	A	C	48992	0.475016	0.08663
15	rs7171515	101769618	T	C	50381	0.366914	0.03809
16	rs142963458	84561361	T	C	49514	0.0310417	-0.17953
17	rs12449580	6337247	G	C	50679	0.190098	-0.0496
17	rs35638197	47284735	C	T	50679	0.0586436	0.09436
17	rs11150803	79621160	A	C	50034	0.475607	0.08229
20	rs1232603	10612963	T	C	50008	0.324218	-0.05485
20	rs6077977	10930708	G	A	50649	0.483771	0.07022
21	rs2251253	45092257	T	A	50619	0.42248	-0.04691

1	rs2235547	23406485	C	G	51352	0.483642	0.04246
1	rs71633898	200310898	G	C	50323	0.385341	-0.06825
1	rs919655	214157972	A	G	50801	0.121021	0.05621
1	rs11118920	222155060	T	A	50921	0.35941	0.03559
1	rs1513617	227447213	C	T	51254	0.335876	0.05577
2	rs7569226	23992582	A	G	49384	0.445094	0.04368
2	rs116350483	145338686	T	C	51352	0.0165622	-0.31098
2	rs3820860	169016460	T	C	50817	0.156089	-0.08612
2	rs17581439	172737267	C	T	50592	0.264044	0.03917
2	rs17384293	202529479	C	T	51319	0.132729	-0.05633
2	rs148388367	216850944	A	T	50475	0.0336899	-0.18092
2	rs7589104	218517174	C	T	50910	0.0685818	-0.10258
2	rs10203008	227956622	G	A	50821	0.48658	0.03762
3	rs4298013	25053643	C	A	50784	0.428806	0.04154
3	rs74471895	25478606	G	A	50520	0.212441	-0.04814
3	rs9837054	27711706	G	A	51257	0.150379	0.06866
3	rs35667547	64547477	C	G	51352	0.125039	0.05349
3	rs111351719	129095665	A	G	51284	0.0541202	0.0763
3	rs58628422	150110582	C	T	50978	0.286427	0.04779
3	rs7638745	156278397	C	T	49866	0.338507	-0.0367
3	rs729257	156828823	A	G	50870	0.397297	0.04022
5	rs13162470	38645365	C	T	50599	0.495909	-0.03736
5	rs30373	55745334	G	C	49224	0.365167	0.04948
5	rs63338061	71486228	T	C	49875	0.362195	0.0405
5	rs79327504	87847988	A	G	51227	0.0720226	0.17558
5	rs28712135	88084016	A	T	49426	0.464159	0.03859
5	rs12523362	126095011	A	G	51137	0.199454	-0.06014
5	rs2400826	148615479	G	A	51066	0.486008	0.03982
5	rs394248	173042832	C	A	51220	0.374815	-0.03941
6	rs17507554	11394287	A	G	51352	0.0498715	0.08628
6	rs7752728	35477152	C	T	51109	0.210755	-0.05952
6	rs13195825	56734711	C	T	51052	0.173333	0.04799
6	rs7742431	79679577	A	G	51201	0.498311	-0.03714
6	rs9398063	106528340	A	G	51127	0.0347761	-0.17244
6	rs12193446	129820038	G	A	51352	0.0956048	0.07256
7	rs75523003	865089	A	G	51166	0.172947	0.06167
7	rs929511	8003017	T	C	50012	0.122291	0.06393
7	rs2237477	50745307	T	C	51078	0.30937	0.03802
7	rs2240093	51102820	A	C	50282	0.279484	-0.06034
7	rs2572019	99478804	T	C	51342	0.414456	0.04525
7	rs10953299	100243411	C	T	50703	0.211122	-0.04359
7	rs13229095	101513672	C	G	50895	0.136988	-0.04991
7	rs34926272	129591807	C	G	50096	0.0236147	-0.12865
8	rs72650452	61617849	T	C	51352	0.137551	-0.06565

8	rs12547986	61986668	A	G	50822	0.294459	0.04458
8	rs13263941	109121945	C	T	50976	0.255512	0.12218
9	rs9298816	21555283	T	C	50020	0.311445	0.06608
9	rs10869406	77114274	A	G	50889	0.377528	0.04886
10	rs1947075	49741135	C	T	50469	0.352543	0.04542
10	rs1900003	70004551	C	A	51114	0.235112	0.06895
10	rs1693686	123426526	C	T	51250	0.321415	0.04342
11	rs10488688	31789114	C	G	50600	0.289437	-0.05588
11	rs11606813	69018588	T	C	50943	0.04295	-0.15571
11	rs2930972	69286205	C	T	50788	0.182661	0.06455
11	rs7932097	117373125	T	C	51207	0.46025	0.03542
11	rs7125694	117670657	C	T	50972	0.490642	0.03633
11	rs11221653	129177537	C	T	51352	0.391601	0.04006
12	rs3138142	56115585	T	C	50999	0.240162	0.16971
12	rs12827484	96244473	G	A	50944	0.214471	-0.06568
12	rs920221	109732523	G	T	50955	0.496163	0.03543
13	rs9513095	28967660	C	T	51314	0.305433	-0.04211
13	rs6561542	50127520	T	C	51286	0.151347	0.05579
13	rs61972411	100602630	G	A	49583	0.450689	-0.03654
14	rs8019326	21773023	T	C	51238	0.498361	-0.03658
14	rs28730443	36154749	A	G	51280	0.121392	0.05696
14	rs112109204	74101756	T	C	50222	0.040082	0.12861
14	rs887595	74666641	A	G	50959	0.178025	-0.29304
14	rs118186707	74686575	A	G	50865	0.0286445	0.26809
14	rs12891131	75240090	A	G	51343	0.462449	-0.05935
15	rs524952	35005886	A	T	51321	0.488903	-0.03852
15	rs556217	53999768	T	C	51143	0.486538	-0.04578
15	rs7183832	59984780	A	C	49230	0.405261	0.04699
15	rs1372613	101204835	T	C	51049	0.301073	0.04666
16	rs7206532	80490131	T	C	51208	0.483811	-0.05602
16	rs12149202	85700360	A	G	51212	0.276576	-0.04689
17	rs4527069	26324978	G	C	51185	0.279652	0.04188
17	rs62054372	43798775	C	G	50684	0.221283	0.07858
17	rs9303434	59013669	G	A	50815	0.145134	0.05576
17	rs28855509	65998167	C	T	51182	0.219071	0.04463
17	rs6565592	79505883	T	G	49931	0.314464	0.06558
18	rs34733940	53303684	C	A	51174	0.177063	0.05105
18	rs189921298	56922264	G	A	49488	0.294102	0.07177
18	rs11662962	57052843	A	G	51197	0.127879	0.06997
19	rs76076446	3771586	A	G	51352	0.0224626	-0.15478
19	rs2913983	8474897	T	G	50098	0.312148	-0.03733
20	rs575770	62785115	G	A	50928	0.158655	-0.04706
21	rs8132685	34220618	C	T	51185	0.47639	0.04836
22	rs13054238	30620891	A	G	49288	0.304547	0.07296

22	rs75159625	46377008	G	T	50924	0.313654	0.03978
1	rs12724921	3051350	A	T	45914	0.0644901	0.07405
1	rs1192415	92077097	G	A	45914	0.187819	0.14399
1	rs12137699	116209762	T	C	45808	0.236367	0.06189
1	rs11808696	227563680	T	C	45792	0.0772187	-0.12423
2	rs78118320	19342779	G	C	45848	0.0125196	-0.18291
3	rs1072823	25416508	G	C	45034	0.442743	0.0545
3	rs17398137	100625703	A	G	45914	0.182069	-0.06386
4	rs74764079	81952637	A	T	45692	0.0262956	-0.12335
4	rs1566795	112375172	C	T	45413	0.356528	-0.04307
5	rs72759609	31952051	C	T	45653	0.10087	-0.07791
5	rs77443179	87826858	G	A	45626	0.0855762	0.07722
6	rs6927837	39485604	G	A	45751	0.158434	0.05261
6	rs7744813	73643289	C	A	44355	0.410878	0.04262
6	rs11758026	122432029	C	T	45416	0.360853	-0.04227
6	rs1313696	127052113	G	A	45392	0.503404	-0.04179
8	rs4876271	1901930	A	G	45914	0.126617	0.05427
8	rs9297976	143752235	C	T	44861	0.432079	0.03816
9	rs7866671	89275519	A	G	45890	0.336947	0.0423
9	rs35704524	134560240	G	A	45852	0.268669	-0.04408
10	rs10764494	25058144	C	A	45741	0.317265	-0.04258
10	rs1900003	70004551	C	A	45705	0.234307	-0.15366
10	rs7893954	104318966	G	A	45914	0.289672	-0.04377
11	rs11031445	31690406	C	A	45914	0.230823	0.04607
12	rs55710412	31067490	T	C	43563	0.144607	-0.06917
12	rs904091	83950668	A	G	45725	0.443488	-0.04864
13	rs9534439	47192049	T	C	45736	0.190419	0.05049
14	rs12147505	60660734	C	T	45813	0.0120162	-0.27683
14	rs12435232	85726683	G	A	45732	0.442896	0.04476
16	rs8050675	51466372	A	G	45527	0.303622	0.05326
17	rs11079418	59239754	T	G	45860	0.186546	0.06341
17	rs9905786	79602063	G	T	45856	0.356856	-0.04986
22	rs1972202	29100564	A	G	45887	0.358871	-0.0574
22	rs6000765	37920993	T	C	45773	0.24616	0.04404
1	rs10127988	110144625	T	C	50634	0.442845	-0.06413
1	rs1329427	196704559	T	C	50586	0.403946	0.11268
1	rs896319	205205651	G	T	50480	0.0893918	-0.07447
2	rs148117129	44236821	A	T	50411	0.0106723	0.16607
2	rs148388367	216850944	A	T	49768	0.0337466	-0.1798
2	rs6719241	234215226	T	G	50547	0.0485093	0.11337
3	rs12493349	14331064	C	T	50509	0.326377	-0.04106
3	rs78248959	58455068	A	G	50634	0.088488	0.06216
3	rs3806709	129109764	A	C	50038	0.146878	0.14244
6	rs6914444	1983440	C	T	50401	0.134035	-0.07122

6	rs394754	42659979	G	T	50479	0.286208	0.0714
6	rs28385609	123122464	T	C	50634	0.140587	0.04983
7	rs112123532	76964256	T	C	50494	0.200143	-0.04619
8	rs57819090	10469340	A	G	50113	0.126205	-0.139
10	rs491995	18244008	A	G	49379	0.374106	0.03746
10	rs28376229	125654919	T	C	49845	0.152162	0.04794
11	rs11230527	60720893	T	G	49264	0.181309	0.07702
11	rs61629638	76939409	T	C	50308	0.211815	0.0488
11	rs1954772	89033269	T	C	50320	0.329203	0.13491
12	rs3138142	56115585	T	C	50288	0.240008	0.04358
14	rs144262012	60881561	A	T	50293	0.284135	0.03885
15	rs17566952	28196885	G	C	50178	0.062089	-0.09753
15	rs1800407	28230318	T	C	50634	0.0832642	-0.20164
15	rs147620932	28473685	A	G	50588	0.0175536	-0.1508
16	rs142963458	84561361	T	C	49477	0.0311054	0.12448
17	rs35638197	47284735	C	T	50634	0.0586464	-0.08114
17	rs112364254	79578287	A	G	50526	0.355718	-0.09233
19	rs117043618	48327482	T	C	50634	0.133043	-0.05405
22	rs5754223	33104058	C	T	50512	0.0490181	-0.07876
1	rs10127988	110144625	T	C	50976	0.442748	-0.0635
1	rs10922109	196704632	A	C	50929	0.403905	0.11143
1	rs6593925	205100663	G	C	50938	0.093712	-0.08275
2	rs1864251	216829638	G	A	50722	0.092869	-0.0967
2	rs6719241	234215226	T	G	50888	0.0484495	0.11202
3	rs9310440	14313725	T	C	50284	0.317546	-0.03828
3	rs1604006	25064354	C	T	50976	0.474606	-0.03483
3	rs139976016	129109698	G	A	49715	0.137383	0.12861
6	rs72841994	1972832	T	A	50786	0.132999	-0.07512
6	rs376389	42662574	A	C	50794	0.286372	0.06852
6	rs943080	43826627	C	T	50757	0.502344	0.04197
7	rs59086319	76947884	A	T	50780	0.199183	-0.04704
7	rs1618426	134946000	T	G	50198	0.497032	-0.0355
8	rs57819090	10469340	A	G	50452	0.125971	-0.13845
8	rs140397694	10480582	C	G	50976	0.0161743	-0.13622
10	rs491995	18244008	A	G	49707	0.374233	0.03636
11	rs11230527	60720893	T	G	49598	0.181661	0.07501
11	rs61629638	76939409	T	C	50647	0.211947	0.05246
11	rs141432217	88849315	T	C	49432	0.064968	-0.07123
11	rs1954772	89033269	T	C	50661	0.329553	0.1347
12	rs567959241	51357930	C	A	50730	0.191652	0.04782
12	rs3138142	56115585	T	C	50626	0.240163	0.0546
15	rs17566952	28196885	G	C	50513	0.0621721	-0.09305
15	rs1800407	28230318	T	C	50976	0.0832745	-0.21027
15	rs147620932	28473685	A	G	50928	0.0175346	-0.13122

15	rs2289702	79237293	T	C	50671	0.103304	-0.06283
16	rs8050818	84583233	A	G	50601	0.21884	0.04898
17	rs35638197	47284735	C	T	50976	0.0585668	-0.08587
17	rs112364254	79578287	A	G	50868	0.356383	-0.09625
1	rs4839276	113524639	G	T	50520	0.228484	-0.06299
1	rs12409048	170699149	A	G	50858	0.254827	-0.04435
1	rs2154328	198943015	T	G	50030	0.358675	0.03746
1	rs7553418	202801296	G	A	50591	0.354193	-0.04781
1	rs80256218	214110713	T	C	50831	0.0764888	-0.06778
2	rs74175027	23998092	A	T	49836	0.419927	-0.03475
2	rs3769393	169013590	T	C	50372	0.160506	-0.06427
2	rs6433705	178851890	C	T	50680	0.38677	0.0376
2	rs7589104	218517174	C	T	50420	0.0688516	-0.06916
2	rs10203008	227956622	G	A	50322	0.486457	0.05065
2	rs7597716	228179901	C	T	50554	0.274152	0.03976
3	rs62251889	32808114	A	G	50303	0.10897	0.05605
3	rs17279437	45814094	A	G	50858	0.106571	-0.10697
4	rs6818978	26083058	T	C	50066	0.239544	0.04123
4	rs10023264	184927422	G	T	48853	0.334289	0.04082
5	rs10474678	11534538	T	G	49695	0.464715	0.0359
5	rs30373	55745334	G	C	48748	0.365379	0.0362
5	rs71580755	87002295	G	A	49737	0.0470173	-0.08919
5	rs17421627	87847586	G	T	50858	0.0732333	0.23475
5	rs28712135	88084016	A	T	48934	0.464411	0.05508
5	rs2963490	148594477	G	A	49560	0.491223	0.05069
6	rs9379066	6901856	C	T	50768	0.364698	-0.0401
6	rs7752728	35477152	C	T	50618	0.210686	-0.06058
6	rs1575676	108861264	T	C	50705	0.368129	-0.03986
6	rs12193446	129820038	G	A	50858	0.095452	0.06949
6	rs10872647	150079823	C	T	50737	0.33718	0.04505
6	rs705773	164606534	T	C	49152	0.473836	0.03585
7	rs113670532	8100884	T	C	50321	0.133006	0.05251
7	rs187136683	27084648	G	A	50401	0.371193	0.0371
7	rs6968945	46640900	C	T	50185	0.406675	-0.05932
7	rs2237468	50734362	T	C	50139	0.221265	0.05283
7	rs2079308	51017122	G	T	50608	0.29116	-0.05186
7	rs34989573	100097895	T	C	50086	0.194316	0.04492
7	rs1302921	155609058	A	T	47722	0.46136	-0.0466
8	rs13263941	109121945	C	T	50486	0.255526	0.05049
9	rs1983810	21552654	C	T	48961	0.308102	0.0514
9	rs6478995	102792856	A	C	50837	0.427376	-0.0374
10	rs4838417	49742205	C	T	50120	0.352993	0.05266
10	rs12217769	85981842	G	A	50858	0.235194	0.04304
11	rs7127818	17004090	C	G	50820	0.289109	0.04356

11	rs10488688	31789114	C	G	50112	0.289492	-0.04795
11	rs11606813	69018588	T	C	50452	0.0428229	-0.09262
11	rs1532547	88475938	A	G	49764	0.353991	-0.03873
11	rs4936637	121447188	C	T	50515	0.223171	0.04586
11	rs10893946	128862342	A	C	50343	0.39359	0.04292
12	rs3138142	56115585	T	C	50510	0.240032	0.14733
12	rs7298676	96177759	C	T	50772	0.188884	-0.06296
13	rs9508027	28986781	A	C	50634	0.296856	-0.04404
14	rs71418269	23445076	A	C	49233	0.394177	-0.03893
14	rs28711886	61072658	G	A	48825	0.318402	-0.04748
14	rs887595	74666641	A	G	50468	0.177994	-0.06423
14	rs12891131	75240090	A	G	50849	0.462998	-0.04611
15	rs11854924	41288239	C	G	50692	0.302326	-0.03946
15	rs556217	53999768	T	C	50644	0.4868	-0.04783
15	rs11854063	59952905	G	A	50647	0.336812	0.03834
15	rs1470108	89153744	A	C	50175	0.333762	0.04856
16	rs4635359	80537760	C	A	50514	0.261878	0.05322
17	rs2732631	44289232	T	G	50198	0.228645	0.06496
17	rs9303434	59013669	G	A	50332	0.144858	0.06669
17	rs28855509	65998167	C	T	50688	0.219056	0.04132
17	rs9905786	79602063	G	T	50794	0.355475	-0.09742
18	rs11662407	56921989	A	G	50809	0.303135	0.05169
18	rs11662962	57052843	A	G	50704	0.127741	0.0563
19	rs4803297	40310084	T	C	50280	0.431156	-0.0376
22	rs11913168	30606564	A	G	50858	0.109314	-0.0608
22	rs75159625	46377008	G	T	50442	0.313518	0.0473
1	rs9426785	22249589	G	A	50991	0.342874	0.03664
1	rs4839276	113524639	G	T	50920	0.228564	-0.05902
1	rs3820416	170707675	C	T	50990	0.270514	-0.03963
1	rs61820950	202709410	C	A	50082	0.455603	-0.04911
1	rs80256218	214110713	T	C	51228	0.0763157	-0.06792
2	rs74175027	23998092	A	T	50208	0.419833	-0.04117
2	rs6433041	169030920	T	C	51100	0.174217	-0.05646
2	rs7589104	218517174	C	T	50812	0.0685862	-0.08555
2	rs2177599	227893561	T	G	51175	0.480176	0.05636
2	rs7597716	228179901	C	T	50950	0.274455	0.04112
3	rs17279437	45814094	A	G	51255	0.106653	-0.10448
4	rs10212888	184924281	G	A	50274	0.342503	0.0393
5	rs62338626	11593100	C	T	50167	0.419668	0.04279
5	rs30373	55745334	G	C	49130	0.365144	0.03635
5	rs1070768	87135896	T	C	50482	0.0676776	-0.09791
5	rs17421627	87847586	G	T	51255	0.0731441	0.22711
5	rs3797315	148620224	G	T	51119	0.48617	0.04879
6	rs17507554	11394287	A	G	51255	0.0498586	0.09609

6	rs7752728	35477152	C	T	51013	0.210672	-0.05618
6	rs6918436	108868422	A	G	49065	0.298094	-0.04379
6	rs12193446	129820038	G	A	51255	0.0956395	0.07112
6	rs10872647	150079823	C	T	51134	0.336997	0.0447
7	rs58849528	8123126	G	A	50576	0.147076	0.05323
7	rs2428418	27079653	A	G	51114	0.356214	0.03907
7	rs6968945	46640900	C	T	50579	0.406849	-0.0527
7	rs2237468	50734362	T	C	50536	0.221387	0.06107
7	rs2079308	51017122	G	T	51004	0.291369	-0.04401
7	rs11761725	100039815	T	C	51250	0.193454	0.04749
7	rs34926272	129591807	C	G	50001	0.0236695	-0.12424
7	rs7807296	155617259	C	T	50447	0.428787	-0.03839
8	rs7816990	10463944	A	C	51255	0.25498	0.04192
8	rs423302	109121063	C	T	50948	0.255663	0.04343
9	rs1377193	21566440	G	A	51082	0.314113	0.05392
9	rs11145415	71754153	T	C	51090	0.208593	-0.04864
9	rs846758	102885162	A	G	50347	0.425269	-0.03882
9	rs10818846	126346105	C	T	51103	0.121891	-0.05916
10	rs1762186	28604448	T	C	49565	0.283416	0.03854
10	rs1947075	49741135	C	T	50374	0.352702	0.05333
10	rs12217769	85981842	G	A	51255	0.234904	0.04453
11	rs7127818	17004090	C	G	51216	0.289636	0.04312
11	rs10488688	31789114	C	G	50507	0.289554	-0.05208
11	rs11606813	69018588	T	C	50848	0.0429614	-0.09439
11	rs4936637	121447188	C	T	50916	0.22272	0.05091
12	rs3138142	56115585	T	C	50904	0.240158	0.14469
12	rs17368197	96196672	A	C	51199	0.192338	-0.05942
12	rs9788041	118473054	C	T	50298	0.253032	0.03985
13	rs9508027	28986781	A	C	51028	0.296729	-0.04595
14	rs59841088	23440049	A	G	50238	0.395866	-0.04137
14	rs1010053	61005625	G	A	51083	0.292818	-0.05301
14	rs887595	74666641	A	G	50862	0.177923	-0.06553
14	rs8021280	75290018	G	C	51175	0.463039	0.04685
15	rs1800407	28230318	T	C	51255	0.0832504	-0.06952
15	rs651237	35004765	A	C	50707	0.328968	0.04649
15	rs556217	53999768	T	C	51046	0.486375	-0.04645
15	rs7173827	59992337	A	G	50856	0.451982	0.04354
15	rs72763822	89143949	T	C	50997	0.342099	0.05215
16	rs4635359	80537760	C	A	50905	0.262518	0.05311
17	rs3785884	44057595	A	G	51192	0.230661	0.07574
17	rs9303434	59013669	G	A	50719	0.145192	0.06816
17	rs4791212	65975385	T	C	51045	0.201313	0.05223
17	rs9905786	79602063	G	T	51191	0.356	-0.08853
18	rs7237204	6730719	A	C	50647	0.477679	0.03471

18	rs150796733	56922290	T	C	49849	0.291641	0.06219
18	rs11662962	57052843	A	G	51100	0.127935	0.06712
19	rs4803297	40310084	T	C	50674	0.43102	-0.03544
21	rs13051661	45111518	A	T	50519	0.407391	-0.03806
22	rs12484778	30430598	T	C	50913	0.0837016	-0.06562
22	rs17728983	30619695	T	G	50901	0.0806369	-0.07673
22	rs9330813	46364161	A	G	51255	0.313023	0.04897
1	rs12741594	3053957	T	A	44689	0.0641097	0.08924
1	rs6690264	12613422	A	G	44752	0.423434	-0.04043
1	rs3125918	68846246	G	A	44683	0.399056	0.04285
1	rs4658101	92077409	A	G	44746	0.188006	0.11169
2	rs1346786	56108333	T	C	44462	0.287302	-0.05045
3	rs4858682	25066225	C	G	44483	0.471405	-0.046
3	rs35117224	32872880	G	T	44313	0.230598	-0.0444
3	rs7614897	99173177	G	T	44298	0.365874	0.05052
3	rs1436939	100608425	T	C	43855	0.168555	-0.05953
4	rs4368668	55098954	G	A	44731	0.213934	-0.04674
5	rs72759609	31952051	C	T	44503	0.100813	-0.07346
6	rs6927173	7222093	T	G	43165	0.447805	0.04301
6	rs2684249	122392511	C	T	44423	0.407604	-0.04925
8	rs10453110	72579250	T	C	44571	0.123679	0.05819
9	rs1360589	22045317	C	T	44642	0.429696	-0.08412
10	rs56238729	70001640	C	T	44745	0.235144	-0.1136
10	rs12778014	94950273	A	G	44245	0.336999	-0.04383
11	rs10835721	30934691	A	G	44445	0.274452	0.04587
11	rs12785585	65225717	T	G	44495	0.202708	-0.05041
11	rs2875238	130282078	T	C	43271	0.36142	-0.05318
12	rs1511582	84009783	C	T	44725	0.456009	-0.09769
13	rs9546383	36683268	C	T	44263	0.245498	0.04317
13	rs10162202	109267985	C	T	43686	0.275924	-0.04774
14	rs3811183	23452128	G	C	43727	0.400599	0.03864
14	rs12589689	61012559	T	A	44610	0.292109	0.04674
14	rs76320564	65115953	C	T	44428	0.16888	0.05491
16	rs8053277	51469726	T	C	44334	0.300638	0.07837
16	rs34900806	51672051	C	T	42911	0.487276	-0.04143
16	rs35640421	86472884	G	A	44752	0.0506458	0.0848
19	rs11084589	32030195	G	A	44429	0.328232	-0.04044
20	rs2326788	6470094	A	G	44254	0.371232	-0.06836
22	rs5762753	29100985	G	C	44692	0.358789	-0.08517
22	rs713875	30592487	C	G	44752	0.445712	0.04326
22	rs6000768	37922553	G	T	44651	0.247206	0.05395
1	rs12741594	3053957	T	A	44689	0.0641097	0.09215
1	rs6690264	12613422	A	G	44752	0.423434	-0.03906
1	rs3125918	68846246	G	A	44683	0.399056	0.0431

1	rs4658101	92077409	A	G	44746	0.188006	0.11575
2	rs1346786	56108333	T	C	44462	0.287302	-0.05194
3	rs4858682	25066225	C	G	44483	0.471405	-0.04594
3	rs56131903	32879823	T	A	43536	0.319689	-0.04226
3	rs7614897	99173177	G	T	44298	0.365874	0.04917
3	rs1436939	100608425	T	C	43855	0.168555	-0.06176
4	rs7678144	55102425	C	T	44710	0.213867	-0.04633
5	rs72759609	31952051	C	T	44503	0.100813	-0.07886
5	rs157851	55759715	T	C	43312	0.156827	-0.05186
6	rs56031192	1980208	A	T	44581	0.133196	-0.06146
6	rs1334576	7211818	A	G	44752	0.4294	0.04065
6	rs2684249	122392511	C	T	44423	0.407604	-0.05016
9	rs1360589	22045317	C	T	44642	0.429696	-0.08674
10	rs56238729	70001640	C	T	44745	0.235144	-0.11615
10	rs12778014	94950273	A	G	44245	0.336999	-0.04409
11	rs10835721	30934691	A	G	44445	0.274452	0.04401
11	rs150101522	65218101	T	C	44524	0.199948	-0.05255
11	rs4937515	130268147	G	C	43633	0.402769	-0.05149
12	rs1511582	84009783	C	T	44725	0.456009	-0.10097
13	rs12430710	36695575	G	C	43720	0.246969	0.04491
13	rs10162202	109267985	C	T	43686	0.275924	-0.04847
14	rs3811183	23452128	G	C	43727	0.400599	0.04193
14	rs12589689	61012559	T	A	44610	0.292109	0.04535
14	rs76320564	65115953	C	T	44428	0.16888	0.05526
16	rs8053277	51469726	T	C	44334	0.300638	0.08002
16	rs34900806	51672051	C	T	42911	0.487276	-0.04371
16	rs1728367	86386888	C	T	44274	0.089827	0.0654
19	rs11084589	32030195	G	A	44429	0.328232	-0.04139
20	rs2326788	6470094	A	G	44254	0.371232	-0.06889
22	rs5762753	29100985	G	C	44692	0.358789	-0.08688
22	rs713875	30592487	C	G	44752	0.445712	0.04429
22	rs6000764	37920097	C	T	44539	0.246952	0.05822
22	rs73175083	46383612	T	C	44349	0.308181	-0.04083
1	rs574350279	23506979	C	T	50830	0.343577	0.03875
1	rs11102510	113144012	T	C	50855	0.255304	-0.0435
1	rs72683434	113445614	T	C	50797	0.223606	-0.07238
1	rs61817386	170681971	A	C	50639	0.254764	-0.0416
1	rs1410996	196696933	A	G	50901	0.404108	0.04127
1	rs36003362	200367088	G	A	50901	0.373735	-0.05104
1	rs919655	214157972	A	G	50355	0.120733	0.06305
1	rs1513617	227447213	C	T	50803	0.335915	0.04768
2	rs4665251	24190263	C	A	50693	0.448691	0.06434
2	rs11892740	145222171	G	A	50901	0.360229	-0.04276
2	rs116350483	145338686	T	C	50901	0.0165714	-0.28379

2	rs10221681	169035382	T	C	50809	0.174192	-0.10508
2	rs11689553	170067947	G	C	50901	0.242991	-0.0474
2	rs7589104	218517174	C	T	50463	0.0689713	-0.09603
2	rs10190621	227914484	C	T	50550	0.482117	-0.03612
2	rs55895356	234224681	T	C	50859	0.0500895	-0.1261
3	rs11129176	25049310	A	G	50832	0.282745	0.04897
3	rs2370990	27690531	T	C	50422	0.242394	0.04619
3	rs58628422	150110582	C	T	50534	0.286599	0.04731
3	rs4680287	156329180	G	A	50728	0.273803	-0.04155
4	rs77766742	72543037	G	T	50541	0.0979304	-0.0596
5	rs13162470	38645365	C	T	50159	0.496132	-0.0413
5	rs30373	55745334	G	C	48784	0.365376	0.05586
5	rs63338061	71486228	T	C	49440	0.362075	0.03886
5	rs71580755	87002295	G	A	49778	0.0470087	-0.08549
5	rs17421627	87847586	G	T	50901	0.0733188	0.18051
5	rs28712135	88084016	A	T	48976	0.464544	0.05177
5	rs12523362	126095011	A	G	50687	0.1994	-0.0614
5	rs748066	148620535	A	G	50567	0.486295	0.04138
6	rs2326838	6901663	A	G	50844	0.363229	-0.04914
6	rs7752728	35477152	C	T	50662	0.210631	-0.07351
6	rs9398063	106528340	A	G	50673	0.0346141	-0.16402
6	rs11155679	150080017	C	T	50729	0.337371	0.03649
7	rs9639276	867033	T	C	50901	0.173788	0.05855
7	rs12531825	8005174	A	G	50284	0.120486	0.06471
7	rs2240093	51102820	A	C	49831	0.279806	-0.05557
7	rs35111986	100000274	A	C	50879	0.193587	0.06793
7	rs13229095	101513672	C	G	50445	0.136981	-0.06692
7	rs34926272	129591807	C	G	49645	0.0237083	-0.13785
8	rs62490815	10461395	A	G	50816	0.13662	0.05009
8	rs113958504	61591436	T	C	50248	0.136801	-0.07493
8	rs12547986	61986668	A	G	50379	0.294905	0.04547
8	rs442355	109128653	C	G	50672	0.255693	0.13277
9	rs10810150	14452729	T	C	50373	0.167451	0.05429
9	rs9657572	21553876	A	T	49466	0.319482	0.05633
9	rs6560395	77164177	C	T	50821	0.454379	0.0402
10	rs1947075	49741135	C	T	50020	0.351939	0.04873
10	rs56238729	70001640	C	T	50887	0.236043	0.07587
10	rs11200922	85961758	G	A	50695	0.460075	0.0577
10	rs4752581	123441729	G	A	49632	0.321859	0.04217
11	rs61879160	622044	T	C	50587	0.146965	0.05852
11	rs10488688	31789114	C	G	50153	0.289634	-0.04913
11	rs67808136	69055051	A	G	50789	0.0530233	-0.10975
11	rs2930972	69286205	C	T	50345	0.182471	0.0623
11	rs149950515	83873446	G	A	49864	0.0113408	0.16301

11	rs2276341	117375461	A	G	50658	0.451192	0.0418
11	rs3819132	117671489	A	G	50296	0.443504	0.04307
11	rs10790981	128821108	T	C	50721	0.330593	0.04557
12	rs3138142	56115585	T	C	50552	0.239921	0.21017
12	rs17368197	96196672	A	C	50844	0.192343	-0.07242
12	rs59966711	109729141	T	G	48669	0.323491	-0.03997
12	rs61952804	133436499	T	C	50740	0.172911	-0.04599
13	rs9319429	28973703	T	C	50898	0.296947	-0.06039
13	rs7325207	50112843	C	G	50738	0.159979	0.05794
13	rs9585141	100246997	G	A	49438	0.479196	-0.04043
13	rs9669831	114313349	G	A	49880	0.484292	-0.05494
14	rs8018562	21783280	T	C	50791	0.485391	-0.03905
14	rs28730443	36154749	A	G	50834	0.121513	0.05728
14	rs112109204	74101756	T	C	49783	0.0401442	0.12175
14	rs887595	74666641	A	G	50510	0.177985	-0.2717
14	rs118186707	74686575	A	G	50424	0.0285975	0.26692
14	rs12891131	75240090	A	G	50892	0.46299	-0.05745
15	rs7161746	34987432	A	T	49642	0.305729	-0.04092
15	rs556217	53999768	T	C	50686	0.486831	-0.0536
15	rs16956273	72117102	A	G	50901	0.235369	0.04594
15	rs7175019	89732471	C	T	50472	0.501932	0.04385
16	rs1134760	67964203	C	T	50901	0.167531	-0.05565
16	rs4635359	80537760	C	A	50558	0.262046	0.05283
16	rs3815795	85712105	T	C	50407	0.303281	-0.04512
17	rs12449582	6337965	T	G	50812	0.190427	-0.05719
17	rs35524223	44192590	A	T	50061	0.219582	0.07272
17	rs9303434	59013669	G	A	50375	0.144844	0.06416
17	rs6565596	79525118	T	G	48721	0.336313	0.04982
18	rs28774981	56936000	A	G	48981	0.277169	0.0683
18	rs11664409	57026673	G	T	50051	0.0899083	0.07005
21	rs8132685	34220618	C	T	50734	0.476406	0.04647
21	rs969060	45080852	T	G	50538	0.419279	-0.05255
22	rs4443100	23372864	G	C	50047	0.306862	-0.0387
22	rs13054238	30620891	A	G	48857	0.304317	0.07182
22	rs9330814	46364191	T	C	50575	0.312407	0.04151
1	rs2473851	23489869	G	A	51181	0.443895	0.04199
1	rs7519368	113150644	A	T	51014	0.274229	-0.03964
1	rs112806902	113508391	A	G	51103	0.220995	-0.07223
1	rs12409048	170699149	A	G	51319	0.255178	-0.03995
1	rs12134598	196681001	G	T	51289	0.173926	0.05391
1	rs71633898	200310898	G	C	50291	0.385377	-0.05898
1	rs12032598	222148552	C	A	48117	0.199763	0.05053
1	rs1513617	227447213	C	T	51221	0.33581	0.05766
2	rs4665251	24190263	C	A	51107	0.449342	0.06531

2	rs11892740	145222171	G	A	51319	0.360266	-0.04067
2	rs116350483	145338686	T	C	51319	0.0165631	-0.30508
2	rs12613885	169032900	T	G	51217	0.157301	-0.10108
2	rs11689553	170067947	G	C	51319	0.243048	-0.0423
2	rs7589104	218517174	C	T	50877	0.0685968	-0.09691
2	rs10203008	227956622	G	A	50789	0.486562	0.03704
2	rs55895356	234224681	T	C	51274	0.0499571	-0.11714
3	rs11129176	25049310	A	G	51250	0.282751	0.04815
3	rs2370990	27690531	T	C	50838	0.241984	0.04217
3	rs9845475	32842101	G	T	51319	0.136295	0.05394
3	rs35667547	64547477	C	G	51319	0.12508	0.05196
3	rs6784048	150117934	G	A	50998	0.28606	0.04883
3	rs4680287	156329180	G	A	51143	0.273713	-0.03877
3	rs729257	156828823	A	G	50838	0.397272	0.03494
4	rs7678144	55102425	C	T	51268	0.21372	0.04144
5	rs327264	38653601	T	G	50914	0.494294	0.04195
5	rs30373	55745334	G	C	49192	0.36509	0.05301
5	rs63338061	71486228	T	C	49841	0.362222	0.04149
5	rs79327504	87847988	A	G	51195	0.072009	0.17925
5	rs28712135	88084016	A	T	49396	0.464218	0.04722
5	rs12523362	126095011	A	G	51104	0.199446	-0.05999
5	rs2400826	148615479	G	A	51032	0.486009	0.04142
6	rs2326838	6901663	A	G	51263	0.36344	-0.04139
6	rs7752728	35477152	C	T	51076	0.210745	-0.07251
6	rs9398063	106528340	A	G	51094	0.0347888	-0.16597
7	rs7809344	901574	T	C	51222	0.182636	0.05301
7	rs12531825	8005174	A	G	50698	0.120606	0.0672
7	rs2876869	50706178	T	C	50822	0.45012	0.03582
7	rs62448278	51098849	A	G	50211	0.281293	-0.0536
7	rs62482222	100197866	A	G	50430	0.131231	-0.07891
7	rs13229095	101513672	C	G	50862	0.137008	-0.06519
7	rs34926272	129591807	C	G	50063	0.0236103	-0.13815
8	rs7816990	10463944	A	C	51319	0.254847	0.04233
8	rs113958504	61591436	T	C	50664	0.136991	-0.07
8	rs12547986	61986668	A	G	50789	0.294522	0.05153
8	rs13263941	109121945	C	T	50943	0.255462	0.12793
9	rs1377193	21566440	G	A	51146	0.314199	0.05716
9	rs717299	77185933	G	A	51194	0.455503	0.04385
10	rs7067692	45430699	A	G	51085	0.219448	-0.04153
10	rs1947075	49741135	C	T	50436	0.352556	0.04614
10	rs1900003	70004551	C	A	51081	0.235136	0.07642
10	rs11200922	85961758	G	A	51109	0.459303	0.05626
10	rs4752581	123441729	G	A	50029	0.321773	0.0404
11	rs61879160	622044	T	C	51003	0.146982	0.05988

11	rs10488688	31789114	C	G	50568	0.289412	-0.04848
11	rs67808136	69055051	A	G	51208	0.0532436	-0.11821
11	rs2930972	69286205	C	T	50755	0.182672	0.06062
11	rs2155394	117378116	A	G	51129	0.451886	0.04349
11	rs7125694	117670657	C	T	50940	0.490724	0.0464
11	rs10790981	128821108	T	C	51137	0.330319	0.04668
12	rs3138142	56115585	T	C	50965	0.240126	0.20678
12	rs17370487	96272463	A	G	51319	0.192346	-0.07199
12	rs59966711	109729141	T	G	49094	0.323655	-0.03969
12	rs77770402	133422998	G	A	49921	0.382124	-0.03631
13	rs2296284	28963676	A	G	50963	0.29437	-0.06009
13	rs6561539	50112129	C	G	51244	0.157218	0.05307
13	rs7327333	100244965	C	T	48933	0.488351	0.03654
13	rs7399672	114467309	C	A	50728	0.457913	-0.05508
14	rs8018562	21783280	T	C	51209	0.485325	-0.03641
14	rs28730443	36154749	A	G	51248	0.121429	0.06744
14	rs17825846	65598752	G	C	51244	0.349924	-0.03559
14	rs112109204	74101756	T	C	50189	0.0400685	0.12212
14	rs887595	74666641	A	G	50927	0.17801	-0.27872
14	rs118186707	74686575	A	G	50832	0.0286335	0.26242
14	rs973879	75297727	T	C	51253	0.462275	-0.05736
15	rs634990	35006073	C	T	51319	0.488455	-0.04073
15	rs556217	53999768	T	C	51110	0.486431	-0.05379
15	rs7183832	59984780	A	C	49201	0.405215	0.04206
15	rs16956273	72117102	A	G	51319	0.235264	0.04323
15	rs4932480	89723858	T	C	49987	0.486506	0.04178
15	rs1372613	101204835	T	C	51016	0.301131	0.04094
16	rs1134760	67964203	C	T	51319	0.167891	-0.05874
16	rs9921725	80489718	T	A	51162	0.483855	-0.04962
16	rs9930110	85720780	C	T	50549	0.291895	-0.05204
17	rs12449582	6337965	T	G	51226	0.190567	-0.06098
17	rs8068292	26321436	T	C	51235	0.279818	0.0401
17	rs62054372	43798775	C	G	50652	0.221314	0.07574
17	rs9303434	59013669	G	A	50782	0.145081	0.055
17	rs59588726	79505768	A	G	50058	0.236855	0.05388
18	rs17696543	56971398	T	C	49999	0.179794	-0.08101
19	rs76076446	3771586	A	G	51319	0.0224673	-0.11692
20	rs1327226	10920872	C	T	50900	0.078556	-0.06409
21	rs8132685	34220618	C	T	51152	0.476404	0.04173
21	rs2838338	45075582	T	C	51062	0.421076	-0.05086
22	rs4443100	23372864	G	C	50459	0.307002	-0.04098
22	rs13054238	30620891	A	G	49256	0.304511	0.07298
22	rs9330813	46364161	A	G	51319	0.313013	0.03955
1	rs72637739	9165685	A	G	50579	0.215781	-0.05213

1	rs11587687	110147801	A	G	50482	0.442692	0.04364
1	rs7519368	113150644	A	T	50278	0.273678	-0.07176
1	rs2149036	113479886	A	T	50557	0.222205	-0.08139
1	rs1329428	196702810	T	C	50579	0.404209	0.07689
2	rs11125168	23922383	C	T	50531	0.451782	-0.04842
2	rs817741	103761767	T	G	50579	0.507889	0.03597
2	rs72886128	165822558	G	A	49721	0.302639	0.04262
2	rs6728947	169059511	C	G	49796	0.389921	0.04957
2	rs7588399	198954831	T	C	48522	0.169902	0.04984
2	rs1864251	216829638	G	A	50327	0.0929322	0.10097
2	rs7564805	234228946	G	A	50579	0.0495957	-0.19523
3	rs56124905	14385648	G	A	50239	0.18623	-0.04752
3	rs6771073	58458043	A	G	50335	0.0886063	-0.06524
3	rs348882	100971181	C	G	50436	0.210455	-0.06804
3	rs3806709	129109764	A	C	49984	0.146857	-0.09132
3	rs6778948	150134304	G	A	50567	0.242678	-0.04362
3	rs56351888	183904226	T	C	50574	0.167863	-0.05387
5	rs250346	16789362	T	C	48623	0.474364	-0.03694
5	rs113100410	17168087	G	A	50579	0.0714032	-0.07913
5	rs201456755	88043466	C	T	50296	0.284605	-0.04039
6	rs2326838	6901663	A	G	50522	0.36303	-0.06049
6	rs556679	31894355	T	C	50447	0.118738	0.06106
6	rs749523	35489775	G	A	50055	0.314484	-0.0382
6	rs9381202	42580545	G	T	50561	0.313789	-0.06649
6	rs111791023	56714017	C	A	50390	0.18078	-0.04698
6	rs1581836	76796339	C	T	50314	0.311583	0.06142
7	rs1964242	99976703	A	G	50491	0.18638	0.06643
8	rs57819090	10469340	A	G	50065	0.126186	0.09822
10	rs10793567	45415207	T	C	49703	0.284993	-0.04046
10	rs7073076	85962650	G	T	50361	0.460197	0.09328
10	rs60401382	124227624	T	C	50025	0.227426	-0.07297
11	rs11230527	60720893	T	G	49206	0.181594	-0.05619
11	rs12574286	76937602	C	G	50226	0.211365	-0.0618
11	rs6415994	88972346	T	C	49898	0.30867	-0.06732
12	rs2080402	345175	T	C	50198	0.440555	-0.0583
12	rs3138142	56115585	T	C	50232	0.239927	0.11003
13	rs9556964	99159306	T	C	50171	0.324381	0.04146
13	rs9669831	114313349	G	A	49568	0.484304	-0.06152
14	rs28711886	61072658	G	A	48551	0.318171	-0.04741
15	rs1800407	28230318	T	C	50579	0.0831373	0.117
15	rs111466394	52742888	G	A	50133	0.165879	0.05741
15	rs3825991	89761664	A	C	48892	0.475027	0.0916
15	rs11433	101716583	A	G	50388	0.437932	-0.04268
16	rs142963458	84561361	T	C	49426	0.0310464	-0.20153

17	rs12449582	6337965	T	G	50491	0.190569	-0.04762
17	rs4794029	47280301	T	C	50050	0.317323	-0.04872
17	rs111812808	79585834	A	G	50534	0.342848	-0.08147
20	rs7828	10619014	C	A	50122	0.328309	-0.04631
20	rs6077977	10930708	G	A	50551	0.483324	0.07313
21	rs2251253	45092257	T	A	50520	0.422338	-0.0456
1	rs6669802	110163076	A	G	49552	0.474088	-0.03718
1	rs6668533	113064468	A	G	50677	0.25995	-0.07173
1	rs7542229	113463749	T	C	50715	0.222725	-0.07573
1	rs1329428	196702810	T	C	50743	0.404095	0.07076
2	rs12988200	24100770	T	A	50703	0.255064	-0.04874
2	rs72886118	165819021	T	A	50093	0.303156	0.04108
2	rs10930308	169017436	A	G	50513	0.388979	0.04523
2	rs1864251	216829638	G	A	50493	0.0928644	0.08641
2	rs7583380	234223886	A	G	50659	0.0488363	-0.1965
3	rs4684200	14338285	C	T	50170	0.390482	0.03621
3	rs13094898	58290143	A	G	50691	0.0821546	-0.07124
3	rs2660806	100926254	A	C	50734	0.209485	-0.06367
3	rs68155216	129217968	C	T	50370	0.112775	-0.09619
3	rs6778948	150134304	G	A	50731	0.243145	-0.0467
5	rs186024631	17154247	T	C	50502	0.0244743	-0.12926
5	rs115237855	17186336	A	G	50743	0.0242201	-0.1554
5	rs304152	88124123	G	T	50560	0.304134	-0.04031
6	rs2326838	6901663	A	G	50687	0.36317	-0.06023
6	rs556679	31894355	T	C	50612	0.118668	0.07541
6	rs394754	42659979	G	T	50587	0.286348	-0.06796
6	rs13194625	56685244	G	A	50675	0.177454	-0.05519
6	rs1952134	76786554	T	A	50505	0.311732	0.05504
6	rs11153159	109376647	G	C	50395	0.113999	-0.05759
7	rs111963714	99948655	G	T	49973	0.210143	0.06103
8	rs57819090	10469340	A	G	50218	0.125841	0.08145
10	rs914696	45425266	G	T	50743	0.195544	-0.05299
10	rs16915682	62650959	G	A	49953	0.246592	0.04282
10	rs4562752	85978809	G	T	50572	0.471862	0.09055
10	rs200227426	124213671	A	C	49618	0.20291	-0.08056
11	rs12788170	635569	C	A	48852	0.25045	0.04111
11	rs11230527	60720893	T	G	49372	0.181793	-0.05674
11	rs11235841	73367122	C	T	50530	0.128973	-0.05806
11	rs61629638	76939409	T	C	50417	0.211962	-0.07127
11	rs1847142	89021574	A	G	50438	0.327997	-0.0762
12	rs7969761	355842	C	T	50370	0.447369	-0.06016
12	rs3138142	56115585	T	C	50391	0.240221	0.1157
13	rs7337610	28962666	T	C	49971	0.373236	-0.03982
13	rs9556964	99159306	T	C	50338	0.323533	0.03749

13	rs9669831	114313349	G	A	49737	0.484066	-0.05729
14	rs1254260	60835737	A	G	50664	0.288173	-0.05137
15	rs1800407	28230318	T	C	50743	0.0831346	0.12447
15	rs117089270	52804249	A	G	50419	0.167397	0.05838
15	rs12898755	63574641	A	G	50659	0.207091	0.05468
15	rs3825991	89761664	A	C	49055	0.475161	0.08199
15	rs7171515	101769618	T	C	50445	0.366865	0.04009
16	rs142963458	84561361	T	C	49576	0.0310634	-0.19409
17	rs12449580	6337247	G	C	50743	0.190095	-0.04471
17	rs4794029	47280301	T	C	50214	0.31765	-0.04894
17	rs11150803	79621160	A	C	50098	0.475648	0.08574
19	rs778811	5828895	T	G	50582	0.190038	-0.04436
19	rs12978094	49313239	C	A	50743	0.0457403	-0.08323
20	rs1232603	10612963	T	C	50070	0.324166	-0.04894
20	rs6077977	10930708	G	A	50713	0.483781	0.07125
21	rs2251253	45092257	T	A	50683	0.422538	-0.04223
1	rs34361557	9290707	G	A	47169	0.400454	-0.04229
2	rs6721680	172543913	G	T	50939	0.282318	0.04039
2	rs1894774	191591546	A	G	50324	0.479721	0.03467
3	rs17279437	45814094	A	G	51047	0.10651	-0.12088
4	rs61791630	32756380	A	T	51031	0.158149	0.04814
4	rs12498322	159845612	A	C	50915	0.427418	0.03777
4	rs10212888	184924281	G	A	50059	0.342716	0.05826
5	rs2004187	2612747	A	C	51047	0.403951	-0.03486
5	rs2190989	11584342	C	T	51047	0.452093	0.03436
5	rs17318932	87688421	T	C	51047	0.0654299	-0.08592
5	rs75048985	87847992	A	T	50925	0.0721453	0.16506
5	rs254776	88006893	G	C	50417	0.306028	-0.03846
6	rs115531193	34651311	A	T	51039	0.113364	0.06245
6	rs1536057	108885623	T	C	50785	0.278389	-0.05712
6	rs12193446	129820038	G	A	51047	0.0955198	0.07105
7	rs11762530	46630602	G	C	50337	0.40646	-0.09611
7	rs17725573	50986017	G	C	50855	0.255825	0.07239
9	rs6478995	102792856	A	C	51025	0.42733	-0.03697
10	rs607163	3550079	T	A	50162	0.259758	0.04129
11	rs56213534	17324948	G	A	50722	0.300708	0.03799
11	rs3862368	88529474	A	C	50103	0.325809	-0.04156
11	rs4096587	117437919	C	A	50993	0.0950228	-0.06199
12	rs3138142	56115585	T	C	50695	0.24032	0.05014
12	rs56882977	118472388	C	G	50279	0.174795	0.0571
14	rs887595	74666641	A	G	50655	0.177939	0.06996
14	rs8021126	104546296	G	A	46280	0.14798	0.05162
15	rs1800407	28230318	T	C	51047	0.0830999	-0.10371
15	rs1348003	89113441	C	G	50209	0.319982	0.03956

16	rs246184	14395077	C	G	49046	0.319506	0.038
17	rs7225002	44189067	G	A	50756	0.414355	0.03802
17	rs9905786	79602063	G	T	50982	0.3554	-0.10573
18	rs11876004	57038761	G	A	50885	0.181281	-0.04974
19	rs62106867	40324476	T	C	50957	0.465912	0.03896
21	rs1625244	47364499	T	C	51027	0.2785	-0.05506

se.exposure	pval.exposure	exposure	id.exposure	mr_keep.exposure	pval_origin.exposure
0.00923087	3.12018E-17	Thickness betwee	ELM_ISOS_th	TRUE	reported
0.00623763	4.52721E-08	Thickness betwee	ELM_ISOS_th	TRUE	reported
0.00681449	1.14378E-09	Thickness betwee	ELM_ISOS_th	TRUE	reported
0.00677073	3.93484E-09	Thickness betwee	ELM_ISOS_th	TRUE	reported
0.00632736	3.98202E-08	Thickness betwee	ELM_ISOS_th	TRUE	reported
0.00663496	3.17791E-15	Thickness betwee	ELM_ISOS_th	TRUE	reported
0.00809352	2.02157E-11	Thickness betwee	ELM_ISOS_th	TRUE	reported
0.00825766	6.41937E-11	Thickness betwee	ELM_ISOS_th	TRUE	reported
0.00744424	1.32835E-11	Thickness betwee	ELM_ISOS_th	TRUE	reported
0.00623315	5.61076E-13	Thickness betwee	ELM_ISOS_th	TRUE	reported
0.00744464	1.1553E-10	Thickness betwee	ELM_ISOS_th	TRUE	reported
0.0094797	2.23547E-08	Thickness betwee	ELM_ISOS_th	TRUE	reported
0.00731599	3.77721E-12	Thickness betwee	ELM_ISOS_th	TRUE	reported
0.00792832	1.48746E-08	Thickness betwee	ELM_ISOS_th	TRUE	reported
0.00684562	4.45816E-13	Thickness betwee	ELM_ISOS_th	TRUE	reported
0.00629037	4.05741E-09	Thickness betwee	ELM_ISOS_th	TRUE	reported
0.00819739	6.52053E-42	Thickness betwee	ELM_ISOS_th	TRUE	reported
0.00626574	1.31341E-15	Thickness betwee	ELM_ISOS_th	TRUE	reported
0.00713612	1.74395E-10	Thickness betwee	ELM_ISOS_th	TRUE	reported
0.00625499	5.75493E-20	Thickness betwee	ELM_ISOS_th	TRUE	reported
0.00685547	3.4115E-11	Thickness betwee	ELM_ISOS_th	TRUE	reported
0.00998159	1.42778E-10	Thickness betwee	ELM_ISOS_th	TRUE	reported
0.0073443	4.88767E-08	Ganglion cell-inne	GCIPL_thickn	TRUE	reported
0.00685397	3.72403E-10	Ganglion cell-inne	GCIPL_thickn	TRUE	reported
0.00617934	3.66316E-08	Ganglion cell-inne	GCIPL_thickn	TRUE	reported
0.00912712	1.99524E-10	Ganglion cell-inne	GCIPL_thickn	TRUE	reported
0.00995422	2.96579E-36	Ganglion cell-inne	GCIPL_thickn	TRUE	reported
0.00623888	2.46854E-09	Ganglion cell-inne	GCIPL_thickn	TRUE	reported
0.00623891	5.57811E-10	Ganglion cell-inne	GCIPL_thickn	TRUE	reported
0.00655809	4.5525E-17	Ganglion cell-inne	GCIPL_thickn	TRUE	reported
0.0063554	2.73973E-08	Ganglion cell-inne	GCIPL_thickn	TRUE	reported
0.0123997	2.65711E-12	Ganglion cell-inne	GCIPL_thickn	TRUE	reported
0.0119519	5.31218E-45	Ganglion cell-inne	GCIPL_thickn	TRUE	reported
0.00977258	1.67387E-08	Ganglion cell-inne	GCIPL_thickn	TRUE	reported
0.00689995	6.4592E-17	Ganglion cell-inne	GCIPL_thickn	TRUE	reported
0.0104903	1.2897E-11	Ganglion cell-inne	GCIPL_thickn	TRUE	reported
0.00632166	1.10407E-49	Ganglion cell-inne	GCIPL_thickn	TRUE	reported
0.00714449	1.68378E-22	Ganglion cell-inne	GCIPL_thickn	TRUE	reported
0.00630149	2.34406E-08	Ganglion cell-inne	GCIPL_thickn	TRUE	reported
0.00762782	1.83427E-10	Ganglion cell-inne	GCIPL_thickn	TRUE	reported
0.00622732	7.35443E-12	Ganglion cell-inne	GCIPL_thickn	TRUE	reported

0.00617453	3.30892E-09	Ganglion cell-inne	GCIPL_thickn	TRUE	reported
0.00667287	5.55717E-10	Ganglion cell-inne	GCIPL_thickn	TRUE	reported
0.00678217	6.01198E-09	Ganglion cell-inne	GCIPL_thickn	TRUE	reported
0.00727763	2.31426E-11	Ganglion cell-inne	GCIPL_thickn	TRUE	reported
0.00818944	2.78597E-11	Ganglion cell-inne	GCIPL_thickn	TRUE	reported
0.00811053	9.91018E-15	Ganglion cell-inne	GCIPL_thickn	TRUE	reported
0.00912338	6.57096E-10	Ganglion cell-inne	GCIPL_thickn	TRUE	reported
0.0111285	8.69049E-22	Ganglion cell-inne	GCIPL_thickn	TRUE	reported
0.0061783	1.00953E-09	Ganglion cell-inne	GCIPL_thickn	TRUE	reported
0.00664842	3.62558E-09	Ganglion cell-inne	GCIPL_thickn	TRUE	reported
0.00751088	2.90431E-11	Ganglion cell-inne	GCIPL_thickn	TRUE	reported
0.00749078	9.72668E-09	Ganglion cell-inne	GCIPL_thickn	TRUE	reported
0.00747126	1.30384E-08	Ganglion cell-inne	GCIPL_thickn	TRUE	reported
0.00646778	8.06431E-53	Ganglion cell-inne	GCIPL_thickn	TRUE	reported
0.00802394	5.14985E-11	Ganglion cell-inne	GCIPL_thickn	TRUE	reported
0.00618774	2.90807E-08	Ganglion cell-inne	GCIPL_thickn	TRUE	reported
0.0063422	2.3874E-09	Ganglion cell-inne	GCIPL_thickn	TRUE	reported
0.00625842	1.48624E-11	Ganglion cell-inne	GCIPL_thickn	TRUE	reported
0.0100146	2.06435E-08	Ganglion cell-inne	GCIPL_thickn	TRUE	reported
0.00687112	8.07269E-21	Ganglion cell-inne	GCIPL_thickn	TRUE	reported
0.0114892	5.66349E-09	Inner nuclear laye	INL_thickness	TRUE	reported
0.00746455	5.12096E-23	Inner nuclear laye	INL_thickness	TRUE	reported
0.0064382	4.21681E-08	Inner nuclear laye	INL_thickness	TRUE	reported
0.00745406	2.85359E-09	Inner nuclear laye	INL_thickness	TRUE	reported
0.0161557	2.12038E-26	Inner nuclear laye	INL_thickness	TRUE	reported
0.00634629	2.54164E-09	Inner nuclear laye	INL_thickness	TRUE	reported
0.0117842	8.79488E-33	Inner nuclear laye	INL_thickness	TRUE	reported
0.0114068	1.64472E-10	Inner nuclear laye	INL_thickness	TRUE	reported
0.00768742	2.7353E-10	Inner nuclear laye	INL_thickness	TRUE	reported
0.00629208	2.91054E-09	Inner nuclear laye	INL_thickness	TRUE	reported
0.00697435	6.12407E-12	Inner nuclear laye	INL_thickness	TRUE	reported
0.00714022	3.90655E-11	Inner nuclear laye	INL_thickness	TRUE	reported
0.00632981	5.00649E-09	Inner nuclear laye	INL_thickness	TRUE	reported
0.00705962	2.04236E-08	Inner nuclear laye	INL_thickness	TRUE	reported
0.00709718	6.71788E-10	Inner nuclear laye	INL_thickness	TRUE	reported
0.0083949	9.78604E-10	Inner nuclear laye	INL_thickness	TRUE	reported
0.00719322	8.37602E-09	Inner nuclear laye	INL_thickness	TRUE	reported
0.00637517	1.56318E-08	Inner nuclear laye	INL_thickness	TRUE	reported
0.00636226	1.92012E-08	Inner nuclear laye	INL_thickness	TRUE	reported
0.00675738	8.89981E-13	Inner nuclear laye	INL_thickness	TRUE	reported
0.00630521	2.12397E-09	Inner nuclear laye	INL_thickness	TRUE	reported
0.00652498	1.61064E-08	Inner nuclear laye	INL_thickness	TRUE	reported
0.0125747	1.24185E-22	Inner nuclear laye	INL_thickness	TRUE	reported
0.0254884	3.56535E-11	Inner nuclear laye	INL_thickness	TRUE	reported

0.0119873	2.9172E-212	Inner nuclear laye	INL_thickness	TRUE	reported
0.00632502	3.39151E-15	Inner nuclear laye	INL_thickness	TRUE	reported
0.0144041	4.72318E-12	Inner nuclear laye	INL_thickness	TRUE	reported
0.00633297	3.74257E-38	Inner nuclear laye	INL_thickness	TRUE	reported
0.0106438	2.22318E-18	Inner nuclear laye	INL_thickness	TRUE	reported
0.00660633	1.6403E-12	Inner nuclear laye	INL_thickness	TRUE	reported
0.00957853	4.83793E-10	Inner nuclear laye	INL_thickness	TRUE	reported
0.00651791	9.95874E-11	Inner nuclear laye	INL_thickness	TRUE	reported
0.00667015	1.48529E-08	Inner nuclear laye	INL_thickness	TRUE	reported
0.00636838	1.31162E-21	Inner nuclear laye	INL_thickness	TRUE	reported
0.00681623	1.22881E-08	Inner nuclear laye	INL_thickness	TRUE	reported
0.00639935	6.35295E-13	Inner nuclear laye	INL_thickness	TRUE	reported
0.00707879	3.37513E-09	Inner nuclear laye	INL_thickness	TRUE	reported
0.0079334	9.05873E-09	Inner nuclear laye	INL_thickness	TRUE	reported
0.00689984	3.3094E-10	Inner nuclear laye	INL_thickness	TRUE	reported
0.0110031	2.49764E-13	Inner nuclear laye	INL_thickness	TRUE	reported
0.00658116	1.20286E-08	Inner nuclear laye	INL_thickness	TRUE	reported
0.00690491	3.8208E-09	Inner nuclear laye	INL_thickness	TRUE	reported
0.00629486	1.06996E-12	Inner nuclear laye	INL_thickness	TRUE	reported
0.0161141	2.77374E-09	Inner nuclear laye	INL_thickness	TRUE	reported
0.00733952	4.8621E-20	Inner nuclear laye	INL_thickness	TRUE	reported
0.00739171	7.53112E-10	Inner nuclear laye	INL_thickness	TRUE	reported
0.00683053	1.14044E-32	Inner nuclear laye	INL_thickness	TRUE	reported
0.00822717	9.78505E-31	Inner nuclear laye	INL_thickness	TRUE	reported
0.00630236	4.30228E-08	Inner nuclear laye	INL_thickness	TRUE	reported
0.0116147	1.75106E-08	Inner nuclear laye	INL_thickness	TRUE	reported
0.00893308	3.70001E-15	Inner nuclear laye	INL_thickness	TRUE	reported
0.00626625	2.70862E-10	Inner nuclear laye	INL_thickness	TRUE	reported
0.00693302	2.33271E-09	Inner nuclear laye	INL_thickness	TRUE	reported
0.0099465	4.0705E-08	Inner nuclear laye	INL_thickness	TRUE	reported
0.00642842	2.05842E-10	Inner nuclear laye	INL_thickness	TRUE	reported
0.00627115	3.32696E-15	Inner nuclear laye	INL_thickness	TRUE	reported
0.00660816	3.33548E-12	Inner nuclear laye	INL_thickness	TRUE	reported
0.00794327	2.08964E-09	Inner nuclear laye	INL_thickness	TRUE	reported
0.00712531	3.31843E-09	Inner nuclear laye	INL_thickness	TRUE	reported
0.00778901	1.14328E-09	Inner nuclear laye	INL_thickness	TRUE	reported
0.0065542	7.2462E-30	Inner nuclear laye	INL_thickness	TRUE	reported
0.00632442	1.56415E-08	Inner nuclear laye	INL_thickness	TRUE	reported
0.00825735	5.57186E-12	Inner nuclear laye	INL_thickness	TRUE	reported
0.00634659	5.12026E-12	Inner nuclear laye	INL_thickness	TRUE	reported
0.00795176	2.34776E-15	Inner nuclear laye	INL_thickness	TRUE	reported
0.0066819	1.65452E-08	Inner nuclear laye	INL_thickness	TRUE	reported
0.00760569	5.055E-23	Inner nuclear laye	INL_thickness	TRUE	reported
0.00737963	4.67812E-11	Inner nuclear laye	INL_thickness	TRUE	reported

0.0160951	1.14701E-33	Inner nuclear laye	INL_thickness	TRUE	reported
0.00632032	4.21256E-09	Inner nuclear laye	INL_thickness	TRUE	reported
0.0117618	1.37169E-33	Inner nuclear laye	INL_thickness	TRUE	reported
0.00763359	5.74075E-13	Inner nuclear laye	INL_thickness	TRUE	reported
0.00626182	2.09345E-10	Inner nuclear laye	INL_thickness	TRUE	reported
0.00694047	5.22741E-13	Inner nuclear laye	INL_thickness	TRUE	reported
0.0182314	4.13093E-08	Inner nuclear laye	INL_thickness	TRUE	reported
0.00710898	1.58576E-12	Inner nuclear laye	INL_thickness	TRUE	reported
0.00753319	3.08501E-08	Inner nuclear laye	INL_thickness	TRUE	reported
0.00621523	6.8321E-09	Inner nuclear laye	INL_thickness	TRUE	reported
0.00835233	1.06745E-08	Inner nuclear laye	INL_thickness	TRUE	reported
0.015243	2.38048E-08	Inner nuclear laye	INL_thickness	TRUE	reported
0.00634565	3.15052E-09	Inner nuclear laye	INL_thickness	TRUE	reported
0.00629466	4.36171E-11	Inner nuclear laye	INL_thickness	TRUE	reported
0.00623556	2.33704E-08	Inner nuclear laye	INL_thickness	TRUE	reported
0.00670695	2.67361E-13	Inner nuclear laye	INL_thickness	TRUE	reported
0.00627644	2.32941E-09	Inner nuclear laye	INL_thickness	TRUE	reported
0.00823006	1.57619E-09	Inner nuclear laye	INL_thickness	TRUE	reported
0.0125156	1.00845E-22	Inner nuclear laye	INL_thickness	TRUE	reported
0.0254058	3.309E-12	Inner nuclear laye	INL_thickness	TRUE	reported
0.0119463	5.6832E-210	Inner nuclear laye	INL_thickness	TRUE	reported
0.00643505	2.34212E-16	Inner nuclear laye	INL_thickness	TRUE	reported
0.0143483	1.35435E-15	Inner nuclear laye	INL_thickness	TRUE	reported
0.00657747	7.92215E-09	Inner nuclear laye	INL_thickness	TRUE	reported
0.00630656	1.48112E-35	Inner nuclear laye	INL_thickness	TRUE	reported
0.0105882	3.16809E-21	Inner nuclear laye	INL_thickness	TRUE	reported
0.00659996	3.63326E-11	Inner nuclear laye	INL_thickness	TRUE	reported
0.00912397	6.39034E-10	Inner nuclear laye	INL_thickness	TRUE	reported
0.00671257	3.49082E-09	Inner nuclear laye	INL_thickness	TRUE	reported
0.00663487	8.39942E-09	Inner nuclear laye	INL_thickness	TRUE	reported
0.00633965	8.44454E-19	Inner nuclear laye	INL_thickness	TRUE	reported
0.00627857	3.81923E-14	Inner nuclear laye	INL_thickness	TRUE	reported
0.00657483	2.36234E-08	Inner nuclear laye	INL_thickness	TRUE	reported
0.00726112	9.98601E-10	Inner nuclear laye	INL_thickness	TRUE	reported
0.00682249	4.65875E-10	Inner nuclear laye	INL_thickness	TRUE	reported
0.00673427	1.69672E-08	Inner nuclear laye	INL_thickness	TRUE	reported
0.0109437	1.11798E-09	Inner nuclear laye	INL_thickness	TRUE	reported
0.00654595	8.81899E-10	Inner nuclear laye	INL_thickness	TRUE	reported
0.00686741	1.86885E-10	Inner nuclear laye	INL_thickness	TRUE	reported
0.00625911	3.38532E-11	Inner nuclear laye	INL_thickness	TRUE	reported
0.0158235	1.62385E-14	Inner nuclear laye	INL_thickness	TRUE	reported
0.00634407	8.17891E-09	Inner nuclear laye	INL_thickness	TRUE	reported
0.00730588	8.98746E-18	Inner nuclear laye	INL_thickness	TRUE	reported
0.00735724	5.01812E-09	Inner nuclear laye	INL_thickness	TRUE	reported

0.00685957	7.37008E-32	Inner nuclear laye	INL_thickness	TRUE	reported
0.0110782	5.07489E-09	Inner nuclear laye	INL_thickness	TRUE	reported
0.00818075	3.98213E-23	Inner nuclear laye	INL_thickness	TRUE	reported
0.0115666	1.78025E-08	Inner nuclear laye	INL_thickness	TRUE	reported
0.00888415	4.09678E-14	Inner nuclear laye	INL_thickness	TRUE	reported
0.0062344	7.91789E-10	Inner nuclear laye	INL_thickness	TRUE	reported
0.00625202	1.37766E-11	Inner nuclear laye	INL_thickness	TRUE	reported
0.00638747	3.88792E-09	Inner nuclear laye	INL_thickness	TRUE	reported
0.00623671	1.38131E-16	Inner nuclear laye	INL_thickness	TRUE	reported
0.0065742	6.00104E-10	Inner nuclear laye	INL_thickness	TRUE	reported
0.00790898	1.2612E-09	Inner nuclear laye	INL_thickness	TRUE	reported
0.00726631	2.89453E-11	Inner nuclear laye	INL_thickness	TRUE	reported
0.00775785	2.00289E-09	Inner nuclear laye	INL_thickness	TRUE	reported
0.0065271	3.24277E-30	Inner nuclear laye	INL_thickness	TRUE	reported
0.00629307	6.35852E-10	Inner nuclear laye	INL_thickness	TRUE	reported
0.00812398	4.48366E-12	Inner nuclear laye	INL_thickness	TRUE	reported
0.00631451	2.90298E-11	Inner nuclear laye	INL_thickness	TRUE	reported
0.00791135	1.67658E-12	Inner nuclear laye	INL_thickness	TRUE	reported
0.00671899	2.01466E-11	Inner nuclear laye	INL_thickness	TRUE	reported
0.00640849	1.26161E-08	Retinal nerve fibre	RNFL_thickne	TRUE	reported
0.00682829	4.12334E-09	Retinal nerve fibre	RNFL_thickne	TRUE	reported
0.00674355	1.97774E-08	Retinal nerve fibre	RNFL_thickne	TRUE	reported
0.00637861	2.92954E-11	Retinal nerve fibre	RNFL_thickne	TRUE	reported
0.0100624	3.54692E-10	Retinal nerve fibre	RNFL_thickne	TRUE	reported
0.00779855	3.68703E-11	Retinal nerve fibre	RNFL_thickne	TRUE	reported
0.0129123	1.64611E-09	Retinal nerve fibre	RNFL_thickne	TRUE	reported
0.00633675	3.97429E-14	Retinal nerve fibre	RNFL_thickne	TRUE	reported
0.00715451	1.91118E-09	Retinal nerve fibre	RNFL_thickne	TRUE	reported
0.00665903	1.20617E-08	Retinal nerve fibre	RNFL_thickne	TRUE	reported
0.00718691	8.95743E-32	Retinal nerve fibre	RNFL_thickne	TRUE	reported
0.00738095	6.71746E-32	Retinal nerve fibre	RNFL_thickne	TRUE	reported
0.00624175	8.89132E-16	Retinal nerve fibre	RNFL_thickne	TRUE	reported
0.00746692	5.33272E-11	Retinal nerve fibre	RNFL_thickne	TRUE	reported
0.00961982	2.76006E-29	Retinal nerve fibre	RNFL_thickne	TRUE	reported
0.0187997	1.67792E-09	Retinal nerve fibre	RNFL_thickne	TRUE	reported
0.00653712	1.648E-28	Retinal nerve fibre	RNFL_thickne	TRUE	reported
0.00639872	4.68054E-10	Retinal nerve fibre	RNFL_thickne	TRUE	reported
0.00628302	2.51928E-08	Retinal nerve fibre	RNFL_thickne	TRUE	reported
0.0216905	2.20476E-08	Retinal nerve fibre	RNFL_thickne	TRUE	reported
0.00634003	1.61508E-11	Retinal nerve fibre	RNFL_thickne	TRUE	reported
0.00953742	4.39403E-12	Retinal nerve fibre	RNFL_thickne	TRUE	reported
0.00775857	9.66954E-16	Retinal nerve fibre	RNFL_thickne	TRUE	reported
0.00630867	9.44157E-13	Retinal nerve fibre	RNFL_thickne	TRUE	reported
0.00699666	2.36052E-08	Retinal nerve fibre	RNFL_thickne	TRUE	reported

0.00721573	8.66903E-10	Retinal nerve fibre	RNFL_thickne	TRUE	reported
0.00733179	3.04215E-32	Retinal nerve fibre	RNFL_thickne	TRUE	reported
0.00631565	3.96709E-08	Retinal nerve fibre	RNFL_thickne	TRUE	reported
0.00709819	2.59096E-26	Retinal nerve fibre	RNFL_thickne	TRUE	reported
0.00868264	1.54558E-10	Retinal nerve fibre	RNFL_thickne	TRUE	reported
0.00630117	2.27909E-11	Retinal nerve fibre	RNFL_thickne	TRUE	reported
0.00644678	1.86906E-08	Retinal nerve fibre	RNFL_thickne	TRUE	reported
0.00681644	3.92949E-10	Retinal nerve fibre	RNFL_thickne	TRUE	reported
0.00935494	8.92917E-09	Retinal nerve fibre	RNFL_thickne	TRUE	reported
0.00816531	2.81691E-36	Retinal nerve fibre	RNFL_thickne	TRUE	reported
0.0187249	4.35129E-08	Retinal nerve fibre	RNFL_thickne	TRUE	reported
0.00667151	8.06908E-10	Retinal nerve fibre	RNFL_thickne	TRUE	reported
0.00623047	2.85201E-08	Retinal nerve fibre	RNFL_thickne	TRUE	reported
0.00651231	1.03962E-19	Retinal nerve fibre	RNFL_thickne	TRUE	reported
0.0134661	4.56553E-10	Disc diameter afte	Disc_diamete	TRUE	reported
0.00845259	3.72548E-83	Disc diameter afte	Disc_diamete	TRUE	reported
0.00783633	9.17914E-18	Disc diameter afte	Disc_diamete	TRUE	reported
0.00895255	3.99265E-08	Disc diameter afte	Disc_diamete	TRUE	reported
0.012535	7.42765E-26	Disc diameter afte	Disc_diamete	TRUE	reported
0.0288981	2.18011E-11	Disc diameter afte	Disc_diamete	TRUE	reported
0.00667784	1.16666E-11	Disc diameter afte	Disc_diamete	TRUE	reported
0.00704051	5.64124E-09	Disc diameter afte	Disc_diamete	TRUE	reported
0.00735499	2.17711E-08	Disc diameter afte	Disc_diamete	TRUE	reported
0.0068465	2.95914E-16	Disc diameter afte	Disc_diamete	TRUE	reported
0.00862822	1.34946E-16	Disc diameter afte	Disc_diamete	TRUE	reported
0.0207869	1.49743E-11	Disc diameter afte	Disc_diamete	TRUE	reported
0.00696411	4.18258E-11	Disc diameter afte	Disc_diamete	TRUE	reported
0.0110348	3.13357E-17	Disc diameter afte	Disc_diamete	TRUE	reported
0.0122594	2.18995E-08	Disc diameter afte	Disc_diamete	TRUE	reported
0.0119379	2.46977E-10	Disc diameter afte	Disc_diamete	TRUE	reported
0.00686934	7.1876E-10	Disc diameter afte	Disc_diamete	TRUE	reported
0.00687902	7.50174E-12	Disc diameter afte	Disc_diamete	TRUE	reported
0.00725964	1.04148E-11	Disc diameter afte	Disc_diamete	TRUE	reported
0.00667804	1.29729E-11	Disc diameter afte	Disc_diamete	TRUE	reported
0.00997826	1.35242E-09	Disc diameter afte	Disc_diamete	TRUE	reported
0.00670502	1.16821E-08	Disc diameter afte	Disc_diamete	TRUE	reported
0.00777249	4.16795E-08	Disc diameter afte	Disc_diamete	TRUE	reported
0.00676038	3.48233E-10	Disc diameter afte	Disc_diamete	TRUE	reported
0.00701161	1.49454E-08	Disc diameter afte	Disc_diamete	TRUE	reported
0.0110448	2.72533E-09	Disc diameter afte	Disc_diamete	TRUE	reported
0.00748277	3.45624E-10	Disc diameter afte	Disc_diamete	TRUE	reported
0.00710965	2.39744E-09	Disc diameter afte	Disc_diamete	TRUE	reported
0.00759305	2.0051E-114	Disc diameter afte	Disc_diamete	TRUE	reported
0.00728688	8.6797E-10	Disc diameter afte	Disc_diamete	TRUE	reported

0.00670524	2.25591E-08	Disc diameter afte	Disc_diamete	TRUE	reported
0.00789497	1.35364E-12	Disc diameter afte	Disc_diamete	TRUE	reported
0.00916888	2.26374E-14	Disc diameter afte	Disc_diamete	TRUE	reported
0.00672605	5.35904E-14	Disc diameter afte	Disc_diamete	TRUE	reported
0.00846445	1.49445E-10	Disc diameter afte	Disc_diamete	TRUE	reported
0.00673998	3.95104E-09	Disc diameter afte	Disc_diamete	TRUE	reported
0.030454	7.8996E-25	Disc diameter afte	Disc_diamete	TRUE	reported
0.00671324	1.90138E-13	Disc diameter afte	Disc_diamete	TRUE	reported
0.00722967	7.18522E-18	Disc diameter afte	Disc_diamete	TRUE	reported
0.00852606	2.43787E-19	Disc diameter afte	Disc_diamete	TRUE	reported
0.00693145	7.51622E-17	Disc diameter afte	Disc_diamete	TRUE	reported
0.0074794	3.41433E-09	Disc diameter afte	Disc_diamete	TRUE	reported
0.00689582	4.54972E-20	Disc diameter afte	Disc_diamete	TRUE	reported
0.0076862	2.08942E-15	Disc diameter afte	Disc_diamete	TRUE	reported
0.00720438	6.47467E-09	Disc diameter afte	Disc_diamete	TRUE	reported
0.00698784	6.6554E-09	Thickness betwee	ELM_ISOS_th	TRUE	reported
0.00620748	2.1886E-15	Thickness betwee	ELM_ISOS_th	TRUE	reported
0.0076338	7.68336E-10	Thickness betwee	ELM_ISOS_th	TRUE	reported
0.0127299	1.04703E-13	Thickness betwee	ELM_ISOS_th	TRUE	reported
0.00657618	3.98426E-11	Thickness betwee	ELM_ISOS_th	TRUE	reported
0.00631861	4.83528E-08	Thickness betwee	ELM_ISOS_th	TRUE	reported
0.00728801	1.59307E-34	Thickness betwee	ELM_ISOS_th	TRUE	reported
0.0186303	6.81525E-09	Thickness betwee	ELM_ISOS_th	TRUE	reported
0.0071061	2.23092E-08	Thickness betwee	ELM_ISOS_th	TRUE	reported
0.00784584	3.33744E-08	Thickness betwee	ELM_ISOS_th	TRUE	reported
0.00620956	3.20107E-09	Thickness betwee	ELM_ISOS_th	TRUE	reported
0.00795319	1.48771E-09	Thickness betwee	ELM_ISOS_th	TRUE	reported
0.00624124	2.30104E-09	Thickness betwee	ELM_ISOS_th	TRUE	reported
0.00629734	6.82576E-41	Thickness betwee	ELM_ISOS_th	TRUE	reported
0.00628994	4.57034E-15	Thickness betwee	ELM_ISOS_th	TRUE	reported
0.025242	2.60625E-09	Thickness betwee	ELM_ISOS_th	TRUE	reported
0.00790606	1.81746E-09	Thickness betwee	ELM_ISOS_th	TRUE	reported
0.00630393	5.64624E-18	Thickness betwee	ELM_ISOS_th	TRUE	reported
0.00733405	8.49656E-09	Thickness betwee	ELM_ISOS_th	TRUE	reported
0.0181154	3.35366E-08	Thickness betwee	ELM_ISOS_th	TRUE	reported
0.00644079	1.44822E-09	Thickness betwee	ELM_ISOS_th	TRUE	reported
0.0062315	6.80867E-09	Thickness betwee	ELM_ISOS_th	TRUE	reported
0.00658069	9.18788E-09	Thickness betwee	ELM_ISOS_th	TRUE	reported
0.00622293	1.05787E-13	Thickness betwee	ELM_ISOS_th	TRUE	reported
0.00681075	4.00378E-34	Thickness betwee	ELM_ISOS_th	TRUE	reported
0.00817274	2.13925E-09	Thickness betwee	ELM_ISOS_th	TRUE	reported
0.0208727	1.27027E-08	Thickness betwee	ELM_ISOS_th	TRUE	reported
0.00637428	2.67521E-09	Thickness betwee	ELM_ISOS_th	TRUE	reported
0.00630604	2.01094E-16	Thickness betwee	ELM_ISOS_th	TRUE	reported

0.00629901	5.21021E-19	Thickness betwee	ELM_ISOS_th	TRUE	reported
0.00694661	2.24147E-08	Thickness betwee	ELM_ISOS_th	TRUE	reported
0.00620399	1.17207E-11	Thickness betwee	ELM_ISOS_th	TRUE	reported
0.00755844	1.81878E-11	Thickness betwee	ELM_ISOS_th	TRUE	reported
0.0127331	2.10503E-14	Thickness betwee	ELM_ISOS_th	TRUE	reported
0.021954	1.6547E-08	Thickness betwee	ELM_ISOS_th	TRUE	reported
0.00689268	2.92868E-08	Thickness betwee	ELM_ISOS_th	TRUE	reported
0.00657748	1.2711E-13	Thickness betwee	ELM_ISOS_th	TRUE	reported
0.00646319	5.60208E-09	Thickness betwee	ELM_ISOS_th	TRUE	reported
0.00732275	9.82063E-39	Thickness betwee	ELM_ISOS_th	TRUE	reported
0.0186305	1.22026E-08	Thickness betwee	ELM_ISOS_th	TRUE	reported
0.0154407	4.17738E-10	Thickness betwee	ELM_ISOS_th	TRUE	reported
0.0118727	5.12373E-09	Thickness betwee	ELM_ISOS_th	TRUE	reported
0.00748841	1.46572E-09	Thickness betwee	ELM_ISOS_th	TRUE	reported
0.00790958	2.53481E-10	Thickness betwee	ELM_ISOS_th	TRUE	reported
0.00629232	1.05193E-44	Thickness betwee	ELM_ISOS_th	TRUE	reported
0.00627702	1.29044E-16	Thickness betwee	ELM_ISOS_th	TRUE	reported
0.0221099	5.1626E-13	Thickness betwee	ELM_ISOS_th	TRUE	reported
0.0080995	7.42204E-11	Thickness betwee	ELM_ISOS_th	TRUE	reported
0.00632232	9.89298E-18	Thickness betwee	ELM_ISOS_th	TRUE	reported
0.00755568	3.57101E-09	Thickness betwee	ELM_ISOS_th	TRUE	reported
0.00732696	9.52366E-09	Thickness betwee	ELM_ISOS_th	TRUE	reported
0.00657939	1.15259E-08	Thickness betwee	ELM_ISOS_th	TRUE	reported
0.00814473	3.96809E-08	Thickness betwee	ELM_ISOS_th	TRUE	reported
0.00633705	1.04303E-08	Thickness betwee	ELM_ISOS_th	TRUE	reported
0.00655146	1.55677E-11	Thickness betwee	ELM_ISOS_th	TRUE	reported
0.00620778	7.54886E-09	Thickness betwee	ELM_ISOS_th	TRUE	reported
0.0062939	2.9256E-09	Thickness betwee	ELM_ISOS_th	TRUE	reported
0.00621831	7.79723E-11	Thickness betwee	ELM_ISOS_th	TRUE	reported
0.00779607	1.18608E-08	Thickness betwee	ELM_ISOS_th	TRUE	reported
0.00669705	4.00791E-46	Thickness betwee	ELM_ISOS_th	TRUE	reported
0.00812933	6.9692E-10	Thickness betwee	ELM_ISOS_th	TRUE	reported
0.00630058	5.47338E-20	Thickness betwee	ELM_ISOS_th	TRUE	reported
0.00627776	1.07766E-20	Thickness betwee	ELM_ISOS_th	TRUE	reported
0.00830882	4.12107E-08	Thickness betwee	ELM_ISOS_th	TRUE	reported
0.00715041	2.71841E-22	Thickness betwee	ELM_ISOS_th	TRUE	reported
0.00651791	2.00605E-11	Thickness betwee	ELM_ISOS_th	TRUE	reported
0.00709901	3.03977E-09	Thickness betwee	ELM_ISOS_th	TRUE	reported
0.0122812	1.67283E-08	Thickness betwee	ELM_ISOS_th	TRUE	reported
0.00629725	1.42243E-08	Thickness betwee	ELM_ISOS_th	TRUE	reported
0.00641971	1.92626E-09	Thickness betwee	ELM_ISOS_th	TRUE	reported
0.00658023	7.83942E-10	Thickness betwee	ELM_ISOS_th	TRUE	reported
0.00716828	1.9788E-11	Thickness betwee	ELM_ISOS_th	TRUE	reported
0.00999467	1.73304E-09	Thickness betwee	ELM_ISOS_th	TRUE	reported

0.00869558	3.28897E-10	Thickness betwee	ELM_ISOS_th	TRUE	reported
0.0286517	1.64891E-08	Thickness betwee	ELM_ISOS_th	TRUE	reported
0.00658981	7.3196E-12	Thickness betwee	ELM_ISOS_th	TRUE	reported
0.00622729	1.47429E-08	Thickness betwee	ELM_ISOS_th	TRUE	reported
0.00729223	1.15275E-35	Thickness betwee	ELM_ISOS_th	TRUE	reported
0.00622101	1.54834E-09	Thickness betwee	ELM_ISOS_th	TRUE	reported
0.00627153	8.17981E-09	Thickness betwee	ELM_ISOS_th	TRUE	reported
0.00631278	9.52828E-10	Thickness betwee	ELM_ISOS_th	TRUE	reported
0.00653666	4.44429E-08	Thickness betwee	ELM_ISOS_th	TRUE	reported
0.0118704	6.59892E-13	Thickness betwee	ELM_ISOS_th	TRUE	reported
0.00701168	6.33015E-10	Thickness betwee	ELM_ISOS_th	TRUE	reported
0.00622014	1.51618E-09	Thickness betwee	ELM_ISOS_th	TRUE	reported
0.00806158	2.83085E-09	Thickness betwee	ELM_ISOS_th	TRUE	reported
0.00665632	4.38253E-14	Thickness betwee	ELM_ISOS_th	TRUE	reported
0.00820815	3.09303E-10	Thickness betwee	ELM_ISOS_th	TRUE	reported
0.00631058	2.96167E-18	Thickness betwee	ELM_ISOS_th	TRUE	reported
0.00628406	3.93313E-10	Thickness betwee	ELM_ISOS_th	TRUE	reported
0.013506	4.22835E-09	Thickness betwee	ELM_ISOS_th	TRUE	reported
0.00712094	5.13254E-19	Thickness betwee	ELM_ISOS_th	TRUE	reported
0.00744401	1.22865E-14	Thickness betwee	ELM_ISOS_th	TRUE	reported
0.00820148	7.0874E-16	Thickness betwee	ELM_ISOS_th	TRUE	reported
0.00619795	7.71821E-19	Thickness betwee	ELM_ISOS_th	TRUE	reported
0.00739431	1.05302E-14	Thickness betwee	ELM_ISOS_th	TRUE	reported
0.00941984	1.52295E-09	Thickness betwee	ELM_ISOS_th	TRUE	reported
0.00727084	2.39591E-11	Thickness betwee	ELM_ISOS_th	TRUE	reported
0.00727458	5.53273E-16	Thickness betwee	ELM_ISOS_th	TRUE	reported
0.00633938	2.43132E-09	Thickness betwee	ELM_ISOS_th	TRUE	reported
0.00623679	5.72737E-10	Thickness betwee	ELM_ISOS_th	TRUE	reported
0.00680492	1.61666E-13	Thickness betwee	ELM_ISOS_th	TRUE	reported
0.00956159	1.08468E-24	Thickness betwee	ELM_ISOS_th	TRUE	reported
0.0144061	1.68179E-08	Thickness betwee	ELM_ISOS_th	TRUE	reported
0.00626146	1.76518E-18	Thickness betwee	ELM_ISOS_th	TRUE	reported
0.0101523	1.94154E-08	Thickness betwee	ELM_ISOS_th	TRUE	reported
0.00921928	1.28678E-08	Thickness betwee	ELM_ISOS_th	TRUE	reported
0.0062166	7.12594E-11	Thickness betwee	ELM_ISOS_th	TRUE	reported
0.00709064	1.07007E-08	Thickness betwee	ELM_ISOS_th	TRUE	reported
0.0158609	2.01881E-10	Thickness betwee	ELM_ISOS_th	TRUE	reported
0.00622611	8.11091E-26	Thickness betwee	ELM_ISOS_th	TRUE	reported
0.00681296	8.36744E-29	Thickness betwee	ELM_ISOS_th	TRUE	reported
0.00839838	1.48942E-09	Thickness betwee	ELM_ISOS_th	TRUE	reported
0.00817043	6.6431E-17	Thickness betwee	ELM_ISOS_th	TRUE	reported
0.00626888	1.30811E-09	Thickness betwee	ELM_ISOS_th	TRUE	reported
0.00627487	2.44001E-08	Thickness betwee	ELM_ISOS_th	TRUE	reported
0.00805095	3.03289E-08	Thickness betwee	ELM_ISOS_th	TRUE	reported

0.00626126	5.26503E-30	Thickness betwee	ELM_ISOS_th	TRUE	reported
0.00715575	1.017E-17	Thickness betwee	ELM_ISOS_th	TRUE	reported
0.00693448	2.87303E-20	Thickness betwee	ELM_ISOS_th	TRUE	reported
0.00712912	3.34902E-20	Thickness betwee	ELM_ISOS_th	TRUE	reported
0.00626469	4.24382E-10	Thickness betwee	ELM_ISOS_th	TRUE	reported
0.00652046	1.19676E-08	Thickness betwee	ELM_ISOS_th	TRUE	reported
0.00717058	7.3005E-09	Thickness betwee	ELM_ISOS_th	TRUE	reported
0.0122952	2.55325E-10	Thickness betwee	ELM_ISOS_th	TRUE	reported
0.00642482	1.82746E-09	Thickness betwee	ELM_ISOS_th	TRUE	reported
0.00657385	5.99403E-14	Thickness betwee	ELM_ISOS_th	TRUE	reported
0.00859161	7.09587E-15	Thickness betwee	ELM_ISOS_th	TRUE	reported
0.0095173	1.66413E-10	Thickness betwee	ELM_ISOS_th	TRUE	reported
0.00852183	4.08686E-08	Thickness betwee	ELM_ISOS_th	TRUE	reported
0.0086966	2.38344E-09	Thickness betwee	ELM_ISOS_th	TRUE	reported
0.00662717	1.95892E-10	Thickness betwee	ELM_ISOS_th	TRUE	reported
0.00762452	7.33949E-10	Thickness betwee	ELM_ISOS_th	TRUE	reported
0.00732219	7.16339E-42	Thickness betwee	ELM_ISOS_th	TRUE	reported
0.00667312	1.7834E-08	Thickness betwee	ELM_ISOS_th	TRUE	reported
0.00662255	1.32055E-09	Thickness betwee	ELM_ISOS_th	TRUE	reported
0.00653895	6.69107E-12	Thickness betwee	ELM_ISOS_th	TRUE	reported
0.0118922	5.17945E-10	Thickness betwee	ELM_ISOS_th	TRUE	reported
0.00767215	4.91585E-08	Thickness betwee	ELM_ISOS_th	TRUE	reported
0.00700403	1.02389E-13	Thickness betwee	ELM_ISOS_th	TRUE	reported
0.00622359	7.46506E-12	Thickness betwee	ELM_ISOS_th	TRUE	reported
0.00651446	1.27034E-14	Thickness betwee	ELM_ISOS_th	TRUE	reported
0.00875748	1.91968E-10	Thickness betwee	ELM_ISOS_th	TRUE	reported
0.0100057	2.39243E-11	Thickness betwee	ELM_ISOS_th	TRUE	reported
0.00631238	6.29691E-18	Thickness betwee	ELM_ISOS_th	TRUE	reported
0.00627938	7.27728E-11	Thickness betwee	ELM_ISOS_th	TRUE	reported
0.0117803	2.90155E-09	Thickness betwee	ELM_ISOS_th	TRUE	reported
0.00712475	1.07465E-22	Thickness betwee	ELM_ISOS_th	TRUE	reported
0.00729599	7.60293E-09	Thickness betwee	ELM_ISOS_th	TRUE	reported
0.00745355	1.23587E-08	Thickness betwee	ELM_ISOS_th	TRUE	reported
0.00787987	2.57803E-17	Thickness betwee	ELM_ISOS_th	TRUE	reported
0.00619892	2.58392E-17	Thickness betwee	ELM_ISOS_th	TRUE	reported
0.00756225	1.3687E-12	Thickness betwee	ELM_ISOS_th	TRUE	reported
0.0100312	1.26346E-08	Thickness betwee	ELM_ISOS_th	TRUE	reported
0.00941067	1.1356E-08	Thickness betwee	ELM_ISOS_th	TRUE	reported
0.0072758	1.76045E-09	Thickness betwee	ELM_ISOS_th	TRUE	reported
0.00727246	1.22648E-14	Thickness betwee	ELM_ISOS_th	TRUE	reported
0.00678384	1.58315E-09	Thickness betwee	ELM_ISOS_th	TRUE	reported
0.00674329	1.7768E-11	Thickness betwee	ELM_ISOS_th	TRUE	reported
0.00622131	2.66543E-08	Thickness betwee	ELM_ISOS_th	TRUE	reported
0.00680507	1.51857E-11	Thickness betwee	ELM_ISOS_th	TRUE	reported

0.00625097	3.4269E-09	Thickness betwee	ELM_ISOS_th	TRUE	reported
0.00814704	2.0962E-31	Thickness betwee	ELM_ISOS_th	TRUE	reported
0.00650472	1.50105E-15	Thickness betwee	ELM_ISOS_th	TRUE	reported
0.00623254	3.82409E-10	Thickness betwee	ELM_ISOS_th	TRUE	reported
0.00709151	2.61564E-12	Thickness betwee	ELM_ISOS_th	TRUE	reported
0.0149405	5.26179E-09	Thickness betwee	ELM_ISOS_th	TRUE	reported
0.00622281	2.25761E-21	Thickness betwee	ELM_ISOS_th	TRUE	reported
0.00670189	9.24024E-30	Thickness betwee	ELM_ISOS_th	TRUE	reported
0.0083413	1.6571E-09	Thickness betwee	ELM_ISOS_th	TRUE	reported
0.00813416	1.77534E-18	Thickness betwee	ELM_ISOS_th	TRUE	reported
0.0079252	2.10504E-09	Thickness betwee	ELM_ISOS_th	TRUE	reported
0.00805596	6.88311E-11	Thickness betwee	ELM_ISOS_th	TRUE	reported
0.00626299	8.68444E-26	Thickness betwee	ELM_ISOS_th	TRUE	reported
0.00715437	4.93622E-16	Thickness betwee	ELM_ISOS_th	TRUE	reported
0.00694623	1.13259E-17	Thickness betwee	ELM_ISOS_th	TRUE	reported
0.00739709	8.67787E-13	Thickness betwee	ELM_ISOS_th	TRUE	reported
0.00628345	2.36913E-08	Thickness betwee	ELM_ISOS_th	TRUE	reported
0.00659686	1.77019E-08	Thickness betwee	ELM_ISOS_th	TRUE	reported
0.00636335	6.69438E-11	Thickness betwee	ELM_ISOS_th	TRUE	reported
0.0082192	2.40091E-08	Thickness betwee	ELM_ISOS_th	TRUE	reported
0.0067937	5.68345E-10	Thickness betwee	ELM_ISOS_th	TRUE	reported
0.00655931	1.47902E-18	Thickness betwee	ELM_ISOS_th	TRUE	reported
0.00630801	5.98895E-09	Thickness betwee	ELM_ISOS_th	TRUE	reported
0.00816365	5.04525E-09	Thickness betwee	ELM_ISOS_th	TRUE	reported
0.00653482	3.86872E-09	Thickness betwee	ELM_ISOS_th	TRUE	reported
0.00815617	5.29688E-09	Thickness betwee	ELM_ISOS_th	TRUE	reported
0.00630877	1.02921E-08	Thickness betwee	ELM_ISOS_th	TRUE	reported
0.0126319	4.86238E-08	Thickness betwee	ELM_ISOS_th	TRUE	reported
0.00714582	3.93371E-15	Thickness betwee	ELM_ISOS_th	TRUE	reported
0.00731896	2.44525E-08	Thickness betwee	ELM_ISOS_th	TRUE	reported
0.00622251	8.86116E-09	Thickness betwee	ELM_ISOS_th	TRUE	reported
0.00707301	9.83883E-11	Thickness betwee	ELM_ISOS_th	TRUE	reported
0.00669582	1.89046E-08	Thickness betwee	ELM_ISOS_th	TRUE	reported
0.00944135	3.33016E-09	Thickness betwee	ELM_ISOS_th	TRUE	reported
0.00720877	2.31508E-10	Thickness betwee	ELM_ISOS_th	TRUE	reported
0.00790467	6.159E-10	Thickness betwee	ELM_ISOS_th	TRUE	reported
0.00733653	9.75005E-11	Thickness betwee	ELM_ISOS_th	TRUE	reported
0.00816941	7.51324E-44	Thickness betwee	ELM_ISOS_th	TRUE	reported
0.018769	2.08508E-12	Thickness betwee	ELM_ISOS_th	TRUE	reported
0.00652649	4.91025E-09	Thickness betwee	ELM_ISOS_th	TRUE	reported
0.00635238	4.81304E-08	Thickness betwee	ELM_ISOS_th	TRUE	reported
0.00711438	3.05162E-10	Thickness betwee	ELM_ISOS_th	TRUE	reported
0.00623935	3.75051E-17	Thickness betwee	ELM_ISOS_th	TRUE	reported
0.00683487	1.03293E-09	Thickness betwee	ELM_ISOS_th	TRUE	reported

0.00717624	4.42668E-10	Thickness betwee	ELM_ISOS_th	TRUE	reported
0.00689734	9.4628E-12	Thickness betwee	ELM_ISOS_th	TRUE	reported
0.00925009	1.2149E-13	Thickness betwee	ELM_ISOS_th	TRUE	reported
0.00682861	8.44321E-09	Thickness betwee	ELM_ISOS_th	TRUE	reported
0.00678438	3.16886E-08	Thickness betwee	ELM_ISOS_th	TRUE	reported
0.0196195	4.90626E-09	Thickness betwee	ELM_ISOS_th	TRUE	reported
0.00664894	1.19779E-16	Thickness betwee	ELM_ISOS_th	TRUE	reported
0.00810955	1.59246E-10	Thickness betwee	ELM_ISOS_th	TRUE	reported
0.00827617	6.66191E-09	Thickness betwee	ELM_ISOS_th	TRUE	reported
0.0106208	1.95515E-08	Thickness betwee	ELM_ISOS_th	TRUE	reported
0.00746151	9.40638E-09	Thickness betwee	ELM_ISOS_th	TRUE	reported
0.00738088	1.34367E-08	Thickness betwee	ELM_ISOS_th	TRUE	reported
0.00624618	2.72129E-10	Thickness betwee	ELM_ISOS_th	TRUE	reported
0.00761881	3.55182E-10	Thickness betwee	ELM_ISOS_th	TRUE	reported
0.00626421	1.88358E-09	Thickness betwee	ELM_ISOS_th	TRUE	reported
0.00732857	9.55877E-10	Thickness betwee	ELM_ISOS_th	TRUE	reported
0.0068616	9.25874E-11	Thickness betwee	ELM_ISOS_th	TRUE	reported
0.00821202	3.81059E-41	Thickness betwee	ELM_ISOS_th	TRUE	reported
0.00643327	4.38269E-09	Thickness betwee	ELM_ISOS_th	TRUE	reported
0.00633758	4.7597E-12	Thickness betwee	ELM_ISOS_th	TRUE	reported
0.0071518	2.25266E-10	Thickness betwee	ELM_ISOS_th	TRUE	reported
0.00626827	8.45379E-14	Thickness betwee	ELM_ISOS_th	TRUE	reported
0.00741076	4.82305E-10	Thickness betwee	ELM_ISOS_th	TRUE	reported
0.00637535	4.27715E-10	Thickness betwee	ELM_ISOS_th	TRUE	reported
0.0103891	4.66152E-08	Thickness betwee	ELM_ISOS_th	TRUE	reported
0.00745159	1.61008E-09	Thickness betwee	ELM_ISOS_th	TRUE	reported
0.00657107	6.74794E-18	Thickness betwee	ELM_ISOS_th	TRUE	reported
0.00631875	4.56427E-12	Thickness betwee	ELM_ISOS_th	TRUE	reported
0.00818526	2.30475E-09	Thickness betwee	ELM_ISOS_th	TRUE	reported
0.00817081	4.94472E-09	Thickness betwee	ELM_ISOS_th	TRUE	reported
0.00631828	1.85124E-08	Thickness betwee	ELM_ISOS_th	TRUE	reported
0.00716064	6.80083E-11	Thickness betwee	ELM_ISOS_th	TRUE	reported
0.00646094	4.73978E-08	Thickness betwee	ELM_ISOS_th	TRUE	reported
0.00724595	2.4328E-08	Thickness betwee	ELM_ISOS_th	TRUE	reported
0.00624099	1.9623E-09	Thickness betwee	ELM_ISOS_th	TRUE	reported
0.00945619	1.23502E-08	Thickness betwee	ELM_ISOS_th	TRUE	reported
0.00722573	1.58564E-09	Thickness betwee	ELM_ISOS_th	TRUE	reported
0.00791619	2.26887E-09	Thickness betwee	ELM_ISOS_th	TRUE	reported
0.00734936	1.2656E-09	Thickness betwee	ELM_ISOS_th	TRUE	reported
0.00818331	1.57069E-40	Thickness betwee	ELM_ISOS_th	TRUE	reported
0.0188119	2.88401E-11	Thickness betwee	ELM_ISOS_th	TRUE	reported
0.00713087	3.93564E-09	Thickness betwee	ELM_ISOS_th	TRUE	reported
0.0062423	2.41394E-14	Thickness betwee	ELM_ISOS_th	TRUE	reported
0.00618926	1.2917E-09	Thickness betwee	INL_ELM_thic	TRUE	reported

0.00734626	4.26664E-09	Thickness betwee	INL_ELM_thic	TRUE	reported
0.00755908	5.06516E-11	Thickness betwee	INL_ELM_thic	TRUE	reported
0.00655447	1.41092E-08	Thickness betwee	INL_ELM_thic	TRUE	reported
0.0072274	5.25841E-10	Thickness betwee	INL_ELM_thic	TRUE	reported
0.00906359	4.71306E-10	Thickness betwee	INL_ELM_thic	TRUE	reported
0.010647	3.2955E-08	Thickness betwee	INL_ELM_thic	TRUE	reported
0.0086643	4.49049E-12	Thickness betwee	INL_ELM_thic	TRUE	reported
0.0120725	5.80639E-09	Thickness betwee	INL_ELM_thic	TRUE	reported
0.00719968	3.39537E-14	Thickness betwee	INL_ELM_thic	TRUE	reported
0.00634712	1.16251E-09	Thickness betwee	INL_ELM_thic	TRUE	reported
0.00622794	1.15784E-08	Thickness betwee	INL_ELM_thic	TRUE	reported
0.0118551	1.00045E-27	Thickness betwee	INL_ELM_thic	TRUE	reported
0.00624589	1.31839E-11	Thickness betwee	INL_ELM_thic	TRUE	reported
0.0103393	1.93905E-08	Thickness betwee	INL_ELM_thic	TRUE	reported
0.00637936	8.40773E-11	Thickness betwee	INL_ELM_thic	TRUE	reported
0.00804445	9.36558E-22	Thickness betwee	INL_ELM_thic	TRUE	reported
0.0142473	2.00377E-10	Thickness betwee	INL_ELM_thic	TRUE	reported
0.00756793	1.97015E-15	Thickness betwee	INL_ELM_thic	TRUE	reported
0.00635988	7.64201E-10	Thickness betwee	INL_ELM_thic	TRUE	reported
0.00661427	3.0103E-12	Thickness betwee	INL_ELM_thic	TRUE	reported
0.0064411	1.49445E-10	Thickness betwee	INL_ELM_thic	TRUE	reported
0.00674055	1.80427E-12	Thickness betwee	INL_ELM_thic	TRUE	reported
0.00620858	8.55157E-09	Thickness betwee	INL_ELM_thic	TRUE	reported
0.00694629	2.91396E-09	Thickness betwee	INL_ELM_thic	TRUE	reported
0.00682267	8.12619E-10	Thickness betwee	INL_ELM_thic	TRUE	reported
0.00662189	1.53336E-08	Thickness betwee	INL_ELM_thic	TRUE	reported
0.00668915	1.95997E-09	Thickness betwee	INL_ELM_thic	TRUE	reported
0.00734833	3.12961E-09	Thickness betwee	INL_ELM_thic	TRUE	reported
0.00724877	1.27116E-51	Thickness betwee	INL_ELM_thic	TRUE	reported
0.00775321	6.22973E-10	Thickness betwee	INL_ELM_thic	TRUE	reported
0.00697	5.47213E-11	Thickness betwee	INL_ELM_thic	TRUE	reported
0.00622786	3.29607E-14	Thickness betwee	INL_ELM_thic	TRUE	reported
0.00710947	2.34033E-09	Thickness betwee	INL_ELM_thic	TRUE	reported
0.00975399	3.28266E-39	Thickness betwee	INL_ELM_thic	TRUE	reported
0.00620898	1.02051E-09	Thickness betwee	INL_ELM_thic	TRUE	reported
0.00623613	8.26783E-17	Thickness betwee	INL_ELM_thic	TRUE	reported
0.00638438	1.44886E-08	Thickness betwee	INL_ELM_thic	TRUE	reported
0.00744755	1.74824E-18	Thickness betwee	INL_ELM_thic	TRUE	reported
0.00647605	7.7117E-10	Thickness betwee	INL_ELM_thic	TRUE	reported
0.0113139	3.56419E-08	Thickness betwee	INL_ELM_thic	TRUE	reported
0.00692832	2.80419E-13	Thickness betwee	INL_ELM_thic	TRUE	reported
0.00673951	1.15052E-08	Thickness betwee	INL_ELM_thic	TRUE	reported
0.00760094	1.40772E-09	Thickness betwee	INL_ELM_thic	TRUE	reported
0.00638677	1.20837E-13	Thickness betwee	INL_ELM_thic	TRUE	reported

0.00752174	2.52621E-11	Thickness betwee	INL_ELM_thic	TRUE	reported
0.00626429	9.38668E-10	Thickness betwee	INL_ELM_thic	TRUE	reported
0.00663962	2.23495E-09	Thickness betwee	INL_ELM_thic	TRUE	reported
0.00721047	1.69788E-08	Thickness betwee	INL_ELM_thic	TRUE	reported
0.00906067	1.6848E-08	Thickness betwee	INL_ELM_thic	TRUE	reported
0.00618764	4.27842E-10	Thickness betwee	INL_ELM_thic	TRUE	reported
0.00864938	1.63627E-15	Thickness betwee	INL_ELM_thic	TRUE	reported
0.00716948	3.73458E-14	Thickness betwee	INL_ELM_thic	TRUE	reported
0.0142084	2.72223E-08	Thickness betwee	INL_ELM_thic	TRUE	reported
0.00633477	3.25008E-10	Thickness betwee	INL_ELM_thic	TRUE	reported
0.00621802	1.64148E-10	Thickness betwee	INL_ELM_thic	TRUE	reported
0.0119216	2.97654E-28	Thickness betwee	INL_ELM_thic	TRUE	reported
0.00637418	1.04895E-13	Thickness betwee	INL_ELM_thic	TRUE	reported
0.00622694	2.82539E-10	Thickness betwee	INL_ELM_thic	TRUE	reported
0.00636077	3.08862E-12	Thickness betwee	INL_ELM_thic	TRUE	reported
0.00802668	2.79885E-24	Thickness betwee	INL_ELM_thic	TRUE	reported
0.00874908	3.56553E-13	Thickness betwee	INL_ELM_thic	TRUE	reported
0.00645907	1.04383E-12	Thickness betwee	INL_ELM_thic	TRUE	reported
0.00660011	8.68116E-14	Thickness betwee	INL_ELM_thic	TRUE	reported
0.00811758	1.78927E-09	Thickness betwee	INL_ELM_thic	TRUE	reported
0.00676145	2.74268E-12	Thickness betwee	INL_ELM_thic	TRUE	reported
0.00622059	2.49532E-08	Thickness betwee	INL_ELM_thic	TRUE	reported
0.00694366	6.40248E-11	Thickness betwee	INL_ELM_thic	TRUE	reported
0.00658519	3.04959E-08	Thickness betwee	INL_ELM_thic	TRUE	reported
0.00680829	1.14156E-10	Thickness betwee	INL_ELM_thic	TRUE	reported
0.00667365	5.32191E-11	Thickness betwee	INL_ELM_thic	TRUE	reported
0.00723131	9.43867E-60	Thickness betwee	INL_ELM_thic	TRUE	reported
0.00796521	2.16912E-09	Thickness betwee	INL_ELM_thic	TRUE	reported
0.0063154	3.30969E-10	Thickness betwee	INL_ELM_thic	TRUE	reported
0.00620613	2.8183E-15	Thickness betwee	INL_ELM_thic	TRUE	reported
0.0158662	3.50729E-08	Thickness betwee	INL_ELM_thic	TRUE	reported
0.00656869	9.26202E-12	Thickness betwee	INL_ELM_thic	TRUE	reported
0.00810484	1.03729E-54	Thickness betwee	INL_ELM_thic	TRUE	reported
0.00634395	8.12218E-14	Thickness betwee	INL_ELM_thic	TRUE	reported
0.00652889	3.46028E-09	Thickness betwee	INL_ELM_thic	TRUE	reported
0.00623273	9.07688E-18	Thickness betwee	INL_ELM_thic	TRUE	reported
0.00630632	3.45898E-08	Thickness betwee	INL_ELM_thic	TRUE	reported
0.00740166	5.36511E-24	Thickness betwee	INL_ELM_thic	TRUE	reported
0.00691293	2.36893E-14	Thickness betwee	INL_ELM_thic	TRUE	reported
0.00624747	5.40312E-19	Thickness betwee	INL_ELM_thic	TRUE	reported
0.00641687	6.76408E-10	Thickness betwee	INL_ELM_thic	TRUE	reported
0.00750873	1.99631E-09	Thickness betwee	INL_ELM_thic	TRUE	reported
0.00716567	1.13493E-08	Thickness betwee	INL_ELM_thic	TRUE	reported
0.0064604	5.8167E-30	Thickness betwee	INL_ELM_thic	TRUE	reported

0.00806173	1.44527E-10	Thickness betwee	INL_ELM_thic	TRUE	reported
0.00661843	1.542E-16	Thickness betwee	INL_ELM_thic	TRUE	reported
0.0244625	1.91198E-21	Thickness betwee	INL_ELM_thic	TRUE	reported
0.00866153	2.62393E-15	Thickness betwee	INL_ELM_thic	TRUE	reported
0.00729297	7.52281E-13	Thickness betwee	INL_ELM_thic	TRUE	reported
0.00916282	6.0819E-13	Thickness betwee	INL_ELM_thic	TRUE	reported
0.00768985	3.58569E-08	Thickness betwee	INL_ELM_thic	TRUE	reported
0.0174321	2.6527E-15	Thickness betwee	INL_ELM_thic	TRUE	reported
0.00769237	2.74533E-12	Thickness betwee	INL_ELM_thic	TRUE	reported
0.0087482	8.59108E-23	Thickness betwee	INL_ELM_thic	TRUE	reported
0.00633296	1.28336E-08	Thickness betwee	INL_ELM_thic	TRUE	reported
0.0121888	8.46043E-11	Thickness betwee	INL_ELM_thic	TRUE	reported
0.00887552	9.46766E-09	Thickness betwee	INL_ELM_thic	TRUE	reported
0.00692051	2.62345E-09	Thickness betwee	INL_ELM_thic	TRUE	reported
0.00640723	7.07105E-18	Thickness betwee	INL_ELM_thic	TRUE	reported
0.0181015	4.51815E-08	Thickness betwee	INL_ELM_thic	TRUE	reported
0.00630167	3.81818E-08	Thickness betwee	INL_ELM_thic	TRUE	reported
0.0119312	4.26315E-30	Thickness betwee	INL_ELM_thic	TRUE	reported
0.00630242	3.35737E-11	Thickness betwee	INL_ELM_thic	TRUE	reported
0.00643781	9.38378E-10	Thickness betwee	INL_ELM_thic	TRUE	reported
0.00811927	5.95931E-13	Thickness betwee	INL_ELM_thic	TRUE	reported
0.0143668	1.67611E-09	Thickness betwee	INL_ELM_thic	TRUE	reported
0.00763671	9.81114E-22	Thickness betwee	INL_ELM_thic	TRUE	reported
0.0099895	7.06723E-10	Thickness betwee	INL_ELM_thic	TRUE	reported
0.0171051	1.81192E-20	Thickness betwee	INL_ELM_thic	TRUE	reported
0.00646653	8.7864E-10	Thickness betwee	INL_ELM_thic	TRUE	reported
0.00669943	3.21821E-10	Thickness betwee	INL_ELM_thic	TRUE	reported
0.0082277	1.58593E-11	Thickness betwee	INL_ELM_thic	TRUE	reported
0.00649825	6.31167E-10	Thickness betwee	INL_ELM_thic	TRUE	reported
0.00677073	8.84715E-09	Thickness betwee	INL_ELM_thic	TRUE	reported
0.00702446	2.45311E-10	Thickness betwee	INL_ELM_thic	TRUE	reported
0.0063483	3.49377E-11	Thickness betwee	INL_ELM_thic	TRUE	reported
0.00790617	6.21839E-10	Thickness betwee	INL_ELM_thic	TRUE	reported
0.00913013	1.23629E-08	Thickness betwee	INL_ELM_thic	TRUE	reported
0.00912385	1.15247E-08	Thickness betwee	INL_ELM_thic	TRUE	reported
0.00642482	2.12402E-08	Thickness betwee	INL_ELM_thic	TRUE	reported
0.00719651	1.95972E-33	Thickness betwee	INL_ELM_thic	TRUE	reported
0.00680534	2.09997E-21	Thickness betwee	INL_ELM_thic	TRUE	reported
0.00627707	7.63513E-13	Thickness betwee	INL_ELM_thic	TRUE	reported
0.00655965	1.50037E-11	Thickness betwee	INL_ELM_thic	TRUE	reported
0.00735097	4.33303E-09	Thickness betwee	INL_ELM_thic	TRUE	reported
0.00769597	3.64582E-08	Thickness betwee	INL_ELM_thic	TRUE	reported
0.00688818	1.937E-18	Thickness betwee	INL_ELM_thic	TRUE	reported
0.00635107	1.14169E-09	Thickness betwee	INL_ELM_thic	TRUE	reported

0.00635144	2.12588E-11	Thickness betwee	INL_ELM_thic	TRUE	reported
0.00668137	3.24551E-11	Thickness betwee	INL_ELM_thic	TRUE	reported
0.00731716	1.186E-137	Thickness betwee	INL_ELM_thic	TRUE	reported
0.0082866	2.8676E-15	Thickness betwee	INL_ELM_thic	TRUE	reported
0.00627008	3.21683E-10	Thickness betwee	INL_ELM_thic	TRUE	reported
0.00690299	3.15469E-10	Thickness betwee	INL_ELM_thic	TRUE	reported
0.00648806	5.73891E-10	Thickness betwee	INL_ELM_thic	TRUE	reported
0.00627906	1.0341E-15	Thickness betwee	INL_ELM_thic	TRUE	reported
0.00700497	7.91317E-09	Thickness betwee	INL_ELM_thic	TRUE	reported
0.00684891	1.63393E-11	Thickness betwee	INL_ELM_thic	TRUE	reported
0.0160701	3.70455E-15	Thickness betwee	INL_ELM_thic	TRUE	reported
0.00819782	1.1143E-190	Thickness betwee	INL_ELM_thic	TRUE	reported
0.0187998	1.34892E-36	Thickness betwee	INL_ELM_thic	TRUE	reported
0.00628046	2.42729E-23	Thickness betwee	INL_ELM_thic	TRUE	reported
0.00684538	1.21032E-08	Thickness betwee	INL_ELM_thic	TRUE	reported
0.00624677	1.42666E-09	Thickness betwee	INL_ELM_thic	TRUE	reported
0.0065177	1.95184E-15	Thickness betwee	INL_ELM_thic	TRUE	reported
0.00644376	7.82124E-09	Thickness betwee	INL_ELM_thic	TRUE	reported
0.00634846	4.7886E-16	Thickness betwee	INL_ELM_thic	TRUE	reported
0.0182378	1.689E-10	Thickness betwee	INL_ELM_thic	TRUE	reported
0.00697038	2.39253E-10	Thickness betwee	INL_ELM_thic	TRUE	reported
0.0075121	1.6883E-27	Thickness betwee	INL_ELM_thic	TRUE	reported
0.00883141	3.24486E-19	Thickness betwee	INL_ELM_thic	TRUE	reported
0.00653194	4.34807E-21	Thickness betwee	INL_ELM_thic	TRUE	reported
0.00681463	2.57535E-19	Thickness betwee	INL_ELM_thic	TRUE	reported
0.0093555	2.7653E-18	Thickness betwee	INL_ELM_thic	TRUE	reported
0.00624417	8.92166E-11	Thickness betwee	INL_ELM_thic	TRUE	reported
0.00691589	4.55365E-30	Thickness betwee	INL_ELM_thic	TRUE	reported
0.00675837	1.20806E-08	Thickness betwee	INL_ELM_thic	TRUE	reported
0.00622015	2.20289E-18	Thickness betwee	INL_ELM_thic	TRUE	reported
0.00643519	2.97186E-36	Thickness betwee	INL_ELM_thic	TRUE	reported
0.00802203	1.85039E-14	Thickness betwee	INL_ELM_thic	TRUE	reported
0.00659012	5.60389E-19	Thickness betwee	INL_ELM_thic	TRUE	reported
0.00635729	6.47268E-10	Thickness betwee	INL_ELM_thic	TRUE	reported
0.00675215	2.11009E-08	Thickness betwee	INL_ELM_thic	TRUE	reported
0.0243163	6.39017E-29	Thickness betwee	INL_ELM_thic	TRUE	reported
0.00862818	1.5903E-13	Thickness betwee	INL_ELM_thic	TRUE	reported
0.00726505	3.06313E-08	Thickness betwee	INL_ELM_thic	TRUE	reported
0.00913088	1.33024E-08	Thickness betwee	INL_ELM_thic	TRUE	reported
0.0173516	3.04195E-17	Thickness betwee	INL_ELM_thic	TRUE	reported
0.0119714	4.15582E-08	Thickness betwee	INL_ELM_thic	TRUE	reported
0.00765587	6.9921E-15	Thickness betwee	INL_ELM_thic	TRUE	reported
0.00871467	8.79567E-22	Thickness betwee	INL_ELM_thic	TRUE	reported
0.0121199	2.7347E-08	Thickness betwee	INL_ELM_thic	TRUE	reported

0.014296	3.1188E-09	Thickness betwee	INL_ELM_thic	TRUE	reported
0.00689223	2.59931E-09	Thickness betwee	INL_ELM_thic	TRUE	reported
0.00638244	4.34563E-14	Thickness betwee	INL_ELM_thic	TRUE	reported
0.00627275	7.36318E-11	Thickness betwee	INL_ELM_thic	TRUE	reported
0.0120241	2.34794E-29	Thickness betwee	INL_ELM_thic	TRUE	reported
0.0062227	1.59248E-13	Thickness betwee	INL_ELM_thic	TRUE	reported
0.00640986	1.03791E-12	Thickness betwee	INL_ELM_thic	TRUE	reported
0.00808635	2.9892E-14	Thickness betwee	INL_ELM_thic	TRUE	reported
0.0143192	2.46845E-10	Thickness betwee	INL_ELM_thic	TRUE	reported
0.00761091	9.21226E-21	Thickness betwee	INL_ELM_thic	TRUE	reported
0.0170116	9.96688E-17	Thickness betwee	INL_ELM_thic	TRUE	reported
0.00643967	1.42501E-12	Thickness betwee	INL_ELM_thic	TRUE	reported
0.0067034	1.86018E-10	Thickness betwee	INL_ELM_thic	TRUE	reported
0.00824384	2.78326E-08	Thickness betwee	INL_ELM_thic	TRUE	reported
0.00669589	1.5508E-08	Thickness betwee	INL_ELM_thic	TRUE	reported
0.00700032	6.40436E-11	Thickness betwee	INL_ELM_thic	TRUE	reported
0.00629348	1.38897E-12	Thickness betwee	INL_ELM_thic	TRUE	reported
0.00764687	7.23002E-10	Thickness betwee	INL_ELM_thic	TRUE	reported
0.0090864	7.459E-09	Thickness betwee	INL_ELM_thic	TRUE	reported
0.00683232	1.65293E-09	Thickness betwee	INL_ELM_thic	TRUE	reported
0.00716807	1.55535E-27	Thickness betwee	INL_ELM_thic	TRUE	reported
0.0068081	8.65995E-22	Thickness betwee	INL_ELM_thic	TRUE	reported
0.00643915	1.03583E-13	Thickness betwee	INL_ELM_thic	TRUE	reported
0.00635306	3.74606E-09	Thickness betwee	INL_ELM_thic	TRUE	reported
0.00654305	2.56391E-13	Thickness betwee	INL_ELM_thic	TRUE	reported
0.00735096	2.69518E-11	Thickness betwee	INL_ELM_thic	TRUE	reported
0.00686049	2.17416E-17	Thickness betwee	INL_ELM_thic	TRUE	reported
0.01536	1.93289E-18	Thickness betwee	INL_ELM_thic	TRUE	reported
0.00808804	3.90166E-11	Thickness betwee	INL_ELM_thic	TRUE	reported
0.00674009	4.53114E-10	Thickness betwee	INL_ELM_thic	TRUE	reported
0.00728578	1.3491E-130	Thickness betwee	INL_ELM_thic	TRUE	reported
0.0075983	1.25568E-12	Thickness betwee	INL_ELM_thic	TRUE	reported
0.00624509	3.58295E-09	Thickness betwee	INL_ELM_thic	TRUE	reported
0.00679918	2.06811E-08	Thickness betwee	INL_ELM_thic	TRUE	reported
0.008658	2.2636E-08	Thickness betwee	INL_ELM_thic	TRUE	reported
0.00625719	2.01865E-16	Thickness betwee	INL_ELM_thic	TRUE	reported
0.00951189	6.37228E-09	Thickness betwee	INL_ELM_thic	TRUE	reported
0.00682222	1.13984E-10	Thickness betwee	INL_ELM_thic	TRUE	reported
0.00661759	3.93117E-22	Thickness betwee	INL_ELM_thic	TRUE	reported
0.0156161	4.05822E-14	Thickness betwee	INL_ELM_thic	TRUE	reported
0.00816564	8.1716E-222	Thickness betwee	INL_ELM_thic	TRUE	reported
0.00639242	1.22191E-33	Thickness betwee	INL_ELM_thic	TRUE	reported
0.00621656	1.49108E-08	Thickness betwee	INL_ELM_thic	TRUE	reported
0.00622299	1.2437E-09	Thickness betwee	INL_ELM_thic	TRUE	reported

0.0064361	8.71361E-19	Thickness betwee	INL_ELM_thic	TRUE	reported
0.00677848	2.40644E-08	Thickness betwee	INL_ELM_thic	TRUE	reported
0.00627604	1.35637E-08	Thickness betwee	INL_ELM_thic	TRUE	reported
0.00632216	6.5612E-16	Thickness betwee	INL_ELM_thic	TRUE	reported
0.00688325	9.20015E-11	Thickness betwee	INL_ELM_thic	TRUE	reported
0.00751705	9.27081E-33	Thickness betwee	INL_ELM_thic	TRUE	reported
0.00879466	1.2776E-18	Thickness betwee	INL_ELM_thic	TRUE	reported
0.0075209	1.25395E-09	Thickness betwee	INL_ELM_thic	TRUE	reported
0.0077555	6.48968E-16	Thickness betwee	INL_ELM_thic	TRUE	reported
0.00818656	1.91008E-08	Thickness betwee	INL_ELM_thic	TRUE	reported
0.0135562	1.55486E-14	Thickness betwee	INL_ELM_thic	TRUE	reported
0.00749698	4.72836E-21	Thickness betwee	INL_ELM_thic	TRUE	reported
0.0209611	7.18011E-09	Thickness betwee	INL_ELM_thic	TRUE	reported
0.0062167	7.1948E-12	Thickness betwee	INL_ELM_thic	TRUE	reported
0.00624274	8.9089E-09	Thickness betwee	INL_ELM_thic	TRUE	reported
0.00689795	2.32495E-31	Thickness betwee	INL_ELM_thic	TRUE	reported
0.00672945	3.09003E-10	Thickness betwee	INL_ELM_thic	TRUE	reported
0.00639329	2.07592E-08	Thickness betwee	INL_ELM_thic	TRUE	reported
0.00724371	1.2015E-09	Thickness betwee	INL_ELM_thic	TRUE	reported
0.00650728	1.34004E-18	Thickness betwee	INL_ELM_thic	TRUE	reported
0.00667042	2.07782E-13	Thickness betwee	INL_ELM_thic	TRUE	reported
0.00632923	2.49038E-10	Thickness betwee	INL_ELM_thic	TRUE	reported
0.0246387	1.01655E-31	Thickness betwee	INL_ELM_thic	TRUE	reported
0.00832666	1.41121E-29	Thickness betwee	INL_ELM_thic	TRUE	reported
0.0175427	1.24736E-24	Thickness betwee	INL_ELM_thic	TRUE	reported
0.0124394	2.12205E-20	Thickness betwee	INL_ELM_thic	TRUE	reported
0.00630576	3.95277E-11	Thickness betwee	INL_ELM_thic	TRUE	reported
0.008661	1.78429E-08	Thickness betwee	INL_ELM_thic	TRUE	reported
0.00697666	4.42753E-15	Thickness betwee	INL_ELM_thic	TRUE	reported
0.00736874	4.92577E-12	Thickness betwee	INL_ELM_thic	TRUE	reported
0.00635207	1.06874E-08	Thickness betwee	INL_ELM_thic	TRUE	reported
0.00950355	2.85505E-08	Thickness betwee	INL_ELM_thic	TRUE	reported
0.00697297	8.93764E-11	Thickness betwee	INL_ELM_thic	TRUE	reported
0.00703628	3.3308E-09	Thickness betwee	INL_ELM_thic	TRUE	reported
0.00645549	4.39454E-08	Thickness betwee	INL_ELM_thic	TRUE	reported
0.00633875	1.19055E-08	Thickness betwee	INL_ELM_thic	TRUE	reported
0.00665146	8.18444E-18	Thickness betwee	INL_ELM_thic	TRUE	reported
0.00662609	2.63863E-10	Thickness betwee	INL_ELM_thic	TRUE	reported
0.0120328	4.84347E-50	Thickness betwee	INL_ELM_thic	TRUE	reported
0.00645905	4.71652E-12	Thickness betwee	INL_ELM_thic	TRUE	reported
0.00785227	3.11815E-23	Thickness betwee	INL_ELM_thic	TRUE	reported
0.00649733	4.10273E-10	Thickness betwee	INL_ELM_thic	TRUE	reported
0.0105467	1.22895E-08	Thickness betwee	INL_ELM_thic	TRUE	reported
0.00769717	1.06339E-09	Thickness betwee	INL_ELM_thic	TRUE	reported

0.00631101	9.59808E-12	Thickness betwee	INL_ELM_thic	TRUE	reported
0.0172517	3.51042E-26	Thickness betwee	INL_ELM_thic	TRUE	reported
0.0106926	5.56809E-14	Thickness betwee	INL_ELM_thic	TRUE	reported
0.00829514	6.50518E-17	Thickness betwee	INL_ELM_thic	TRUE	reported
0.00971643	2.16746E-13	Thickness betwee	INL_ELM_thic	TRUE	reported
0.00707727	4.68119E-16	Thickness betwee	INL_ELM_thic	TRUE	reported
0.00941772	5.08799E-11	Thickness betwee	INL_ELM_thic	TRUE	reported
0.0210519	1.71127E-13	Thickness betwee	INL_ELM_thic	TRUE	reported
0.00919032	1.52573E-16	Thickness betwee	INL_ELM_thic	TRUE	reported
0.00690491	2.17762E-08	Thickness betwee	INL_ELM_thic	TRUE	reported
0.00724574	3.66814E-78	Thickness betwee	INL_ELM_thic	TRUE	reported
0.00845661	1.08632E-08	Thickness betwee	INL_ELM_thic	TRUE	reported
0.00689242	7.7437E-19	Thickness betwee	INL_ELM_thic	TRUE	reported
0.00651566	2.38408E-11	Thickness betwee	INL_ELM_thic	TRUE	reported
0.00662624	2.62313E-11	Thickness betwee	INL_ELM_thic	TRUE	reported
0.00743742	2.01435E-24	Thickness betwee	INL_ELM_thic	TRUE	reported
0.00670896	2.27409E-11	Thickness betwee	INL_ELM_thic	TRUE	reported
0.00686695	1.03566E-13	Thickness betwee	INL_ELM_thic	TRUE	reported
0.0155616	2.29262E-17	Thickness betwee	INL_ELM_thic	TRUE	reported
0.00817725	3.08901E-13	Thickness betwee	INL_ELM_thic	TRUE	reported
0.00631588	7.76517E-09	Thickness betwee	INL_ELM_thic	TRUE	reported
0.00683702	3.816E-11	Thickness betwee	INL_ELM_thic	TRUE	reported
0.00756587	2.65953E-08	Thickness betwee	INL_ELM_thic	TRUE	reported
0.00737165	2.5042E-111	Thickness betwee	INL_ELM_thic	TRUE	reported
0.00797059	1.86477E-18	Thickness betwee	INL_ELM_thic	TRUE	reported
0.00631902	2.98998E-09	Thickness betwee	INL_ELM_thic	TRUE	reported
0.00686172	3.78342E-11	Thickness betwee	INL_ELM_thic	TRUE	reported
0.00857973	3.45909E-11	Thickness betwee	INL_ELM_thic	TRUE	reported
0.00638397	7.4231E-12	Thickness betwee	INL_ELM_thic	TRUE	reported
0.0162117	5.68561E-15	Thickness betwee	INL_ELM_thic	TRUE	reported
0.00826086	6.5655E-273	Thickness betwee	INL_ELM_thic	TRUE	reported
0.0189469	4.42004E-53	Thickness betwee	INL_ELM_thic	TRUE	reported
0.00632641	6.76414E-17	Thickness betwee	INL_ELM_thic	TRUE	reported
0.00629131	5.95169E-10	Thickness betwee	INL_ELM_thic	TRUE	reported
0.00689978	2.30361E-10	Thickness betwee	INL_ELM_thic	TRUE	reported
0.00628365	2.15664E-14	Thickness betwee	INL_ELM_thic	TRUE	reported
0.00657059	1.96364E-08	Thickness betwee	INL_ELM_thic	TRUE	reported
0.00770304	6.29155E-11	Thickness betwee	INL_ELM_thic	TRUE	reported
0.00685595	1.45965E-08	Thickness betwee	INL_ELM_thic	TRUE	reported
0.0146657	1.53342E-08	Thickness betwee	INL_ELM_thic	TRUE	reported
0.00728055	3.57901E-16	Thickness betwee	INL_ELM_thic	TRUE	reported
0.0183573	8.60414E-11	Thickness betwee	INL_ELM_thic	TRUE	reported
0.00701903	1.73876E-08	Thickness betwee	INL_ELM_thic	TRUE	reported
0.00760101	4.50034E-23	Thickness betwee	INL_ELM_thic	TRUE	reported

0.00890179	1.43739E-10	Thickness betwee	INL_ELM_thic	TRUE	reported
0.00658257	1.63614E-26	Thickness betwee	INL_ELM_thic	TRUE	reported
0.00685173	3.72358E-24	Thickness betwee	INL_ELM_thic	TRUE	reported
0.00942402	2.27213E-13	Thickness betwee	INL_ELM_thic	TRUE	reported
0.0211681	1.05499E-12	Thickness betwee	INL_ELM_thic	TRUE	reported
0.00863409	1.88364E-09	Thickness betwee	INL_ELM_thic	TRUE	reported
0.00629211	2.71196E-16	Thickness betwee	INL_ELM_thic	TRUE	reported
0.0063581	1.57162E-10	Thickness betwee	INL_ELM_thic	TRUE	reported
0.00846347	2.07154E-08	Thickness betwee	INL_ELM_thic	TRUE	reported
0.00695246	1.89795E-20	Thickness betwee	INL_ELM_thic	TRUE	reported
0.00680974	2.04746E-08	Thickness betwee	INL_ELM_thic	TRUE	reported
0.00628637	7.62345E-09	Thickness betwee	INL_ELM_thic	TRUE	reported
0.00647698	5.05277E-19	Thickness betwee	INL_ELM_thic	TRUE	reported
0.00634734	1.89799E-08	Thickness betwee	INL_ELM_thic	TRUE	reported
0.0066441	3.24586E-09	Thickness betwee	INL_ELM_thic	TRUE	reported
0.00663192	2.64433E-15	Thickness betwee	INL_ELM_thic	TRUE	reported
0.00640349	1.04105E-11	Thickness betwee	INL_ELM_thic	TRUE	reported
0.00654368	2.25111E-08	Thickness betwee	INL_ELM_thic	TRUE	reported
0.024497	6.60801E-37	Thickness betwee	INL_ELM_thic	TRUE	reported
0.00868617	1.47526E-25	Thickness betwee	INL_ELM_thic	TRUE	reported
0.00918538	3.28405E-08	Thickness betwee	INL_ELM_thic	TRUE	reported
0.0174669	1.29349E-25	Thickness betwee	INL_ELM_thic	TRUE	reported
0.0124143	2.82652E-19	Thickness betwee	INL_ELM_thic	TRUE	reported
0.00627386	2.39323E-09	Thickness betwee	INL_ELM_thic	TRUE	reported
0.00692835	1.05256E-15	Thickness betwee	INL_ELM_thic	TRUE	reported
0.00733877	6.16912E-11	Thickness betwee	INL_ELM_thic	TRUE	reported
0.00945894	6.59136E-09	Thickness betwee	INL_ELM_thic	TRUE	reported
0.00693644	2.60276E-11	Thickness betwee	INL_ELM_thic	TRUE	reported
0.00636922	1.66519E-09	Thickness betwee	INL_ELM_thic	TRUE	reported
0.00630899	1.44346E-09	Thickness betwee	INL_ELM_thic	TRUE	reported
0.00661518	2.43941E-16	Thickness betwee	INL_ELM_thic	TRUE	reported
0.00659354	1.3045E-11	Thickness betwee	INL_ELM_thic	TRUE	reported
0.0120963	2.72417E-49	Thickness betwee	INL_ELM_thic	TRUE	reported
0.00643217	1.75069E-10	Thickness betwee	INL_ELM_thic	TRUE	reported
0.0078138	4.00718E-20	Thickness betwee	INL_ELM_thic	TRUE	reported
0.00765806	9.65306E-11	Thickness betwee	INL_ELM_thic	TRUE	reported
0.00828562	1.44851E-10	Thickness betwee	INL_ELM_thic	TRUE	reported
0.00628033	5.17553E-10	Thickness betwee	INL_ELM_thic	TRUE	reported
0.0171276	7.06605E-24	Thickness betwee	INL_ELM_thic	TRUE	reported
0.0106321	3.58397E-14	Thickness betwee	INL_ELM_thic	TRUE	reported
0.00829724	1.04208E-15	Thickness betwee	INL_ELM_thic	TRUE	reported
0.00966487	2.26658E-13	Thickness betwee	INL_ELM_thic	TRUE	reported
0.00678642	3.25026E-08	Thickness betwee	INL_ELM_thic	TRUE	reported
0.0078128	3.99907E-20	Thickness betwee	INL_ELM_thic	TRUE	reported

0.00633518	1.8435E-11	Thickness betwee	INL_ELM_thic	TRUE	reported
0.00737089	2.42495E-08	Thickness betwee	INL_ELM_thic	TRUE	reported
0.0209867	1.75766E-11	Thickness betwee	INL_ELM_thic	TRUE	reported
0.00905394	3.90467E-14	Thickness betwee	INL_ELM_thic	TRUE	reported
0.0068765	2.54344E-09	Thickness betwee	INL_ELM_thic	TRUE	reported
0.00721622	2.43836E-77	Thickness betwee	INL_ELM_thic	TRUE	reported
0.00685208	3.68213E-20	Thickness betwee	INL_ELM_thic	TRUE	reported
0.00648676	1.6927E-12	Thickness betwee	INL_ELM_thic	TRUE	reported
0.00658391	2.70837E-10	Thickness betwee	INL_ELM_thic	TRUE	reported
0.00739812	6.25894E-24	Thickness betwee	INL_ELM_thic	TRUE	reported
0.00667898	2.46607E-11	Thickness betwee	INL_ELM_thic	TRUE	reported
0.00695633	1.00436E-14	Thickness betwee	INL_ELM_thic	TRUE	reported
0.0154544	2.00889E-23	Thickness betwee	INL_ELM_thic	TRUE	reported
0.00813767	5.37118E-15	Thickness betwee	INL_ELM_thic	TRUE	reported
0.00628295	3.71579E-11	Thickness betwee	INL_ELM_thic	TRUE	reported
0.00680375	1.1484E-09	Thickness betwee	INL_ELM_thic	TRUE	reported
0.00639758	4.62108E-10	Thickness betwee	INL_ELM_thic	TRUE	reported
0.00733469	2.1796E-99	Thickness betwee	INL_ELM_thic	TRUE	reported
0.00765236	4.03803E-18	Thickness betwee	INL_ELM_thic	TRUE	reported
0.00684732	1.11269E-10	Thickness betwee	INL_ELM_thic	TRUE	reported
0.00857556	8.18104E-10	Thickness betwee	INL_ELM_thic	TRUE	reported
0.0063896	1.77463E-09	Thickness betwee	INL_ELM_thic	TRUE	reported
0.00863896	3.17543E-08	Thickness betwee	INL_ELM_thic	TRUE	reported
0.0178801	1.788E-14	Thickness betwee	INL_ELM_thic	TRUE	reported
0.00821447	1.9975E-277	Thickness betwee	INL_ELM_thic	TRUE	reported
0.0188415	1.62773E-46	Thickness betwee	INL_ELM_thic	TRUE	reported
0.00629657	4.17043E-18	Thickness betwee	INL_ELM_thic	TRUE	reported
0.00688137	2.81557E-10	Thickness betwee	INL_ELM_thic	TRUE	reported
0.0062636	2.84934E-14	Thickness betwee	INL_ELM_thic	TRUE	reported
0.00653207	9.57352E-10	Thickness betwee	INL_ELM_thic	TRUE	reported
0.0109559	2.32621E-08	Thickness betwee	INL_ELM_thic	TRUE	reported
0.00681952	4.70432E-12	Thickness betwee	INL_ELM_thic	TRUE	reported
0.0150868	8.82075E-09	Thickness betwee	INL_ELM_thic	TRUE	reported
0.00627351	7.73002E-18	Thickness betwee	INL_ELM_thic	TRUE	reported
0.00696799	2.40502E-11	Thickness betwee	INL_ELM_thic	TRUE	reported
0.00699816	6.12603E-09	Thickness betwee	INL_ELM_thic	TRUE	reported
0.00756862	4.93776E-19	Thickness betwee	INL_ELM_thic	TRUE	reported
0.00790617	1.83446E-08	Thickness betwee	INL_ELM_thic	TRUE	reported
0.00683354	2.20528E-22	Thickness betwee	INL_ELM_thic	TRUE	reported
0.00820286	6.15964E-09	Thickness betwee	INL_ELM_thic	TRUE	reported
0.00699195	1.29948E-23	Thickness betwee	INL_ELM_thic	TRUE	reported
0.00937246	4.49778E-11	Thickness betwee	INL_ELM_thic	TRUE	reported
0.00630838	3.70031E-08	Thickness betwee	INL_ELM_thic	TRUE	reported
0.0210607	2.57929E-13	Thickness betwee	INL_ELM_thic	TRUE	reported

0.00625702	4.87988E-14	Thickness betwee	INL_ELM_thic	TRUE	reported
0.00635211	6.31761E-11	Thickness betwee	INL_ELM_thic	TRUE	reported
0.00693879	2.12644E-21	Thickness betwee	INL_ELM_thic	TRUE	reported
0.00618961	2.94848E-10	Thickness betwee	INL_RPE_thic	TRUE	reported
0.00740957	2.65209E-14	Thickness betwee	INL_RPE_thic	TRUE	reported
0.00623385	1.11111E-12	Thickness betwee	INL_RPE_thic	TRUE	reported
0.00624147	2.54959E-08	Thickness betwee	INL_RPE_thic	TRUE	reported
0.00722873	1.1634E-08	Thickness betwee	INL_RPE_thic	TRUE	reported
0.00720204	1.89033E-11	Thickness betwee	INL_RPE_thic	TRUE	reported
0.0118535	2.00975E-23	Thickness betwee	INL_RPE_thic	TRUE	reported
0.00804523	3.94362E-15	Thickness betwee	INL_RPE_thic	TRUE	reported
0.00756911	1.9948E-16	Thickness betwee	INL_RPE_thic	TRUE	reported
0.0066143	2.57063E-11	Thickness betwee	INL_RPE_thic	TRUE	reported
0.00632209	4.605E-09	Thickness betwee	INL_RPE_thic	TRUE	reported
0.00921806	5.72859E-09	Thickness betwee	INL_RPE_thic	TRUE	reported
0.0067421	3.62371E-09	Thickness betwee	INL_RPE_thic	TRUE	reported
0.00620415	7.46486E-10	Thickness betwee	INL_RPE_thic	TRUE	reported
0.00739917	7.8187E-09	Thickness betwee	INL_RPE_thic	TRUE	reported
0.00682395	3.5598E-08	Thickness betwee	INL_RPE_thic	TRUE	reported
0.00659528	6.45056E-11	Thickness betwee	INL_RPE_thic	TRUE	reported
0.00724999	4.44528E-71	Thickness betwee	INL_RPE_thic	TRUE	reported
0.00775549	3.06729E-08	Thickness betwee	INL_RPE_thic	TRUE	reported
0.0062287	1.52203E-11	Thickness betwee	INL_RPE_thic	TRUE	reported
0.0097577	7.15398E-21	Thickness betwee	INL_RPE_thic	TRUE	reported
0.00623493	8.26678E-12	Thickness betwee	INL_RPE_thic	TRUE	reported
0.00742397	2.89816E-15	Thickness betwee	INL_RPE_thic	TRUE	reported
0.00692938	5.10971E-13	Thickness betwee	INL_RPE_thic	TRUE	reported
0.00673401	3.96346E-11	Thickness betwee	INL_RPE_thic	TRUE	reported
0.00738764	1.72554E-12	Thickness betwee	INL_RPE_thic	TRUE	reported
0.00626077	6.34797E-10	Thickness betwee	INL_RPE_thic	TRUE	reported
0.00704488	1.66123E-08	Thickness betwee	INL_RPE_thic	TRUE	reported
0.00625932	2.28332E-10	Thickness betwee	INL_RPE_thic	TRUE	reported
0.00619109	9.85352E-12	Thickness betwee	INL_RPE_thic	TRUE	reported
0.00636743	1.5037E-08	Thickness betwee	INL_RPE_thic	TRUE	reported
0.00720554	3.76649E-08	Thickness betwee	INL_RPE_thic	TRUE	reported
0.00623053	9.84798E-10	Thickness betwee	INL_RPE_thic	TRUE	reported
0.00864078	7.70319E-09	Thickness betwee	INL_RPE_thic	TRUE	reported
0.00716454	6.95964E-11	Thickness betwee	INL_RPE_thic	TRUE	reported
0.0073305	1.77782E-08	Thickness betwee	INL_RPE_thic	TRUE	reported
0.00621333	1.3312E-08	Thickness betwee	INL_RPE_thic	TRUE	reported
0.0119146	6.72403E-24	Thickness betwee	INL_RPE_thic	TRUE	reported
0.00636973	3.4796E-11	Thickness betwee	INL_RPE_thic	TRUE	reported
0.00972145	4.9805E-10	Thickness betwee	INL_RPE_thic	TRUE	reported
0.00802022	3.24275E-18	Thickness betwee	INL_RPE_thic	TRUE	reported

0.00800139	1.64237E-16	Thickness betwee	INL_RPE_thic	TRUE	reported
0.00645562	1.42627E-09	Thickness betwee	INL_RPE_thic	TRUE	reported
0.00659514	4.67199E-12	Thickness betwee	INL_RPE_thic	TRUE	reported
0.00763505	6.09163E-11	Thickness betwee	INL_RPE_thic	TRUE	reported
0.00750725	9.45182E-12	Thickness betwee	INL_RPE_thic	TRUE	reported
0.00680304	5.1176E-11	Thickness betwee	INL_RPE_thic	TRUE	reported
0.00669855	5.95106E-09	Thickness betwee	INL_RPE_thic	TRUE	reported
0.00722792	3.06306E-90	Thickness betwee	INL_RPE_thic	TRUE	reported
0.00802796	2.54786E-08	Thickness betwee	INL_RPE_thic	TRUE	reported
0.00620228	6.82885E-13	Thickness betwee	INL_RPE_thic	TRUE	reported
0.00809945	9.77382E-32	Thickness betwee	INL_RPE_thic	TRUE	reported
0.00620692	1.58076E-09	Thickness betwee	INL_RPE_thic	TRUE	reported
0.00617652	6.34127E-16	Thickness betwee	INL_RPE_thic	TRUE	reported
0.00740737	2.65061E-23	Thickness betwee	INL_RPE_thic	TRUE	reported
0.00924151	1.63327E-08	Thickness betwee	INL_RPE_thic	TRUE	reported
0.00690708	5.74323E-14	Thickness betwee	INL_RPE_thic	TRUE	reported
0.0066376	7.85129E-09	Thickness betwee	INL_RPE_thic	TRUE	reported
0.00628538	2.22721E-11	Thickness betwee	INL_ELM_thic	TRUE	reported
0.00723644	6.1558E-10	Thickness betwee	INL_ELM_thic	TRUE	reported
0.00649938	1.80021E-23	Thickness betwee	INL_ELM_thic	TRUE	reported
0.00968628	3.58306E-09	Thickness betwee	INL_ELM_thic	TRUE	reported
0.0066616	7.12773E-16	Thickness betwee	INL_ELM_thic	TRUE	reported
0.0063059	4.24957E-10	Thickness betwee	INL_ELM_thic	TRUE	reported
0.0246047	2.54994E-31	Thickness betwee	INL_ELM_thic	TRUE	reported
0.00831672	2.32645E-26	Thickness betwee	INL_ELM_thic	TRUE	reported
0.00734079	2.05002E-09	Thickness betwee	INL_ELM_thic	TRUE	reported
0.00922212	2.55117E-10	Thickness betwee	INL_ELM_thic	TRUE	reported
0.0175255	8.15729E-24	Thickness betwee	INL_ELM_thic	TRUE	reported
0.012427	8.23923E-17	Thickness betwee	INL_ELM_thic	TRUE	reported
0.00629885	3.27058E-11	Thickness betwee	INL_ELM_thic	TRUE	reported
0.00636003	1.04396E-10	Thickness betwee	INL_ELM_thic	TRUE	reported
0.00774398	2.02082E-09	Thickness betwee	INL_ELM_thic	TRUE	reported
0.00879912	5.07693E-16	Thickness betwee	INL_ELM_thic	TRUE	reported
0.00634549	3.4921E-09	Thickness betwee	INL_ELM_thic	TRUE	reported
0.0069654	7.84636E-11	Thickness betwee	INL_ELM_thic	TRUE	reported
0.00702759	1.34459E-08	Thickness betwee	INL_ELM_thic	TRUE	reported
0.00644744	1.62397E-11	Thickness betwee	INL_ELM_thic	TRUE	reported
0.0063311	7.00282E-09	Thickness betwee	INL_ELM_thic	TRUE	reported
0.00664372	1.80322E-14	Thickness betwee	INL_ELM_thic	TRUE	reported
0.00661825	5.26786E-09	Thickness betwee	INL_ELM_thic	TRUE	reported
0.012019	4.50846E-49	Thickness betwee	INL_ELM_thic	TRUE	reported
0.00645144	6.07945E-11	Thickness betwee	INL_ELM_thic	TRUE	reported
0.00782054	8.73002E-16	Thickness betwee	INL_ELM_thic	TRUE	reported
0.00649001	1.77016E-12	Thickness betwee	INL_ELM_thic	TRUE	reported

0.0105325	1.71327E-09	Thickness betwee	INL_ELM_thic	TRUE	reported
0.00768777	2.68241E-14	Thickness betwee	INL_ELM_thic	TRUE	reported
0.00630325	1.78534E-11	Thickness betwee	INL_ELM_thic	TRUE	reported
0.0172324	2.33921E-26	Thickness betwee	INL_ELM_thic	TRUE	reported
0.0106791	1.2342E-10	Thickness betwee	INL_ELM_thic	TRUE	reported
0.00828562	1.86937E-16	Thickness betwee	INL_ELM_thic	TRUE	reported
0.00970358	5.13524E-10	Thickness betwee	INL_ELM_thic	TRUE	reported
0.00706965	8.90305E-16	Thickness betwee	INL_ELM_thic	TRUE	reported
0.00940508	1.37507E-11	Thickness betwee	INL_ELM_thic	TRUE	reported
0.00912557	3.37261E-08	Thickness betwee	INL_ELM_thic	TRUE	reported
0.0210313	2.49095E-11	Thickness betwee	INL_ELM_thic	TRUE	reported
0.00642776	3.27392E-08	Thickness betwee	INL_ELM_thic	TRUE	reported
0.00917928	1.31785E-15	Thickness betwee	INL_ELM_thic	TRUE	reported
0.00689631	1.85986E-08	Thickness betwee	INL_ELM_thic	TRUE	reported
0.00723761	5.94978E-69	Thickness betwee	INL_ELM_thic	TRUE	reported
0.00688433	1.70868E-21	Thickness betwee	INL_ELM_thic	TRUE	reported
0.00650894	2.05963E-12	Thickness betwee	INL_ELM_thic	TRUE	reported
0.00661788	1.01502E-11	Thickness betwee	INL_ELM_thic	TRUE	reported
0.00739917	8.76896E-21	Thickness betwee	INL_ELM_thic	TRUE	reported
0.00679602	2.86742E-10	Thickness betwee	INL_ELM_thic	TRUE	reported
0.00685878	2.60238E-16	Thickness betwee	INL_ELM_thic	TRUE	reported
0.015545	1.66419E-16	Thickness betwee	INL_ELM_thic	TRUE	reported
0.0081671	1.11144E-12	Thickness betwee	INL_ELM_thic	TRUE	reported
0.00683007	1.10945E-09	Thickness betwee	INL_ELM_thic	TRUE	reported
0.00755618	2.98367E-08	Thickness betwee	INL_ELM_thic	TRUE	reported
0.00642138	1.20384E-08	Thickness betwee	INL_ELM_thic	TRUE	reported
0.00736339	2.1731E-131	Thickness betwee	INL_ELM_thic	TRUE	reported
0.00796138	5.98155E-19	Thickness betwee	INL_ELM_thic	TRUE	reported
0.00631126	1.77256E-09	Thickness betwee	INL_ELM_thic	TRUE	reported
0.00680134	2.59172E-10	Thickness betwee	INL_ELM_thic	TRUE	reported
0.00856916	8.79606E-12	Thickness betwee	INL_ELM_thic	TRUE	reported
0.00637667	1.69394E-12	Thickness betwee	INL_ELM_thic	TRUE	reported
0.00638131	1.01265E-09	Thickness betwee	INL_ELM_thic	TRUE	reported
0.0161913	7.38311E-17	Thickness betwee	INL_ELM_thic	TRUE	reported
0.00825258	2.2533E-269	Thickness betwee	INL_ELM_thic	TRUE	reported
0.0189209	3.97304E-52	Thickness betwee	INL_ELM_thic	TRUE	reported
0.00631987	4.90279E-21	Thickness betwee	INL_ELM_thic	TRUE	reported
0.00628386	2.44565E-09	Thickness betwee	INL_ELM_thic	TRUE	reported
0.00687334	8.47424E-11	Thickness betwee	INL_ELM_thic	TRUE	reported
0.00631288	1.0911E-13	Thickness betwee	INL_ELM_thic	TRUE	reported
0.00651023	7.03653E-12	Thickness betwee	INL_ELM_thic	TRUE	reported
0.00769353	1.1236E-10	Thickness betwee	INL_ELM_thic	TRUE	reported
0.00732262	2.76598E-17	Thickness betwee	INL_ELM_thic	TRUE	reported
0.0183321	3.12487E-11	Thickness betwee	INL_ELM_thic	TRUE	reported

0.00701134	5.28576E-10	Thickness betwee	INL_ELM_thic	TRUE	reported
0.00759275	3.03982E-27	Thickness betwee	INL_ELM_thic	TRUE	reported
0.00889135	1.12552E-14	Thickness betwee	INL_ELM_thic	TRUE	reported
0.00657502	9.13434E-27	Thickness betwee	INL_ELM_thic	TRUE	reported
0.00823352	2.73956E-08	Thickness betwee	INL_ELM_thic	TRUE	reported
0.00685784	1.1565E-24	Thickness betwee	INL_ELM_thic	TRUE	reported
0.00941261	4.37412E-16	Thickness betwee	INL_ELM_thic	TRUE	reported
0.0211389	2.49949E-12	Thickness betwee	INL_ELM_thic	TRUE	reported
0.00862259	4.69148E-09	Thickness betwee	INL_ELM_thic	TRUE	reported
0.0062841	6.3501E-17	Thickness betwee	INL_ELM_thic	TRUE	reported
0.00845444	1.4188E-08	Thickness betwee	INL_ELM_thic	TRUE	reported
0.00694495	3.25499E-25	Thickness betwee	INL_ELM_thic	TRUE	reported
0.00680195	3.15067E-09	Thickness betwee	INL_ELM_thic	TRUE	reported
0.00920248	8.13757E-10	Thickness betwee	INL_RPE_thic	TRUE	reported
0.00623955	1.9297E-15	Thickness betwee	INL_RPE_thic	TRUE	reported
0.00698476	3.34438E-08	Thickness betwee	INL_RPE_thic	TRUE	reported
0.00746471	1.14441E-28	Thickness betwee	INL_RPE_thic	TRUE	reported
0.00713455	2.46308E-09	Thickness betwee	INL_RPE_thic	TRUE	reported
0.00632798	2.64146E-17	Thickness betwee	INL_RPE_thic	TRUE	reported
0.00645303	4.10092E-17	Thickness betwee	INL_RPE_thic	TRUE	reported
0.008052	1.27437E-11	Thickness betwee	INL_RPE_thic	TRUE	reported
0.00661057	5.28604E-13	Thickness betwee	INL_RPE_thic	TRUE	reported
0.00625657	8.93122E-19	Thickness betwee	INL_RPE_thic	TRUE	reported
0.0065194	2.21627E-08	Thickness betwee	INL_RPE_thic	TRUE	reported
0.024422	8.36686E-19	Thickness betwee	INL_RPE_thic	TRUE	reported
0.00825169	4.63026E-17	Thickness betwee	INL_RPE_thic	TRUE	reported
0.00728313	1.09305E-12	Thickness betwee	INL_RPE_thic	TRUE	reported
0.0143485	1.06262E-09	Thickness betwee	INL_RPE_thic	TRUE	reported
0.00873799	2.835E-20	Thickness betwee	INL_RPE_thic	TRUE	reported
0.00691133	1.50729E-08	Thickness betwee	INL_RPE_thic	TRUE	reported
0.00639903	3.05856E-12	Thickness betwee	INL_RPE_thic	TRUE	reported
0.00694686	6.94909E-09	Thickness betwee	INL_RPE_thic	TRUE	reported
0.00623795	9.2777E-09	Thickness betwee	INL_RPE_thic	TRUE	reported
0.00623778	5.99456E-09	Thickness betwee	INL_RPE_thic	TRUE	reported
0.0119382	1.77662E-35	Thickness betwee	INL_RPE_thic	TRUE	reported
0.0064039	1.64842E-10	Thickness betwee	INL_RPE_thic	TRUE	reported
0.00796471	5.41005E-13	Thickness betwee	INL_RPE_thic	TRUE	reported
0.0076272	1.59129E-29	Thickness betwee	INL_RPE_thic	TRUE	reported
0.0170838	1.23638E-13	Thickness betwee	INL_RPE_thic	TRUE	reported
0.00664239	9.07454E-13	Thickness betwee	INL_RPE_thic	TRUE	reported
0.00838388	1.54739E-09	Thickness betwee	INL_RPE_thic	TRUE	reported
0.00701563	1.57993E-08	Thickness betwee	INL_RPE_thic	TRUE	reported
0.00929792	3.75649E-12	Thickness betwee	INL_RPE_thic	TRUE	reported
0.0091188	5.42798E-12	Thickness betwee	INL_RPE_thic	TRUE	reported

0.00911483	2.35053E-08	Thickness betwee	INL_RPE_thic	TRUE	reported
0.00684454	2.99699E-09	Thickness betwee	INL_RPE_thic	TRUE	reported
0.00718645	2.33555E-35	Thickness betwee	INL_RPE_thic	TRUE	reported
0.00679678	1.44668E-15	Thickness betwee	INL_RPE_thic	TRUE	reported
0.00626959	3.03252E-12	Thickness betwee	INL_RPE_thic	TRUE	reported
0.0065511	1.50677E-11	Thickness betwee	INL_RPE_thic	TRUE	reported
0.00734152	2.63993E-11	Thickness betwee	INL_RPE_thic	TRUE	reported
0.00624671	6.11504E-17	Thickness betwee	INL_RPE_thic	TRUE	reported
0.00734605	4.62412E-12	Thickness betwee	INL_RPE_thic	TRUE	reported
0.00884338	5.09529E-11	Thickness betwee	INL_RPE_thic	TRUE	reported
0.0068804	1.61771E-16	Thickness betwee	INL_RPE_thic	TRUE	reported
0.00964259	1.29261E-11	Thickness betwee	INL_RPE_thic	TRUE	reported
0.0081097	5.19708E-11	Thickness betwee	INL_RPE_thic	TRUE	reported
0.00627798	4.75903E-08	Thickness betwee	INL_RPE_thic	TRUE	reported
0.0066963	2.48231E-13	Thickness betwee	INL_RPE_thic	TRUE	reported
0.0073077	2.0362E-223	Thickness betwee	INL_RPE_thic	TRUE	reported
0.00827899	8.62462E-13	Thickness betwee	INL_RPE_thic	TRUE	reported
0.00626288	2.10766E-08	Thickness betwee	INL_RPE_thic	TRUE	reported
0.00681686	1.9127E-10	Thickness betwee	INL_RPE_thic	TRUE	reported
0.00849812	2.07933E-10	Thickness betwee	INL_RPE_thic	TRUE	reported
0.00634379	1.05293E-11	Thickness betwee	INL_RPE_thic	TRUE	reported
0.00627625	6.49421E-15	Thickness betwee	INL_RPE_thic	TRUE	reported
0.0160533	2.801E-11	Thickness betwee	INL_RPE_thic	TRUE	reported
0.00818704	1.3068E-139	Thickness betwee	INL_RPE_thic	TRUE	reported
0.018777	4.376E-29	Thickness betwee	INL_RPE_thic	TRUE	reported
0.00627261	2.55293E-20	Thickness betwee	INL_RPE_thic	TRUE	reported
0.00623888	2.91108E-14	Thickness betwee	INL_RPE_thic	TRUE	reported
0.00650878	2.7771E-11	Thickness betwee	INL_RPE_thic	TRUE	reported
0.00634682	3.33309E-08	Thickness betwee	INL_RPE_thic	TRUE	reported
0.00625416	7.9835E-12	Thickness betwee	INL_RPE_thic	TRUE	reported
0.00623987	9.98348E-11	Thickness betwee	INL_RPE_thic	TRUE	reported
0.00750414	1.6019E-20	Thickness betwee	INL_RPE_thic	TRUE	reported
0.00883235	2.24516E-15	Thickness betwee	INL_RPE_thic	TRUE	reported
0.00777246	1.7161E-08	Thickness betwee	INL_RPE_thic	TRUE	reported
0.00625536	1.72287E-08	Thickness betwee	INL_RPE_thic	TRUE	reported
0.00709932	2.40932E-18	Thickness betwee	INL_RPE_thic	TRUE	reported
0.010971	2.82541E-12	Thickness betwee	INL_RPE_thic	TRUE	reported
0.0121984	1.19207E-08	Thickness betwee	INL_RPE_thic	TRUE	reported
0.00852912	5.52028E-09	Thickness betwee	INL_RPE_thic	TRUE	reported
0.00689302	1.51082E-31	Thickness betwee	INL_RPE_thic	TRUE	reported
0.00674826	6.49766E-09	Thickness betwee	INL_RPE_thic	TRUE	reported
0.00920293	8.45066E-09	Thickness betwee	INL_RPE_thic	TRUE	reported
0.00620601	2.08238E-19	Thickness betwee	INL_RPE_thic	TRUE	reported
0.00742971	5.05441E-26	Thickness betwee	INL_RPE_thic	TRUE	reported

0.00629768	2.49708E-16	Thickness betwee	INL_RPE_thic	TRUE	reported
0.00642078	2.50238E-22	Thickness betwee	INL_RPE_thic	TRUE	reported
0.0080031	6.74115E-15	Thickness betwee	INL_RPE_thic	TRUE	reported
0.00657612	1.13099E-18	Thickness betwee	INL_RPE_thic	TRUE	reported
0.00622911	1.99461E-20	Thickness betwee	INL_RPE_thic	TRUE	reported
0.0242691	1.89386E-24	Thickness betwee	INL_RPE_thic	TRUE	reported
0.0085282	3.47705E-16	Thickness betwee	INL_RPE_thic	TRUE	reported
0.00725061	2.48987E-09	Thickness betwee	INL_RPE_thic	TRUE	reported
0.0144097	9.90083E-11	Thickness betwee	INL_RPE_thic	TRUE	reported
0.00764024	1.61303E-09	Thickness betwee	INL_RPE_thic	TRUE	reported
0.00869594	1.79954E-18	Thickness betwee	INL_RPE_thic	TRUE	reported
0.00687574	2.78496E-08	Thickness betwee	INL_RPE_thic	TRUE	reported
0.00636783	1.93955E-10	Thickness betwee	INL_RPE_thic	TRUE	reported
0.00824277	4.97304E-08	Thickness betwee	INL_RPE_thic	TRUE	reported
0.00625154	1.92E-09	Thickness betwee	INL_RPE_thic	TRUE	reported
0.00620871	2.13474E-08	Thickness betwee	INL_RPE_thic	TRUE	reported
0.0119981	2.76028E-32	Thickness betwee	INL_RPE_thic	TRUE	reported
0.00637317	7.29013E-10	Thickness betwee	INL_RPE_thic	TRUE	reported
0.00620838	1.73204E-09	Thickness betwee	INL_RPE_thic	TRUE	reported
0.00639878	2.64903E-09	Thickness betwee	INL_RPE_thic	TRUE	reported
0.0080698	6.44443E-14	Thickness betwee	INL_RPE_thic	TRUE	reported
0.00759443	7.22833E-28	Thickness betwee	INL_RPE_thic	TRUE	reported
0.0171534	2.20524E-12	Thickness betwee	INL_RPE_thic	TRUE	reported
0.00642491	1.58139E-09	Thickness betwee	INL_RPE_thic	TRUE	reported
0.00668906	1.01621E-11	Thickness betwee	INL_RPE_thic	TRUE	reported
0.00668005	2.4498E-08	Thickness betwee	INL_RPE_thic	TRUE	reported
0.00924241	1.00797E-15	Thickness betwee	INL_RPE_thic	TRUE	reported
0.00906798	2.6413E-12	Thickness betwee	INL_RPE_thic	TRUE	reported
0.0068183	1.66329E-12	Thickness betwee	INL_RPE_thic	TRUE	reported
0.00715226	3.63379E-31	Thickness betwee	INL_RPE_thic	TRUE	reported
0.00668345	3.72601E-15	Thickness betwee	INL_RPE_thic	TRUE	reported
0.00624471	1.77189E-11	Thickness betwee	INL_RPE_thic	TRUE	reported
0.00653004	7.62357E-12	Thickness betwee	INL_RPE_thic	TRUE	reported
0.00730556	1.19539E-13	Thickness betwee	INL_RPE_thic	TRUE	reported
0.00622146	4.77727E-13	Thickness betwee	INL_RPE_thic	TRUE	reported
0.00755346	4.87348E-11	Thickness betwee	INL_RPE_thic	TRUE	reported
0.00732065	3.77814E-11	Thickness betwee	INL_RPE_thic	TRUE	reported
0.00684663	1.29104E-17	Thickness betwee	INL_RPE_thic	TRUE	reported
0.0137926	1.83486E-16	Thickness betwee	INL_RPE_thic	TRUE	reported
0.00806955	5.92033E-10	Thickness betwee	INL_RPE_thic	TRUE	reported
0.0067382	9.80736E-15	Thickness betwee	INL_RPE_thic	TRUE	reported
0.00727012	1.0818E-228	Thickness betwee	INL_RPE_thic	TRUE	reported
0.00824058	3.16614E-12	Thickness betwee	INL_RPE_thic	TRUE	reported
0.00621899	1.40298E-08	Thickness betwee	INL_RPE_thic	TRUE	reported

0.00681148	2.88906E-12	Thickness betwee	INL_RPE_thic	TRUE	reported
0.00850764	6.25521E-09	Thickness betwee	INL_RPE_thic	TRUE	reported
0.00628383	1.20335E-10	Thickness betwee	INL_RPE_thic	TRUE	reported
0.00624344	2.51594E-15	Thickness betwee	INL_RPE_thic	TRUE	reported
0.00948251	9.62257E-12	Thickness betwee	INL_RPE_thic	TRUE	reported
0.0159927	9.91221E-10	Thickness betwee	INL_RPE_thic	TRUE	reported
0.008148	1.0328E-161	Thickness betwee	INL_RPE_thic	TRUE	reported
0.0186658	2.20388E-29	Thickness betwee	INL_RPE_thic	TRUE	reported
0.00624344	1.05732E-21	Thickness betwee	INL_RPE_thic	TRUE	reported
0.00620218	5.19089E-10	Thickness betwee	INL_RPE_thic	TRUE	reported
0.00621003	1.04063E-15	Thickness betwee	INL_RPE_thic	TRUE	reported
0.00621354	3.33013E-14	Thickness betwee	INL_RPE_thic	TRUE	reported
0.00762299	2.23345E-08	Thickness betwee	INL_RPE_thic	TRUE	reported
0.00632313	3.38771E-09	Thickness betwee	INL_RPE_thic	TRUE	reported
0.00621563	3.97202E-14	Thickness betwee	INL_RPE_thic	TRUE	reported
0.00684119	8.96797E-10	Thickness betwee	INL_RPE_thic	TRUE	reported
0.00750068	1.05847E-26	Thickness betwee	INL_RPE_thic	TRUE	reported
0.00878961	3.0961E-14	Thickness betwee	INL_RPE_thic	TRUE	reported
0.00750369	2.11053E-08	Thickness betwee	INL_RPE_thic	TRUE	reported
0.00773843	3.98922E-08	Thickness betwee	INL_RPE_thic	TRUE	reported
0.00680872	7.44235E-19	Thickness betwee	INL_RPE_thic	TRUE	reported
0.00929671	1.42676E-15	Thickness betwee	INL_RPE_thic	TRUE	reported
0.0115338	1.28043E-09	Thickness betwee	INL_RPE_thic	TRUE	reported
0.00627331	1.31951E-08	Thickness betwee	INL_RPE_thic	TRUE	reported
0.00676157	1.24641E-08	Thickness betwee	INL_RPE_thic	TRUE	reported
0.00688325	1.04732E-34	Thickness betwee	INL_RPE_thic	TRUE	reported
0.00668409	2.4504E-11	Thickness betwee	INL_RPE_thic	TRUE	reported
0.00717144	1.01004E-08	Thickness betwee	INL_RPE_thic	TRUE	reported
0.00752715	1.66878E-18	Thickness betwee	INL_RPE_thic	TRUE	reported
0.00723599	7.70143E-09	Thickness betwee	INL_RPE_thic	TRUE	reported
0.0076232	9.23488E-09	Thickness betwee	INL_RPE_thic	TRUE	reported
0.00658243	1.64012E-14	Thickness betwee	INL_RPE_thic	TRUE	reported
0.00969114	8.39274E-10	Thickness betwee	INL_RPE_thic	TRUE	reported
0.00895215	4.45495E-11	Thickness betwee	INL_RPE_thic	TRUE	reported
0.00629385	5.65904E-24	Thickness betwee	INL_RPE_thic	TRUE	reported
0.00629269	5.09216E-10	Thickness betwee	INL_RPE_thic	TRUE	reported
0.00657071	1.56997E-10	Thickness betwee	INL_RPE_thic	TRUE	reported
0.0246053	1.28511E-33	Thickness betwee	INL_RPE_thic	TRUE	reported
0.00830063	3.66541E-42	Thickness betwee	INL_RPE_thic	TRUE	reported
0.00734261	7.26273E-09	Thickness betwee	INL_RPE_thic	TRUE	reported
0.012427	2.8575E-18	Thickness betwee	INL_RPE_thic	TRUE	reported
0.00631135	1.57674E-08	Thickness betwee	INL_RPE_thic	TRUE	reported
0.014401	1.55328E-20	Thickness betwee	INL_RPE_thic	TRUE	reported
0.00696935	1.37843E-13	Thickness betwee	INL_RPE_thic	TRUE	reported

0.00949263	9.90025E-09	Thickness betwee	INL_RPE_thic	TRUE	reported
0.00696696	2.74036E-11	Thickness betwee	INL_RPE_thic	TRUE	reported
0.00702868	1.63483E-10	Thickness betwee	INL_RPE_thic	TRUE	reported
0.0106176	5.74456E-09	Thickness betwee	INL_RPE_thic	TRUE	reported
0.00633241	1.03359E-10	Thickness betwee	INL_RPE_thic	TRUE	reported
0.00664459	1.35341E-21	Thickness betwee	INL_RPE_thic	TRUE	reported
0.00661867	1.22501E-10	Thickness betwee	INL_RPE_thic	TRUE	reported
0.0149845	8.76026E-09	Thickness betwee	INL_RPE_thic	TRUE	reported
0.0120009	1.36971E-51	Thickness betwee	INL_RPE_thic	TRUE	reported
0.00645294	1.45805E-17	Thickness betwee	INL_RPE_thic	TRUE	reported
0.00784425	4.71375E-21	Thickness betwee	INL_RPE_thic	TRUE	reported
0.00628206	4.68961E-11	Thickness betwee	INL_RPE_thic	TRUE	reported
0.00654336	2.13445E-13	Thickness betwee	INL_RPE_thic	TRUE	reported
0.00768838	1.79869E-15	Thickness betwee	INL_RPE_thic	TRUE	reported
0.007021	3.77331E-08	Thickness betwee	INL_RPE_thic	TRUE	reported
0.0172304	9.75877E-24	Thickness betwee	INL_RPE_thic	TRUE	reported
0.010682	3.39161E-09	Thickness betwee	INL_RPE_thic	TRUE	reported
0.00766042	4.94044E-08	Thickness betwee	INL_RPE_thic	TRUE	reported
0.008287	3.94569E-13	Thickness betwee	INL_RPE_thic	TRUE	reported
0.00970426	1.82893E-14	Thickness betwee	INL_RPE_thic	TRUE	reported
0.00707051	1.59399E-15	Thickness betwee	INL_RPE_thic	TRUE	reported
0.00796923	1.34203E-18	Thickness betwee	INL_RPE_thic	TRUE	reported
0.00918413	6.91091E-12	Thickness betwee	INL_RPE_thic	TRUE	reported
0.0210302	4.40926E-13	Thickness betwee	INL_RPE_thic	TRUE	reported
0.00918128	4.70534E-19	Thickness betwee	INL_RPE_thic	TRUE	reported
0.00689675	5.46978E-11	Thickness betwee	INL_RPE_thic	TRUE	reported
0.00723853	6.97244E-88	Thickness betwee	INL_RPE_thic	TRUE	reported
0.00845064	5.80125E-12	Thickness betwee	INL_RPE_thic	TRUE	reported
0.00684835	4.46645E-15	Thickness betwee	INL_RPE_thic	TRUE	reported
0.00631602	8.73133E-10	Thickness betwee	INL_RPE_thic	TRUE	reported
0.00661836	1.22981E-12	Thickness betwee	INL_RPE_thic	TRUE	reported
0.00743077	3.10517E-29	Thickness betwee	INL_RPE_thic	TRUE	reported
0.00628972	3.06502E-20	Thickness betwee	INL_RPE_thic	TRUE	reported
0.00670164	3.38583E-11	Thickness betwee	INL_RPE_thic	TRUE	reported
0.0089192	7.99121E-10	Thickness betwee	INL_RPE_thic	TRUE	reported
0.00698357	1.93516E-09	Thickness betwee	INL_RPE_thic	TRUE	reported
0.0139831	2.27065E-15	Thickness betwee	INL_RPE_thic	TRUE	reported
0.00816815	1.881E-15	Thickness betwee	INL_RPE_thic	TRUE	reported
0.0299378	4.42693E-08	Thickness betwee	INL_RPE_thic	TRUE	reported
0.0063269	2.35291E-11	Thickness betwee	INL_RPE_thic	TRUE	reported
0.00636452	5.11917E-13	Thickness betwee	INL_RPE_thic	TRUE	reported
0.00667316	2.82062E-10	Thickness betwee	INL_RPE_thic	TRUE	reported
0.0074371	4.77571E-08	Thickness betwee	INL_RPE_thic	TRUE	reported
0.00736394	2.075E-141	Thickness betwee	INL_RPE_thic	TRUE	reported

0.00796784	8.89114E-22	Thickness betwee	INL_RPE_thic	TRUE	reported
0.00686112	7.47406E-09	Thickness betwee	INL_RPE_thic	TRUE	reported
0.00697683	1.51456E-08	Thickness betwee	INL_RPE_thic	TRUE	reported
0.00685488	9.67166E-21	Thickness betwee	INL_RPE_thic	TRUE	reported
0.00856843	2.01382E-10	Thickness betwee	INL_RPE_thic	TRUE	reported
0.00637733	7.41869E-11	Thickness betwee	INL_RPE_thic	TRUE	reported
0.00632819	2.28119E-18	Thickness betwee	INL_RPE_thic	TRUE	reported
0.00960109	4.93532E-09	Thickness betwee	INL_RPE_thic	TRUE	reported
0.0161875	3.35851E-13	Thickness betwee	INL_RPE_thic	TRUE	reported
0.00825352	7.6392E-262	Thickness betwee	INL_RPE_thic	TRUE	reported
0.0189282	1.93802E-49	Thickness betwee	INL_RPE_thic	TRUE	reported
0.0063201	1.66937E-17	Thickness betwee	INL_RPE_thic	TRUE	reported
0.00690592	2.55642E-10	Thickness betwee	INL_RPE_thic	TRUE	reported
0.00629208	9.68505E-18	Thickness betwee	INL_RPE_thic	TRUE	reported
0.00739282	2.6851E-11	Thickness betwee	INL_RPE_thic	TRUE	reported
0.00640987	3.9757E-11	Thickness betwee	INL_RPE_thic	TRUE	reported
0.00684901	1.61397E-08	Thickness betwee	INL_RPE_thic	TRUE	reported
0.0151907	1.15041E-15	Thickness betwee	INL_RPE_thic	TRUE	reported
0.00627684	3.69984E-11	Thickness betwee	INL_RPE_thic	TRUE	reported
0.0069518	1.86833E-12	Thickness betwee	INL_RPE_thic	TRUE	reported
0.00799977	5.07106E-14	Thickness betwee	INL_RPE_thic	TRUE	reported
0.00763492	5.60501E-19	Thickness betwee	INL_RPE_thic	TRUE	reported
0.00898131	2.98234E-10	Thickness betwee	INL_RPE_thic	TRUE	reported
0.00685775	9.32362E-15	Thickness betwee	INL_RPE_thic	TRUE	reported
0.00827792	9.233E-20	Thickness betwee	INL_RPE_thic	TRUE	reported
0.0211336	2.82369E-09	Thickness betwee	INL_RPE_thic	TRUE	reported
0.00628602	5.84963E-15	Thickness betwee	INL_RPE_thic	TRUE	reported
0.00635124	9.18856E-21	Thickness betwee	INL_RPE_thic	TRUE	reported
0.00692691	4.01854E-08	Thickness betwee	INL_RPE_thic	TRUE	reported
0.0100391	3.19861E-20	Thickness betwee	INL_RPE_thic	TRUE	reported
0.00680052	6.85769E-09	Thickness betwee	INL_RPE_thic	TRUE	reported
0.00699157	5.21169E-09	Thickness betwee	INL_RPE_thic	TRUE	reported
0.00751162	4.5363E-18	Thickness betwee	INL_RPE_thic	TRUE	reported
0.00714565	2.63945E-08	Thickness betwee	INL_RPE_thic	TRUE	reported
0.00820599	1.50585E-09	Thickness betwee	INL_RPE_thic	TRUE	reported
0.00646262	2.66088E-16	Thickness betwee	INL_RPE_thic	TRUE	reported
0.00661712	2.86856E-15	Thickness betwee	INL_RPE_thic	TRUE	reported
0.00627516	8.17119E-27	Thickness betwee	INL_RPE_thic	TRUE	reported
0.00625559	4.69878E-08	Thickness betwee	INL_RPE_thic	TRUE	reported
0.00652904	2.33818E-10	Thickness betwee	INL_RPE_thic	TRUE	reported
0.0244513	3.83922E-37	Thickness betwee	INL_RPE_thic	TRUE	reported
0.00861215	4.04674E-37	Thickness betwee	INL_RPE_thic	TRUE	reported
0.012388	1.61706E-17	Thickness betwee	INL_RPE_thic	TRUE	reported
0.0062693	2.25929E-09	Thickness betwee	INL_RPE_thic	TRUE	reported

0.0143235	2.17621E-19	Thickness betwee	INL_RPE_thic	TRUE	reported
0.00692328	9.92473E-13	Thickness betwee	INL_RPE_thic	TRUE	reported
0.00908512	5.03186E-09	Thickness betwee	INL_RPE_thic	TRUE	reported
0.00943645	2.60205E-08	Thickness betwee	INL_RPE_thic	TRUE	reported
0.00692177	1.01938E-12	Thickness betwee	INL_RPE_thic	TRUE	reported
0.00698637	3.5486E-09	Thickness betwee	INL_RPE_thic	TRUE	reported
0.00627634	1.62862E-11	Thickness betwee	INL_RPE_thic	TRUE	reported
0.00660031	6.2649E-19	Thickness betwee	INL_RPE_thic	TRUE	reported
0.00657846	5.17939E-13	Thickness betwee	INL_RPE_thic	TRUE	reported
0.0120688	3.0458E-51	Thickness betwee	INL_RPE_thic	TRUE	reported
0.00641676	2.02685E-14	Thickness betwee	INL_RPE_thic	TRUE	reported
0.00779679	1.83413E-19	Thickness betwee	INL_RPE_thic	TRUE	reported
0.0062491	6.65139E-10	Thickness betwee	INL_RPE_thic	TRUE	reported
0.0065047	5.57999E-10	Thickness betwee	INL_RPE_thic	TRUE	reported
0.00764097	1.49472E-16	Thickness betwee	INL_RPE_thic	TRUE	reported
0.0171433	8.75186E-25	Thickness betwee	INL_RPE_thic	TRUE	reported
0.010608	2.82006E-09	Thickness betwee	INL_RPE_thic	TRUE	reported
0.00817571	8.03037E-13	Thickness betwee	INL_RPE_thic	TRUE	reported
0.00964175	1.06401E-14	Thickness betwee	INL_RPE_thic	TRUE	reported
0.0063274	1.18587E-08	Thickness betwee	INL_RPE_thic	TRUE	reported
0.00701968	7.28882E-16	Thickness betwee	INL_RPE_thic	TRUE	reported
0.00791441	4.3588E-17	Thickness betwee	INL_RPE_thic	TRUE	reported
0.00912922	3.01688E-11	Thickness betwee	INL_RPE_thic	TRUE	reported
0.0209344	4.16708E-14	Thickness betwee	INL_RPE_thic	TRUE	reported
0.00716826	1.07845E-08	Thickness betwee	INL_RPE_thic	TRUE	reported
0.00911279	3.90071E-17	Thickness betwee	INL_RPE_thic	TRUE	reported
0.00686126	1.45646E-13	Thickness betwee	INL_RPE_thic	TRUE	reported
0.00720043	1.32994E-85	Thickness betwee	INL_RPE_thic	TRUE	reported
0.0067257	6.90191E-17	Thickness betwee	INL_RPE_thic	TRUE	reported
0.00627312	1.7999E-11	Thickness betwee	INL_RPE_thic	TRUE	reported
0.00759145	1.04336E-08	Thickness betwee	INL_RPE_thic	TRUE	reported
0.00656892	1.18711E-11	Thickness betwee	INL_RPE_thic	TRUE	reported
0.00738168	3.63086E-28	Thickness betwee	INL_RPE_thic	TRUE	reported
0.00626263	5.82267E-20	Thickness betwee	INL_RPE_thic	TRUE	reported
0.00666286	1.29284E-09	Thickness betwee	INL_RPE_thic	TRUE	reported
0.00885754	6.13436E-10	Thickness betwee	INL_RPE_thic	TRUE	reported
0.00694172	2.49967E-09	Thickness betwee	INL_RPE_thic	TRUE	reported
0.0158657	1.09622E-16	Thickness betwee	INL_RPE_thic	TRUE	reported
0.00812097	9.21125E-15	Thickness betwee	INL_RPE_thic	TRUE	reported
0.00628249	7.16159E-13	Thickness betwee	INL_RPE_thic	TRUE	reported
0.00626835	1.5105E-17	Thickness betwee	INL_RPE_thic	TRUE	reported
0.00671145	2.2186E-10	Thickness betwee	INL_RPE_thic	TRUE	reported
0.00646328	2.80726E-08	Thickness betwee	INL_RPE_thic	TRUE	reported
0.00731836	8.035E-135	Thickness betwee	INL_RPE_thic	TRUE	reported

0.00791719	3.5322E-21	Thickness betwee	INL_RPE_thic	TRUE	reported
0.0068157	1.81021E-08	Thickness betwee	INL_RPE_thic	TRUE	reported
0.0068152	5.64002E-20	Thickness betwee	INL_RPE_thic	TRUE	reported
0.00855481	1.7986E-09	Thickness betwee	INL_RPE_thic	TRUE	reported
0.0063382	2.06478E-09	Thickness betwee	INL_RPE_thic	TRUE	reported
0.00627729	3.49308E-21	Thickness betwee	INL_RPE_thic	TRUE	reported
0.00955064	1.09028E-11	Thickness betwee	INL_RPE_thic	TRUE	reported
0.00653561	1.23431E-08	Thickness betwee	INL_RPE_thic	TRUE	reported
0.0161158	1.01199E-14	Thickness betwee	INL_RPE_thic	TRUE	reported
0.00819742	4.3781E-272	Thickness betwee	INL_RPE_thic	TRUE	reported
0.0187996	4.70922E-46	Thickness betwee	INL_RPE_thic	TRUE	reported
0.00628582	2.89138E-18	Thickness betwee	INL_RPE_thic	TRUE	reported
0.00686728	1.96218E-10	Thickness betwee	INL_RPE_thic	TRUE	reported
0.00625013	3.10378E-18	Thickness betwee	INL_RPE_thic	TRUE	reported
0.00734566	1.00876E-09	Thickness betwee	INL_RPE_thic	TRUE	reported
0.00632286	6.71636E-10	Thickness betwee	INL_RPE_thic	TRUE	reported
0.00680459	1.22088E-10	Thickness betwee	INL_RPE_thic	TRUE	reported
0.0151801	6.43131E-15	Thickness betwee	INL_RPE_thic	TRUE	reported
0.00626075	1.4304E-15	Thickness betwee	INL_RPE_thic	TRUE	reported
0.00689534	7.43102E-15	Thickness betwee	INL_RPE_thic	TRUE	reported
0.00795151	6.70972E-18	Thickness betwee	INL_RPE_thic	TRUE	reported
0.00697671	1.64646E-09	Thickness betwee	INL_RPE_thic	TRUE	reported
0.00755062	4.1156E-19	Thickness betwee	INL_RPE_thic	TRUE	reported
0.0074735	3.03425E-15	Thickness betwee	INL_RPE_thic	TRUE	reported
0.00822787	1.11738E-23	Thickness betwee	INL_RPE_thic	TRUE	reported
0.0210166	1.13691E-09	Thickness betwee	INL_RPE_thic	TRUE	reported
0.00624289	4.00923E-12	Thickness betwee	INL_RPE_thic	TRUE	reported
0.00631434	6.49276E-20	Thickness betwee	INL_RPE_thic	TRUE	reported
0.0068076	1.00409E-08	Thickness betwee	INL_RPE_thic	TRUE	reported
0.00692305	2.01105E-20	Thickness betwee	INL_RPE_thic	TRUE	reported
0.0075501	1.76706E-08	Thickness betwee	ISOS_RPE_thi	TRUE	reported
0.00706241	1.12781E-17	Thickness betwee	ISOS_RPE_thi	TRUE	reported
0.00748604	6.39275E-33	Thickness betwee	ISOS_RPE_thi	TRUE	reported
0.00631454	7.73919E-53	Thickness betwee	ISOS_RPE_thi	TRUE	reported
0.00636014	1.53905E-08	Thickness betwee	ISOS_RPE_thi	TRUE	reported
0.00687438	9.20143E-11	Thickness betwee	ISOS_RPE_thi	TRUE	reported
0.00642303	1.32022E-12	Thickness betwee	ISOS_RPE_thi	TRUE	reported
0.00639854	6.80209E-09	Thickness betwee	ISOS_RPE_thi	TRUE	reported
0.00667974	1.09617E-09	Thickness betwee	ISOS_RPE_thi	TRUE	reported
0.0107003	5.40661E-24	Thickness betwee	ISOS_RPE_thi	TRUE	reported
0.0143269	6.30928E-19	Thickness betwee	ISOS_RPE_thi	TRUE	reported
0.0219001	1.9296E-08	Thickness betwee	ISOS_RPE_thi	TRUE	reported
0.0109704	2.24223E-10	Thickness betwee	ISOS_RPE_thi	TRUE	reported
0.00708374	5.4671E-10	Thickness betwee	ISOS_RPE_thi	TRUE	reported

0.00765286	1.13011E-09	Thickness betwee	ISOS_RPE_thi	TRUE	reported
0.0088479	9.54126E-10	Thickness betwee	ISOS_RPE_thi	TRUE	reported
0.0196488	4.0231E-08	Thickness betwee	ISOS_RPE_thi	TRUE	reported
0.00710703	2.86027E-10	Thickness betwee	ISOS_RPE_thi	TRUE	reported
0.0201094	7.15064E-12	Thickness betwee	ISOS_RPE_thi	TRUE	reported
0.020153	3.54379E-10	Thickness betwee	ISOS_RPE_thi	TRUE	reported
0.00648064	3.00577E-11	Thickness betwee	ISOS_RPE_thi	TRUE	reported
0.00963664	1.06009E-19	Thickness betwee	ISOS_RPE_thi	TRUE	reported
0.00674081	5.80738E-10	Thickness betwee	ISOS_RPE_thi	TRUE	reported
0.006249	1.29572E-09	Thickness betwee	ISOS_RPE_thi	TRUE	reported
0.00941447	6.14165E-24	Thickness betwee	ISOS_RPE_thi	TRUE	reported
0.0169433	3.07772E-09	Thickness betwee	ISOS_RPE_thi	TRUE	reported
0.00622862	5.13306E-14	Thickness betwee	ISOS_RPE_thi	TRUE	reported
0.00757633	2.51038E-54	Thickness betwee	ISOS_RPE_thi	TRUE	reported
0.00815918	2.48849E-09	Thickness betwee	ISOS_RPE_thi	TRUE	reported
0.00759983	1.48159E-13	Thickness betwee	ISOS_RPE_thi	TRUE	reported
0.0105673	2.10957E-08	Thickness betwee	ISOS_RPE_thi	TRUE	reported
0.00663968	7.3589E-18	Thickness betwee	ISOS_RPE_thi	TRUE	reported
0.00626743	1.55212E-14	Thickness betwee	ISOS_RPE_thi	TRUE	reported
0.0072911	2.21997E-51	Thickness betwee	ISOS_RPE_thi	TRUE	reported
0.00665587	9.61903E-09	Thickness betwee	ISOS_RPE_thi	TRUE	reported
0.00675169	4.33367E-16	Thickness betwee	ISOS_RPE_thi	TRUE	reported
0.0112129	1.69765E-13	Thickness betwee	ISOS_RPE_thi	TRUE	reported
0.00763646	6.19075E-10	Thickness betwee	ISOS_RPE_thi	TRUE	reported
0.00634364	4.96584E-17	Thickness betwee	ISOS_RPE_thi	TRUE	reported
0.018202	7.23457E-26	Thickness betwee	ISOS_RPE_thi	TRUE	reported
0.00623613	1.09134E-15	Thickness betwee	ISOS_RPE_thi	TRUE	reported
0.00763071	6.25742E-10	Thickness betwee	ISOS_RPE_thi	TRUE	reported
0.00851048	3.28327E-11	Thickness betwee	ISOS_RPE_thi	TRUE	reported
0.00619549	2.8045E-24	Thickness betwee	ISOS_RPE_thi	TRUE	reported
0.0066013	1.63923E-11	Thickness betwee	ISOS_RPE_thi	TRUE	reported
0.00754971	5.08023E-09	Thickness betwee	ISOS_RPE_thi	TRUE	reported
0.00708558	3.3987E-16	Thickness betwee	ISOS_RPE_thi	TRUE	reported
0.00748759	3.89397E-26	Thickness betwee	ISOS_RPE_thi	TRUE	reported
0.00631331	1.60355E-59	Thickness betwee	ISOS_RPE_thi	TRUE	reported
0.00626021	6.48792E-14	Thickness betwee	ISOS_RPE_thi	TRUE	reported
0.0167572	5.87451E-09	Thickness betwee	ISOS_RPE_thi	TRUE	reported
0.0173638	1.60593E-24	Thickness betwee	ISOS_RPE_thi	TRUE	reported
0.0144391	1.27413E-18	Thickness betwee	ISOS_RPE_thi	TRUE	reported
0.0203025	1.50566E-08	Thickness betwee	ISOS_RPE_thi	TRUE	reported
0.00764962	1.17988E-08	Thickness betwee	ISOS_RPE_thi	TRUE	reported
0.00627906	4.98642E-08	Thickness betwee	ISOS_RPE_thi	TRUE	reported
0.0195761	2.64637E-11	Thickness betwee	ISOS_RPE_thi	TRUE	reported
0.0202122	1.85333E-13	Thickness betwee	ISOS_RPE_thi	TRUE	reported

0.00648058	2.63846E-12	Thickness betwee	ISOS_RPE_thi	TRUE	reported
0.00963683	5.28485E-25	Thickness betwee	ISOS_RPE_thi	TRUE	reported
0.00630952	4.3174E-08	Thickness betwee	ISOS_RPE_thi	TRUE	reported
0.0067305	2.39605E-10	Thickness betwee	ISOS_RPE_thi	TRUE	reported
0.00627887	5.49066E-09	Thickness betwee	ISOS_RPE_thi	TRUE	reported
0.00942527	3.96733E-20	Thickness betwee	ISOS_RPE_thi	TRUE	reported
0.0169617	1.70331E-08	Thickness betwee	ISOS_RPE_thi	TRUE	reported
0.00622837	9.93045E-12	Thickness betwee	ISOS_RPE_thi	TRUE	reported
0.0076043	4.05789E-57	Thickness betwee	ISOS_RPE_thi	TRUE	reported
0.00919158	1.15132E-09	Thickness betwee	ISOS_RPE_thi	TRUE	reported
0.00793404	3.43663E-14	Thickness betwee	ISOS_RPE_thi	TRUE	reported
0.00664376	2.08494E-17	Thickness betwee	ISOS_RPE_thi	TRUE	reported
0.00626782	2.85952E-15	Thickness betwee	ISOS_RPE_thi	TRUE	reported
0.00728709	3.15987E-56	Thickness betwee	ISOS_RPE_thi	TRUE	reported
0.00665513	3.2153E-08	Thickness betwee	ISOS_RPE_thi	TRUE	reported
0.00683649	2.84411E-15	Thickness betwee	ISOS_RPE_thi	TRUE	reported
0.0112137	7.66452E-13	Thickness betwee	ISOS_RPE_thi	TRUE	reported
0.00764417	1.88245E-14	Thickness betwee	ISOS_RPE_thi	TRUE	reported
0.00634211	1.41826E-14	Thickness betwee	ISOS_RPE_thi	TRUE	reported
0.0181805	6.32543E-27	Thickness betwee	ISOS_RPE_thi	TRUE	reported
0.00777392	3.14257E-08	Thickness betwee	ISOS_RPE_thi	TRUE	reported
0.00656119	5.8423E-21	Thickness betwee	ISOS_RPE_thi	TRUE	reported
0.00768662	2.14998E-08	Thickness betwee	ISOS_RPE_thi	TRUE	reported
0.00847546	7.99068E-19	Thickness betwee	ISOS_RPE_thi	TRUE	reported
0.00625041	1.05569E-27	Thickness betwee	ISOS_RPE_thi	TRUE	reported
0.00659995	3.54899E-10	Thickness betwee	ISOS_RPE_thi	TRUE	reported
0.00761592	1.35396E-10	Thickness betwee	ISOS_RPE_thi	TRUE	reported
0.00712599	5.39987E-24	Thickness betwee	ISOS_RPE_thi	TRUE	reported
0.0075233	1.30285E-33	Thickness betwee	ISOS_RPE_thi	TRUE	reported
0.00636986	2.75944E-40	Thickness betwee	ISOS_RPE_thi	TRUE	reported
0.0069352	7.03465E-15	Thickness betwee	ISOS_RPE_thi	TRUE	reported
0.00686522	8.41318E-10	Thickness betwee	ISOS_RPE_thi	TRUE	reported
0.0108088	8.27301E-29	Thickness betwee	ISOS_RPE_thi	TRUE	reported
0.0144527	9.33682E-29	Thickness betwee	ISOS_RPE_thi	TRUE	reported
0.00641023	1.36017E-08	Thickness betwee	ISOS_RPE_thi	TRUE	reported
0.0110552	1.29641E-12	Thickness betwee	ISOS_RPE_thi	TRUE	reported
0.00770861	4.67268E-16	Thickness betwee	ISOS_RPE_thi	TRUE	reported
0.00892357	9.14784E-16	Thickness betwee	ISOS_RPE_thi	TRUE	reported
0.00730757	2.13969E-10	Thickness betwee	ISOS_RPE_thi	TRUE	reported
0.00813794	2.64635E-10	Thickness betwee	ISOS_RPE_thi	TRUE	reported
0.00716936	3.14475E-08	Thickness betwee	ISOS_RPE_thi	TRUE	reported
0.020334	2.0153E-11	Thickness betwee	ISOS_RPE_thi	TRUE	reported
0.0203596	1.37168E-10	Thickness betwee	ISOS_RPE_thi	TRUE	reported
0.00653811	1.82916E-20	Thickness betwee	ISOS_RPE_thi	TRUE	reported

0.00972062	4.55331E-14	Thickness betwee	ISOS_RPE_thi	TRUE	reported
0.00674646	2.64312E-10	Thickness betwee	ISOS_RPE_thi	TRUE	reported
0.00679759	2.3108E-18	Thickness betwee	ISOS_RPE_thi	TRUE	reported
0.00776476	3.07205E-13	Thickness betwee	ISOS_RPE_thi	TRUE	reported
0.00949775	1.26678E-21	Thickness betwee	ISOS_RPE_thi	TRUE	reported
0.00702368	5.80544E-11	Thickness betwee	ISOS_RPE_thi	TRUE	reported
0.00764202	2.1416E-12	Thickness betwee	ISOS_RPE_thi	TRUE	reported
0.00628617	2.48473E-25	Thickness betwee	ISOS_RPE_thi	TRUE	reported
0.0078766	5.95457E-39	Thickness betwee	ISOS_RPE_thi	TRUE	reported
0.00823605	3.11578E-11	Thickness betwee	ISOS_RPE_thi	TRUE	reported
0.00772354	8.36125E-17	Thickness betwee	ISOS_RPE_thi	TRUE	reported
0.00669978	3.17762E-27	Thickness betwee	ISOS_RPE_thi	TRUE	reported
0.00630993	1.08432E-19	Thickness betwee	ISOS_RPE_thi	TRUE	reported
0.00735599	3.32301E-84	Thickness betwee	ISOS_RPE_thi	TRUE	reported
0.00671329	8.89079E-12	Thickness betwee	ISOS_RPE_thi	TRUE	reported
0.00631973	3.92455E-12	Thickness betwee	ISOS_RPE_thi	TRUE	reported
0.0068095	5.21372E-12	Thickness betwee	ISOS_RPE_thi	TRUE	reported
0.0113122	1.05859E-21	Thickness betwee	ISOS_RPE_thi	TRUE	reported
0.00770737	3.14468E-08	Thickness betwee	ISOS_RPE_thi	TRUE	reported
0.00640103	1.56449E-35	Thickness betwee	ISOS_RPE_thi	TRUE	reported
0.00633944	1.87144E-09	Thickness betwee	ISOS_RPE_thi	TRUE	reported
0.0183408	6.25621E-29	Thickness betwee	ISOS_RPE_thi	TRUE	reported
0.00783906	1.65463E-08	Thickness betwee	ISOS_RPE_thi	TRUE	reported
0.00679309	6.05571E-13	Thickness betwee	ISOS_RPE_thi	TRUE	reported
0.00656943	7.46497E-32	Thickness betwee	ISOS_RPE_thi	TRUE	reported
0.00769704	5.9014E-10	Thickness betwee	ISOS_RPE_thi	TRUE	reported
0.0062507	5.35874E-28	Thickness betwee	ISOS_RPE_thi	TRUE	reported
0.00665567	9.66328E-10	Thickness betwee	ISOS_RPE_thi	TRUE	reported
0.00764295	2.20125E-08	Thickness betwee	ISOS_RPE_thi	TRUE	reported
0.00712525	1.61052E-24	Thickness betwee	ISOS_RPE_thi	TRUE	reported
0.00753126	1.09251E-32	Thickness betwee	ISOS_RPE_thi	TRUE	reported
0.00634978	3.14339E-40	Thickness betwee	ISOS_RPE_thi	TRUE	reported
0.00628874	3.27809E-10	Thickness betwee	ISOS_RPE_thi	TRUE	reported
0.00683898	2.82168E-09	Thickness betwee	ISOS_RPE_thi	TRUE	reported
0.0174572	2.11731E-22	Thickness betwee	ISOS_RPE_thi	TRUE	reported
0.0145122	1.4126E-27	Thickness betwee	ISOS_RPE_thi	TRUE	reported
0.0110323	4.72751E-12	Thickness betwee	ISOS_RPE_thi	TRUE	reported
0.0077058	7.16548E-12	Thickness betwee	ISOS_RPE_thi	TRUE	reported
0.00988221	1.93407E-15	Thickness betwee	ISOS_RPE_thi	TRUE	reported
0.00738753	1.42309E-12	Thickness betwee	ISOS_RPE_thi	TRUE	reported
0.0202514	1.19648E-10	Thickness betwee	ISOS_RPE_thi	TRUE	reported
0.0203151	3.79572E-15	Thickness betwee	ISOS_RPE_thi	TRUE	reported
0.00651961	9.75036E-19	Thickness betwee	ISOS_RPE_thi	TRUE	reported
0.00969351	3.93285E-18	Thickness betwee	ISOS_RPE_thi	TRUE	reported

0.00634636	4.62761E-13	Thickness betwee	ISOS_RPE_thi	TRUE	reported
0.00677042	8.18599E-15	Thickness betwee	ISOS_RPE_thi	TRUE	reported
0.00774087	4.73055E-09	Thickness betwee	ISOS_RPE_thi	TRUE	reported
0.0094829	1.50182E-17	Thickness betwee	ISOS_RPE_thi	TRUE	reported
0.00700367	2.74561E-10	Thickness betwee	ISOS_RPE_thi	TRUE	reported
0.0170314	5.81971E-09	Thickness betwee	ISOS_RPE_thi	TRUE	reported
0.00730388	4.2534E-08	Thickness betwee	ISOS_RPE_thi	TRUE	reported
0.00626542	8.5513E-21	Thickness betwee	ISOS_RPE_thi	TRUE	reported
0.00762252	1.46862E-39	Thickness betwee	ISOS_RPE_thi	TRUE	reported
0.00820653	1.89006E-13	Thickness betwee	ISOS_RPE_thi	TRUE	reported
0.00934562	3.35118E-10	Thickness betwee	ISOS_RPE_thi	TRUE	reported
0.00764498	2.39793E-15	Thickness betwee	ISOS_RPE_thi	TRUE	reported
0.00668369	1.56817E-30	Thickness betwee	ISOS_RPE_thi	TRUE	reported
0.00630325	6.24231E-23	Thickness betwee	ISOS_RPE_thi	TRUE	reported
0.00733017	7.49056E-92	Thickness betwee	ISOS_RPE_thi	TRUE	reported
0.00669167	6.8359E-10	Thickness betwee	ISOS_RPE_thi	TRUE	reported
0.00630479	6.70921E-13	Thickness betwee	ISOS_RPE_thi	TRUE	reported
0.00687789	5.16925E-13	Thickness betwee	ISOS_RPE_thi	TRUE	reported
0.0112797	5.86622E-28	Thickness betwee	ISOS_RPE_thi	TRUE	reported
0.00769004	6.47939E-14	Thickness betwee	ISOS_RPE_thi	TRUE	reported
0.00638156	3.18047E-31	Thickness betwee	ISOS_RPE_thi	TRUE	reported
0.00651965	1.29761E-10	Thickness betwee	ISOS_RPE_thi	TRUE	reported
0.0182787	1.21822E-26	Thickness betwee	ISOS_RPE_thi	TRUE	reported
0.00781675	5.23445E-09	Thickness betwee	ISOS_RPE_thi	TRUE	reported
0.00677016	1.65724E-11	Thickness betwee	ISOS_RPE_thi	TRUE	reported
0.00630943	1.12965E-44	Thickness betwee	ISOS_RPE_thi	TRUE	reported
0.00624443	2.05862E-10	Thickness betwee	ISOS_RPE_thi	TRUE	reported
0.00672038	4.20852E-08	Thickness betwee	ISOS_RPE_thi	TRUE	reported
0.00623116	2.02379E-31	Thickness betwee	ISOS_RPE_thi	TRUE	reported
0.00637898	1.49161E-11	Thickness betwee	ISOS_RPE_thi	TRUE	reported
0.00766489	1.47589E-10	Thickness betwee	ISOS_RPE_thi	TRUE	reported
0.00635822	3.76552E-15	Thickness betwee	ISOS_RPE_thi	TRUE	reported
0.00708447	1.33828E-22	Thickness betwee	ISOS_RPE_thi	TRUE	reported
0.00757983	2.73302E-23	Thickness betwee	ISOS_RPE_thi	TRUE	reported
0.00641053	1.8113E-25	Thickness betwee	ISOS_RPE_thi	TRUE	reported
0.00633203	4.44187E-14	Thickness betwee	ISOS_RPE_thi	TRUE	reported
0.00694214	1.4552E-09	Thickness betwee	ISOS_RPE_thi	TRUE	reported
0.00652099	1.40806E-16	Thickness betwee	ISOS_RPE_thi	TRUE	reported
0.00738942	1.34187E-08	Thickness betwee	ISOS_RPE_thi	TRUE	reported
0.0108849	2.98957E-17	Thickness betwee	ISOS_RPE_thi	TRUE	reported
0.0145511	2.95013E-46	Thickness betwee	ISOS_RPE_thi	TRUE	reported
0.00657482	1.57937E-08	Thickness betwee	ISOS_RPE_thi	TRUE	reported
0.00776176	8.94029E-20	Thickness betwee	ISOS_RPE_thi	TRUE	reported
0.00898505	2.27357E-27	Thickness betwee	ISOS_RPE_thi	TRUE	reported

0.00636032	1.59216E-08	Thickness betwee	ISOS_RPE_thi	TRUE	reported
0.00839544	7.29221E-10	Thickness betwee	ISOS_RPE_thi	TRUE	reported
0.0122496	1.07709E-10	Thickness betwee	ISOS_RPE_thi	TRUE	reported
0.00702345	1.46259E-09	Thickness betwee	ISOS_RPE_thi	TRUE	reported
0.00658375	2.39468E-19	Thickness betwee	ISOS_RPE_thi	TRUE	reported
0.0120936	4.4811E-08	Thickness betwee	ISOS_RPE_thi	TRUE	reported
0.00691034	2.30112E-08	Thickness betwee	ISOS_RPE_thi	TRUE	reported
0.00699481	6.01428E-28	Thickness betwee	ISOS_RPE_thi	TRUE	reported
0.00824354	7.42272E-12	Thickness betwee	ISOS_RPE_thi	TRUE	reported
0.00684605	8.04892E-19	Thickness betwee	ISOS_RPE_thi	TRUE	reported
0.00813359	2.78091E-16	Thickness betwee	ISOS_RPE_thi	TRUE	reported
0.00957089	3.0526E-24	Thickness betwee	ISOS_RPE_thi	TRUE	reported
0.0078414	4.55918E-08	Thickness betwee	ISOS_RPE_thi	TRUE	reported
0.00633968	1.6363E-59	Thickness betwee	ISOS_RPE_thi	TRUE	reported
0.00756151	2.2175E-13	Thickness betwee	ISOS_RPE_thi	TRUE	reported
0.00829712	3.41538E-10	Thickness betwee	ISOS_RPE_thi	TRUE	reported
0.00773341	1.8166E-14	Thickness betwee	ISOS_RPE_thi	TRUE	reported
0.00690115	2.71076E-19	Thickness betwee	ISOS_RPE_thi	TRUE	reported
0.00635081	6.7646E-19	Thickness betwee	ISOS_RPE_thi	TRUE	reported
0.0074083	9.0748E-35	Thickness betwee	ISOS_RPE_thi	TRUE	reported
0.00692259	2.37962E-10	Thickness betwee	ISOS_RPE_thi	TRUE	reported
0.006762	1.1274E-08	Thickness betwee	ISOS_RPE_thi	TRUE	reported
0.00636526	1.57669E-24	Thickness betwee	ISOS_RPE_thi	TRUE	reported
0.00689294	1.51935E-10	Thickness betwee	ISOS_RPE_thi	TRUE	reported
0.0113976	6.02629E-24	Thickness betwee	ISOS_RPE_thi	TRUE	reported
0.00850296	1.08956E-14	Thickness betwee	ISOS_RPE_thi	TRUE	reported
0.00644687	1.09949E-44	Thickness betwee	ISOS_RPE_thi	TRUE	reported
0.00638262	9.07277E-11	Thickness betwee	ISOS_RPE_thi	TRUE	reported
0.0184485	3.52987E-24	Thickness betwee	ISOS_RPE_thi	TRUE	reported
0.00804488	3.79822E-11	Thickness betwee	ISOS_RPE_thi	TRUE	reported
0.00684189	1.01517E-12	Thickness betwee	ISOS_RPE_thi	TRUE	reported
0.00665573	1.66218E-31	Thickness betwee	ISOS_RPE_thi	TRUE	reported
0.0082466	4.77245E-08	Thickness betwee	ISOS_RPE_thi	TRUE	reported
0.00674595	4.51371E-15	Thickness betwee	ISOS_RPE_thi	TRUE	reported
0.00629357	1.58E-29	Thickness betwee	ISOS_RPE_thi	TRUE	reported
0.00637419	8.59713E-14	Thickness betwee	ISOS_RPE_thi	TRUE	reported
0.00639008	3.86131E-12	Thickness betwee	ISOS_RPE_thi	TRUE	reported
0.00714779	8.25686E-22	Thickness betwee	ISOS_RPE_thi	TRUE	reported
0.00758164	1.97961E-20	Thickness betwee	ISOS_RPE_thi	TRUE	reported
0.00639378	5.54683E-22	Thickness betwee	ISOS_RPE_thi	TRUE	reported
0.00769581	1.41721E-08	Thickness betwee	ISOS_RPE_thi	TRUE	reported
0.00645731	3.23746E-12	Thickness betwee	ISOS_RPE_thi	TRUE	reported
0.00689449	9.26896E-10	Thickness betwee	ISOS_RPE_thi	TRUE	reported
0.00646981	8.1795E-15	Thickness betwee	ISOS_RPE_thi	TRUE	reported

0.0108522	1.87035E-12	Thickness betwee	ISOS_RPE_thi	TRUE	reported
0.0146164	2.57156E-44	Thickness betwee	ISOS_RPE_thi	TRUE	reported
0.00648613	5.5448E-09	Thickness betwee	ISOS_RPE_thi	TRUE	reported
0.0114568	4.24734E-09	Thickness betwee	ISOS_RPE_thi	TRUE	reported
0.00774144	4.32794E-17	Thickness betwee	ISOS_RPE_thi	TRUE	reported
0.00995232	1.93662E-24	Thickness betwee	ISOS_RPE_thi	TRUE	reported
0.00733469	4.59809E-09	Thickness betwee	ISOS_RPE_thi	TRUE	reported
0.0203634	1.71628E-11	Thickness betwee	ISOS_RPE_thi	TRUE	reported
0.0204813	2.02061E-11	Thickness betwee	ISOS_RPE_thi	TRUE	reported
0.00687138	2.68646E-09	Thickness betwee	ISOS_RPE_thi	TRUE	reported
0.00656228	7.0456E-20	Thickness betwee	ISOS_RPE_thi	TRUE	reported
0.00975004	6.63133E-11	Thickness betwee	ISOS_RPE_thi	TRUE	reported
0.00697096	4.5798E-27	Thickness betwee	ISOS_RPE_thi	TRUE	reported
0.00827169	2.85352E-15	Thickness betwee	ISOS_RPE_thi	TRUE	reported
0.00681502	1.67553E-14	Thickness betwee	ISOS_RPE_thi	TRUE	reported
0.0099423	9.15293E-10	Thickness betwee	ISOS_RPE_thi	TRUE	reported
0.00779043	3.73343E-15	Thickness betwee	ISOS_RPE_thi	TRUE	reported
0.00954787	4.1985E-15	Thickness betwee	ISOS_RPE_thi	TRUE	reported
0.00746804	2.18875E-08	Thickness betwee	ISOS_RPE_thi	TRUE	reported
0.00765856	6.96313E-12	Thickness betwee	ISOS_RPE_thi	TRUE	reported
0.00735348	1.74439E-08	Thickness betwee	ISOS_RPE_thi	TRUE	reported
0.00630512	4.56206E-56	Thickness betwee	ISOS_RPE_thi	TRUE	reported
0.00791341	3.52349E-16	Thickness betwee	ISOS_RPE_thi	TRUE	reported
0.00634608	4.80318E-09	Thickness betwee	ISOS_RPE_thi	TRUE	reported
0.00825853	1.39155E-10	Thickness betwee	ISOS_RPE_thi	TRUE	reported
0.00769444	2.84923E-19	Thickness betwee	ISOS_RPE_thi	TRUE	reported
0.00672508	7.6471E-26	Thickness betwee	ISOS_RPE_thi	TRUE	reported
0.00634354	1.40182E-20	Thickness betwee	ISOS_RPE_thi	TRUE	reported
0.00738082	1.02138E-36	Thickness betwee	ISOS_RPE_thi	TRUE	reported
0.00689821	2.6916E-12	Thickness betwee	ISOS_RPE_thi	TRUE	reported
0.00634564	7.96809E-24	Thickness betwee	ISOS_RPE_thi	TRUE	reported
0.00691541	1.04777E-11	Thickness betwee	ISOS_RPE_thi	TRUE	reported
0.0113569	5.84873E-25	Thickness betwee	ISOS_RPE_thi	TRUE	reported
0.00843455	1.24959E-16	Thickness betwee	ISOS_RPE_thi	TRUE	reported
0.00773434	1.52637E-10	Thickness betwee	ISOS_RPE_thi	TRUE	reported
0.00642633	2.03129E-41	Thickness betwee	ISOS_RPE_thi	TRUE	reported
0.00656179	6.47179E-09	Thickness betwee	ISOS_RPE_thi	TRUE	reported
0.0183912	1.64159E-22	Thickness betwee	ISOS_RPE_thi	TRUE	reported
0.00802278	6.32595E-10	Thickness betwee	ISOS_RPE_thi	TRUE	reported
0.0133909	1.83015E-12	Thickness betwee	ISOS_RPE_thi	TRUE	reported
0.00635147	2.17912E-38	Thickness betwee	ISOS_RPE_thi	TRUE	reported
0.00676241	5.01258E-16	Thickness betwee	ISOS_RPE_thi	TRUE	reported
0.00626975	4.07154E-29	Thickness betwee	ISOS_RPE_thi	TRUE	reported
0.00635718	1.60107E-13	Thickness betwee	ISOS_RPE_thi	TRUE	reported

0.00625406	1.12457E-11	Thickness betwee	INL_ELM_thic	TRUE	reported
0.00646951	5.08538E-26	Thickness betwee	INL_ELM_thic	TRUE	reported
0.00963179	5.35752E-09	Thickness betwee	INL_ELM_thic	TRUE	reported
0.00652005	4.7881E-08	Thickness betwee	INL_ELM_thic	TRUE	reported
0.00662468	3.81469E-17	Thickness betwee	INL_ELM_thic	TRUE	reported
0.00639619	8.58118E-12	Thickness betwee	INL_ELM_thic	TRUE	reported
0.0244722	5.37691E-37	Thickness betwee	INL_ELM_thic	TRUE	reported
0.00867613	3.19563E-23	Thickness betwee	INL_ELM_thic	TRUE	reported
0.00712435	3.84585E-08	Thickness betwee	INL_ELM_thic	TRUE	reported
0.00917697	8.34601E-10	Thickness betwee	INL_ELM_thic	TRUE	reported
0.0174444	3.3589E-25	Thickness betwee	INL_ELM_thic	TRUE	reported
0.0124016	1.32442E-16	Thickness betwee	INL_ELM_thic	TRUE	reported
0.00627586	2.04801E-09	Thickness betwee	INL_ELM_thic	TRUE	reported
0.00632783	5.20385E-11	Thickness betwee	INL_ELM_thic	TRUE	reported
0.00770377	4.15002E-10	Thickness betwee	INL_ELM_thic	TRUE	reported
0.00876218	4.65529E-15	Thickness betwee	INL_ELM_thic	TRUE	reported
0.00944865	1.50419E-08	Thickness betwee	INL_ELM_thic	TRUE	reported
0.0138289	3.43581E-08	Thickness betwee	INL_ELM_thic	TRUE	reported
0.00692878	5.28424E-12	Thickness betwee	INL_ELM_thic	TRUE	reported
0.00669599	4.22931E-08	Thickness betwee	INL_ELM_thic	TRUE	reported
0.00641729	3.66435E-10	Thickness betwee	INL_ELM_thic	TRUE	reported
0.00630203	3.05043E-09	Thickness betwee	INL_ELM_thic	TRUE	reported
0.00660721	6.91873E-14	Thickness betwee	INL_ELM_thic	TRUE	reported
0.00658614	7.82008E-10	Thickness betwee	INL_ELM_thic	TRUE	reported
0.0120822	7.61117E-48	Thickness betwee	INL_ELM_thic	TRUE	reported
0.00642492	1.89715E-09	Thickness betwee	INL_ELM_thic	TRUE	reported
0.00780459	1.29495E-14	Thickness betwee	INL_ELM_thic	TRUE	reported
0.00625577	1.95771E-10	Thickness betwee	INL_ELM_thic	TRUE	reported
0.00644591	9.70894E-10	Thickness betwee	INL_ELM_thic	TRUE	reported
0.0143788	1.96704E-09	Thickness betwee	INL_ELM_thic	TRUE	reported
0.00765014	7.2531E-15	Thickness betwee	INL_ELM_thic	TRUE	reported
0.00827622	6.69276E-09	Thickness betwee	INL_ELM_thic	TRUE	reported
0.00627327	3.21329E-09	Thickness betwee	INL_ELM_thic	TRUE	reported
0.0171078	6.79225E-24	Thickness betwee	INL_ELM_thic	TRUE	reported
0.0106202	8.35895E-12	Thickness betwee	INL_ELM_thic	TRUE	reported
0.00828766	9.9464E-14	Thickness betwee	INL_ELM_thic	TRUE	reported
0.00966027	3.64528E-11	Thickness betwee	INL_ELM_thic	TRUE	reported
0.00677944	2.04104E-08	Thickness betwee	INL_ELM_thic	TRUE	reported
0.00703725	1.00169E-17	Thickness betwee	INL_ELM_thic	TRUE	reported
0.00632811	8.6316E-13	Thickness betwee	INL_ELM_thic	TRUE	reported
0.00768778	1.42965E-08	Thickness betwee	INL_ELM_thic	TRUE	reported
0.00913902	4.71509E-08	Thickness betwee	INL_ELM_thic	TRUE	reported
0.0209596	8.36965E-10	Thickness betwee	INL_ELM_thic	TRUE	reported
0.0090434	3.87707E-13	Thickness betwee	INL_ELM_thic	TRUE	reported

0.00686949	8.5734E-11	Thickness betwee	INL_ELM_thic	TRUE	reported
0.00720741	1.87409E-64	Thickness betwee	INL_ELM_thic	TRUE	reported
0.0068444	4.69531E-22	Thickness betwee	INL_ELM_thic	TRUE	reported
0.00647979	4.71102E-14	Thickness betwee	INL_ELM_thic	TRUE	reported
0.00657689	4.9721E-12	Thickness betwee	INL_ELM_thic	TRUE	reported
0.0073903	1.06555E-20	Thickness betwee	INL_ELM_thic	TRUE	reported
0.00670214	9.29327E-11	Thickness betwee	INL_ELM_thic	TRUE	reported
0.00694925	8.93516E-16	Thickness betwee	INL_ELM_thic	TRUE	reported
0.0154362	6.28002E-24	Thickness betwee	INL_ELM_thic	TRUE	reported
0.00812831	2.00621E-15	Thickness betwee	INL_ELM_thic	TRUE	reported
0.00627587	1.66319E-08	Thickness betwee	INL_ELM_thic	TRUE	reported
0.00627602	7.10736E-09	Thickness betwee	INL_ELM_thic	TRUE	reported
0.0063902	3.62402E-10	Thickness betwee	INL_ELM_thic	TRUE	reported
0.00732611	1.0226E-118	Thickness betwee	INL_ELM_thic	TRUE	reported
0.00764292	8.38747E-18	Thickness betwee	INL_ELM_thic	TRUE	reported
0.00627875	1.666E-08	Thickness betwee	INL_ELM_thic	TRUE	reported
0.00676944	4.95041E-10	Thickness betwee	INL_ELM_thic	TRUE	reported
0.00870253	1.45051E-10	Thickness betwee	INL_ELM_thic	TRUE	reported
0.00638236	1.0328E-08	Thickness betwee	INL_ELM_thic	TRUE	reported
0.00628898	5.99621E-09	Thickness betwee	INL_ELM_thic	TRUE	reported
0.00956231	2.56773E-09	Thickness betwee	INL_ELM_thic	TRUE	reported
0.0161278	1.53265E-15	Thickness betwee	INL_ELM_thic	TRUE	reported
0.00820662	2.9449E-279	Thickness betwee	INL_ELM_thic	TRUE	reported
0.0188172	4.68587E-46	Thickness betwee	INL_ELM_thic	TRUE	reported
0.0062896	3.8391E-21	Thickness betwee	INL_ELM_thic	TRUE	reported
0.00625032	7.14594E-10	Thickness betwee	INL_ELM_thic	TRUE	reported
0.00625737	2.56659E-13	Thickness betwee	INL_ELM_thic	TRUE	reported
0.00646912	3.77636E-13	Thickness betwee	INL_ELM_thic	TRUE	reported
0.00681198	7.36653E-12	Thickness betwee	INL_ELM_thic	TRUE	reported
0.00626664	3.88861E-19	Thickness betwee	INL_ELM_thic	TRUE	reported
0.00696014	1.62094E-11	Thickness betwee	INL_ELM_thic	TRUE	reported
0.00699031	2.09042E-09	Thickness betwee	INL_ELM_thic	TRUE	reported
0.0075594	2.61442E-25	Thickness betwee	INL_ELM_thic	TRUE	reported
0.00893184	4.29231E-10	Thickness betwee	INL_ELM_thic	TRUE	reported
0.00755814	3.52997E-09	Thickness betwee	INL_ELM_thic	TRUE	reported
0.00682604	7.42164E-22	Thickness betwee	INL_ELM_thic	TRUE	reported
0.00819431	4.68457E-10	Thickness betwee	INL_ELM_thic	TRUE	reported
0.00698371	8.95172E-25	Thickness betwee	INL_ELM_thic	TRUE	reported
0.00936202	7.7692E-14	Thickness betwee	INL_ELM_thic	TRUE	reported
0.0210377	1.87846E-13	Thickness betwee	INL_ELM_thic	TRUE	reported
0.00682323	4.4586E-08	Thickness betwee	INL_ELM_thic	TRUE	reported
0.00857597	4.08128E-08	Thickness betwee	INL_ELM_thic	TRUE	reported
0.00624969	1.00649E-14	Thickness betwee	INL_ELM_thic	TRUE	reported
0.00693086	6.53739E-26	Thickness betwee	INL_ELM_thic	TRUE	reported

0.00676636	4.11105E-09	Thickness betwee	INL_ELM_thic	TRUE	reported
0.0133277	2.75741E-08	Mean of vertical d	mean_of_ver	TRUE	reported
0.00839692	6.56774E-66	Mean of vertical d	mean_of_ver	TRUE	reported
0.00778519	1.87603E-15	Mean of vertical d	mean_of_ver	TRUE	reported
0.0123552	8.71929E-24	Mean of vertical d	mean_of_ver	TRUE	reported
0.0297273	7.61463E-10	Mean of vertical d	mean_of_ver	TRUE	reported
0.00669591	3.99953E-16	Mean of vertical d	mean_of_ver	TRUE	reported
0.00857142	9.30236E-14	Mean of vertical d	mean_of_ver	TRUE	reported
0.0206566	2.3528E-09	Mean of vertical d	mean_of_ver	TRUE	reported
0.00691816	4.78284E-10	Mean of vertical d	mean_of_ver	TRUE	reported
0.010962	1.18442E-12	Mean of vertical d	mean_of_ver	TRUE	reported
0.0118597	7.4641E-11	Mean of vertical d	mean_of_ver	TRUE	reported
0.00905279	6.19175E-09	Mean of vertical d	mean_of_ver	TRUE	reported
0.00683349	4.46445E-10	Mean of vertical d	mean_of_ver	TRUE	reported
0.00687342	7.76813E-10	Mean of vertical d	mean_of_ver	TRUE	reported
0.00663403	2.9979E-10	Mean of vertical d	mean_of_ver	TRUE	reported
0.00991333	4.38666E-08	Mean of vertical d	mean_of_ver	TRUE	reported
0.00671593	1.32875E-08	Mean of vertical d	mean_of_ver	TRUE	reported
0.00696568	1.25542E-09	Mean of vertical d	mean_of_ver	TRUE	reported
0.00743285	3.03219E-09	Mean of vertical d	mean_of_ver	TRUE	reported
0.00710034	2.01435E-09	Mean of vertical d	mean_of_ver	TRUE	reported
0.00780629	2.93831E-86	Mean of vertical d	mean_of_ver	TRUE	reported
0.00723943	1.48583E-09	Mean of vertical d	mean_of_ver	TRUE	reported
0.00782841	3.97667E-09	Mean of vertical d	mean_of_ver	TRUE	reported
0.0096321	6.88501E-13	Mean of vertical d	mean_of_ver	TRUE	reported
0.00664416	2.47525E-13	Mean of vertical d	mean_of_ver	TRUE	reported
0.00840878	1.9182E-09	Mean of vertical d	mean_of_ver	TRUE	reported
0.0302513	5.64158E-20	Mean of vertical d	mean_of_ver	TRUE	reported
0.00666686	1.90314E-11	Mean of vertical d	mean_of_ver	TRUE	reported
0.00718545	1.24111E-13	Mean of vertical d	mean_of_ver	TRUE	reported
0.0084716	7.13174E-14	Mean of vertical d	mean_of_ver	TRUE	reported
0.00688618	4.49139E-13	Mean of vertical d	mean_of_ver	TRUE	reported
0.00685063	5.36279E-17	Mean of vertical d	mean_of_ver	TRUE	reported
0.00763553	8.02329E-09	Mean of vertical d	mean_of_ver	TRUE	reported
0.00626717	1.40598E-24	Retinal pigment e	overall_avera	TRUE	reported
0.00632658	5.90982E-71	Retinal pigment e	overall_avera	TRUE	reported
0.0109248	9.30815E-12	Retinal pigment e	overall_avera	TRUE	reported
0.0303643	4.51894E-08	Retinal pigment e	overall_avera	TRUE	reported
0.0173814	4.44796E-25	Retinal pigment e	overall_avera	TRUE	reported
0.0144972	5.27571E-15	Retinal pigment e	overall_avera	TRUE	reported
0.00667108	7.52591E-10	Retinal pigment e	overall_avera	TRUE	reported
0.0109664	1.44262E-08	Retinal pigment e	overall_avera	TRUE	reported
0.00886636	4.4836E-58	Retinal pigment e	overall_avera	TRUE	reported
0.00918829	9.07213E-15	Retinal pigment e	overall_avera	TRUE	reported

0.00689764	4.09742E-25	Retinal pigment e|	overall_avera	TRUE	reported
0.00895028	2.58629E-08	Retinal pigment e|	overall_avera	TRUE	reported
0.00777032	2.76612E-09	Retinal pigment e|	overall_avera	TRUE	reported
0.00943351	3.8578E-49	Retinal pigment e|	overall_avera	TRUE	reported
0.00649203	7.91601E-09	Retinal pigment e|	overall_avera	TRUE	reported
0.00877562	4.68228E-08	Retinal pigment e|	overall_avera	TRUE	reported
0.00818585	5.01268E-21	Retinal pigment e|	overall_avera	TRUE	reported
0.00761996	1.51183E-10	Retinal pigment e|	overall_avera	TRUE	reported
0.00664902	1.59136E-91	Retinal pigment e|	overall_avera	TRUE	reported
0.00730489	2.43863E-09	Retinal pigment e|	overall_avera	TRUE	reported
0.00690973	1.87675E-08	Retinal pigment e|	overall_avera	TRUE	reported
0.0129346	4.68348E-14	Retinal pigment e|	overall_avera	TRUE	reported
0.0112318	4.57175E-72	Retinal pigment e|	overall_avera	TRUE	reported
0.0237446	2.14319E-10	Retinal pigment e|	overall_avera	TRUE	reported
0.0181754	7.45583E-12	Retinal pigment e|	overall_avera	TRUE	reported
0.0132509	9.1741E-10	Retinal pigment e|	overall_avera	TRUE	reported
0.0065257	1.91741E-45	Retinal pigment e|	overall_avera	TRUE	reported
0.00918398	3.97882E-09	Retinal pigment e|	overall_avera	TRUE	reported
0.0143711	4.25132E-08	Retinal pigment e|	overall_avera	TRUE	reported
0.00625206	3.07646E-24	Retinal pigment e|	overall_avera	TRUE	reported
0.00631356	1.03725E-69	Retinal pigment e|	overall_avera	TRUE	reported
0.0106492	7.79634E-15	Retinal pigment e|	overall_avera	TRUE	reported
0.0107102	1.72981E-19	Retinal pigment e|	overall_avera	TRUE	reported
0.0144785	1.0212E-14	Retinal pigment e|	overall_avera	TRUE	reported
0.0067261	1.26074E-08	Retinal pigment e|	overall_avera	TRUE	reported
0.0061946	1.88644E-08	Retinal pigment e|	overall_avera	TRUE	reported
0.00915442	7.87281E-45	Retinal pigment e|	overall_avera	TRUE	reported
0.00918541	2.87622E-16	Retinal pigment e|	overall_avera	TRUE	reported
0.00687835	2.23368E-23	Retinal pigment e|	overall_avera	TRUE	reported
0.00622978	1.61569E-11	Retinal pigment e|	overall_avera	TRUE	reported
0.0077701	1.41697E-09	Retinal pigment e|	overall_avera	TRUE	reported
0.00624814	1.32945E-08	Retinal pigment e|	overall_avera	TRUE	reported
0.0094216	6.96648E-49	Retinal pigment e|	overall_avera	TRUE	reported
0.024656	3.30027E-08	Retinal pigment e|	overall_avera	TRUE	reported
0.00647637	1.97258E-08	Retinal pigment e|	overall_avera	TRUE	reported
0.00815616	3.69453E-20	Retinal pigment e|	overall_avera	TRUE	reported
0.00759138	4.82248E-12	Retinal pigment e|	overall_avera	TRUE	reported
0.0127934	2.58092E-08	Retinal pigment e|	overall_avera	TRUE	reported
0.0066293	8.66637E-92	Retinal pigment e|	overall_avera	TRUE	reported
0.00791446	1.51596E-09	Retinal pigment e|	overall_avera	TRUE	reported
0.00728678	6.70047E-14	Retinal pigment e|	overall_avera	TRUE	reported
0.0128973	5.418E-13	Retinal pigment e|	overall_avera	TRUE	reported
0.011204	1.39181E-78	Retinal pigment e|	overall_avera	TRUE	reported
0.0236962	3.06854E-08	Retinal pigment e|	overall_avera	TRUE	reported

0.0102109	7.61266E-10	Retinal pigment e	overall_avera	TRUE	reported
0.00753348	7.9192E-11	Retinal pigment e	overall_avera	TRUE	reported
0.0132239	8.38894E-11	Retinal pigment e	overall_avera	TRUE	reported
0.00651031	1.86415E-49	Retinal pigment e	overall_avera	TRUE	reported
0.00741577	1.99406E-17	Overall macular th	overall_macu	TRUE	reported
0.00712953	4.93949E-10	Overall macular th	overall_macu	TRUE	reported
0.00656735	1.17344E-08	Overall macular th	overall_macu	TRUE	reported
0.00653301	2.50391E-13	Overall macular th	overall_macu	TRUE	reported
0.0117412	7.80381E-09	Overall macular th	overall_macu	TRUE	reported
0.00636508	4.76453E-08	Overall macular th	overall_macu	TRUE	reported
0.00854602	5.47518E-14	Overall macular th	overall_macu	TRUE	reported
0.00643708	5.17912E-09	Overall macular th	overall_macu	TRUE	reported
0.0123293	2.02592E-08	Overall macular th	overall_macu	TRUE	reported
0.00625726	5.72972E-16	Overall macular th	overall_macu	TRUE	reported
0.00700369	1.37174E-08	Overall macular th	overall_macu	TRUE	reported
0.0100296	2.29338E-08	Overall macular th	overall_macu	TRUE	reported
0.01004	1.65964E-26	Overall macular th	overall_macu	TRUE	reported
0.0073299	1.85898E-08	Overall macular th	overall_macu	TRUE	reported
0.00671096	1.18672E-09	Overall macular th	overall_macu	TRUE	reported
0.00631968	1.34317E-08	Overall macular th	overall_macu	TRUE	reported
0.00658711	3.90076E-08	Overall macular th	overall_macu	TRUE	reported
0.0148538	1.92143E-09	Overall macular th	overall_macu	TRUE	reported
0.0119232	2.71998E-86	Overall macular th	overall_macu	TRUE	reported
0.00639803	7.40454E-18	Overall macular th	overall_macu	TRUE	reported
0.00629913	8.42809E-16	Overall macular th	overall_macu	TRUE	reported
0.00648409	6.25795E-10	Overall macular th	overall_macu	TRUE	reported
0.00762168	1.88734E-15	Overall macular th	overall_macu	TRUE	reported
0.00645447	6.5783E-10	Overall macular th	overall_macu	TRUE	reported
0.0105895	5.29238E-11	Overall macular th	overall_macu	TRUE	reported
0.00663513	1.12078E-11	Overall macular th	overall_macu	TRUE	reported
0.00632354	1.43462E-08	Overall macular th	overall_macu	TRUE	reported
0.00920019	1.14542E-08	Overall macular th	overall_macu	TRUE	reported
0.00648344	1.04917E-08	Overall macular th	overall_macu	TRUE	reported
0.0063785	1.39884E-20	Overall macular th	overall_macu	TRUE	reported
0.00756269	2.82398E-12	Overall macular th	overall_macu	TRUE	reported
0.00687342	4.53023E-14	Overall macular th	overall_macu	TRUE	reported
0.00794322	1.55389E-08	Overall macular th	overall_macu	TRUE	reported
0.00643965	4.6287E-13	Overall macular th	overall_macu	TRUE	reported
0.00718055	2.03636E-12	Overall macular th	overall_macu	TRUE	reported
0.00686191	6.86187E-14	Overall macular th	overall_macu	TRUE	reported
0.00629523	2.83773E-09	Overall macular th	overall_macu	TRUE	reported
0.00654428	8.48113E-16	Overall macular th	overall_macu	TRUE	reported
0.00732565	4.2213E-09	Overall macular th	overall_macu	TRUE	reported
0.00686306	2.20489E-10	Overall macular th	overall_macu	TRUE	reported

0.00692335	4.32529E-12	Overall macular th	overall_macu	TRUE	reported
0.0153984	1.79992E-09	Overall macular th	overall_macu	TRUE	reported
0.00660178	4.43049E-09	Overall macular th	overall_macu	TRUE	reported
0.00750021	9.69812E-10	Overall macular th	overall_macu	TRUE	reported
0.00639023	1.87044E-11	Overall macular th	overall_macu	TRUE	reported
0.00729994	1.41392E-90	Overall macular th	overall_macu	TRUE	reported
0.00795185	2.42232E-15	Overall macular th	overall_macu	TRUE	reported
0.00680991	9.9824E-11	Overall macular th	overall_macu	TRUE	reported
0.00647771	1.85116E-09	Overall macular th	overall_macu	TRUE	reported
0.00679225	2.72927E-12	Overall macular th	overall_macu	TRUE	reported
0.00818198	4.15307E-15	Overall macular th	overall_macu	TRUE	reported
0.00626643	1.86232E-13	Overall macular th	overall_macu	TRUE	reported
0.00678124	5.91308E-09	Overall macular th	overall_macu	TRUE	reported
0.00623948	1.77467E-14	Overall macular th	overall_macu	TRUE	reported
0.00656779	5.27291E-09	Overall macular th	overall_macu	TRUE	reported
0.00663307	2.47689E-13	Overall macular th	overall_macu	TRUE	reported
0.0071057	6.90741E-14	Overall macular th	overall_macu	TRUE	reported
0.00744502	2.64214E-18	Overall macular th	overall_macu	TRUE	reported
0.00890581	6.97498E-14	Overall macular th	overall_macu	TRUE	reported
0.00753025	4.09493E-08	Overall macular th	overall_macu	TRUE	reported
0.00651956	1.74573E-50	Overall macular th	overall_macu	TRUE	reported
0.00677482	2.34972E-14	Overall macular th	overall_macu	TRUE	reported
0.00933209	1.60537E-09	Overall macular th	overall_macu	TRUE	reported
0.00631172	2.5743E-09	Overall macular th	overall_macu	TRUE	reported
0.00995049	9.94133E-10	Overall macular th	overall_macu	TRUE	reported
0.00674659	2.37964E-12	Overall macular th	overall_macu	TRUE	reported
0.00660508	2.89089E-08	Overall macular th	overall_macu	TRUE	reported
0.00741278	1.68655E-15	Overall macular th	overall_macu	TRUE	reported
0.00700537	1.53465E-08	Overall macular th	overall_macu	TRUE	reported
0.00631267	7.26509E-15	Overall macular th	overall_macu	TRUE	reported
0.0117524	7.50787E-09	Overall macular th	overall_macu	TRUE	reported
0.0063667	9.98953E-11	Overall macular th	overall_macu	TRUE	reported
0.00822814	6.82345E-12	Overall macular th	overall_macu	TRUE	reported
0.0123512	4.31374E-12	Overall macular th	overall_macu	TRUE	reported
0.00624379	1.76105E-19	Overall macular th	overall_macu	TRUE	reported
0.00700053	4.27319E-09	Overall macular th	overall_macu	TRUE	reported
0.0100349	2.20131E-25	Overall macular th	overall_macu	TRUE	reported
0.00661041	2.76479E-09	Overall macular th	overall_macu	TRUE	reported
0.00639396	2.20452E-11	Overall macular th	overall_macu	TRUE	reported
0.00658274	3.35485E-08	Overall macular th	overall_macu	TRUE	reported
0.0124959	4.68333E-15	Overall macular th	overall_macu	TRUE	reported
0.0119335	9.43381E-81	Overall macular th	overall_macu	TRUE	reported
0.00622372	4.55337E-15	Overall macular th	overall_macu	TRUE	reported
0.0143246	1.97727E-11	Overall macular th	overall_macu	TRUE	reported

0.00762046	1.68257E-13	Overall macular th	overall_macu	TRUE	reported
0.00695251	2.99962E-10	Overall macular th	overall_macu	TRUE	reported
0.0105759	1.76068E-11	Overall macular th	overall_macu	TRUE	reported
0.00663563	1.6167E-11	Overall macular th	overall_macu	TRUE	reported
0.00882117	1.59445E-09	Overall macular th	overall_macu	TRUE	reported
0.00652698	2.15766E-09	Overall macular th	overall_macu	TRUE	reported
0.00636772	1.26757E-16	Overall macular th	overall_macu	TRUE	reported
0.00756338	6.76633E-16	Overall macular th	overall_macu	TRUE	reported
0.00686876	1.48587E-10	Overall macular th	overall_macu	TRUE	reported
0.00789525	1.79905E-09	Overall macular th	overall_macu	TRUE	reported
0.0208564	2.56745E-09	Overall macular th	overall_macu	TRUE	reported
0.0063303	1.32664E-09	Overall macular th	overall_macu	TRUE	reported
0.00714657	4.47326E-09	Overall macular th	overall_macu	TRUE	reported
0.00717398	1.41076E-09	Overall macular th	overall_macu	TRUE	reported
0.0067085	9.14354E-16	Overall macular th	overall_macu	TRUE	reported
0.00768723	2.49692E-10	Overall macular th	overall_macu	TRUE	reported
0.00635284	9.89417E-10	Overall macular th	overall_macu	TRUE	reported
0.00950726	4.87511E-10	Overall macular th	overall_macu	TRUE	reported
0.00703112	4.20815E-08	Overall macular th	overall_macu	TRUE	reported
0.00655147	3.95176E-16	Overall macular th	overall_macu	TRUE	reported
0.00732237	1.19319E-09	Overall macular th	overall_macu	TRUE	reported
0.00685731	3.22428E-10	Overall macular th	overall_macu	TRUE	reported
0.00692115	5.29382E-14	Overall macular th	overall_macu	TRUE	reported
0.0153743	8.28963E-10	Overall macular th	overall_macu	TRUE	reported
0.00750499	1.17347E-11	Overall macular th	overall_macu	TRUE	reported
0.0072985	1.80483E-87	Overall macular th	overall_macu	TRUE	reported
0.0078953	5.2524E-14	Overall macular th	overall_macu	TRUE	reported
0.0072232	3.4612E-08	Overall macular th	overall_macu	TRUE	reported
0.00681017	1.50667E-11	Overall macular th	overall_macu	TRUE	reported
0.00643052	1.24908E-10	Overall macular th	overall_macu	TRUE	reported
0.00682889	8.29658E-15	Overall macular th	overall_macu	TRUE	reported
0.00817658	1.10643E-15	Overall macular th	overall_macu	TRUE	reported
0.00626489	7.55534E-14	Overall macular th	overall_macu	TRUE	reported
0.0112217	5.81287E-10	Overall macular th	overall_macu	TRUE	reported
0.00662558	2.26821E-12	Overall macular th	overall_macu	TRUE	reported
0.0062332	9.15706E-14	Overall macular th	overall_macu	TRUE	reported
0.00623419	2.86003E-12	Overall macular th	overall_macu	TRUE	reported
0.00656528	1.97173E-15	Overall macular th	overall_macu	TRUE	reported
0.00710325	7.6069E-14	Overall macular th	overall_macu	TRUE	reported
0.00738197	1.07105E-24	Overall macular th	overall_macu	TRUE	reported
0.008901	1.90242E-14	Overall macular th	overall_macu	TRUE	reported
0.00777403	1.83124E-11	Overall macular th	overall_macu	TRUE	reported
0.00651984	5.37681E-42	Overall macular th	overall_macu	TRUE	reported
0.00628372	3.31799E-08	Overall macular th	overall_macu	TRUE	reported

0.00694267	3.32967E-19	Overall macular th	overall_macu	TRUE	reported
0.00932695	6.17238E-13	Overall macular th	overall_macu	TRUE	reported
0.00630882	1.92981E-08	Overall macular th	overall_macu	TRUE	reported
0.00636507	2.23898E-09	Overall macular th	overall_macu	TRUE	reported
0.0112124	4.84422E-09	Overall macular th	overall_macu	TRUE	reported
0.0114583	2.14093E-11	Overall macular th	overall_macu	TRUE	reported
0.00671021	2.93436E-13	Overall macular th	overall_macu	TRUE	reported
0.0136293	5.83131E-11	Vertical cup to dis	VCDR_left	TRUE	reported
0.00679058	2.61865E-09	Vertical cup to dis	VCDR_left	TRUE	reported
0.0068634	4.27631E-10	Vertical cup to dis	VCDR_left	TRUE	reported
0.0085558	6.01041E-39	Vertical cup to dis	VCDR_left	TRUE	reported
0.00741131	9.98833E-12	Vertical cup to dis	VCDR_left	TRUE	reported
0.00673042	8.25985E-12	Vertical cup to dis	VCDR_left	TRUE	reported
0.00802568	3.16011E-08	Vertical cup to dis	VCDR_left	TRUE	reported
0.00702954	6.66188E-13	Vertical cup to dis	VCDR_left	TRUE	reported
0.0090975	6.01032E-11	Vertical cup to dis	VCDR_left	TRUE	reported
0.00816919	1.05396E-08	Vertical cup to dis	VCDR_left	TRUE	reported
0.0111772	4.9648E-11	Vertical cup to dis	VCDR_left	TRUE	reported
0.00687951	4.05327E-10	Vertical cup to dis	VCDR_left	TRUE	reported
0.00684835	6.40905E-13	Vertical cup to dis	VCDR_left	TRUE	reported
0.0102389	1.31877E-08	Vertical cup to dis	VCDR_left	TRUE	reported
0.00681856	5.74056E-35	Vertical cup to dis	VCDR_left	TRUE	reported
0.00792779	1.44573E-46	Vertical cup to dis	VCDR_left	TRUE	reported
0.00712432	7.63568E-10	Vertical cup to dis	VCDR_left	TRUE	reported
0.00756679	1.34066E-09	Vertical cup to dis	VCDR_left	TRUE	reported
0.00836524	1.68102E-09	Vertical cup to dis	VCDR_left	TRUE	reported
0.00711122	7.52063E-14	Vertical cup to dis	VCDR_left	TRUE	reported
0.00675193	1.91079E-47	Vertical cup to dis	VCDR_left	TRUE	reported
0.00784672	3.76129E-08	Vertical cup to dis	VCDR_left	TRUE	reported
0.00760027	3.3437E-10	Vertical cup to dis	VCDR_left	TRUE	reported
0.00694344	2.61275E-08	Vertical cup to dis	VCDR_left	TRUE	reported
0.00737684	2.36401E-10	Vertical cup to dis	VCDR_left	TRUE	reported
0.00899211	1.02047E-09	Vertical cup to dis	VCDR_left	TRUE	reported
0.00735335	1.60021E-26	Vertical cup to dis	VCDR_left	TRUE	reported
0.00685949	1.54757E-09	Vertical cup to dis	VCDR_left	TRUE	reported
0.0152577	2.73092E-08	Vertical cup to dis	VCDR_left	TRUE	reported
0.00717652	1.75146E-08	Vertical cup to dis	VCDR_left	TRUE	reported
0.00697669	1.13787E-22	Vertical cup to dis	VCDR_left	TRUE	reported
0.00698387	3.30751E-34	Vertical cup to dis	VCDR_left	TRUE	reported
0.00673149	1.30443E-10	Vertical cup to dis	VCDR_left	TRUE	reported
0.00777232	3.90026E-12	Vertical cup to dis	VCDR_left	TRUE	reported
0.0136351	1.39864E-11	Vertical cup to dis	VCDR_regres	TRUE	reported
0.00679347	8.93927E-09	Vertical cup to dis	VCDR_regres	TRUE	reported
0.00686632	3.4578E-10	Vertical cup to dis	VCDR_regres	TRUE	reported

0.00855944	1.13748E-41	Vertical cup to dis	VCDR_regress	TRUE	reported
0.00741446	2.45705E-12	Vertical cup to dis	VCDR_regress	TRUE	reported
0.00673328	8.91909E-12	Vertical cup to dis	VCDR_regress	TRUE	reported
0.00731049	7.45333E-09	Vertical cup to dis	VCDR_regress	TRUE	reported
0.00703252	2.71682E-12	Vertical cup to dis	VCDR_regress	TRUE	reported
0.00910137	1.15859E-11	Vertical cup to dis	VCDR_regress	TRUE	reported
0.00817375	1.44212E-08	Vertical cup to dis	VCDR_regress	TRUE	reported
0.011182	1.75984E-12	Vertical cup to dis	VCDR_regress	TRUE	reported
0.00939503	3.39803E-08	Vertical cup to dis	VCDR_regress	TRUE	reported
0.00993982	6.29177E-10	Vertical cup to dis	VCDR_regress	TRUE	reported
0.00677936	2.02692E-09	Vertical cup to dis	VCDR_regress	TRUE	reported
0.00685126	2.45533E-13	Vertical cup to dis	VCDR_regress	TRUE	reported
0.00682146	4.83255E-37	Vertical cup to dis	VCDR_regress	TRUE	reported
0.00793116	1.45143E-48	Vertical cup to dis	VCDR_regress	TRUE	reported
0.00712734	6.15062E-10	Vertical cup to dis	VCDR_regress	TRUE	reported
0.00757001	6.12524E-09	Vertical cup to dis	VCDR_regress	TRUE	reported
0.00840998	4.14676E-10	Vertical cup to dis	VCDR_regress	TRUE	reported
0.0069328	1.10598E-13	Vertical cup to dis	VCDR_regress	TRUE	reported
0.0067548	1.60921E-50	Vertical cup to dis	VCDR_regress	TRUE	reported
0.00789688	1.29243E-08	Vertical cup to dis	VCDR_regress	TRUE	reported
0.0076035	1.83233E-10	Vertical cup to dis	VCDR_regress	TRUE	reported
0.0069464	1.58358E-09	Vertical cup to dis	VCDR_regress	TRUE	reported
0.00737998	7.98112E-10	Vertical cup to dis	VCDR_regress	TRUE	reported
0.00899593	8.12914E-10	Vertical cup to dis	VCDR_regress	TRUE	reported
0.00735647	1.46701E-27	Vertical cup to dis	VCDR_regress	TRUE	reported
0.00686241	1.89408E-10	Vertical cup to dis	VCDR_regress	TRUE	reported
0.0117765	2.80545E-08	Vertical cup to dis	VCDR_regress	TRUE	reported
0.00717957	8.13746E-09	Vertical cup to dis	VCDR_regress	TRUE	reported
0.00697965	5.64574E-23	Vertical cup to dis	VCDR_regress	TRUE	reported
0.00698684	1.68687E-35	Vertical cup to dis	VCDR_regress	TRUE	reported
0.00673435	4.79902E-11	Vertical cup to dis	VCDR_regress	TRUE	reported
0.00778922	7.7834E-14	Vertical cup to dis	VCDR_regress	TRUE	reported
0.00731697	2.40245E-08	Vertical cup to dis	VCDR_regress	TRUE	reported
0.0065942	4.18314E-09	Thickness betwee	INL_RPE_thic	TRUE	reported
0.00715778	1.22246E-09	Thickness betwee	INL_RPE_thic	TRUE	reported
0.00751496	5.8898E-22	Thickness betwee	INL_RPE_thic	TRUE	reported
0.00719529	7.37735E-09	Thickness betwee	INL_RPE_thic	TRUE	reported
0.00636274	8.8087E-11	Thickness betwee	INL_RPE_thic	TRUE	reported
0.00649261	3.79147E-15	Thickness betwee	INL_RPE_thic	TRUE	reported
0.00967339	7.12902E-11	Thickness betwee	INL_RPE_thic	TRUE	reported
0.00665003	7.53323E-13	Thickness betwee	INL_RPE_thic	TRUE	reported
0.00630072	1.76486E-24	Thickness betwee	INL_RPE_thic	TRUE	reported
0.00655859	7.03199E-11	Thickness betwee	INL_RPE_thic	TRUE	reported
0.0245591	6.92774E-31	Thickness betwee	INL_RPE_thic	TRUE	reported

0.00828458	7.22782E-37	Thickness betwee	INL_RPE_thic	TRUE	reported
0.00732938	9.96354E-11	Thickness betwee	INL_RPE_thic	TRUE	reported
0.0124052	9.86585E-15	Thickness betwee	INL_RPE_thic	TRUE	reported
0.00630684	1.01981E-08	Thickness betwee	INL_RPE_thic	TRUE	reported
0.0143753	1.75061E-18	Thickness betwee	INL_RPE_thic	TRUE	reported
0.00695705	1.9379E-12	Thickness betwee	INL_RPE_thic	TRUE	reported
0.00734746	3.25655E-10	Thickness betwee	INL_RPE_thic	TRUE	reported
0.00695491	1.02903E-11	Thickness betwee	INL_RPE_thic	TRUE	reported
0.00701625	3.19304E-09	Thickness betwee	INL_RPE_thic	TRUE	reported
0.0105983	1.87224E-08	Thickness betwee	INL_RPE_thic	TRUE	reported
0.00632099	6.44179E-11	Thickness betwee	INL_RPE_thic	TRUE	reported
0.00663308	3.70793E-17	Thickness betwee	INL_RPE_thic	TRUE	reported
0.00660644	4.03421E-09	Thickness betwee	INL_RPE_thic	TRUE	reported
0.0149565	1.09067E-08	Thickness betwee	INL_RPE_thic	TRUE	reported
0.0119959	3.59368E-51	Thickness betwee	INL_RPE_thic	TRUE	reported
0.00644217	9.24297E-16	Thickness betwee	INL_RPE_thic	TRUE	reported
0.00783038	4.45123E-15	Thickness betwee	INL_RPE_thic	TRUE	reported
0.00628354	4.53538E-11	Thickness betwee	INL_RPE_thic	TRUE	reported
0.00653219	5.34981E-14	Thickness betwee	INL_RPE_thic	TRUE	reported
0.00767408	9.77309E-22	Thickness betwee	INL_RPE_thic	TRUE	reported
0.0171981	1.4737E-21	Thickness betwee	INL_RPE_thic	TRUE	reported
0.00668226	4.75554E-08	Thickness betwee	INL_RPE_thic	TRUE	reported
0.00827198	1.45649E-12	Thickness betwee	INL_RPE_thic	TRUE	reported
0.00968604	2.38068E-11	Thickness betwee	INL_RPE_thic	TRUE	reported
0.0070578	3.44295E-15	Thickness betwee	INL_RPE_thic	TRUE	reported
0.00795496	1.3497E-17	Thickness betwee	INL_RPE_thic	TRUE	reported
0.0091677	2.89619E-13	Thickness betwee	INL_RPE_thic	TRUE	reported
0.0209907	5.12207E-11	Thickness betwee	INL_RPE_thic	TRUE	reported
0.00914344	4.28879E-08	Thickness betwee	INL_RPE_thic	TRUE	reported
0.00916591	2.97333E-16	Thickness betwee	INL_RPE_thic	TRUE	reported
0.00688501	4.00206E-11	Thickness betwee	INL_RPE_thic	TRUE	reported
0.00721696	1.39606E-75	Thickness betwee	INL_RPE_thic	TRUE	reported
0.0084348	1.22711E-10	Thickness betwee	INL_RPE_thic	TRUE	reported
0.00683673	1.72482E-16	Thickness betwee	INL_RPE_thic	TRUE	reported
0.00630552	1.82721E-10	Thickness betwee	INL_RPE_thic	TRUE	reported
0.00660617	1.62728E-13	Thickness betwee	INL_RPE_thic	TRUE	reported
0.00738741	9.59782E-25	Thickness betwee	INL_RPE_thic	TRUE	reported
0.00628942	4.58209E-20	Thickness betwee	INL_RPE_thic	TRUE	reported
0.00678433	5.11927E-10	Thickness betwee	INL_RPE_thic	TRUE	reported
0.00890333	4.93412E-11	Thickness betwee	INL_RPE_thic	TRUE	reported
0.00697038	1.81728E-12	Thickness betwee	INL_RPE_thic	TRUE	reported
0.013957	3.72867E-15	Thickness betwee	INL_RPE_thic	TRUE	reported
0.00815251	2.13204E-14	Thickness betwee	INL_RPE_thic	TRUE	reported
0.0298815	4.89612E-08	Thickness betwee	INL_RPE_thic	TRUE	reported

0.00631956	3.73339E-11	Thickness betwee	INL_RPE_thic	TRUE	reported
0.00635334	1.20634E-11	Thickness betwee	INL_RPE_thic	TRUE	reported
0.00666158	7.89296E-12	Thickness betwee	INL_RPE_thic	TRUE	reported
0.00735071	8.6412E-180	Thickness betwee	INL_RPE_thic	TRUE	reported
0.00795425	8.66982E-20	Thickness betwee	INL_RPE_thic	TRUE	reported
0.00684882	5.35347E-09	Thickness betwee	INL_RPE_thic	TRUE	reported
0.00828726	2.8632E-08	Thickness betwee	INL_RPE_thic	TRUE	reported
0.00684241	1.08861E-18	Thickness betwee	INL_RPE_thic	TRUE	reported
0.00855325	1.25517E-11	Thickness betwee	INL_RPE_thic	TRUE	reported
0.00636692	2.15395E-10	Thickness betwee	INL_RPE_thic	TRUE	reported
0.0063171	3.40927E-18	Thickness betwee	INL_RPE_thic	TRUE	reported
0.00631372	6.2295E-10	Thickness betwee	INL_RPE_thic	TRUE	reported
0.00958556	2.29818E-09	Thickness betwee	INL_RPE_thic	TRUE	reported
0.0161625	4.96543E-14	Thickness betwee	INL_RPE_thic	TRUE	reported
0.00823961	1.8645E-238	Thickness betwee	INL_RPE_thic	TRUE	reported
0.0188897	2.47105E-45	Thickness betwee	INL_RPE_thic	TRUE	reported
0.00630925	8.57847E-20	Thickness betwee	INL_RPE_thic	TRUE	reported
0.00689361	2.91896E-09	Thickness betwee	INL_RPE_thic	TRUE	reported
0.00628084	1.42423E-17	Thickness betwee	INL_RPE_thic	TRUE	reported
0.00737905	4.81346E-10	Thickness betwee	INL_RPE_thic	TRUE	reported
0.00629073	3.16152E-12	Thickness betwee	INL_RPE_thic	TRUE	reported
0.00843171	4.09944E-11	Thickness betwee	INL_RPE_thic	TRUE	reported
0.0071508	1.48831E-13	Thickness betwee	INL_RPE_thic	TRUE	reported
0.00682301	3.7745E-11	Thickness betwee	INL_RPE_thic	TRUE	reported
0.00798596	8.02043E-13	Thickness betwee	INL_RPE_thic	TRUE	reported
0.00762203	1.41695E-21	Thickness betwee	INL_RPE_thic	TRUE	reported
0.00896576	8.30266E-13	Thickness betwee	INL_RPE_thic	TRUE	reported
0.00678253	2.04593E-13	Thickness betwee	INL_RPE_thic	TRUE	reported
0.00714085	1.12379E-21	Thickness betwee	INL_RPE_thic	TRUE	reported
0.0110311	2.15044E-10	Thickness betwee	INL_RPE_thic	TRUE	reported
0.00627401	1.29611E-13	Thickness betwee	INL_RPE_thic	TRUE	reported
0.00635066	1.29511E-16	Thickness betwee	INL_RPE_thic	TRUE	reported
0.00683557	1.50576E-08	Thickness betwee	INL_RPE_thic	TRUE	reported
0.00696081	5.88754E-25	Thickness betwee	INL_RPE_thic	TRUE	reported
0.00678761	9.63549E-10	Thickness betwee	INL_RPE_thic	TRUE	reported
0.00626544	2.05307E-11	Thickness betwee	INL_RPE_thic	TRUE	reported
0.0069819	1.36244E-08	Thickness betwee	INL_RPE_thic	TRUE	reported
0.00750255	6.16404E-22	Thickness betwee	INL_RPE_thic	TRUE	reported
0.00713643	2.16524E-08	Thickness betwee	INL_RPE_thic	TRUE	reported
0.0081951	4.74983E-11	Thickness betwee	INL_RPE_thic	TRUE	reported
0.00645392	6.29366E-20	Thickness betwee	INL_RPE_thic	TRUE	reported
0.0080466	3.40263E-10	Thickness betwee	INL_RPE_thic	TRUE	reported
0.00660845	2.6523E-18	Thickness betwee	INL_RPE_thic	TRUE	reported
0.00626707	1.99607E-25	Thickness betwee	INL_RPE_thic	TRUE	reported

0.00652026	4.43292E-10	Thickness betwee	INL_RPE_thic	TRUE	reported
0.0244113	7.70776E-36	Thickness betwee	INL_RPE_thic	TRUE	reported
0.00860042	6.80207E-32	Thickness betwee	INL_RPE_thic	TRUE	reported
0.00728903	6.50519E-09	Thickness betwee	INL_RPE_thic	TRUE	reported
0.0123699	4.70304E-15	Thickness betwee	INL_RPE_thic	TRUE	reported
0.00626054	3.30523E-09	Thickness betwee	INL_RPE_thic	TRUE	reported
0.0143153	2.76942E-16	Thickness betwee	INL_RPE_thic	TRUE	reported
0.00691407	3.31508E-12	Thickness betwee	INL_RPE_thic	TRUE	reported
0.00731274	8.09064E-09	Thickness betwee	INL_RPE_thic	TRUE	reported
0.00907211	2.75579E-09	Thickness betwee	INL_RPE_thic	TRUE	reported
0.00942412	3.52558E-08	Thickness betwee	INL_RPE_thic	TRUE	reported
0.00691049	1.59858E-12	Thickness betwee	INL_RPE_thic	TRUE	reported
0.00697763	2.75713E-08	Thickness betwee	INL_RPE_thic	TRUE	reported
0.00640128	4.80588E-08	Thickness betwee	INL_RPE_thic	TRUE	reported
0.00758369	4.65765E-08	Thickness betwee	INL_RPE_thic	TRUE	reported
0.00626819	2.19361E-11	Thickness betwee	INL_RPE_thic	TRUE	reported
0.00659156	8.83686E-16	Thickness betwee	INL_RPE_thic	TRUE	reported
0.00656956	2.70361E-10	Thickness betwee	INL_RPE_thic	TRUE	reported
0.0120548	5.16599E-50	Thickness betwee	INL_RPE_thic	TRUE	reported
0.00640853	1.72907E-13	Thickness betwee	INL_RPE_thic	TRUE	reported
0.00778531	1.3085E-14	Thickness betwee	INL_RPE_thic	TRUE	reported
0.00624023	3.17887E-11	Thickness betwee	INL_RPE_thic	TRUE	reported
0.00649602	1.86267E-10	Thickness betwee	INL_RPE_thic	TRUE	reported
0.00763029	2.03616E-21	Thickness betwee	INL_RPE_thic	TRUE	reported
0.0170628	2.31934E-22	Thickness betwee	INL_RPE_thic	TRUE	reported
0.00808711	5.58584E-11	Thickness betwee	INL_RPE_thic	TRUE	reported
0.00963124	2.9988E-12	Thickness betwee	INL_RPE_thic	TRUE	reported
0.006319	1.43377E-08	Thickness betwee	INL_RPE_thic	TRUE	reported
0.00701037	2.06836E-14	Thickness betwee	INL_RPE_thic	TRUE	reported
0.00929185	2.01918E-17	Thickness betwee	INL_RPE_thic	TRUE	reported
0.00911655	8.63124E-13	Thickness betwee	INL_RPE_thic	TRUE	reported
0.0209101	3.93183E-11	Thickness betwee	INL_RPE_thic	TRUE	reported
0.0071583	3.36313E-09	Thickness betwee	INL_RPE_thic	TRUE	reported
0.00910068	1.45123E-14	Thickness betwee	INL_RPE_thic	TRUE	reported
0.00685157	5.44327E-14	Thickness betwee	INL_RPE_thic	TRUE	reported
0.00719078	8.35475E-71	Thickness betwee	INL_RPE_thic	TRUE	reported
0.006717	1.74756E-17	Thickness betwee	INL_RPE_thic	TRUE	reported
0.00626891	2.65646E-12	Thickness betwee	INL_RPE_thic	TRUE	reported
0.00758119	4.30248E-08	Thickness betwee	INL_RPE_thic	TRUE	reported
0.00656109	2.03734E-12	Thickness betwee	INL_RPE_thic	TRUE	reported
0.00737221	3.56014E-25	Thickness betwee	INL_RPE_thic	TRUE	reported
0.00625426	2.34936E-19	Thickness betwee	INL_RPE_thic	TRUE	reported
0.00675303	2.18967E-09	Thickness betwee	INL_RPE_thic	TRUE	reported
0.00884628	1.3019E-11	Thickness betwee	INL_RPE_thic	TRUE	reported

0.00693153	2.67841E-12	Thickness betwee	INL_RPE_thic	TRUE	reported
0.0138592	1.46814E-17	Thickness betwee	INL_RPE_thic	TRUE	reported
0.00810878	7.70151E-14	Thickness betwee	INL_RPE_thic	TRUE	reported
0.0062741	4.15009E-12	Thickness betwee	INL_RPE_thic	TRUE	reported
0.00626015	1.24615E-13	Thickness betwee	INL_RPE_thic	TRUE	reported
0.00662502	1.83845E-12	Thickness betwee	INL_RPE_thic	TRUE	reported
0.00730903	4.4622E-176	Thickness betwee	INL_RPE_thic	TRUE	reported
0.00790654	8.56906E-20	Thickness betwee	INL_RPE_thic	TRUE	reported
0.00680624	5.48747E-09	Thickness betwee	INL_RPE_thic	TRUE	reported
0.00649006	2.21455E-08	Thickness betwee	INL_RPE_thic	TRUE	reported
0.00684885	1.73934E-18	Thickness betwee	INL_RPE_thic	TRUE	reported
0.00855272	5.44901E-10	Thickness betwee	INL_RPE_thic	TRUE	reported
0.00639386	1.09865E-08	Thickness betwee	INL_RPE_thic	TRUE	reported
0.00626866	1.53714E-18	Thickness betwee	INL_RPE_thic	TRUE	reported
0.00628077	6.75061E-09	Thickness betwee	INL_RPE_thic	TRUE	reported
0.00953748	1.53929E-12	Thickness betwee	INL_RPE_thic	TRUE	reported
0.00652716	4.9572E-08	Thickness betwee	INL_RPE_thic	TRUE	reported
0.0160905	3.20282E-14	Thickness betwee	INL_RPE_thic	TRUE	reported
0.00818654	4.7046E-254	Thickness betwee	INL_RPE_thic	TRUE	reported
0.0187741	2.13495E-44	Thickness betwee	INL_RPE_thic	TRUE	reported
0.00627799	6.47608E-20	Thickness betwee	INL_RPE_thic	TRUE	reported
0.00623322	6.39942E-11	Thickness betwee	INL_RPE_thic	TRUE	reported
0.00624153	6.85346E-18	Thickness betwee	INL_RPE_thic	TRUE	reported
0.00645354	7.19285E-11	Thickness betwee	INL_RPE_thic	TRUE	reported
0.00733626	3.81262E-09	Thickness betwee	INL_RPE_thic	TRUE	reported
0.00631465	3.68983E-11	Thickness betwee	INL_RPE_thic	TRUE	reported
0.00679608	1.70055E-09	Thickness betwee	INL_RPE_thic	TRUE	reported
0.00837759	2.36042E-12	Thickness betwee	INL_RPE_thic	TRUE	reported
0.00625312	2.1004E-15	Thickness betwee	INL_RPE_thic	TRUE	reported
0.00688674	4.14483E-14	Thickness betwee	INL_RPE_thic	TRUE	reported
0.00794117	1.60956E-14	Thickness betwee	INL_RPE_thic	TRUE	reported
0.00696643	8.59962E-09	Thickness betwee	INL_RPE_thic	TRUE	reported
0.00754132	9.78746E-24	Thickness betwee	INL_RPE_thic	TRUE	reported
0.00891239	6.79131E-10	Thickness betwee	INL_RPE_thic	TRUE	reported
0.00742243	3.88356E-13	Thickness betwee	INL_RPE_thic	TRUE	reported
0.00821645	6.2069E-23	Thickness betwee	INL_RPE_thic	TRUE	reported
0.0209839	2.51803E-08	Thickness betwee	INL_RPE_thic	TRUE	reported
0.0116083	3.37094E-08	Thickness betwee	INL_RPE_thic	TRUE	reported
0.00623438	2.18638E-11	Thickness betwee	INL_RPE_thic	TRUE	reported
0.00630568	7.2942E-16	Thickness betwee	INL_RPE_thic	TRUE	reported
0.00679821	1.65378E-09	Thickness betwee	INL_RPE_thic	TRUE	reported
0.00691408	4.81082E-26	Thickness betwee	INL_RPE_thic	TRUE	reported
0.00671953	3.96403E-09	Thickness betwee	INL_RPE_thic	TRUE	reported
0.00765858	9.984E-12	Thickness betwee	ISOS_RPE_thi	TRUE	reported

0.00635396	6.5197E-12	Thickness betwee	ISOS_RPE_thi	TRUE	reported
0.00707765	3.68933E-24	Thickness betwee	ISOS_RPE_thi	TRUE	reported
0.00757449	6.23492E-27	Thickness betwee	ISOS_RPE_thi	TRUE	reported
0.00640598	3.44901E-33	Thickness betwee	ISOS_RPE_thi	TRUE	reported
0.00633137	2.03741E-14	Thickness betwee	ISOS_RPE_thi	TRUE	reported
0.00629508	1.10483E-08	Thickness betwee	ISOS_RPE_thi	TRUE	reported
0.00693546	7.98613E-10	Thickness betwee	ISOS_RPE_thi	TRUE	reported
0.00651501	2.76872E-14	Thickness betwee	ISOS_RPE_thi	TRUE	reported
0.00858829	6.52194E-09	Thickness betwee	ISOS_RPE_thi	TRUE	reported
0.0108724	1.58737E-20	Thickness betwee	ISOS_RPE_thi	TRUE	reported
0.0145461	4.51099E-41	Thickness betwee	ISOS_RPE_thi	TRUE	reported
0.00813608	5.19378E-09	Thickness betwee	ISOS_RPE_thi	TRUE	reported
0.0111304	4.58301E-09	Thickness betwee	ISOS_RPE_thi	TRUE	reported
0.00775598	1.73825E-18	Thickness betwee	ISOS_RPE_thi	TRUE	reported
0.00897917	2.68989E-24	Thickness betwee	ISOS_RPE_thi	TRUE	reported
0.00735795	3.064E-09	Thickness betwee	ISOS_RPE_thi	TRUE	reported
0.00839038	1.35908E-10	Thickness betwee	ISOS_RPE_thi	TRUE	reported
0.00643839	9.60289E-09	Thickness betwee	ISOS_RPE_thi	TRUE	reported
0.0122322	9.88231E-11	Thickness betwee	ISOS_RPE_thi	TRUE	reported
0.00701812	8.64085E-09	Thickness betwee	ISOS_RPE_thi	TRUE	reported
0.00657782	3.73443E-20	Thickness betwee	ISOS_RPE_thi	TRUE	reported
0.00977862	4.27015E-10	Thickness betwee	ISOS_RPE_thi	TRUE	reported
0.00681033	2.02534E-08	Thickness betwee	ISOS_RPE_thi	TRUE	reported
0.00679001	1.21559E-22	Thickness betwee	ISOS_RPE_thi	TRUE	reported
0.00823713	1.17599E-08	Thickness betwee	ISOS_RPE_thi	TRUE	reported
0.00684097	2.75325E-19	Thickness betwee	ISOS_RPE_thi	TRUE	reported
0.00812733	3.00702E-16	Thickness betwee	ISOS_RPE_thi	TRUE	reported
0.00955814	9.00513E-25	Thickness betwee	ISOS_RPE_thi	TRUE	reported
0.00706578	1.02669E-08	Thickness betwee	ISOS_RPE_thi	TRUE	reported
0.00633405	4.3698E-49	Thickness betwee	ISOS_RPE_thi	TRUE	reported
0.00755273	4.40199E-22	Thickness betwee	ISOS_RPE_thi	TRUE	reported
0.00828848	1.20968E-11	Thickness betwee	ISOS_RPE_thi	TRUE	reported
0.00772715	1.26396E-15	Thickness betwee	ISOS_RPE_thi	TRUE	reported
0.00687425	1.20334E-22	Thickness betwee	ISOS_RPE_thi	TRUE	reported
0.0063632	5.05624E-20	Thickness betwee	ISOS_RPE_thi	TRUE	reported
0.00740092	5.38775E-50	Thickness betwee	ISOS_RPE_thi	TRUE	reported
0.00675588	8.44956E-10	Thickness betwee	ISOS_RPE_thi	TRUE	reported
0.00635877	3.84121E-22	Thickness betwee	ISOS_RPE_thi	TRUE	reported
0.00688659	5.81116E-12	Thickness betwee	ISOS_RPE_thi	TRUE	reported
0.0113867	9.1513E-25	Thickness betwee	ISOS_RPE_thi	TRUE	reported
0.00849454	1.39356E-11	Thickness betwee	ISOS_RPE_thi	TRUE	reported
0.00644067	6.72343E-46	Thickness betwee	ISOS_RPE_thi	TRUE	reported
0.00637835	2.22108E-11	Thickness betwee	ISOS_RPE_thi	TRUE	reported
0.0184334	8.03013E-28	Thickness betwee	ISOS_RPE_thi	TRUE	reported

0.00803761	3.12073E-09	Thickness betwee	ISOS_RPE_thi	TRUE	reported
0.00683527	1.01583E-12	Thickness betwee	ISOS_RPE_thi	TRUE	reported
0.00664875	1.60686E-34	Thickness betwee	ISOS_RPE_thi	TRUE	reported
0.00674022	6.37115E-12	Thickness betwee	ISOS_RPE_thi	TRUE	reported
0.00628836	2.93514E-31	Thickness betwee	ISOS_RPE_thi	TRUE	reported
0.00636888	8.08576E-13	Thickness betwee	ISOS_RPE_thi	TRUE	reported
0.00638544	5.78007E-09	Thickness betwee	ISOS_RPE_thi	TRUE	reported
0.00713964	9.54363E-24	Thickness betwee	ISOS_RPE_thi	TRUE	reported
0.00754248	1.01326E-23	Thickness betwee	ISOS_RPE_thi	TRUE	reported
0.00638762	1.61012E-28	Thickness betwee	ISOS_RPE_thi	TRUE	reported
0.00723074	1.58417E-11	Thickness betwee	ISOS_RPE_thi	TRUE	reported
0.00688699	2.45877E-09	Thickness betwee	ISOS_RPE_thi	TRUE	reported
0.00646227	2.57304E-12	Thickness betwee	ISOS_RPE_thi	TRUE	reported
0.01084	1.57491E-15	Thickness betwee	ISOS_RPE_thi	TRUE	reported
0.0145967	2.62952E-41	Thickness betwee	ISOS_RPE_thi	TRUE	reported
0.00647842	2.27164E-08	Thickness betwee	ISOS_RPE_thi	TRUE	reported
0.0114461	4.86045E-10	Thickness betwee	ISOS_RPE_thi	TRUE	reported
0.00773428	1.84297E-16	Thickness betwee	ISOS_RPE_thi	TRUE	reported
0.00994353	3.91512E-22	Thickness betwee	ISOS_RPE_thi	TRUE	reported
0.00732674	1.84168E-10	Thickness betwee	ISOS_RPE_thi	TRUE	reported
0.0203777	2.25193E-10	Thickness betwee	ISOS_RPE_thi	TRUE	reported
0.0204458	2.94964E-14	Thickness betwee	ISOS_RPE_thi	TRUE	reported
0.00686458	4.30314E-09	Thickness betwee	ISOS_RPE_thi	TRUE	reported
0.00655555	4.00147E-20	Thickness betwee	ISOS_RPE_thi	TRUE	reported
0.00974263	9.93476E-15	Thickness betwee	ISOS_RPE_thi	TRUE	reported
0.00695973	1.60276E-22	Thickness betwee	ISOS_RPE_thi	TRUE	reported
0.00826459	2.41925E-11	Thickness betwee	ISOS_RPE_thi	TRUE	reported
0.00680834	6.24709E-16	Thickness betwee	ISOS_RPE_thi	TRUE	reported
0.00993287	6.73339E-09	Thickness betwee	ISOS_RPE_thi	TRUE	reported
0.00778334	4.46309E-15	Thickness betwee	ISOS_RPE_thi	TRUE	reported
0.00954117	1.37928E-17	Thickness betwee	ISOS_RPE_thi	TRUE	reported
0.00796686	2.91205E-11	Thickness betwee	ISOS_RPE_thi	TRUE	reported
0.00734629	5.58293E-09	Thickness betwee	ISOS_RPE_thi	TRUE	reported
0.00629909	7.41301E-47	Thickness betwee	ISOS_RPE_thi	TRUE	reported
0.00790492	2.16924E-24	Thickness betwee	ISOS_RPE_thi	TRUE	reported
0.00742278	3.04383E-08	Thickness betwee	ISOS_RPE_thi	TRUE	reported
0.00825159	6.12006E-12	Thickness betwee	ISOS_RPE_thi	TRUE	reported
0.00939066	6.28003E-10	Thickness betwee	ISOS_RPE_thi	TRUE	reported
0.00768491	1.78805E-20	Thickness betwee	ISOS_RPE_thi	TRUE	reported
0.0067183	8.11514E-30	Thickness betwee	ISOS_RPE_thi	TRUE	reported
0.00633782	2.26813E-21	Thickness betwee	ISOS_RPE_thi	TRUE	reported
0.00737401	1.76803E-55	Thickness betwee	ISOS_RPE_thi	TRUE	reported
0.0065343	1.1057E-09	Thickness betwee	ISOS_RPE_thi	TRUE	reported
0.00672775	2.51553E-08	Thickness betwee	ISOS_RPE_thi	TRUE	reported

0.00634003	1.63022E-19	Thickness betwee	ISOS_RPE_thi	TRUE	reported
0.00690766	1.03219E-13	Thickness betwee	ISOS_RPE_thi	TRUE	reported
0.0113474	5.38491E-28	Thickness betwee	ISOS_RPE_thi	TRUE	reported
0.00842606	4.23546E-12	Thickness betwee	ISOS_RPE_thi	TRUE	reported
0.00772707	1.47426E-12	Thickness betwee	ISOS_RPE_thi	TRUE	reported
0.00642012	2.40867E-37	Thickness betwee	ISOS_RPE_thi	TRUE	reported
0.00655549	9.62651E-10	Thickness betwee	ISOS_RPE_thi	TRUE	reported
0.0183733	4.38264E-26	Thickness betwee	ISOS_RPE_thi	TRUE	reported
0.00801429	2.42822E-08	Thickness betwee	ISOS_RPE_thi	TRUE	reported
0.0068093	6.613E-13	Thickness betwee	ISOS_RPE_thi	TRUE	reported
0.00634503	1.32477E-41	Thickness betwee	ISOS_RPE_thi	TRUE	reported
0.00798147	2.72711E-08	Thickness betwee	ISOS_RPE_thi	TRUE	reported
0.0150231	3.02787E-08	Thickness betwee	ISOS_RPE_thi	TRUE	reported
0.00675585	4.35216E-13	Thickness betwee	ISOS_RPE_thi	TRUE	reported
0.00626353	5.57202E-30	Thickness betwee	ISOS_RPE_thi	TRUE	reported
0.00634881	2.90078E-11	Thickness betwee	ISOS_RPE_thi	TRUE	reported
0.00655125	1.08218E-10	Ganglion cell-inne	GCIPL_thickn	TRUE	reported
0.00688381	4.44313E-09	Ganglion cell-inne	GCIPL_thickn	TRUE	reported
0.00626724	3.18031E-08	Ganglion cell-inne	GCIPL_thickn	TRUE	reported
0.00997746	8.77418E-34	Ganglion cell-inne	GCIPL_thickn	TRUE	reported
0.00844105	1.17338E-08	Ganglion cell-inne	GCIPL_thickn	TRUE	reported
0.006266	1.66169E-09	Ganglion cell-inne	GCIPL_thickn	TRUE	reported
0.00657525	7.96396E-19	Ganglion cell-inne	GCIPL_thickn	TRUE	reported
0.00629336	3.04043E-08	Ganglion cell-inne	GCIPL_thickn	TRUE	reported
0.00623247	3.51133E-08	Ganglion cell-inne	GCIPL_thickn	TRUE	reported
0.0124582	5.31009E-12	Ganglion cell-inne	GCIPL_thickn	TRUE	reported
0.0119488	2.09128E-43	Ganglion cell-inne	GCIPL_thickn	TRUE	reported
0.0067604	1.28227E-08	Ganglion cell-inne	GCIPL_thickn	TRUE	reported
0.00978357	1.73394E-10	Ganglion cell-inne	GCIPL_thickn	TRUE	reported
0.00691465	1.44664E-16	Ganglion cell-inne	GCIPL_thickn	TRUE	reported
0.0105182	1.42749E-11	Ganglion cell-inne	GCIPL_thickn	TRUE	reported
0.00634099	6.83239E-52	Ganglion cell-inne	GCIPL_thickn	TRUE	reported
0.00709656	1.97907E-24	Ganglion cell-inne	GCIPL_thickn	TRUE	reported
0.00625443	3.40002E-09	Ganglion cell-inne	GCIPL_thickn	TRUE	reported
0.00711004	6.3404E-09	Ganglion cell-inne	GCIPL_thickn	TRUE	reported
0.00677315	2.03998E-08	Ganglion cell-inne	GCIPL_thickn	TRUE	reported
0.00668224	5.00645E-10	Ganglion cell-inne	GCIPL_thickn	TRUE	reported
0.0105347	3.99984E-09	Ganglion cell-inne	GCIPL_thickn	TRUE	reported
0.00725	4.65257E-12	Ganglion cell-inne	GCIPL_thickn	TRUE	reported
0.00820707	3.46948E-12	Ganglion cell-inne	GCIPL_thickn	TRUE	reported
0.00813134	7.69123E-18	Ganglion cell-inne	GCIPL_thickn	TRUE	reported
0.00915425	1.71134E-08	Ganglion cell-inne	GCIPL_thickn	TRUE	reported
0.0111663	1.57823E-20	Ganglion cell-inne	GCIPL_thickn	TRUE	reported
0.006666	2.94112E-09	Ganglion cell-inne	GCIPL_thickn	TRUE	reported

0.00674676	1.7844E-08	Ganglion cell-inne	GCIPL_thickn	TRUE	reported
0.00630738	1.66167E-09	Ganglion cell-inne	GCIPL_thickn	TRUE	reported
0.00647922	7.38712E-60	Ganglion cell-inne	GCIPL_thickn	TRUE	reported
0.00803601	6.01904E-10	Ganglion cell-inne	GCIPL_thickn	TRUE	reported
0.00620137	3.34734E-10	Ganglion cell-inne	GCIPL_thickn	TRUE	reported
0.00687897	1.20609E-15	Ganglion cell-inne	GCIPL_thickn	TRUE	reported

Table S4. The information for GWAS summary data for cardiacvascular disorders.

ID	Trait
finngen_R12_C_STROKE	Stroke
finngen_R12_CARDIAC_ARRHYTM	Cardiac arrhythmias
finngen_R12_I9_AF	Atrial fibrillation and flutter
finngen_R12_I9_CHD	coronary heart disease
finngen_R12_I9_HEARTFAIL	Heart failure
finngen_R12_I9_HYPTENS	Hypertension
finngen_R12_I9_MI_STRICT	Myocardial infarction

Abbreviation	Ncase	Ncontrol	Sample_size	Year
Stroke	53492	360342	413834	2024
CA	92926	288216	381142	2024
AF	63532	252810	316342	2024
CHD	56650	443698	500348	2024
HF	37653	462695	500348	2024
HBP	154630	345634	500264	2024
MI	31666	416171	447837	2024

PMID	Consortium	Sex	Population
36653562	FinnGen	Males and Females	European
36653562	FinnGen	Males and Females	European
36653562	FinnGen	Males and Females	European
36653562	FinnGen	Males and Females	European
36653562	FinnGen	Males and Females	European
36653562	FinnGen	Males and Females	European
36653562	FinnGen	Males and Females	European

Table S5. The instrumental variables for cardiac vascular disorders.

chr.exposu	pos.exposure	other_alle	effect_alle	SNP	pval.exposure	beta.exposure	se.exposure
1	56503083	G	A	rs7528118	1.85481E-08	0.0468746	0.00833322
4	110793263	T	G	rs1314330	4.91541E-10	-0.0490703	0.00788686
4	154604124	G	A	rs2066865	1.80809E-09	0.0476352	0.00792049
5	123313106	A	G	rs7278981	1.38874E-09	-0.0518492	0.00856053
6	1379759	A	T	rs7848665	5.66774E-09	0.0668552	0.0114748
6	31347929	G	A	rs9265969	3.44755E-10	0.0721273	0.0114904
6	34595387	A	G	rs205262	3.45406E-09	0.0478732	0.00810254
6	160589086	A	G	rs1045587	2.01967E-10	0.109981	0.017293
7	106771636	G	A	rs6248185	4.05471E-08	0.0437358	0.0079687
7	150993088	C	T	rs3918226	1.56016E-10	0.0908352	0.0141944
9	22124141	A	T	rs7857118	3.75059E-42	0.100139	0.00736055
12	111427245	C	G	rs7310615	1.35853E-09	-0.0449238	0.00741278
14	99613860	G	A	rs1379430	2.66017E-08	0.150512	0.0270585
19	11065175	A	G	rs1166692	1.56661E-09	-0.0525852	0.00870998
19	11416089	T	G	rs167479	6.13903E-11	0.0484491	0.00740775
20	59150608	G	A	rs7330686	7.59364E-09	0.0568237	0.0098358
1	3410985	A	G	rs1106270	1.65219E-10	0.0423633	0.00662899
1	10722151	C	T	rs188203	3.54014E-08	0.0518159	0.00939992
1	21925769	A	G	rs4654773	9.8712E-09	0.0434486	0.00757879
1	41078607	G	T	rs2885697	4.74745E-09	-0.0383004	0.0065406
1	50612551	G	A	rs7473816	3.3597E-15	0.163795	0.0207948
1	111937914	A	C	rs1214537	2.91783E-10	-0.0528016	0.00837711
1	147845539	C	T	rs7518566	2.56271E-11	-0.089397	0.0134034
1	154742838	T	C	rs2335249	2.79319E-13	0.0458524	0.0062777
1	154839804	C	T	rs3451587	3.00469E-33	0.0813346	0.00677006
1	169938856	C	A	rs488488	1.02052E-10	-0.105106	0.0162605
1	170622169	A	T	rs651386	2.27929E-13	-0.0469761	0.00640762
1	203065778	A	G	rs3737883	2.14684E-13	-0.047496	0.00647145
2	25889649	T	A	rs1298644	1.52915E-08	0.0367179	0.00648926
2	65052280	A	G	rs2723065	1.25083E-14	-0.0505159	0.00655136
2	174648092	G	A	rs7574892	2.08449E-11	0.0416138	0.00621102
2	178546938	A	G	rs2288327	8.25087E-19	0.0729549	0.00823736
2	200368139	C	G	rs296790	9.77913E-13	-0.0443923	0.00622301
3	12800305	A	G	rs7650482	1.88625E-19	0.0573099	0.00635387
3	38551902	T	C	rs1205390	9.56974E-26	-0.0663972	0.00632938
3	38668824	T	C	rs7373065	5.84117E-14	-0.147276	0.0196065
4	25407216	G	A	rs3481147	1.3787E-08	-0.0420541	0.00740909
4	109299246	G	A	rs1723461	2.83009E-13	0.119938	0.0164248
4	110593595	G	A	rs1312174	1.37088E-46	-0.0989423	0.00690335
4	110762205	T	C	rs1432603	2.9717E-213	0.265095	0.00850558
4	110844339	C	T	rs6838973	2.85496E-67	-0.107272	0.0061904
4	147506351	A	C	rs7804927	4.7709E-08	0.0496319	0.00909068

4	148103330	T	C	rs1172551	7.83592E-10	0.0383046	0.00623019
5	114953338	A	C	rs2842406	4.82303E-08	-0.0340402	0.00623708
5	138105376	G	C	rs678897	4.15623E-23	0.0677568	0.00684402
5	143128086	G	A	rs3776299	2.59352E-10	0.0393693	0.00622803
5	143378829	T	C	rs1051552	2.85693E-15	0.061884	0.00783639
5	173930807	T	C	rs5628141	2.44951E-10	-0.0399798	0.0063158
6	16415390	G	A	rs1137552	2.05305E-12	-0.071989	0.0102391
6	18362746	A	G	rs9371048	8.28991E-10	0.0412403	0.00671746
6	22599157	G	C	rs6456496	3.12176E-15	0.0558837	0.0070865
6	32218487	T	C	rs436388	6.28927E-14	0.0465128	0.00620013
6	36679512	G	A	rs3176326	3.15864E-17	-0.0696402	0.00825068
6	117240050	G	T	rs608815	4.25687E-10	0.0486567	0.00779217
6	122067705	G	A	rs1402538	6.01174E-12	-0.0453522	0.00659249
6	160589086	A	G	rs1045587	7.05294E-10	0.0908176	0.0147314
7	4683522	T	C	rs6170777	1.79094E-09	0.058261	0.0096848
7	102012025	T	C	rs202142	2.88875E-09	0.038683	0.00651471
7	116551247	C	A	rs1177384	1.27468E-36	0.0791703	0.0062636
7	128737511	C	T	rs6042505	4.48849E-15	0.0820635	0.0104666
7	150964321	G	A	rs7789146	4.16869E-14	-0.0559658	0.00740719
8	21980483	C	G	rs2858394	4.63106E-09	-0.0657183	0.0112149
8	117851173	A	T	rs1743035	2.22515E-08	0.0468884	0.00838262
8	140988666	C	G	rs1233447	4.5651E-09	-0.0368542	0.00628665
9	16742060	C	T	rs7853096	6.16127E-10	0.0640036	0.0103461
9	22124745	C	G	rs4977575	5.18203E-13	0.045265	0.00626902
9	95027463	A	G	rs1020963	5.18908E-09	0.0556507	0.00952761
9	133279427	T	C	rs635634	2.90677E-10	-0.0485706	0.00770513
10	49102206	T	C	rs4240499	5.61707E-10	-0.0397564	0.00641148
10	67582103	G	T	rs1152523	1.40809E-09	0.0458834	0.00757833
10	73690031	G	C	rs7644371	4.5772E-16	-0.0716164	0.00881734
10	89460885	T	C	rs7281671	1.84718E-09	0.0601151	0.0100013
10	103572695	C	T	rs7067666	1.30107E-11	0.0421047	0.00622066
10	103792691	A	G	rs728713	3.61077E-11	-0.0748785	0.0113123
11	49666620	A	T	rs1076960	5.37242E-09	0.0445241	0.00763025
11	128896471	C	T	rs7555744	6.90399E-15	0.0808173	0.0103795
12	26452912	A	T	rs7298923	7.21207E-10	0.0501063	0.00813234
12	114355435	C	T	rs883079	1.92975E-13	0.0492871	0.00670248
12	124337517	A	G	rs6488930	1.07892E-08	0.0393477	0.00688157
13	21502352	C	T	rs2315545	8.95509E-10	0.0387763	0.00632876
14	32515296	C	T	rs4981979	2.35256E-08	0.0400073	0.00716482
14	64217208	A	T	rs1152589	7.53651E-10	-0.0383013	0.0062234
14	76964338	T	A	rs1781043	1.84272E-10	-0.0402185	0.00630987
15	41539575	C	T	rs1185850	1.18214E-10	-0.0411768	0.00639232
15	73384923	C	T	rs7402296	1.28647E-16	0.0630192	0.00761579
15	80369856	C	A	rs1506955	9.07612E-14	0.096034	0.0128841

16	1932335	C	G	rs1392774	1.63719E-12	0.18694	0.0264701
16	53791576	C	T	rs9941349	4.16677E-11	0.0416158	0.00630732
16	73035989	T	C	rs1293244	1.92841E-37	0.0935356	0.00731467
17	1336980	G	A	rs1487993	3.84079E-08	-0.0581455	0.0105757
17	2296552	C	T	rs216200	1.9293E-10	-0.0413035	0.00648726
17	47060667	A	G	rs1451530	5.37527E-16	0.0628646	0.00775846
17	66205694	G	T	rs6504403	1.10126E-09	-0.0499219	0.00819199
19	11416089	T	G	rs167479	1.29569E-12	0.0446176	0.0062888
19	50439781	C	T	rs1178313	3.42768E-17	0.174861	0.0207403
20	21354475	T	C	rs2025811	3.34334E-09	-0.0465252	0.00786724
20	59159857	G	A	rs7841252	1.2386E-09	0.0504633	0.00830649
21	34746814	T	G	rs2834618	4.292E-09	-0.0576273	0.00981303
21	39311814	A	G	rs6222304	1.56434E-08	-0.0351251	0.00621204
22	18114735	T	C	rs464901	4.94129E-10	-0.0418167	0.00672191
1	10736809	T	C	rs880315	2.11982E-18	0.0765244	0.00874491
1	21977649	T	A	rs1157696	2.61312E-10	-0.0700993	0.0110914
1	50458271	T	G	rs7269046	2.94761E-09	0.164757	0.0277626
1	56503083	G	A	rs7528118	3.11365E-10	0.0618527	0.00982879
1	111912200	C	T	rs1209019	3.56862E-17	-0.0812606	0.00964374
1	112496139	G	C	rs7269904	8.78375E-09	0.0686328	0.0119306
1	147844695	G	A	rs7858128	6.23448E-16	-0.151141	0.0186948
1	154742838	T	C	rs2335249	2.59358E-17	0.0736899	0.00870673
1	154840154	T	C	rs1275418	2.37137E-52	0.14219	0.00933852
1	155114607	C	T	rs7367758	1.41844E-09	-0.0649963	0.0107372
1	170046896	C	T	rs5614794	4.05789E-12	0.153144	0.0220823
1	170622169	A	T	rs651386	3.56615E-19	-0.0796268	0.0088972
1	203065778	A	G	rs3737883	1.2031E-18	-0.0789247	0.00895405
1	228254502	G	T	rs1015827	3.95603E-09	0.0531699	0.00903324
2	25946418	T	C	rs1112629	5.59899E-09	-0.0517331	0.00887617
2	37032497	G	T	rs9309001	4.30289E-08	-0.048472	0.00884857
2	37864400	A	G	rs1149626	3.03676E-09	0.0666636	0.0112425
2	65052671	T	C	rs2723064	1.11224E-12	-0.0646946	0.00909163
2	71430887	T	A	rs6546692	2.45064E-09	0.0514113	0.00861924
2	145984604	A	C	rs1749085	2.5332E-09	-0.0748781	0.0125649
2	174648092	G	A	rs7574892	1.60391E-10	0.0551014	0.00861613
2	178546938	A	G	rs2288327	3.18566E-26	0.120329	0.0113585
2	200298833	A	G	rs295141	3.56115E-08	-0.0491633	0.00892039
3	12800724	T	G	rs4642101	6.29796E-23	0.0871966	0.0088448
3	38551902	T	C	rs1205390	3.95367E-15	-0.0690279	0.00878621
3	38645735	T	C	rs9856387	7.22104E-11	-0.0580156	0.0089036
3	69354767	T	C	rs1843050	3.0439E-09	-0.0583767	0.00984561
3	89447932	G	A	rs7633500	5.84682E-09	-0.0506424	0.00869983
3	111858929	C	G	rs9846313	5.8023E-11	0.0592383	0.00904575
4	38391648	A	T	rs2845608	3.10785E-09	-0.0514781	0.00868712

4	80261400	T	C	rs1250959	2.6804E-19	0.083196	0.00926344
4	109299246	G	A	rs1723461	9.90376E-21	0.209597	0.0224478
4	109646317	G	A	rs1003232	2.40049E-15	0.0678084	0.00856309
4	110593595	G	A	rs1312174	1.84502E-61	-0.159012	0.00961292
4	110774266	A	G	rs1906618	1.1508E-303	0.432827	0.0115439
4	110844339	C	T	rs6838973	2.48085E-97	-0.179339	0.00856576
4	111398117	T	C	rs1450103	7.0173E-09	-0.120123	0.0207448
4	146533954	T	C	rs1509394	1.14921E-08	0.135287	0.0237051
4	148058023	G	T	rs1021956	7.65949E-12	-0.0841519	0.0122943
4	173733984	A	G	rs5572990	1.08044E-11	-0.0814017	0.011979
5	214623	T	C	rs6555042	4.7949E-09	-0.0505083	0.0086278
5	32831833	T	C	rs1265649	5.00899E-09	0.0511012	0.00873991
5	42667769	T	C	rs6878512	4.12762E-10	0.068014	0.0108838
5	72467327	G	T	rs1019457	6.92277E-09	-0.0559376	0.00965643
5	114429285	A	G	rs338623	1.28991E-10	-0.0572854	0.00891136
5	115032070	G	T	rs1003962	1.03877E-09	-0.0532509	0.00872488
5	138105376	G	C	rs678897	2.86352E-26	0.100622	0.00948928
5	141545239	A	G	rs370479	6.92819E-09	0.0757112	0.0130702
5	143128086	G	A	rs3776299	2.64302E-11	0.0576336	0.00864696
5	143378829	T	C	rs1051552	1.02287E-10	0.0700312	0.0108348
5	159072359	G	A	rs5638823	3.41468E-09	-0.0530741	0.00897992
5	173246316	T	C	rs6884881	2.80867E-09	-0.0539033	0.00907095
5	173965395	A	T	rs1005406	2.13157E-12	-0.0617299	0.00878645
6	16414322	G	A	rs5943069	2.66502E-13	-0.104283	0.0142652
6	18209878	G	A	rs3496971	7.40457E-16	0.0744571	0.00923367
6	31723099	C	G	rs7574614	4.66982E-12	-0.0660482	0.00955107
6	36679512	G	A	rs3176326	3.35506E-10	-0.0721444	0.0114854
6	75557647	C	T	rs9341528	2.8086E-10	0.076396	0.0121091
6	117146480	G	A	rs1219632	2.5986E-08	0.0605118	0.0108706
6	122068760	C	T	rs868155	9.92887E-15	-0.073573	0.00950537
6	149077964	G	T	rs1179848	1.04764E-09	0.0785742	0.0128768
6	160584578	C	T	rs5573049	1.26809E-10	0.131986	0.0205235
7	856808	G	A	rs1023296	3.01301E-14	0.0653015	0.00859484
7	27105302	C	T	rs1725074	3.20007E-09	0.072901	0.0123123
7	27203619	G	A	rs2023844	4.81504E-17	0.124613	0.0148506
7	74567941	G	A	rs1027686	2.6833E-08	-0.0608343	0.0109396
7	116546187	A	G	rs3807989	9.74316E-32	0.102419	0.00873678
7	128776990	T	G	rs5598573	2.75931E-14	0.119454	0.0156988
7	150972779	C	G	rs3778873	2.57899E-09	-0.0617961	0.0103748
8	21969880	G	A	rs1706068	4.49966E-09	-0.0918794	0.0156665
8	101586969	T	C	rs1747745	3.67096E-08	0.074038	0.0134468
8	104954086	C	T	rs3571920	1.58234E-11	-0.069765	0.0103507
8	117851173	A	T	rs1743035	5.57571E-10	0.0719598	0.0116027
8	123551185	A	G	rs7732565	6.77485E-09	0.0981698	0.0169363

8	140708215	C	G	rs1010881	1.0937E-09	-0.0533559	0.0087539
9	22125348	A	C	rs1333048	6.62827E-17	0.0729273	0.00873018
9	94714202	A	G	rs1016013	2.42549E-12	-0.0632224	0.00902208
9	133278724	C	T	rs579459	5.13452E-11	-0.0677447	0.010316
10	21007590	A	G	rs599087	1.68547E-10	0.0875382	0.0137045
10	49102206	T	C	rs4240499	7.96159E-12	-0.0608817	0.00890181
10	67582103	G	T	rs1152523	1.88018E-15	0.0835326	0.0105086
10	73690031	G	C	rs7644371	6.13903E-18	-0.106034	0.0122868
10	79362726	C	A	rs1430739	3.60952E-09	-0.115159	0.0195147
10	103565017	C	T	rs1241550	5.89115E-20	0.137752	0.015061
10	103720629	T	A	rs3517605	2.93833E-21	0.118722	0.0125434
11	3854752	G	T	rs3813879	6.32703E-09	0.0546692	0.00941295
11	128896471	C	T	rs7555744	5.665E-20	0.130774	0.0142914
12	20777675	T	C	rs1104548	3.6196E-08	0.0486207	0.00882653
12	24617944	A	G	rs1728729	1.04797E-10	-0.0912466	0.0141252
12	26187053	C	G	rs1104842	1.63614E-09	0.0521665	0.00865066
12	53981037	G	A	rs1117077	2.49695E-09	0.0706962	0.0118585
12	56680399	T	G	rs2958126	4.12202E-09	-0.0598403	0.0101783
12	113945598	G	C	rs1417199	5.7779E-09	-0.0836204	0.0143602
12	114363967	G	A	rs7135659	1.84204E-30	0.108343	0.00944481
12	114935593	A	C	rs3436216	4.95678E-13	0.0628922	0.00870301
12	124337150	C	T	rs2229842	1.00023E-11	0.0652766	0.00959037
13	22798408	A	T	rs1285531	1.54056E-10	0.0666106	0.0104058
14	23413975	C	T	rs3729833	8.49474E-09	0.0760733	0.013211
14	32505638	A	C	rs8010040	1.1801E-08	0.0567705	0.00995524
14	64184945	C	A	rs1266927	5.00035E-15	-0.0682867	0.00872461
14	76964338	T	A	rs1781043	2.21157E-15	-0.0694736	0.0087621
15	73384923	C	T	rs7402296	5.1511E-22	0.101577	0.0105314
15	80369856	C	A	rs1506955	1.47367E-15	0.141674	0.0177556
15	90894158	A	T	rs2521501	6.11097E-09	0.0569759	0.00980029
16	1932335	C	G	rs1392774	3.39391E-15	0.285631	0.0362682
16	1953015	G	A	rs1401856	4.30685E-10	0.128487	0.0205826
16	4316205	C	A	rs1869180	6.92867E-09	0.0565309	0.00975909
16	53771295	C	A	rs1107598	6.23304E-11	0.0568312	0.00869238
16	71417021	T	A	rs7684436	1.33024E-08	0.0790181	0.0139064
16	72677612	T	C	rs7278939	1.15768E-10	0.0978323	0.0151801
16	73035989	T	C	rs1293244	1.91293E-67	0.174895	0.0100794
17	2300159	A	G	rs216193	2.6872E-12	-0.0621141	0.00888209
17	47019579	T	A	rs6504672	7.60852E-21	0.105384	0.011253
17	66205694	G	T	rs6504403	1.3872E-09	-0.0687843	0.0113562
17	70351774	A	G	rs312759	2.71025E-09	0.0524713	0.00882129
18	37092999	A	G	rs1169131	9.41846E-09	0.119512	0.0208177
18	48989546	G	T	rs9945890	1.32239E-09	0.0543743	0.00896576
19	2232050	G	A	rs740404	5.88031E-11	0.0845154	0.0129095

19	11416089	T	G	rs167479	5.36908E-17	0.0731174	0.00872698
19	12317195	C	T	rs1167319	1.97388E-08	0.0595827	0.0106127
19	50439781	C	T	rs1178313	1.28647E-24	0.289711	0.0282868
20	6346007	C	T	rs6054135	1.94993E-09	0.0535706	0.00892557
20	21286459	C	T	rs6113183	1.92619E-11	-0.0624005	0.00929751
20	59173534	G	A	rs7330688	1.43384E-15	0.0928938	0.0116372
21	34746814	T	G	rs2834618	2.05428E-09	-0.0821567	0.0137077
21	39230530	C	A	rs2836949	1.48594E-11	-0.0585014	0.00866783
22	18114735	T	C	rs464901	4.79502E-09	-0.0547689	0.00935559
1	37945773	G	A	rs871524	9.84532E-10	0.0434327	0.00710626
1	55039974	G	T	rs1159114	5.118E-26	-0.206009	0.0195282
1	55052188	C	G	rs613855	1.20606E-08	0.0406725	0.00713695
1	56536226	C	T	rs7266435	6.83125E-18	-0.0975883	0.0113242
1	56620630	C	T	rs7550087	4.05509E-13	0.0841555	0.0116017
1	109274968	G	T	rs1274037	1.82138E-20	-0.0801145	0.00864016
1	205093233	G	A	rs1090044	1.46393E-08	0.0413999	0.00730706
1	222650401	T	G	rs6718093	9.90148E-13	0.0578935	0.00811759
2	19742712	G	A	rs1698695	2.52872E-10	0.0841302	0.0133008
2	43463896	C	T	rs7279093	8.94355E-10	-0.0467909	0.00763658
2	62659875	C	T	rs1017763	1.28662E-08	0.0418986	0.00736636
2	85532004	T	C	rs1702639	2.25061E-13	0.0521617	0.00711328
2	145047651	G	A	rs1247676	1.7002E-08	-0.0435445	0.00772062
2	203104250	T	C	rs7293453	7.2144E-25	0.12157	0.0118055
2	215430291	A	G	rs1250240	3.51593E-10	-0.0550859	0.00877985
2	217816852	G	C	rs4674217	4.76991E-10	-0.0448449	0.00720227
3	14859044	T	G	rs294634	1.07624E-08	0.0431995	0.00755467
3	138403280	C	T	rs9818870	1.29778E-16	0.0934519	0.011295
3	154062618	C	T	rs6227683	1.61573E-09	-0.0542509	0.0089933
4	55748046	C	T	rs1310787	1.44245E-08	-0.0444131	0.00783539
4	119976692	A	G	rs1109855	9.22784E-12	-0.0493186	0.00723352
4	145838890	T	G	rs4835259	1.12785E-10	-0.045571	0.00706666
4	147110243	C	T	rs4350997	2.16471E-23	-0.0849475	0.00852442
4	147351643	T	C	rs7668383	1.56711E-18	-0.0815602	0.00928428
4	147443768	G	A	rs7702877	3.81241E-30	0.118571	0.0103936
5	108743971	A	C	rs1215370	2.28976E-08	-0.0396925	0.00710248
5	112057853	C	T	rs256274	7.85905E-10	-0.0438689	0.00713577
5	143136970	T	C	rs246603	2.85358E-08	0.0391701	0.00705743
6	2139216	A	G	rs1197027	1.88447E-10	0.0530269	0.00832386
6	12903725	A	G	rs9349379	1.67533E-62	0.117378	0.00703478
6	22577877	C	T	rs1211119	3.12738E-08	0.047122	0.00851477
6	32204288	T	G	rs1044506	1.06194E-12	0.0743365	0.0104372
6	36677919	A	G	rs2395655	1.33426E-08	-0.0418591	0.00736746
6	133782994	C	T	rs1208256	1.63832E-14	0.0553454	0.00720996
6	134057013	T	A	rs2492304	1.52223E-08	0.040036	0.0070747

6	160501838	A	G	rs1177333	4.80176E-30	0.366659	0.0321971
6	160564494	G	A	rs1180392	1.3128E-40	0.215517	0.0161529
7	19009765	G	A	rs2107595	4.49055E-18	0.0770296	0.00888911
7	100035359	T	C	rs1133790	5.76766E-11	-0.0492962	0.00752654
7	130023656	C	T	rs1155692	9.3218E-12	-0.0513026	0.00752613
7	150993088	C	T	rs3918226	9.49511E-12	0.0928229	0.0136225
8	19994534	T	A	rs1748926	2.18122E-13	-0.0604057	0.00823283
8	22172552	C	T	rs7322584	2.18524E-16	0.0860555	0.0104799
8	125466208	T	C	rs2001846	9.9106E-13	-0.0503193	0.00705567
9	21484063	C	T	rs7270341	4.55334E-08	0.0695774	0.0127246
9	21987585	T	C	rs3731204	1.77296E-14	0.0875977	0.0114266
9	22100177	G	C	rs1556516	6.0395E-156	0.188283	0.00707714
9	104900213	G	A	rs2575875	9.26873E-09	-0.0444065	0.00773148
9	121656031	G	A	rs6257278	4.70771E-08	0.0443848	0.00812609
10	44001523	T	C	rs1079351	7.81988E-19	0.0681072	0.00768483
10	89245159	C	A	rs2246941	9.53455E-11	0.046165	0.00713066
11	8849755	A	G	rs7127479	2.90623E-09	0.0469237	0.00790387
11	103802566	T	A	rs2839812	3.96917E-11	-0.0564921	0.00855265
11	116778201	G	C	rs964184	1.98016E-13	-0.0727262	0.00989456
12	111569952	C	T	rs653178	2.94171E-17	-0.0602523	0.00713143
13	110136094	G	A	rs2000660	7.89405E-13	-0.0960507	0.0134093
13	110397276	T	C	rs9515203	2.99433E-23	-0.0800161	0.00805567
14	99613860	G	A	rs1379430	1.54525E-20	0.235842	0.0253871
14	99716388	A	G	rs8022722	2.11261E-08	0.0432895	0.00772681
14	99853261	C	T	rs7271000	1.15064E-08	0.0939683	0.0164658
15	74061220	T	G	rs4886868	2.17691E-10	-0.0450224	0.00709203
15	78774311	A	G	rs5770807	1.53179E-30	0.0836979	0.00728625
15	88887075	G	T	rs5658980	6.94065E-32	-0.256596	0.0218353
15	90753689	C	T	rs2838499	6.49038E-09	0.0937267	0.0161497
16	56956804	C	A	rs247617	6.3352E-09	-0.0456857	0.00786645
16	70622878	G	A	rs6205028	5.01291E-10	-0.103876	0.0167039
16	75401563	A	G	rs1043197	4.60893E-19	0.0635125	0.00711921
16	86741733	G	A	rs735344	4.13143E-11	-0.0483378	0.00732469
17	2124812	A	G	rs2760751	1.85866E-13	0.057856	0.00786238
17	49359450	A	T	rs8081878	3.95968E-09	0.0414047	0.00703459
17	64330199	T	A	rs7225219	6.38587E-10	0.0449142	0.00726696
18	62445742	A	C	rs948757	2.61024E-08	0.0415957	0.00747351
19	11072901	G	A	rs1131138	4.65265E-24	-0.08776	0.00867466
19	41307470	T	C	rs15052	2.11553E-09	0.04997	0.00834409
19	44908822	C	T	rs7412	3.46737E-33	-0.193072	0.0160866
20	34845826	A	G	rs5584537	2.261E-14	0.0551828	0.00722776
21	34221526	G	A	rs2845106	6.32266E-26	0.101821	0.00967017
22	43928850	C	T	rs738408	3.88929E-08	-0.0463734	0.00843796
1	50991770	C	T	rs1162877	6.96306E-12	0.185573	0.0270577

1	61429037	G	A	rs2207792	8.77122E-10	-0.0578166	0.00943127
2	36949070	G	A	rs1112455	1.72894E-10	-0.0519503	0.008138
3	12801869	C	T	rs9825233	3.04032E-10	0.0520105	0.00825995
4	110784612	T	C	rs6817105	1.00184E-22	0.111808	0.0113952
4	110844339	C	T	rs6838973	3.87168E-12	-0.0565415	0.00814509
4	147436591	A	T	rs1113440	1.14154E-08	-0.110524	0.0193622
6	36679072	T	C	rs3176323	3.19963E-21	-0.0857263	0.0090658
10	73650119	C	T	rs2177843	4.30279E-08	-0.0628079	0.0114656
11	133294632	C	A	rs7475888	6.95072E-09	0.069921	0.0120718
16	73025260	C	A	rs4499262	3.16242E-08	0.0532507	0.0096256
17	2253616	C	G	rs1295055	7.27244E-10	-0.0525576	0.00853201
1	3412095	C	T	rs2493292	3.19948E-10	0.0505311	0.0080351
1	6717840	A	C	rs4908568	3.56336E-08	-0.027807	0.00504552
1	10736809	T	C	rs880315	2.4322E-45	0.072252	0.00511287
1	11905735	C	T	rs7264028	4.77749E-32	-0.112386	0.00953802
1	25021220	G	A	rs1124924	1.83316E-11	0.0345966	0.00514928
1	26908191	G	A	rs1272405	1.32639E-10	0.0474202	0.00738159
1	27507578	T	C	rs7412540	1.9668E-09	-0.0502432	0.00837313
1	28850345	C	T	rs1507113	3.16665E-08	-0.069085	0.0124883
1	43475681	C	T	rs2105028	6.0927E-09	0.0302014	0.00519443
1	47598380	A	C	rs1121157	1.98107E-11	0.0524963	0.0078266
1	56482752	T	C	rs1088897	2.86154E-16	0.0425579	0.0052033
1	61298334	C	G	rs2806430	4.65232E-08	0.0340912	0.00623911
1	88677066	C	A	rs1092247	7.50689E-10	-0.0328677	0.00533999
1	111797684	A	G	rs509705	3.77485E-08	0.0305255	0.00554901
1	112501706	G	T	rs1077675	4.53211E-61	0.109887	0.00666495
1	113267461	A	G	rs7552539	1.42466E-09	0.0470386	0.00777154
1	115266818	A	C	rs7726710	5.24807E-12	0.142132	0.0206027
1	150718472	T	C	rs7270257	4.25785E-09	-0.0498715	0.00849042
1	156047077	C	G	rs7459304	1.5717E-09	0.0562579	0.00931911
1	175192794	C	G	rs1091290	1.464E-10	-0.0326053	0.00508736
1	228024458	T	A	rs1745414	4.70219E-14	0.0382319	0.00507059
1	230737742	G	T	rs1317181	1.25228E-22	0.0604563	0.00617579
2	26693978	C	G	rs3400478	5.40132E-56	-0.0807468	0.00512184
2	27161153	C	T	rs3578434	2.55035E-12	0.0359635	0.00513726
2	29209910	T	C	rs3738867	7.06399E-09	0.0913956	0.0157867
2	43208318	G	A	rs2888854	1.46791E-13	-0.0400237	0.00541591
2	45678054	A	T	rs513386	3.94285E-10	0.0385845	0.00616733
2	59856209	A	T	rs4672324	2.1928E-10	-0.0319031	0.00502634
2	61440832	A	T	rs1153273	1.3106E-10	-0.0333647	0.00519219
2	111907624	C	T	rs1717487	1.87258E-09	-0.0312231	0.0051965
2	126569144	T	C	rs2119149	1.13752E-08	-0.0448412	0.0078547
2	144969054	A	G	rs5594433	2.85957E-12	0.040219	0.00575835
2	158566826	G	A	rs3771612	1.6092E-08	-0.0416089	0.00736507

2	161743572	G	T	rs1160993	1.23994E-16	-0.0590706	0.0071348
2	163587293	A	G	rs2704368	4.42996E-21	-0.0640883	0.00680206
2	164097664	T	A	rs268263	2.83988E-13	0.0454709	0.00622737
2	180594207	C	T	rs1686733	2.13255E-09	0.0334579	0.00558807
2	181024335	G	C	rs4362519	1.45858E-10	-0.0336203	0.00524527
2	187238749	G	T	rs1682871	3.27393E-08	-0.0289414	0.0052372
2	197257052	T	G	rs1162476	1.39107E-08	-0.109568	0.0193089
2	218835276	G	A	rs7805819	1.38197E-12	0.0647115	0.00913249
2	226233671	A	G	rs1515114	1.30707E-13	0.0377359	0.0050957
3	11562645	A	G	rs1000010	4.8473E-11	-0.0337292	0.00512947
3	14852633	C	T	rs4685218	1.03157E-12	0.059855	0.00839924
3	27243252	A	T	rs9860251	5.38592E-09	0.0531675	0.00911215
3	27521497	C	T	rs2643826	1.41775E-22	0.0501136	0.00512583
3	35603950	A	C	rs5878399	8.15192E-09	0.0310764	0.00539025
3	39546046	T	C	rs1996097	6.85962E-11	-0.0338368	0.00518675
3	53523985	G	A	rs3821843	4.0738E-11	0.0353801	0.00535952
3	53821056	C	T	rs9836592	1.40799E-10	0.0339685	0.00529514
3	113679795	T	C	rs7322652	1.5742E-09	0.033756	0.00559192
3	134110550	T	A	rs9851392	1.43781E-12	0.0357447	0.00504842
3	158496682	C	T	rs6795376	7.63273E-10	0.0311224	0.00505859
3	168869411	A	G	rs6808266	1.78033E-13	0.0463273	0.00629077
3	168977105	A	T	rs1171537	2.46292E-10	-0.0589752	0.00931782
3	169455882	G	A	rs1080483	3.48779E-10	-0.031674	0.00504735
4	26794784	A	G	rs7324986	8.4918E-11	-0.0373447	0.00575275
4	38385623	G	T	rs2291434	7.46999E-10	-0.0312925	0.00508342
4	55633053	A	G	rs5589399	1.47184E-09	0.0327136	0.00540951
4	80261400	T	C	rs1250959	8.31381E-64	0.0915316	0.00542771
4	85798012	T	C	rs1701095	1.39219E-13	0.0518001	0.00700277
4	95072085	A	G	rs4426786	5.66057E-09	-0.0693392	0.0119007
4	105990585	G	C	rs1311272	8.51334E-17	0.0558294	0.00670714
4	108141354	C	T	rs1250310	1.59599E-08	-0.0287243	0.00508312
4	110475792	T	C	rs1879053	1.02023E-16	-0.0419515	0.00505294
4	123785315	A	G	rs1836039	5.28689E-11	-0.0464188	0.00707319
4	137537806	C	A	rs2869559	1.95056E-08	0.0283627	0.00505005
4	143188382	T	C	rs4690777	1.49176E-13	-0.0386924	0.00523728
4	147474942	A	G	rs6537481	1.10634E-09	0.0393164	0.00645245
4	155482095	C	T	rs4691670	8.1903E-12	-0.0352	0.00514982
4	155718736	G	T	rs7268914	8.20352E-30	-0.0690787	0.00609096
5	3714531	A	G	rs6077654	3.61993E-11	0.0371871	0.00561836
5	32831833	T	C	rs1265649	1.09094E-30	0.058705	0.00509752
5	61676692	C	T	rs1881554	8.26418E-21	0.15419	0.01648
5	67019708	T	C	rs3857275	6.50639E-10	-0.0356437	0.00576979
5	78550860	C	T	rs1005046	1.41759E-08	-0.0290328	0.0051193
5	91066193	G	A	rs1819074	4.03943E-10	-0.0332792	0.00532255

5	96841147	T	A	rs2549805	3.3136E-18	0.0558047	0.0064142
5	115151036	C	G	rs7732228	4.38208E-09	-0.0298811	0.00509128
5	123115076	G	A	rs1007741	1.74582E-14	0.0459517	0.0059926
5	128536057	C	T	rs6860901	9.40806E-27	0.0587577	0.00548764
5	138439892	C	G	rs982085	1.85242E-08	0.0284568	0.00505876
5	148504863	C	T	rs9763488	1.06116E-10	0.0333088	0.00515778
5	157967495	C	T	rs1137008	1.59074E-11	0.0428757	0.00636197
5	158450445	G	C	rs4371736	4.62807E-20	-0.0474999	0.00517859
5	158500976	G	A	rs4565196	2.89454E-08	0.0426593	0.00768954
5	158880841	G	C	rs1345721	1.50633E-09	-0.0390761	0.0064656
5	171152405	A	G	rs1113467	9.01945E-10	0.0326195	0.00532489
5	173966701	G	A	rs1007307	4.7469E-08	-0.0275622	0.00504752
6	1619912	T	C	rs2569882	2.73269E-10	-0.032907	0.00521239
6	15168043	G	A	rs9396569	3.78879E-08	0.0378675	0.00688446
6	24185728	T	C	rs2791973	5.64508E-09	-0.0304292	0.00522216
6	26081992	A	G	rs1116639	1.43384E-14	0.0618655	0.00804148
6	31461190	G	A	rs2596472	6.08976E-18	0.0465816	0.00539711
6	43292922	T	C	rs6901866	4.45656E-18	0.0481208	0.00555252
6	43796814	C	T	rs1358980	5.93745E-14	0.037823	0.00503671
6	51551109	T	C	rs6927317	1.6293E-11	0.0346541	0.0051447
6	54163271	T	C	rs6915002	3.32284E-08	-0.0287178	0.00519918
6	71420806	G	T	rs5360147	3.63706E-08	0.0723423	0.0131349
6	79081265	A	G	rs1413969	3.50833E-17	0.0430932	0.00511294
6	125897815	C	G	rs6918791	1.40475E-14	0.0435968	0.00566493
6	126821313	C	T	rs6242632	2.60615E-33	0.0605042	0.00503127
6	142835226	A	T	rs198645	1.49279E-12	-0.0378145	0.00534468
6	150683634	G	C	rs1708010	3.82384E-24	-0.0964243	0.00951304
6	165747361	A	G	rs7303023	4.81172E-11	-0.0727229	0.0110578
7	754991	C	T	rs7303244	2.55817E-08	-0.048751	0.00875358
7	1866830	G	A	rs5723449	1.72552E-10	-0.0371863	0.00582495
7	7201668	A	G	rs4724960	1.43417E-12	0.0358441	0.00506221
7	26346886	C	G	rs3757666	2.55853E-10	0.0340102	0.00537846
7	26383196	T	C	rs2699803	1.64778E-13	0.0373243	0.00506116
7	27206274	T	C	rs3735533	2.13747E-48	0.126923	0.00868232
7	27312031	G	A	rs1156358	1.93598E-23	0.0713354	0.0071505
7	27410467	G	A	rs2836157	5.23745E-09	0.0795727	0.0136268
7	70551042	A	T	rs6245579	5.21303E-10	-0.0402842	0.00648433
7	77011229	T	C	rs3603618	1.39782E-09	-0.0383264	0.00632895
7	106771412	T	C	rs1747717	8.32722E-21	0.0513972	0.00549385
7	116791373	C	T	rs193686	5.9676E-12	-0.0384968	0.00559513
7	130045757	G	A	rs5617956	1.19613E-09	-0.0321086	0.00528036
7	131638951	A	G	rs7551178	4.80507E-13	0.0676895	0.0093614
7	150379067	G	C	rs5727467	3.98841E-11	-0.0377195	0.00571118
7	150993088	C	T	rs3918226	1.7636E-60	0.160152	0.00976242

7	151718170	C	T	rs1025373	2.88602E-14	0.0454905	0.00598296
7	156519248	G	A	rs6249419	2.65993E-09	0.0300406	0.00504771
8	6508313	C	G	rs2442466	3.24459E-09	0.0298193	0.00503814
8	10422718	G	A	rs7831557	2.11495E-18	-0.0444078	0.0050746
8	26030594	G	A	rs1008657	1.84247E-12	-0.0388216	0.00550979
8	30970859	G	A	rs2725366	7.42335E-09	-0.0300842	0.00520392
8	54533008	C	G	rs1731001	4.62839E-08	0.0276407	0.00505773
8	75804502	T	G	rs2977324	2.76516E-09	0.0332573	0.00559419
8	76839799	A	G	rs6986186	3.76765E-08	0.0402248	0.00731173
8	94922679	A	G	rs2515236	9.3218E-13	0.0367359	0.00514497
8	104954030	G	A	rs3578370	3.58839E-20	-0.064205	0.00697901
8	128366357	A	G	rs1178585	6.91911E-09	-0.0329904	0.00569499
8	142910802	A	G	rs3802228	4.07474E-12	-0.035202	0.00507631
9	4099937	C	A	rs628919	4.74395E-09	-0.0296305	0.00505994
9	16755123	A	G	rs1330298	2.07052E-08	0.034474	0.00614949
9	22110132	T	C	rs1412834	1.43351E-14	0.0390153	0.00507132
9	34547538	A	G	rs7874497	3.67451E-16	0.0426638	0.00523556
9	35915060	C	T	rs6722740	1.05342E-12	-0.0601339	0.00844179
9	109989291	C	T	rs1021755	1.17031E-11	0.0370488	0.00546134
9	126896973	G	C	rs1237593	3.03928E-10	0.0555166	0.0088167
9	133533893	C	T	rs9696459	1.14375E-08	-0.0313974	0.00550069
9	133589541	C	A	rs7277926	8.22243E-29	-0.115069	0.0103315
9	133876227	A	G	rs1073999	1.19994E-09	0.046936	0.00771942
9	137327051	C	G	rs5799483	2.25824E-10	-0.037908	0.00597667
10	18151515	G	A	rs1888693	6.65426E-14	0.0386989	0.00516363
10	18437125	G	A	rs7070847	1.98573E-09	-0.035643	0.00594151
10	21439525	C	T	rs1884600	4.04576E-11	-0.0654675	0.0099157
10	61751548	G	A	rs5754119	5.11093E-16	-0.0675587	0.0083315
10	62793174	A	G	rs1848797	5.10529E-10	-0.0326757	0.00525685
10	63450792	T	C	rs3550607	1.94971E-08	0.029605	0.00527116
10	67884201	A	G	rs3740051	8.75931E-09	0.0428419	0.00744667
10	80449104	C	G	rs7088877	1.71969E-09	-0.0304915	0.00506311
10	94279840	G	C	rs2274224	3.91201E-18	-0.0460647	0.00530618
10	103089359	G	C	rs1078673	5.52459E-27	-0.0943608	0.00877246
10	103756892	G	A	rs7898224	1.89365E-12	0.0365343	0.005188
10	104095898	A	T	rs1265002	1.0207E-15	-0.0945746	0.011786
10	105691244	A	C	rs1277112	5.76634E-11	-0.0393278	0.00600455
10	113961456	A	T	rs7415755	1.13708E-08	0.0765638	0.0134113
10	114021768	T	C	rs2782980	2.13698E-29	0.059565	0.00529134
10	127984035	C	T	rs1101599	1.29972E-08	0.0332535	0.00584821
11	1865986	T	C	rs612652	4.15432E-31	0.0589463	0.00508188
11	2293355	C	A	rs5564092	8.91867E-14	-0.0498615	0.00668742
11	10312695	T	A	rs7285165	1.40994E-16	0.0524827	0.00635085
11	17389473	C	T	rs3527117	8.20352E-09	-0.0289483	0.00502207

11	27199522	C	T	rs1102986	3.03222E-09	-0.0299181	0.00504535
11	27439164	T	C	rs4351777	9.89008E-11	-0.0451866	0.00698552
11	28390723	G	T	rs1418780	3.93097E-15	0.0402595	0.00512396
11	30555411	G	A	rs1172328	3.43194E-08	0.0653004	0.0118344
11	32457420	G	A	rs7126322	2.09406E-08	0.0304425	0.00543224
11	45538333	G	T	rs1129687	4.30784E-08	0.0712577	0.0130086
11	47553818	A	T	rs2868996	1.93598E-12	0.0416628	0.00591883
11	57422975	G	A	rs7106223	8.8247E-11	-0.0417618	0.00643895
11	60086382	C	T	rs2583477	1.59503E-08	0.0295545	0.00522995
11	61510774	T	C	rs751984	1.87456E-24	-0.0685263	0.00671468
11	65775268	G	A	rs489574	3.50106E-11	-0.0371468	0.00560809
11	100739815	T	C	rs604723	4.82503E-31	0.06657	0.00574548
11	100994717	C	T	rs483657	2.70178E-09	-0.0404919	0.00680678
11	116833462	C	A	rs7123454	9.33684E-11	-0.042706	0.00659317
11	119922780	G	A	rs665759	1.33909E-09	0.035079	0.00578609
11	122656504	G	C	rs7129204	1.09169E-11	0.0476212	0.00700944
12	12730636	A	G	rs1282843	8.65167E-16	-0.0407047	0.00505987
12	15363011	C	T	rs1084616	7.94127E-10	0.0360716	0.00586903
12	17902271	G	A	rs1148506	7.5024E-13	-0.0937264	0.0130721
12	20198342	A	T	rs7307367	7.90133E-15	-0.0473911	0.0060999
12	49896945	A	G	rs297935	1.38309E-08	0.0335956	0.00591944
12	50279701	G	A	rs7967954	1.12616E-21	-0.0487558	0.00509751
12	51558016	C	T	rs7820402	1.65817E-10	0.0715895	0.0112033
12	53064805	C	T	rs1048565	1.49968E-11	-0.0647104	0.00958965
12	53173006	G	A	rs7139122	1.69278E-28	0.196741	0.0177674
12	54025136	G	A	rs754133	1.06463E-32	-0.0609389	0.00511712
12	56589468	C	T	rs941208	2.12623E-08	-0.0301541	0.00538331
12	69307904	C	A	rs1122851	7.48445E-09	-0.0364272	0.00630264
12	70158859	G	A	rs1177030	1.27101E-08	0.0490701	0.00862404
12	89599730	A	T	rs1110533	3.37287E-24	-0.0962137	0.00948079
12	89995520	A	C	rs6538214	2.77134E-08	-0.0279931	0.00503899
12	111569952	C	T	rs653178	1.09926E-39	-0.067201	0.00509755
12	115118062	A	G	rs35429	3.0662E-19	-0.046921	0.00523303
12	115491115	A	C	rs6490021	1.08518E-17	0.0469457	0.00548141
13	21744572	A	C	rs628137	1.35957E-15	0.0408596	0.00511444
13	29545363	C	T	rs9508490	4.18697E-13	-0.038064	0.00525066
13	71794332	C	G	rs9318030	1.10454E-08	0.0482099	0.00843738
13	71888152	A	G	rs9542771	3.05028E-08	-0.0298694	0.00539302
13	114292933	T	C	rs9590507	1.78916E-10	0.0392333	0.00615094
14	68779316	A	G	rs8013475	4.29072E-08	0.036949	0.00674443
14	103521843	G	T	rs1136165	2.83792E-11	0.036077	0.00542124
15	31383649	C	T	rs7962258	4.71726E-10	-0.0380773	0.00611366
15	40995491	A	G	rs3101436	4.41977E-15	-0.0413807	0.00527653
15	66651647	C	T	rs1291176	3.37839E-09	0.0303769	0.00513812

15	67713674	T	C	rs1984442	2.84315E-13	0.0383436	0.00525138
15	69390577	A	G	rs1175396	1.87612E-09	-0.120139	0.0199959
15	73340528	C	T	rs512943	2.54619E-09	-0.0428565	0.00719254
15	80726137	C	G	rs7174038	3.56123E-19	0.0458499	0.00512301
15	89480327	G	A	rs1780772	5.0782E-10	-0.0399427	0.00642509
15	90882002	C	T	rs6227	3.18053E-34	0.0693057	0.00568167
15	91286867	C	T	rs7403845	4.30517E-08	0.0545857	0.0099648
16	1194631	C	T	rs3519883	2.7919E-12	0.0376302	0.00538511
16	4543238	C	G	rs1176459	1.37145E-10	0.108206	0.016857
16	4853242	C	T	rs8056889	1.83459E-08	-0.0285133	0.0050673
16	20381010	G	A	rs7792461	2.61903E-09	-0.0362295	0.00608506
16	51718812	C	T	rs3549023	3.73585E-09	0.0298403	0.00506155
16	53769275	A	T	rs1558901	1.58708E-34	0.0621802	0.00507402
16	66880589	C	T	rs4547449	1.55797E-10	0.0663013	0.0103602
16	75330981	A	C	rs3975148	1.32343E-09	0.0307628	0.00507259
16	81482117	G	T	rs2966083	1.86114E-09	-0.0307772	0.00512144
16	81569630	T	C	rs8045875	4.7698E-14	0.0386237	0.00512382
16	89662919	G	A	rs1164535	4.45656E-12	0.0383509	0.00554053
17	7237287	A	G	rs222852	6.76239E-19	-0.0456525	0.00514178
17	7587061	G	C	rs2302661	1.39476E-12	-0.0356114	0.00502661
17	45075638	A	G	rs8074938	8.54496E-10	-0.032817	0.00534961
17	46939827	G	A	rs11874	2.2558E-21	0.0634678	0.00668611
17	48881846	G	A	rs1165015	6.38337E-10	-0.0384722	0.00622461
17	49441016	G	A	rs9897429	6.5013E-11	0.0329985	0.00505203
17	58370936	C	T	rs2257205	6.30725E-10	-0.0447288	0.00723468
17	60712745	T	C	rs7953887	5.70099E-09	-0.0800804	0.013747
17	61394696	C	T	rs2286526	6.97911E-19	0.0494006	0.00556612
17	63479839	C	T	rs4298	4.70652E-14	-0.0659777	0.00875059
17	77320798	A	G	rs1436138	3.32506E-13	-0.0383585	0.00526865
18	23577175	C	T	rs4296334	5.57751E-10	-0.0326914	0.00527117
18	25313137	C	T	rs6679444	4.51544E-11	0.0532279	0.00808185
18	46462004	T	G	rs1296211	4.57794E-08	-0.0354622	0.00648664
18	50613868	C	T	rs4599004	1.67032E-12	-0.0394259	0.00558474
19	11113815	A	G	rs1569372	8.62979E-11	0.0327304	0.00504384
19	11207796	G	A	rs1434665	7.84874E-16	0.168031	0.0208565
19	11416089	T	G	rs167479	9.57635E-52	0.0772992	0.00510745
19	12855447	C	T	rs1166734	1.81051E-12	-0.100572	0.0142689
19	17065947	C	G	rs1334665	6.48634E-13	0.0436973	0.00607764
19	46284488	C	T	rs2005873	1.91902E-09	-0.0303602	0.00505622
19	50377070	G	A	rs3765108	1.22546E-09	-0.104453	0.0171886
20	6365809	C	G	rs7610054	1.81176E-08	0.0294954	0.00523984
20	8643454	G	A	rs6108165	7.30113E-09	-0.035825	0.00619398
20	10678234	G	C	rs6040076	2.67055E-10	-0.032312	0.00511525
20	10956332	G	A	rs6218566	2.28087E-19	0.0529043	0.005879

20	21135350	G	A	rs6047267	3.08234E-08	-0.0322241	0.00582011
20	32627862	G	A	rs1248171	5.1428E-13	0.0461136	0.00638564
20	41633783	G	A	rs6029750	1.88847E-08	-0.0293425	0.00521929
20	44181555	C	G	rs6017285	1.02148E-10	0.0489667	0.0075756
20	58855687	C	T	rs5585084	6.29999E-10	-0.0687039	0.0111122
20	58963461	C	A	rs1172343	3.41138E-09	0.0567171	0.00959604
20	59126654	G	C	rs6015445	2.8688E-69	0.119434	0.00678933
20	59302566	A	G	rs9679855	7.28115E-17	0.115692	0.013868
20	59391414	A	C	rs1809576	3.23564E-10	-0.087868	0.013976
20	63852558	T	C	rs1923031	3.21736E-13	-0.0379033	0.00520295
21	15206480	G	A	rs2823140	4.95062E-10	0.0323299	0.00519718
21	33416432	A	T	rs2300373	5.37465E-09	0.0376996	0.00646079
21	43371011	A	C	rs607398	4.1505E-11	0.0366599	0.00555572
21	43592595	A	G	rs1454651	2.81676E-10	0.0343317	0.0054421
22	28870037	A	G	rs5752822	1.27497E-16	0.0508267	0.00614154
22	29952422	A	T	rs7866077	4.24835E-09	0.0391116	0.00665817
22	31167569	A	C	rs5994376	4.46365E-08	0.027602	0.00504474
1	37945773	G	A	rs871524	2.95706E-08	0.0513578	0.00926374
1	55039974	G	T	rs1159114	8.18842E-23	-0.252076	0.0256381
1	55053342	G	T	rs639750	1.12099E-11	0.0634082	0.00933843
1	56025105	C	T	rs1048977	1.28115E-08	0.0546973	0.00961532
1	56536655	C	A	rs3927760	5.07692E-19	0.107663	0.0120827
1	109274968	G	T	rs1274037	8.43141E-14	-0.0839113	0.011243
1	222623985	T	C	rs1909195	3.23594E-13	0.0841541	0.0115529
2	19742712	G	A	rs1698695	1.9635E-09	0.103759	0.0172908
2	43848344	T	C	rs7686638	3.11365E-08	-0.0929413	0.0167918
2	62784536	C	T	rs1301321	1.32828E-09	-0.0579796	0.00956137
2	85533272	C	T	rs6731005	2.22946E-13	0.0676144	0.00921896
2	136240824	C	T	rs4954585	1.43572E-08	0.0555468	0.00979822
2	203104250	T	C	rs7293453	3.75405E-17	0.129643	0.0153964
2	215435462	C	T	rs1250258	4.36506E-08	-0.0614004	0.0112139
2	232843517	C	T	rs7595118	3.35521E-09	-0.0572502	0.00968176
3	138352059	C	T	rs1332434	3.61402E-08	0.0713968	0.0129606
4	57028551	G	C	rs1608165	6.35785E-09	0.0640753	0.011034
4	147110243	C	T	rs4350997	2.61517E-14	-0.0848156	0.0111364
4	147351643	T	C	rs7668383	8.94129E-13	-0.0860319	0.0120394
4	147443768	G	A	rs7702877	2.14931E-24	0.138452	0.0135841
5	111925780	G	A	rs1316072	1.02882E-08	0.0527757	0.009217
5	132631790	T	C	rs1005670	1.34927E-08	-0.0557162	0.00980971
6	12903725	A	G	rs9349379	6.15319E-49	0.134646	0.00915763
6	22612400	G	A	rs6909752	1.66948E-09	0.063038	0.0104591
6	32182024	C	T	rs9391855	4.06668E-10	0.114136	0.0182576
6	39218967	G	C	rs4714224	2.026E-09	-0.0586612	0.00978386
6	133696717	G	A	rs3453704	3.22033E-15	0.0925878	0.0117467

6	133893387	C	G	rs1219028	2.06824E-15	-0.0733258	0.00923828
6	149455573	T	C	rs421453	2.34909E-08	-0.0528374	0.00946211
6	160501838	A	G	rs1177333	5.38394E-21	0.395072	0.0420226
6	160564494	G	A	rs1180392	2.31953E-37	0.2665	0.0208643
7	19009765	G	A	rs2107595	5.665E-13	0.0835046	0.0115845
7	130023656	C	T	rs1155692	2.86424E-08	-0.0544281	0.00980768
7	150993088	C	T	rs3918226	1.46926E-13	0.130245	0.0176247
8	19994534	T	A	rs1748926	4.469E-10	-0.0668966	0.0107263
8	22181786	T	C	rs7829907	3.32751E-08	0.0755816	0.0136842
9	21993965	T	C	rs2811711	2.39823E-09	0.0891164	0.0149318
9	22100177	G	C	rs1556516	2.1135E-111	0.206641	0.00921363
9	133348545	G	C	rs2853896	3.49969E-08	0.0847193	0.0153633
10	44001523	T	C	rs1079351	4.05135E-15	0.0786467	0.0100144
10	89246097	C	T	rs2246833	7.54484E-09	0.0537149	0.00929592
11	8853962	C	T	rs1137174	4.30537E-08	0.0565832	0.0103295
11	65793149	C	T	rs642803	9.35298E-09	-0.0540535	0.0094136
11	103798234	A	T	rs2019090	1.76246E-08	-0.0625806	0.011108
11	116778201	G	C	rs964184	1.00401E-09	-0.0787046	0.0128839
12	57146968	C	G	rs7968719	3.07001E-08	0.0516454	0.00932665
12	111569952	C	T	rs653178	2.82358E-13	-0.067838	0.00928962
13	110149776	G	T	rs13260	2.68677E-10	-0.107169	0.0169682
13	110397276	T	C	rs9515203	9.60285E-19	-0.0928324	0.0105018
14	99682624	T	C	rs8003602	6.60998E-11	0.0674781	0.0103348
15	73982607	T	G	rs7174985	1.45512E-08	0.0533011	0.0094059
15	78761766	A	G	rs1232488	1.65082E-19	-0.0860743	0.00952753
15	88887075	G	T	rs5658980	5.87354E-29	-0.322019	0.028835
16	28242148	T	C	rs9932382	1.1286E-08	0.0576999	0.0101048
16	70662912	T	A	rs6205028	2.10683E-08	-0.112136	0.0200137
16	75401563	A	G	rs1043197	4.66659E-12	0.0641514	0.00927665
17	2249963	G	C	rs7213347	8.71525E-10	0.0620584	0.0101215
19	11048849	G	T	rs1205205	2.66073E-20	-0.104978	0.0113713
19	41307470	T	C	rs15052	3.3123E-09	0.0642585	0.0108631
19	44908822	C	T	rs7412	2.57395E-30	-0.240807	0.0210455
20	34877486	A	C	rs6120750	3.90661E-11	0.061809	0.00935427
21	34221526	G	A	rs2845106	1.58161E-24	0.12817	0.0125387

eaf.exposur	ncase.expo	ncontrol.e	samplesize	id.exposur	exposure	mr_keep.e	pval_origin.exposure
0.257451	53492	360342	413834	STROKE	STROKE	TRUE	reported
0.692036	53492	360342	413834	STROKE	STROKE	TRUE	reported
0.301009	53492	360342	413834	STROKE	STROKE	TRUE	reported
0.24271	53492	360342	413834	STROKE	STROKE	TRUE	reported
0.113477	53492	360342	413834	STROKE	STROKE	TRUE	reported
0.11217	53492	360342	413834	STROKE	STROKE	TRUE	reported
0.280693	53492	360342	413834	STROKE	STROKE	TRUE	reported
0.045393	53492	360342	413834	STROKE	STROKE	TRUE	reported
0.297718	53492	360342	413834	STROKE	STROKE	TRUE	reported
0.069874	53492	360342	413834	STROKE	STROKE	TRUE	reported
0.423842	53492	360342	413834	STROKE	STROKE	TRUE	reported
0.588626	53492	360342	413834	STROKE	STROKE	TRUE	reported
0.018485	53492	360342	413834	STROKE	STROKE	TRUE	reported
0.229236	53492	360342	413834	STROKE	STROKE	TRUE	reported
0.57599	53492	360342	413834	STROKE	STROKE	TRUE	reported
0.162384	53492	360342	413834	STROKE	STROKE	TRUE	reported
0.323414	92926	288216	381142	Cardiac ar	Cardiac ar	TRUE	reported
0.124139	92926	288216	381142	Cardiac ar	Cardiac ar	TRUE	reported
0.784944	92926	288216	381142	Cardiac ar	Cardiac ar	TRUE	reported
0.661009	92926	288216	381142	Cardiac ar	Cardiac ar	TRUE	reported
0.021762	92926	288216	381142	Cardiac ar	Cardiac ar	TRUE	reported
0.166651	92926	288216	381142	Cardiac ar	Cardiac ar	TRUE	reported
0.058242	92926	288216	381142	Cardiac ar	Cardiac ar	TRUE	reported
0.564915	92926	288216	381142	Cardiac ar	Cardiac ar	TRUE	reported
0.297483	92926	288216	381142	Cardiac ar	Cardiac ar	TRUE	reported
0.962997	92926	288216	381142	Cardiac ar	Cardiac ar	TRUE	reported
0.379802	92926	288216	381142	Cardiac ar	Cardiac ar	TRUE	reported
0.643547	92926	288216	381142	Cardiac ar	Cardiac ar	TRUE	reported
0.644713	92926	288216	381142	Cardiac ar	Cardiac ar	TRUE	reported
0.341832	92926	288216	381142	Cardiac ar	Cardiac ar	TRUE	reported
0.484181	92926	288216	381142	Cardiac ar	Cardiac ar	TRUE	reported
0.16787	92926	288216	381142	Cardiac ar	Cardiac ar	TRUE	reported
0.522047	92926	288216	381142	Cardiac ar	Cardiac ar	TRUE	reported
0.609181	92926	288216	381142	Cardiac ar	Cardiac ar	TRUE	reported
0.406355	92926	288216	381142	Cardiac ar	Cardiac ar	TRUE	reported
0.97489	92926	288216	381142	Cardiac ar	Cardiac ar	TRUE	reported
0.229838	92926	288216	381142	Cardiac ar	Cardiac ar	TRUE	reported
0.037177	92926	288216	381142	Cardiac ar	Cardiac ar	TRUE	reported
0.291015	92926	288216	381142	Cardiac ar	Cardiac ar	TRUE	reported
0.148543	92926	288216	381142	Cardiac ar	Cardiac ar	TRUE	reported
0.500003	92926	288216	381142	Cardiac ar	Cardiac ar	TRUE	reported
0.133938	92926	288216	381142	Cardiac ar	Cardiac ar	TRUE	reported

0.531298	92926	288216	381142	Cardiac arı	Cardiac arı	TRUE	reported
0.458915	92926	288216	381142	Cardiac arı	Cardiac arı	TRUE	reported
0.2869	92926	288216	381142	Cardiac arı	Cardiac arı	TRUE	reported
0.505451	92926	288216	381142	Cardiac arı	Cardiac arı	TRUE	reported
0.192527	92926	288216	381142	Cardiac arı	Cardiac arı	TRUE	reported
0.413379	92926	288216	381142	Cardiac arı	Cardiac arı	TRUE	reported
0.104273	92926	288216	381142	Cardiac arı	Cardiac arı	TRUE	reported
0.308	92926	288216	381142	Cardiac arı	Cardiac arı	TRUE	reported
0.256352	92926	288216	381142	Cardiac arı	Cardiac arı	TRUE	reported
0.5074	92926	288216	381142	Cardiac arı	Cardiac arı	TRUE	reported
0.173361	92926	288216	381142	Cardiac arı	Cardiac arı	TRUE	reported
0.196917	92926	288216	381142	Cardiac arı	Cardiac arı	TRUE	reported
0.332534	92926	288216	381142	Cardiac arı	Cardiac arı	TRUE	reported
0.045772	92926	288216	381142	Cardiac arı	Cardiac arı	TRUE	reported
0.116931	92926	288216	381142	Cardiac arı	Cardiac arı	TRUE	reported
0.343629	92926	288216	381142	Cardiac arı	Cardiac arı	TRUE	reported
0.561745	92926	288216	381142	Cardiac arı	Cardiac arı	TRUE	reported
0.096555	92926	288216	381142	Cardiac arı	Cardiac arı	TRUE	reported
0.229082	92926	288216	381142	Cardiac arı	Cardiac arı	TRUE	reported
0.085289	92926	288216	381142	Cardiac arı	Cardiac arı	TRUE	reported
0.16124	92926	288216	381142	Cardiac arı	Cardiac arı	TRUE	reported
0.423248	92926	288216	381142	Cardiac arı	Cardiac arı	TRUE	reported
0.09934	92926	288216	381142	Cardiac arı	Cardiac arı	TRUE	reported
0.429315	92926	288216	381142	Cardiac arı	Cardiac arı	TRUE	reported
0.878015	92926	288216	381142	Cardiac arı	Cardiac arı	TRUE	reported
0.799089	92926	288216	381142	Cardiac arı	Cardiac arı	TRUE	reported
0.377358	92926	288216	381142	Cardiac arı	Cardiac arı	TRUE	reported
0.212752	92926	288216	381142	Cardiac arı	Cardiac arı	TRUE	reported
0.146703	92926	288216	381142	Cardiac arı	Cardiac arı	TRUE	reported
0.107668	92926	288216	381142	Cardiac arı	Cardiac arı	TRUE	reported
0.452599	92926	288216	381142	Cardiac arı	Cardiac arı	TRUE	reported
0.919769	92926	288216	381142	Cardiac arı	Cardiac arı	TRUE	reported
0.214709	92926	288216	381142	Cardiac arı	Cardiac arı	TRUE	reported
0.098209	92926	288216	381142	Cardiac arı	Cardiac arı	TRUE	reported
0.820837	92926	288216	381142	Cardiac arı	Cardiac arı	TRUE	reported
0.687118	92926	288216	381142	Cardiac arı	Cardiac arı	TRUE	reported
0.289421	92926	288216	381142	Cardiac arı	Cardiac arı	TRUE	reported
0.402469	92926	288216	381142	Cardiac arı	Cardiac arı	TRUE	reported
0.745811	92926	288216	381142	Cardiac arı	Cardiac arı	TRUE	reported
0.522491	92926	288216	381142	Cardiac arı	Cardiac arı	TRUE	reported
0.574864	92926	288216	381142	Cardiac arı	Cardiac arı	TRUE	reported
0.618328	92926	288216	381142	Cardiac arı	Cardiac arı	TRUE	reported
0.206109	92926	288216	381142	Cardiac arı	Cardiac arı	TRUE	reported
0.060552	92926	288216	381142	Cardiac arı	Cardiac arı	TRUE	reported

0.013329	92926	288216	381142	Cardiac arı	Cardiac arı	TRUE	reported
0.409229	92926	288216	381142	Cardiac arı	Cardiac arı	TRUE	reported
0.231452	92926	288216	381142	Cardiac arı	Cardiac arı	TRUE	reported
0.095864	92926	288216	381142	Cardiac arı	Cardiac arı	TRUE	reported
0.652806	92926	288216	381142	Cardiac arı	Cardiac arı	TRUE	reported
0.197831	92926	288216	381142	Cardiac arı	Cardiac arı	TRUE	reported
0.829246	92926	288216	381142	Cardiac arı	Cardiac arı	TRUE	reported
0.575959	92926	288216	381142	Cardiac arı	Cardiac arı	TRUE	reported
0.021819	92926	288216	381142	Cardiac arı	Cardiac arı	TRUE	reported
0.808059	92926	288216	381142	Cardiac arı	Cardiac arı	TRUE	reported
0.167278	92926	288216	381142	Cardiac arı	Cardiac arı	TRUE	reported
0.115057	92926	288216	381142	Cardiac arı	Cardiac arı	TRUE	reported
0.517032	92926	288216	381142	Cardiac arı	Cardiac arı	TRUE	reported
0.315638	92926	288216	381142	Cardiac arı	Cardiac arı	TRUE	reported
0.411539	63532	252810	316342	Atrial fibril	Atrial fibril	TRUE	reported
0.189277	63532	252810	316342	Atrial fibril	Atrial fibril	TRUE	reported
0.023466	63532	252810	316342	Atrial fibril	Atrial fibril	TRUE	reported
0.257402	63532	252810	316342	Atrial fibril	Atrial fibril	TRUE	reported
0.276184	63532	252810	316342	Atrial fibril	Atrial fibril	TRUE	reported
0.154511	63532	252810	316342	Atrial fibril	Atrial fibril	TRUE	reported
0.058049	63532	252810	316342	Atrial fibril	Atrial fibril	TRUE	reported
0.565714	63532	252810	316342	Atrial fibril	Atrial fibril	TRUE	reported
0.298907	63532	252810	316342	Atrial fibril	Atrial fibril	TRUE	reported
0.205451	63532	252810	316342	Atrial fibril	Atrial fibril	TRUE	reported
0.037287	63532	252810	316342	Atrial fibril	Atrial fibril	TRUE	reported
0.379018	63532	252810	316342	Atrial fibril	Atrial fibril	TRUE	reported
0.642881	63532	252810	316342	Atrial fibril	Atrial fibril	TRUE	reported
0.346131	63532	252810	316342	Atrial fibril	Atrial fibril	TRUE	reported
0.386587	63532	252810	316342	Atrial fibril	Atrial fibril	TRUE	reported
0.389707	63532	252810	316342	Atrial fibril	Atrial fibril	TRUE	reported
0.179253	63532	252810	316342	Atrial fibril	Atrial fibril	TRUE	reported
0.341207	63532	252810	316342	Atrial fibril	Atrial fibril	TRUE	reported
0.480987	63532	252810	316342	Atrial fibril	Atrial fibril	TRUE	reported
0.136419	63532	252810	316342	Atrial fibril	Atrial fibril	TRUE	reported
0.484844	63532	252810	316342	Atrial fibril	Atrial fibril	TRUE	reported
0.168218	63532	252810	316342	Atrial fibril	Atrial fibril	TRUE	reported
0.369009	63532	252810	316342	Atrial fibril	Atrial fibril	TRUE	reported
0.609747	63532	252810	316342	Atrial fibril	Atrial fibril	TRUE	reported
0.405599	63532	252810	316342	Atrial fibril	Atrial fibril	TRUE	reported
0.620645	63532	252810	316342	Atrial fibril	Atrial fibril	TRUE	reported
0.261035	63532	252810	316342	Atrial fibril	Atrial fibril	TRUE	reported
0.444901	63532	252810	316342	Atrial fibril	Atrial fibril	TRUE	reported
0.647049	63532	252810	316342	Atrial fibril	Atrial fibril	TRUE	reported
0.423411	63532	252810	316342	Atrial fibril	Atrial fibril	TRUE	reported

0.311733	63532	252810	316342	Atrial fibril	Atrial fibril	TRUE	reported
0.037368	63532	252810	316342	Atrial fibril	Atrial fibril	TRUE	reported
0.492426	63532	252810	316342	Atrial fibril	Atrial fibril	TRUE	reported
0.289192	63532	252810	316342	Atrial fibril	Atrial fibril	TRUE	reported
0.150757	63532	252810	316342	Atrial fibril	Atrial fibril	TRUE	reported
0.497848	63532	252810	316342	Atrial fibril	Atrial fibril	TRUE	reported
0.046258	63532	252810	316342	Atrial fibril	Atrial fibril	TRUE	reported
0.033751	63532	252810	316342	Atrial fibril	Atrial fibril	TRUE	reported
0.857819	63532	252810	316342	Atrial fibril	Atrial fibril	TRUE	reported
0.153823	63532	252810	316342	Atrial fibril	Atrial fibril	TRUE	reported
0.491087	63532	252810	316342	Atrial fibril	Atrial fibril	TRUE	reported
0.585485	63532	252810	316342	Atrial fibril	Atrial fibril	TRUE	reported
0.802481	63532	252810	316342	Atrial fibril	Atrial fibril	TRUE	reported
0.279253	63532	252810	316342	Atrial fibril	Atrial fibril	TRUE	reported
0.629999	63532	252810	316342	Atrial fibril	Atrial fibril	TRUE	reported
0.420728	63532	252810	316342	Atrial fibril	Atrial fibril	TRUE	reported
0.287424	63532	252810	316342	Atrial fibril	Atrial fibril	TRUE	reported
0.123863	63532	252810	316342	Atrial fibril	Atrial fibril	TRUE	reported
0.505261	63532	252810	316342	Atrial fibril	Atrial fibril	TRUE	reported
0.1933	63532	252810	316342	Atrial fibril	Atrial fibril	TRUE	reported
0.371147	63532	252810	316342	Atrial fibril	Atrial fibril	TRUE	reported
0.65385	63532	252810	316342	Atrial fibril	Atrial fibril	TRUE	reported
0.413547	63532	252810	316342	Atrial fibril	Atrial fibril	TRUE	reported
0.103923	63532	252810	316342	Atrial fibril	Atrial fibril	TRUE	reported
0.331606	63532	252810	316342	Atrial fibril	Atrial fibril	TRUE	reported
0.289039	63532	252810	316342	Atrial fibril	Atrial fibril	TRUE	reported
0.172365	63532	252810	316342	Atrial fibril	Atrial fibril	TRUE	reported
0.146369	63532	252810	316342	Atrial fibril	Atrial fibril	TRUE	reported
0.193913	63532	252810	316342	Atrial fibril	Atrial fibril	TRUE	reported
0.291839	63532	252810	316342	Atrial fibril	Atrial fibril	TRUE	reported
0.130295	63532	252810	316342	Atrial fibril	Atrial fibril	TRUE	reported
0.045478	63532	252810	316342	Atrial fibril	Atrial fibril	TRUE	reported
0.485999	63532	252810	316342	Atrial fibril	Atrial fibril	TRUE	reported
0.142862	63532	252810	316342	Atrial fibril	Atrial fibril	TRUE	reported
0.903768	63532	252810	316342	Atrial fibril	Atrial fibril	TRUE	reported
0.810133	63532	252810	316342	Atrial fibril	Atrial fibril	TRUE	reported
0.569767	63532	252810	316342	Atrial fibril	Atrial fibril	TRUE	reported
0.081736	63532	252810	316342	Atrial fibril	Atrial fibril	TRUE	reported
0.225836	63532	252810	316342	Atrial fibril	Atrial fibril	TRUE	reported
0.084635	63532	252810	316342	Atrial fibril	Atrial fibril	TRUE	reported
0.114664	63532	252810	316342	Atrial fibril	Atrial fibril	TRUE	reported
0.224491	63532	252810	316342	Atrial fibril	Atrial fibril	TRUE	reported
0.161678	63532	252810	316342	Atrial fibril	Atrial fibril	TRUE	reported
0.069028	63532	252810	316342	Atrial fibril	Atrial fibril	TRUE	reported

0.412056	63532	252810	316342	Atrial fibril	Atrial fibril	TRUE	reported
0.418608	63532	252810	316342	Atrial fibril	Atrial fibril	TRUE	reported
0.652924	63532	252810	316342	Atrial fibril	Atrial fibril	TRUE	reported
0.778123	63532	252810	316342	Atrial fibril	Atrial fibril	TRUE	reported
0.113667	63532	252810	316342	Atrial fibril	Atrial fibril	TRUE	reported
0.377267	63532	252810	316342	Atrial fibril	Atrial fibril	TRUE	reported
0.213393	63532	252810	316342	Atrial fibril	Atrial fibril	TRUE	reported
0.146038	63532	252810	316342	Atrial fibril	Atrial fibril	TRUE	reported
0.053655	63532	252810	316342	Atrial fibril	Atrial fibril	TRUE	reported
0.087389	63532	252810	316342	Atrial fibril	Atrial fibril	TRUE	reported
0.135363	63532	252810	316342	Atrial fibril	Atrial fibril	TRUE	reported
0.694999	63532	252810	316342	Atrial fibril	Atrial fibril	TRUE	reported
0.099074	63532	252810	316342	Atrial fibril	Atrial fibril	TRUE	reported
0.610664	63532	252810	316342	Atrial fibril	Atrial fibril	TRUE	reported
0.105488	63532	252810	316342	Atrial fibril	Atrial fibril	TRUE	reported
0.461216	63532	252810	316342	Atrial fibril	Atrial fibril	TRUE	reported
0.162699	63532	252810	316342	Atrial fibril	Atrial fibril	TRUE	reported
0.769211	63532	252810	316342	Atrial fibril	Atrial fibril	TRUE	reported
0.101708	63532	252810	316342	Atrial fibril	Atrial fibril	TRUE	reported
0.701871	63532	252810	316342	Atrial fibril	Atrial fibril	TRUE	reported
0.433303	63532	252810	316342	Atrial fibril	Atrial fibril	TRUE	reported
0.28443	63532	252810	316342	Atrial fibril	Atrial fibril	TRUE	reported
0.218897	63532	252810	316342	Atrial fibril	Atrial fibril	TRUE	reported
0.119612	63532	252810	316342	Atrial fibril	Atrial fibril	TRUE	reported
0.744423	63532	252810	316342	Atrial fibril	Atrial fibril	TRUE	reported
0.434111	63532	252810	316342	Atrial fibril	Atrial fibril	TRUE	reported
0.575004	63532	252810	316342	Atrial fibril	Atrial fibril	TRUE	reported
0.207315	63532	252810	316342	Atrial fibril	Atrial fibril	TRUE	reported
0.060844	63532	252810	316342	Atrial fibril	Atrial fibril	TRUE	reported
0.265912	63532	252810	316342	Atrial fibril	Atrial fibril	TRUE	reported
0.013469	63532	252810	316342	Atrial fibril	Atrial fibril	TRUE	reported
0.045134	63532	252810	316342	Atrial fibril	Atrial fibril	TRUE	reported
0.279251	63532	252810	316342	Atrial fibril	Atrial fibril	TRUE	reported
0.426895	63532	252810	316342	Atrial fibril	Atrial fibril	TRUE	reported
0.108546	63532	252810	316342	Atrial fibril	Atrial fibril	TRUE	reported
0.087486	63532	252810	316342	Atrial fibril	Atrial fibril	TRUE	reported
0.233058	63532	252810	316342	Atrial fibril	Atrial fibril	TRUE	reported
0.631223	63532	252810	316342	Atrial fibril	Atrial fibril	TRUE	reported
0.175659	63532	252810	316342	Atrial fibril	Atrial fibril	TRUE	reported
0.829701	63532	252810	316342	Atrial fibril	Atrial fibril	TRUE	reported
0.391351	63532	252810	316342	Atrial fibril	Atrial fibril	TRUE	reported
0.04433	63532	252810	316342	Atrial fibril	Atrial fibril	TRUE	reported
0.632074	63532	252810	316342	Atrial fibril	Atrial fibril	TRUE	reported
0.127404	63532	252810	316342	Atrial fibril	Atrial fibril	TRUE	reported

0.574019	63532	252810	316342	Atrial fibril	Atrial fibril	TRUE	reported
0.206577	63532	252810	316342	Atrial fibril	Atrial fibril	TRUE	reported
0.022104	63532	252810	316342	Atrial fibril	Atrial fibril	TRUE	reported
0.627349	63532	252810	316342	Atrial fibril	Atrial fibril	TRUE	reported
0.690574	63532	252810	316342	Atrial fibril	Atrial fibril	TRUE	reported
0.163535	63532	252810	316342	Atrial fibril	Atrial fibril	TRUE	reported
0.114447	63532	252810	316342	Atrial fibril	Atrial fibril	TRUE	reported
0.555586	63532	252810	316342	Atrial fibril	Atrial fibril	TRUE	reported
0.315125	63532	252810	316342	Atrial fibril	Atrial fibril	TRUE	reported
0.429937	56650	443698	500348	Major corc	Major corc	TRUE	reported
0.036292	56650	443698	500348	Major corc	Major corc	TRUE	reported
0.439505	56650	443698	500348	Major corc	Major corc	TRUE	reported
0.11034	56650	443698	500348	Major corc	Major corc	TRUE	reported
0.10134	56650	443698	500348	Major corc	Major corc	TRUE	reported
0.214503	56650	443698	500348	Major corc	Major corc	TRUE	reported
0.366458	56650	443698	500348	Major corc	Major corc	TRUE	reported
0.745526	56650	443698	500348	Major corc	Major corc	TRUE	reported
0.073096	56650	443698	500348	Major corc	Major corc	TRUE	reported
0.311398	56650	443698	500348	Major corc	Major corc	TRUE	reported
0.648553	56650	443698	500348	Major corc	Major corc	TRUE	reported
0.42676	56650	443698	500348	Major corc	Major corc	TRUE	reported
0.706807	56650	443698	500348	Major corc	Major corc	TRUE	reported
0.095654	56650	443698	500348	Major corc	Major corc	TRUE	reported
0.799413	56650	443698	500348	Major corc	Major corc	TRUE	reported
0.396822	56650	443698	500348	Major corc	Major corc	TRUE	reported
0.316549	56650	443698	500348	Major corc	Major corc	TRUE	reported
0.105386	56650	443698	500348	Major corc	Major corc	TRUE	reported
0.191571	56650	443698	500348	Major corc	Major corc	TRUE	reported
0.28596	56650	443698	500348	Major corc	Major corc	TRUE	reported
0.614633	56650	443698	500348	Major corc	Major corc	TRUE	reported
0.544728	56650	443698	500348	Major corc	Major corc	TRUE	reported
0.786105	56650	443698	500348	Major corc	Major corc	TRUE	reported
0.179388	56650	443698	500348	Major corc	Major corc	TRUE	reported
0.127273	56650	443698	500348	Major corc	Major corc	TRUE	reported
0.459931	56650	443698	500348	Major corc	Major corc	TRUE	reported
0.429966	56650	443698	500348	Major corc	Major corc	TRUE	reported
0.527415	56650	443698	500348	Major corc	Major corc	TRUE	reported
0.23136	56650	443698	500348	Major corc	Major corc	TRUE	reported
0.452246	56650	443698	500348	Major corc	Major corc	TRUE	reported
0.215037	56650	443698	500348	Major corc	Major corc	TRUE	reported
0.866157	56650	443698	500348	Major corc	Major corc	TRUE	reported
0.362139	56650	443698	500348	Major corc	Major corc	TRUE	reported
0.393455	56650	443698	500348	Major corc	Major corc	TRUE	reported
0.456081	56650	443698	500348	Major corc	Major corc	TRUE	reported

0.010889	56650	443698	500348	Major corc	Major corc	TRUE	reported
0.04623	56650	443698	500348	Major corc	Major corc	TRUE	reported
0.193217	56650	443698	500348	Major corc	Major corc	TRUE	reported
0.330033	56650	443698	500348	Major corc	Major corc	TRUE	reported
0.325942	56650	443698	500348	Major corc	Major corc	TRUE	reported
0.070159	56650	443698	500348	Major corc	Major corc	TRUE	reported
0.245312	56650	443698	500348	Major corc	Major corc	TRUE	reported
0.128387	56650	443698	500348	Major corc	Major corc	TRUE	reported
0.548703	56650	443698	500348	Major corc	Major corc	TRUE	reported
0.081471	56650	443698	500348	Major corc	Major corc	TRUE	reported
0.103842	56650	443698	500348	Major corc	Major corc	TRUE	reported
0.421157	56650	443698	500348	Major corc	Major corc	TRUE	reported
0.302681	56650	443698	500348	Major corc	Major corc	TRUE	reported
0.245951	56650	443698	500348	Major corc	Major corc	TRUE	reported
0.694529	56650	443698	500348	Major corc	Major corc	TRUE	reported
0.420195	56650	443698	500348	Major corc	Major corc	TRUE	reported
0.274068	56650	443698	500348	Major corc	Major corc	TRUE	reported
0.78678	56650	443698	500348	Major corc	Major corc	TRUE	reported
0.855258	56650	443698	500348	Major corc	Major corc	TRUE	reported
0.585729	56650	443698	500348	Major corc	Major corc	TRUE	reported
0.078898	56650	443698	500348	Major corc	Major corc	TRUE	reported
0.261355	56650	443698	500348	Major corc	Major corc	TRUE	reported
0.018761	56650	443698	500348	Major corc	Major corc	TRUE	reported
0.29152	56650	443698	500348	Major corc	Major corc	TRUE	reported
0.046961	56650	443698	500348	Major corc	Major corc	TRUE	reported
0.544595	56650	443698	500348	Major corc	Major corc	TRUE	reported
0.368519	56650	443698	500348	Major corc	Major corc	TRUE	reported
0.028765	56650	443698	500348	Major corc	Major corc	TRUE	reported
0.050673	56650	443698	500348	Major corc	Major corc	TRUE	reported
0.278611	56650	443698	500348	Major corc	Major corc	TRUE	reported
0.049064	56650	443698	500348	Major corc	Major corc	TRUE	reported
0.566989	56650	443698	500348	Major corc	Major corc	TRUE	reported
0.371563	56650	443698	500348	Major corc	Major corc	TRUE	reported
0.717751	56650	443698	500348	Major corc	Major corc	TRUE	reported
0.491204	56650	443698	500348	Major corc	Major corc	TRUE	reported
0.623841	56650	443698	500348	Major corc	Major corc	TRUE	reported
0.329874	56650	443698	500348	Major corc	Major corc	TRUE	reported
0.214486	56650	443698	500348	Major corc	Major corc	TRUE	reported
0.230151	56650	443698	500348	Major corc	Major corc	TRUE	reported
0.05304	56650	443698	500348	Major corc	Major corc	TRUE	reported
0.384133	56650	443698	500348	Major corc	Major corc	TRUE	reported
0.153285	56650	443698	500348	Major corc	Major corc	TRUE	reported
0.227296	56650	443698	500348	Major corc	Major corc	TRUE	reported
0.021249	37653	462695	500348	Heart failu	Heart failu	TRUE	reported

0.755351	37653	462695	500348	Heart failu	Heart failu	TRUE	reported
0.516937	37653	462695	500348	Heart failu	Heart failu	TRUE	reported
0.585214	37653	462695	500348	Heart failu	Heart failu	TRUE	reported
0.144866	37653	462695	500348	Heart failu	Heart failu	TRUE	reported
0.502542	37653	462695	500348	Heart failu	Heart failu	TRUE	reported
0.0492	37653	462695	500348	Heart failu	Heart failu	TRUE	reported
0.291453	37653	462695	500348	Heart failu	Heart failu	TRUE	reported
0.151319	37653	462695	500348	Heart failu	Heart failu	TRUE	reported
0.12828	37653	462695	500348	Heart failu	Heart failu	TRUE	reported
0.229128	37653	462695	500348	Heart failu	Heart failu	TRUE	reported
0.657642	37653	462695	500348	Heart failu	Heart failu	TRUE	reported
0.109488	154630	345634	500264	Hypertens	Hypertens	TRUE	reported
0.524976	154630	345634	500264	Hypertens	Hypertens	TRUE	reported
0.412749	154630	345634	500264	Hypertens	Hypertens	TRUE	reported
0.077354	154630	345634	500264	Hypertens	Hypertens	TRUE	reported
0.601212	154630	345634	500264	Hypertens	Hypertens	TRUE	reported
0.133605	154630	345634	500264	Hypertens	Hypertens	TRUE	reported
0.101422	154630	345634	500264	Hypertens	Hypertens	TRUE	reported
0.043243	154630	345634	500264	Hypertens	Hypertens	TRUE	reported
0.379095	154630	345634	500264	Hypertens	Hypertens	TRUE	reported
0.117631	154630	345634	500264	Hypertens	Hypertens	TRUE	reported
0.370012	154630	345634	500264	Hypertens	Hypertens	TRUE	reported
0.794559	154630	345634	500264	Hypertens	Hypertens	TRUE	reported
0.66957	154630	345634	500264	Hypertens	Hypertens	TRUE	reported
0.28819	154630	345634	500264	Hypertens	Hypertens	TRUE	reported
0.170746	154630	345634	500264	Hypertens	Hypertens	TRUE	reported
0.119113	154630	345634	500264	Hypertens	Hypertens	TRUE	reported
0.014918	154630	345634	500264	Hypertens	Hypertens	TRUE	reported
0.098238	154630	345634	500264	Hypertens	Hypertens	TRUE	reported
0.079407	154630	345634	500264	Hypertens	Hypertens	TRUE	reported
0.574509	154630	345634	500264	Hypertens	Hypertens	TRUE	reported
0.566013	154630	345634	500264	Hypertens	Hypertens	TRUE	reported
0.209516	154630	345634	500264	Hypertens	Hypertens	TRUE	reported
0.593665	154630	345634	500264	Hypertens	Hypertens	TRUE	reported
0.578667	154630	345634	500264	Hypertens	Hypertens	TRUE	reported
0.025577	154630	345634	500264	Hypertens	Hypertens	TRUE	reported
0.681635	154630	345634	500264	Hypertens	Hypertens	TRUE	reported
0.213087	154630	345634	500264	Hypertens	Hypertens	TRUE	reported
0.517888	154630	345634	500264	Hypertens	Hypertens	TRUE	reported
0.381009	154630	345634	500264	Hypertens	Hypertens	TRUE	reported
0.375655	154630	345634	500264	Hypertens	Hypertens	TRUE	reported
0.118278	154630	345634	500264	Hypertens	Hypertens	TRUE	reported
0.256687	154630	345634	500264	Hypertens	Hypertens	TRUE	reported
0.866355	154630	345634	500264	Hypertens	Hypertens	TRUE	reported

0.145805	154630	345634	500264	Hypertens	Hypertens	TRUE	reported
0.836599	154630	345634	500264	Hypertens	Hypertens	TRUE	reported
0.794292	154630	345634	500264	Hypertens	Hypertens	TRUE	reported
0.280122	154630	345634	500264	Hypertens	Hypertens	TRUE	reported
0.36195	154630	345634	500264	Hypertens	Hypertens	TRUE	reported
0.357829	154630	345634	500264	Hypertens	Hypertens	TRUE	reported
0.017652	154630	345634	500264	Hypertens	Hypertens	TRUE	reported
0.082671	154630	345634	500264	Hypertens	Hypertens	TRUE	reported
0.4287	154630	345634	500264	Hypertens	Hypertens	TRUE	reported
0.409979	154630	345634	500264	Hypertens	Hypertens	TRUE	reported
0.098855	154630	345634	500264	Hypertens	Hypertens	TRUE	reported
0.082902	154630	345634	500264	Hypertens	Hypertens	TRUE	reported
0.403949	154630	345634	500264	Hypertens	Hypertens	TRUE	reported
0.324152	154630	345634	500264	Hypertens	Hypertens	TRUE	reported
0.379147	154630	345634	500264	Hypertens	Hypertens	TRUE	reported
0.659464	154630	345634	500264	Hypertens	Hypertens	TRUE	reported
0.651274	154630	345634	500264	Hypertens	Hypertens	TRUE	reported
0.2818	154630	345634	500264	Hypertens	Hypertens	TRUE	reported
0.550968	154630	345634	500264	Hypertens	Hypertens	TRUE	reported
0.445422	154630	345634	500264	Hypertens	Hypertens	TRUE	reported
0.798999	154630	345634	500264	Hypertens	Hypertens	TRUE	reported
0.079232	154630	345634	500264	Hypertens	Hypertens	TRUE	reported
0.496932	154630	345634	500264	Hypertens	Hypertens	TRUE	reported
0.260247	154630	345634	500264	Hypertens	Hypertens	TRUE	reported
0.438917	154630	345634	500264	Hypertens	Hypertens	TRUE	reported
0.317993	154630	345634	500264	Hypertens	Hypertens	TRUE	reported
0.313523	154630	345634	500264	Hypertens	Hypertens	TRUE	reported
0.152488	154630	345634	500264	Hypertens	Hypertens	TRUE	reported
0.952977	154630	345634	500264	Hypertens	Hypertens	TRUE	reported
0.82869	154630	345634	500264	Hypertens	Hypertens	TRUE	reported
0.446345	154630	345634	500264	Hypertens	Hypertens	TRUE	reported
0.548106	154630	345634	500264	Hypertens	Hypertens	TRUE	reported
0.149343	154630	345634	500264	Hypertens	Hypertens	TRUE	reported
0.45189	154630	345634	500264	Hypertens	Hypertens	TRUE	reported
0.643235	154630	345634	500264	Hypertens	Hypertens	TRUE	reported
0.186376	154630	345634	500264	Hypertens	Hypertens	TRUE	reported
0.39936	154630	345634	500264	Hypertens	Hypertens	TRUE	reported
0.217825	154630	345634	500264	Hypertens	Hypertens	TRUE	reported
0.275475	154630	345634	500264	Hypertens	Hypertens	TRUE	reported
0.585758	154630	345634	500264	Hypertens	Hypertens	TRUE	reported
0.023783	154630	345634	500264	Hypertens	Hypertens	TRUE	reported
0.255532	154630	345634	500264	Hypertens	Hypertens	TRUE	reported
0.407878	154630	345634	500264	Hypertens	Hypertens	TRUE	reported
0.665515	154630	345634	500264	Hypertens	Hypertens	TRUE	reported

0.807937	154630	345634	500264	Hypertens	Hypertens	TRUE	reported
0.422457	154630	345634	500264	Hypertens	Hypertens	TRUE	reported
0.226638	154630	345634	500264	Hypertens	Hypertens	TRUE	reported
0.297626	154630	345634	500264	Hypertens	Hypertens	TRUE	reported
0.5492	154630	345634	500264	Hypertens	Hypertens	TRUE	reported
0.385548	154630	345634	500264	Hypertens	Hypertens	TRUE	reported
0.19532	154630	345634	500264	Hypertens	Hypertens	TRUE	reported
0.381942	154630	345634	500264	Hypertens	Hypertens	TRUE	reported
0.121298	154630	345634	500264	Hypertens	Hypertens	TRUE	reported
0.185476	154630	345634	500264	Hypertens	Hypertens	TRUE	reported
0.66543	154630	345634	500264	Hypertens	Hypertens	TRUE	reported
0.534647	154630	345634	500264	Hypertens	Hypertens	TRUE	reported
0.375924	154630	345634	500264	Hypertens	Hypertens	TRUE	reported
0.157839	154630	345634	500264	Hypertens	Hypertens	TRUE	reported
0.627674	154630	345634	500264	Hypertens	Hypertens	TRUE	reported
0.10856	154630	345634	500264	Hypertens	Hypertens	TRUE	reported
0.681594	154630	345634	500264	Hypertens	Hypertens	TRUE	reported
0.284123	154630	345634	500264	Hypertens	Hypertens	TRUE	reported
0.463043	154630	345634	500264	Hypertens	Hypertens	TRUE	reported
0.399451	154630	345634	500264	Hypertens	Hypertens	TRUE	reported
0.628799	154630	345634	500264	Hypertens	Hypertens	TRUE	reported
0.038406	154630	345634	500264	Hypertens	Hypertens	TRUE	reported
0.410634	154630	345634	500264	Hypertens	Hypertens	TRUE	reported
0.72833	154630	345634	500264	Hypertens	Hypertens	TRUE	reported
0.500767	154630	345634	500264	Hypertens	Hypertens	TRUE	reported
0.332303	154630	345634	500264	Hypertens	Hypertens	TRUE	reported
0.075997	154630	345634	500264	Hypertens	Hypertens	TRUE	reported
0.055701	154630	345634	500264	Hypertens	Hypertens	TRUE	reported
0.09186	154630	345634	500264	Hypertens	Hypertens	TRUE	reported
0.249921	154630	345634	500264	Hypertens	Hypertens	TRUE	reported
0.440224	154630	345634	500264	Hypertens	Hypertens	TRUE	reported
0.325321	154630	345634	500264	Hypertens	Hypertens	TRUE	reported
0.53533	154630	345634	500264	Hypertens	Hypertens	TRUE	reported
0.904702	154630	345634	500264	Hypertens	Hypertens	TRUE	reported
0.146598	154630	345634	500264	Hypertens	Hypertens	TRUE	reported
0.034989	154630	345634	500264	Hypertens	Hypertens	TRUE	reported
0.187869	154630	345634	500264	Hypertens	Hypertens	TRUE	reported
0.803325	154630	345634	500264	Hypertens	Hypertens	TRUE	reported
0.298223	154630	345634	500264	Hypertens	Hypertens	TRUE	reported
0.719052	154630	345634	500264	Hypertens	Hypertens	TRUE	reported
0.353722	154630	345634	500264	Hypertens	Hypertens	TRUE	reported
0.07924	154630	345634	500264	Hypertens	Hypertens	TRUE	reported
0.264632	154630	345634	500264	Hypertens	Hypertens	TRUE	reported
0.070162	154630	345634	500264	Hypertens	Hypertens	TRUE	reported

0.231241	154630	345634	500264	Hypertens	Hypertens	TRUE	reported
0.450276	154630	345634	500264	Hypertens	Hypertens	TRUE	reported
0.504625	154630	345634	500264	Hypertens	Hypertens	TRUE	reported
0.46642	154630	345634	500264	Hypertens	Hypertens	TRUE	reported
0.296207	154630	345634	500264	Hypertens	Hypertens	TRUE	reported
0.62031	154630	345634	500264	Hypertens	Hypertens	TRUE	reported
0.544844	154630	345634	500264	Hypertens	Hypertens	TRUE	reported
0.717795	154630	345634	500264	Hypertens	Hypertens	TRUE	reported
0.13771	154630	345634	500264	Hypertens	Hypertens	TRUE	reported
0.390933	154630	345634	500264	Hypertens	Hypertens	TRUE	reported
0.155224	154630	345634	500264	Hypertens	Hypertens	TRUE	reported
0.26574	154630	345634	500264	Hypertens	Hypertens	TRUE	reported
0.56906	154630	345634	500264	Hypertens	Hypertens	TRUE	reported
0.539654	154630	345634	500264	Hypertens	Hypertens	TRUE	reported
0.212875	154630	345634	500264	Hypertens	Hypertens	TRUE	reported
0.433695	154630	345634	500264	Hypertens	Hypertens	TRUE	reported
0.627829	154630	345634	500264	Hypertens	Hypertens	TRUE	reported
0.099644	154630	345634	500264	Hypertens	Hypertens	TRUE	reported
0.693556	154630	345634	500264	Hypertens	Hypertens	TRUE	reported
0.088866	154630	345634	500264	Hypertens	Hypertens	TRUE	reported
0.319067	154630	345634	500264	Hypertens	Hypertens	TRUE	reported
0.06431	154630	345634	500264	Hypertens	Hypertens	TRUE	reported
0.120586	154630	345634	500264	Hypertens	Hypertens	TRUE	reported
0.242065	154630	345634	500264	Hypertens	Hypertens	TRUE	reported
0.386299	154630	345634	500264	Hypertens	Hypertens	TRUE	reported
0.234804	154630	345634	500264	Hypertens	Hypertens	TRUE	reported
0.069982	154630	345634	500264	Hypertens	Hypertens	TRUE	reported
0.102127	154630	345634	500264	Hypertens	Hypertens	TRUE	reported
0.357618	154630	345634	500264	Hypertens	Hypertens	TRUE	reported
0.349841	154630	345634	500264	Hypertens	Hypertens	TRUE	reported
0.130405	154630	345634	500264	Hypertens	Hypertens	TRUE	reported
0.566153	154630	345634	500264	Hypertens	Hypertens	TRUE	reported
0.344235	154630	345634	500264	Hypertens	Hypertens	TRUE	reported
0.091241	154630	345634	500264	Hypertens	Hypertens	TRUE	reported
0.384295	154630	345634	500264	Hypertens	Hypertens	TRUE	reported
0.952379	154630	345634	500264	Hypertens	Hypertens	TRUE	reported
0.228081	154630	345634	500264	Hypertens	Hypertens	TRUE	reported
0.036289	154630	345634	500264	Hypertens	Hypertens	TRUE	reported
0.653998	154630	345634	500264	Hypertens	Hypertens	TRUE	reported
0.24735	154630	345634	500264	Hypertens	Hypertens	TRUE	reported
0.573687	154630	345634	500264	Hypertens	Hypertens	TRUE	reported
0.175012	154630	345634	500264	Hypertens	Hypertens	TRUE	reported
0.196874	154630	345634	500264	Hypertens	Hypertens	TRUE	reported
0.49893	154630	345634	500264	Hypertens	Hypertens	TRUE	reported

0.497138	154630	345634	500264	Hypertens	Hypertens	TRUE	reported
0.847964	154630	345634	500264	Hypertens	Hypertens	TRUE	reported
0.404875	154630	345634	500264	Hypertens	Hypertens	TRUE	reported
0.047554	154630	345634	500264	Hypertens	Hypertens	TRUE	reported
0.309216	154630	345634	500264	Hypertens	Hypertens	TRUE	reported
0.039879	154630	345634	500264	Hypertens	Hypertens	TRUE	reported
0.238408	154630	345634	500264	Hypertens	Hypertens	TRUE	reported
0.190683	154630	345634	500264	Hypertens	Hypertens	TRUE	reported
0.629158	154630	345634	500264	Hypertens	Hypertens	TRUE	reported
0.170813	154630	345634	500264	Hypertens	Hypertens	TRUE	reported
0.28243	154630	345634	500264	Hypertens	Hypertens	TRUE	reported
0.740685	154630	345634	500264	Hypertens	Hypertens	TRUE	reported
0.164976	154630	345634	500264	Hypertens	Hypertens	TRUE	reported
0.823985	154630	345634	500264	Hypertens	Hypertens	TRUE	reported
0.250672	154630	345634	500264	Hypertens	Hypertens	TRUE	reported
0.151473	154630	345634	500264	Hypertens	Hypertens	TRUE	reported
0.534515	154630	345634	500264	Hypertens	Hypertens	TRUE	reported
0.240612	154630	345634	500264	Hypertens	Hypertens	TRUE	reported
0.039178	154630	345634	500264	Hypertens	Hypertens	TRUE	reported
0.219525	154630	345634	500264	Hypertens	Hypertens	TRUE	reported
0.762602	154630	345634	500264	Hypertens	Hypertens	TRUE	reported
0.589284	154630	345634	500264	Hypertens	Hypertens	TRUE	reported
0.052922	154630	345634	500264	Hypertens	Hypertens	TRUE	reported
0.074981	154630	345634	500264	Hypertens	Hypertens	TRUE	reported
0.020308	154630	345634	500264	Hypertens	Hypertens	TRUE	reported
0.407391	154630	345634	500264	Hypertens	Hypertens	TRUE	reported
0.676445	154630	345634	500264	Hypertens	Hypertens	TRUE	reported
0.199058	154630	345634	500264	Hypertens	Hypertens	TRUE	reported
0.093422	154630	345634	500264	Hypertens	Hypertens	TRUE	reported
0.077024	154630	345634	500264	Hypertens	Hypertens	TRUE	reported
0.507067	154630	345634	500264	Hypertens	Hypertens	TRUE	reported
0.585727	154630	345634	500264	Hypertens	Hypertens	TRUE	reported
0.364597	154630	345634	500264	Hypertens	Hypertens	TRUE	reported
0.696365	154630	345634	500264	Hypertens	Hypertens	TRUE	reported
0.57958	154630	345634	500264	Hypertens	Hypertens	TRUE	reported
0.64185	154630	345634	500264	Hypertens	Hypertens	TRUE	reported
0.098393	154630	345634	500264	Hypertens	Hypertens	TRUE	reported
0.673216	154630	345634	500264	Hypertens	Hypertens	TRUE	reported
0.212009	154630	345634	500264	Hypertens	Hypertens	TRUE	reported
0.166544	154630	345634	500264	Hypertens	Hypertens	TRUE	reported
0.679986	154630	345634	500264	Hypertens	Hypertens	TRUE	reported
0.221123	154630	345634	500264	Hypertens	Hypertens	TRUE	reported
0.649532	154630	345634	500264	Hypertens	Hypertens	TRUE	reported
0.398294	154630	345634	500264	Hypertens	Hypertens	TRUE	reported

0.644197	154630	345634	500264	Hypertens	Hypertens	TRUE	reported
0.0166	154630	345634	500264	Hypertens	Hypertens	TRUE	reported
0.143565	154630	345634	500264	Hypertens	Hypertens	TRUE	reported
0.40561	154630	345634	500264	Hypertens	Hypertens	TRUE	reported
0.19048	154630	345634	500264	Hypertens	Hypertens	TRUE	reported
0.267776	154630	345634	500264	Hypertens	Hypertens	TRUE	reported
0.068306	154630	345634	500264	Hypertens	Hypertens	TRUE	reported
0.321637	154630	345634	500264	Hypertens	Hypertens	TRUE	reported
0.022784	154630	345634	500264	Hypertens	Hypertens	TRUE	reported
0.441332	154630	345634	500264	Hypertens	Hypertens	TRUE	reported
0.222992	154630	345634	500264	Hypertens	Hypertens	TRUE	reported
0.452627	154630	345634	500264	Hypertens	Hypertens	TRUE	reported
0.428346	154630	345634	500264	Hypertens	Hypertens	TRUE	reported
0.062634	154630	345634	500264	Hypertens	Hypertens	TRUE	reported
0.555606	154630	345634	500264	Hypertens	Hypertens	TRUE	reported
0.586981	154630	345634	500264	Hypertens	Hypertens	TRUE	reported
0.594071	154630	345634	500264	Hypertens	Hypertens	TRUE	reported
0.292622	154630	345634	500264	Hypertens	Hypertens	TRUE	reported
0.394852	154630	345634	500264	Hypertens	Hypertens	TRUE	reported
0.48793	154630	345634	500264	Hypertens	Hypertens	TRUE	reported
0.671971	154630	345634	500264	Hypertens	Hypertens	TRUE	reported
0.16935	154630	345634	500264	Hypertens	Hypertens	TRUE	reported
0.206841	154630	345634	500264	Hypertens	Hypertens	TRUE	reported
0.517076	154630	345634	500264	Hypertens	Hypertens	TRUE	reported
0.14227	154630	345634	500264	Hypertens	Hypertens	TRUE	reported
0.035309	154630	345634	500264	Hypertens	Hypertens	TRUE	reported
0.712826	154630	345634	500264	Hypertens	Hypertens	TRUE	reported
0.092199	154630	345634	500264	Hypertens	Hypertens	TRUE	reported
0.355608	154630	345634	500264	Hypertens	Hypertens	TRUE	reported
0.647406	154630	345634	500264	Hypertens	Hypertens	TRUE	reported
0.108512	154630	345634	500264	Hypertens	Hypertens	TRUE	reported
0.191176	154630	345634	500264	Hypertens	Hypertens	TRUE	reported
0.285972	154630	345634	500264	Hypertens	Hypertens	TRUE	reported
0.490265	154630	345634	500264	Hypertens	Hypertens	TRUE	reported
0.014662	154630	345634	500264	Hypertens	Hypertens	TRUE	reported
0.57652	154630	345634	500264	Hypertens	Hypertens	TRUE	reported
0.033073	154630	345634	500264	Hypertens	Hypertens	TRUE	reported
0.217404	154630	345634	500264	Hypertens	Hypertens	TRUE	reported
0.551877	154630	345634	500264	Hypertens	Hypertens	TRUE	reported
0.022435	154630	345634	500264	Hypertens	Hypertens	TRUE	reported
0.63704	154630	345634	500264	Hypertens	Hypertens	TRUE	reported
0.209034	154630	345634	500264	Hypertens	Hypertens	TRUE	reported
0.432705	154630	345634	500264	Hypertens	Hypertens	TRUE	reported
0.242386	154630	345634	500264	Hypertens	Hypertens	TRUE	reported

0.750748	154630	345634	500264	Hypertens	Hypertens	TRUE	reported
0.194192	154630	345634	500264	Hypertens	Hypertens	TRUE	reported
0.376054	154630	345634	500264	Hypertens	Hypertens	TRUE	reported
0.125552	154630	345634	500264	Hypertens	Hypertens	TRUE	reported
0.055091	154630	345634	500264	Hypertens	Hypertens	TRUE	reported
0.073759	154630	345634	500264	Hypertens	Hypertens	TRUE	reported
0.162428	154630	345634	500264	Hypertens	Hypertens	TRUE	reported
0.033552	154630	345634	500264	Hypertens	Hypertens	TRUE	reported
0.034338	154630	345634	500264	Hypertens	Hypertens	TRUE	reported
0.613094	154630	345634	500264	Hypertens	Hypertens	TRUE	reported
0.384536	154630	345634	500264	Hypertens	Hypertens	TRUE	reported
0.185375	154630	345634	500264	Hypertens	Hypertens	TRUE	reported
0.290956	154630	345634	500264	Hypertens	Hypertens	TRUE	reported
0.685915	154630	345634	500264	Hypertens	Hypertens	TRUE	reported
0.212573	154630	345634	500264	Hypertens	Hypertens	TRUE	reported
0.171592	154630	345634	500264	Hypertens	Hypertens	TRUE	reported
0.538786	154630	345634	500264	Hypertens	Hypertens	TRUE	reported
0.429306	31666	416171	447837	Myocardia	Myocardia	TRUE	reported
0.036643	31666	416171	447837	Myocardia	Myocardia	TRUE	reported
0.583276	31666	416171	447837	Myocardia	Myocardia	TRUE	reported
0.341802	31666	416171	447837	Myocardia	Myocardia	TRUE	reported
0.168101	31666	416171	447837	Myocardia	Myocardia	TRUE	reported
0.215394	31666	416171	447837	Myocardia	Myocardia	TRUE	reported
0.799386	31666	416171	447837	Myocardia	Myocardia	TRUE	reported
0.072856	31666	416171	447837	Myocardia	Myocardia	TRUE	reported
0.083892	31666	416171	447837	Myocardia	Myocardia	TRUE	reported
0.365917	31666	416171	447837	Myocardia	Myocardia	TRUE	reported
0.448374	31666	416171	447837	Myocardia	Myocardia	TRUE	reported
0.666787	31666	416171	447837	Myocardia	Myocardia	TRUE	reported
0.095283	31666	416171	447837	Myocardia	Myocardia	TRUE	reported
0.790377	31666	416171	447837	Myocardia	Myocardia	TRUE	reported
0.667249	31666	416171	447837	Myocardia	Myocardia	TRUE	reported
0.143756	31666	416171	447837	Myocardia	Myocardia	TRUE	reported
0.218259	31666	416171	447837	Myocardia	Myocardia	TRUE	reported
0.786718	31666	416171	447837	Myocardia	Myocardia	TRUE	reported
0.18031	31666	416171	447837	Myocardia	Myocardia	TRUE	reported
0.12643	31666	416171	447837	Myocardia	Myocardia	TRUE	reported
0.528965	31666	416171	447837	Myocardia	Myocardia	TRUE	reported
0.331139	31666	416171	447837	Myocardia	Myocardia	TRUE	reported
0.451334	31666	416171	447837	Myocardia	Myocardia	TRUE	reported
0.256216	31666	416171	447837	Myocardia	Myocardia	TRUE	reported
0.064325	31666	416171	447837	Myocardia	Myocardia	TRUE	reported
0.327546	31666	416171	447837	Myocardia	Myocardia	TRUE	reported
0.183276	31666	416171	447837	Myocardia	Myocardia	TRUE	reported

0.448216	31666	416171	447837	Myocardia	Myocardia	TRUE	reported
0.389978	31666	416171	447837	Myocardia	Myocardia	TRUE	reported
0.01071	31666	416171	447837	Myocardia	Myocardia	TRUE	reported
0.045766	31666	416171	447837	Myocardia	Myocardia	TRUE	reported
0.192744	31666	416171	447837	Myocardia	Myocardia	TRUE	reported
0.3262	31666	416171	447837	Myocardia	Myocardia	TRUE	reported
0.070058	31666	416171	447837	Myocardia	Myocardia	TRUE	reported
0.246008	31666	416171	447837	Myocardia	Myocardia	TRUE	reported
0.128118	31666	416171	447837	Myocardia	Myocardia	TRUE	reported
0.103529	31666	416171	447837	Myocardia	Myocardia	TRUE	reported
0.419164	31666	416171	447837	Myocardia	Myocardia	TRUE	reported
0.096912	31666	416171	447837	Myocardia	Myocardia	TRUE	reported
0.69389	31666	416171	447837	Myocardia	Myocardia	TRUE	reported
0.414924	31666	416171	447837	Myocardia	Myocardia	TRUE	reported
0.267746	31666	416171	447837	Myocardia	Myocardia	TRUE	reported
0.396681	31666	416171	447837	Myocardia	Myocardia	TRUE	reported
0.785321	31666	416171	447837	Myocardia	Myocardia	TRUE	reported
0.855772	31666	416171	447837	Myocardia	Myocardia	TRUE	reported
0.520585	31666	416171	447837	Myocardia	Myocardia	TRUE	reported
0.585853	31666	416171	447837	Myocardia	Myocardia	TRUE	reported
0.084557	31666	416171	447837	Myocardia	Myocardia	TRUE	reported
0.262009	31666	416171	447837	Myocardia	Myocardia	TRUE	reported
0.723912	31666	416171	447837	Myocardia	Myocardia	TRUE	reported
0.381622	31666	416171	447837	Myocardia	Myocardia	TRUE	reported
0.636146	31666	416171	447837	Myocardia	Myocardia	TRUE	reported
0.028969	31666	416171	447837	Myocardia	Myocardia	TRUE	reported
0.7001	31666	416171	447837	Myocardia	Myocardia	TRUE	reported
0.058071	31666	416171	447837	Myocardia	Myocardia	TRUE	reported
0.56629	31666	416171	447837	Myocardia	Myocardia	TRUE	reported
0.707531	31666	416171	447837	Myocardia	Myocardia	TRUE	reported
0.211189	31666	416171	447837	Myocardia	Myocardia	TRUE	reported
0.229951	31666	416171	447837	Myocardia	Myocardia	TRUE	reported
0.053352	31666	416171	447837	Myocardia	Myocardia	TRUE	reported
0.399726	31666	416171	447837	Myocardia	Myocardia	TRUE	reported
0.152941	31666	416171	447837	Myocardia	Myocardia	TRUE	reported

Table S6. The Reverse MR result Between Retina and cardiovascular disorders.

outcome	exposure	nsnp	b_Inverse	se_Inverse	pval_Inver	FDR	egger_inte	se_egger_i	pval_egger
INL_RPE_t	Heart failu	10	-0.13766	0.042628	0.001241	0.0817568	-0.01093	0.009597	0.287837
INL_RPE_t	Atrial fibril	87	-0.03321	0.0106	0.001732	0.0817568	0.000404	0.002032	0.842894
ISOS_RPE_	Atrial fibril	96	-0.02846	0.009468	0.00265	0.0817568	0.003701	0.001785	0.040872
INL_RPE_t	Atrial fibril	88	-0.03136	0.010543	0.002935	0.0817568	0.000353	0.002026	0.862199
mean_of_	Atrial fibril	94	-0.03121	0.010531	0.00304	0.0817568	0.002899	0.00198	0.146563
INL_ELM_t	Heart failu	10	-0.13658	0.046197	0.003111	0.0817568	-0.01724	0.00941	0.104267
ISOS_RPE_	Cardiac arr	62	-0.04908	0.016775	0.003434	0.0817568	0.00358	0.002332	0.130014
ISOS_RPE_	Atrial fibril	96	-0.0276	0.009528	0.003772	0.0817568	0.00357	0.0018	0.050203
ISOS_RPE_	Cardiac arr	62	-0.04975	0.01739	0.004229	0.0817568	0.003789	0.002416	0.122049
INL_ELM_t	Major corc	59	-0.04494	0.01674	0.007265	0.1177164	0.00031	0.002516	0.90227
ISOS_RPE_	Atrial fibril	96	-0.02575	0.00962	0.007442	0.1177164	0.002638	0.001835	0.153792
INL_RPE_t	Heart failu	10	-0.1284	0.051765	0.013124	0.190296	-0.01581	0.011251	0.197477
ISOS_RPE_	Cardiac arr	62	-0.04143	0.017062	0.015171	0.2030637	0.001878	0.002406	0.438127
INL_RPE_t	Heart failu	10	-0.11669	0.051508	0.023483	0.287165	-0.01445	0.011411	0.241129
mean_of_	Cardiac arr	59	-0.04197	0.018694	0.024756	0.287165	0.004541	0.002539	0.079025
GCIPL_thic	Atrial fibril	91	0.022278	0.010459	0.033179	0.360827	-0.00307	0.001982	0.125286
Disc_diam	Atrial fibril	93	-0.02208	0.010866	0.04217	0.3893334	0.002201	0.002055	0.287104
INL_RPE_t	Atrial fibril	89	-0.0202	0.01012	0.045947	0.3893334	0.000724	0.001932	0.708923
ISOS_RPE_	Myocardia	49	-0.02596	0.013103	0.047607	0.3893334	-0.00388	0.002694	0.156293
ISOS_RPE_	Atrial fibril	96	-0.01963	0.009946	0.04845	0.3893334	0.002331	0.001902	0.223541
INL_thickn	Major corc	62	-0.03183	0.016136	0.048552	0.3893334	-0.00028	0.002501	0.911539
INL_ELM_t	Heart failu	10	-0.07528	0.038646	0.051435	0.3893334	-0.01204	0.008704	0.204034
INL_thickn	STROKE	13	-0.07692	0.039744	0.052945	0.3893334	0.000108	0.007927	0.989341
mean_of_	Hypertens	195	-0.02726	0.014174	0.054487	0.3893334	0.000484	0.001781	0.785876
INL_ELM_t	Major corc	59	-0.03172	0.016683	0.057245	0.3893334	0.002122	0.002531	0.405324
overall_ma	Heart failu	10	-0.07152	0.038286	0.061768	0.3893334	-0.00605	0.008758	0.509293
ISOS_RPE_	Cardiac arr	62	-0.03486	0.018715	0.062541	0.3893334	0.002625	0.002631	0.322329
ELM_ISOS_	Myocardia	49	0.02856	0.015341	0.062651	0.3893334	0.002693	0.0032	0.404177
INL_RPE_t	Cardiac arr	62	-0.0361	0.019669	0.06643	0.398582	0.001085	0.002785	0.69818
ELM_ISOS_	Heart failu	10	-0.09329	0.051579	0.070506	0.4070764	-0.02365	0.009308	0.034695
VCDR_regr	Cardiac arr	59	0.035887	0.019984	0.072525	0.4070764	0.001107	0.002791	0.693193
INL_ELM_t	Heart failu	9	-0.07977	0.04632	0.085059	0.4576841	-0.01232	0.010526	0.280024
INL_RPE_t	Cardiac arr	62	-0.03574	0.020918	0.08753	0.4576841	0.002398	0.002949	0.419294
overall_av	STROKE	13	-0.06716	0.039545	0.089455	0.4576841	-0.00612	0.007885	0.454058
INL_ELM_t	Heart failu	9	-0.0831	0.04978	0.095064	0.4576841	-0.01215	0.011487	0.325258
VCDR	Cardiac arr	59	0.032299	0.019699	0.101078	0.4576841	0.00186	0.002744	0.50055
ISOS_RPE_	Myocardia	49	-0.02181	0.013327	0.101687	0.4576841	-0.00361	0.00275	0.196299
GCIPL_thic	Major corc	65	-0.02487	0.015205	0.101969	0.4576841	-0.00118	0.002371	0.619434
INL_RPE_t	Heart failu	10	-0.06208	0.038028	0.102584	0.4576841	-0.00454	0.008704	0.616048
ISOS_RPE_	STROKE	13	-0.09048	0.056954	0.112148	0.4812215	-0.0042	0.011794	0.728782
ISOS_RPE_	Major corc	65	-0.02225	0.014055	0.113391	0.4812215	0.000137	0.002178	0.949945
RNFL_thicl	Heart failu	10	0.059931	0.038382	0.118428	0.4906289	0.007358	0.008786	0.426612

INL_RPE_t	Cardiac arı	62	-0.03129	0.020322	0.123632	0.5002767	0.00262	0.002861	0.363353
ISOS_RPE_	Heart failu	10	-0.0579	0.038847	0.136096	0.5341809	0.013115	0.008887	0.178243
ISOS_RPE_	Major corc	65	-0.02332	0.015829	0.14064	0.5341809	1.69E-05	0.002472	0.994581
overall_av	Hypertens	195	-0.02113	0.014458	0.143821	0.5341809	0.000671	0.001816	0.712235
overall_av	Cardiac arı	62	0.024666	0.016895	0.14429	0.5341809	0.000288	0.002395	0.904542
ISOS_RPE_	Heart failu	10	-0.05448	0.038817	0.160439	0.5785698	0.011085	0.008881	0.247233
Disc_diam	Hypertens	195	-0.02026	0.014521	0.162931	0.5785698	-0.00068	0.001824	0.709868
INL_ELM_t	Atrial fibril	96	-0.01589	0.01182	0.178877	0.5914483	-0.00074	0.002278	0.747135
ELM_ISOS_	STROKE	13	0.053371	0.039707	0.178911	0.5914483	-0.00263	0.007919	0.746437
Disc_diam	Cardiac arı	58	-0.02643	0.019805	0.181995	0.5914483	0.004107	0.002739	0.139405
INL_ELM_t	Major corc	59	-0.0222	0.01667	0.182931	0.5914483	0.002815	0.00255	0.274394
ISOS_RPE_	Major corc	65	-0.01957	0.014829	0.186892	0.5914483	0.000498	0.002315	0.830274
INL_thickn	Atrial fibril	86	-0.01444	0.010945	0.186952	0.5914483	-0.00437	0.002064	0.037103
ELM_ISOS_	Myocardia	48	0.018505	0.014136	0.190507	0.5919326	0.00468	0.002816	0.103347
ISOS_RPE_	Myocardia	49	-0.01661	0.012893	0.197691	0.6034763	-0.00109	0.002681	0.685919
INL_RPE_t	STROKE	12	-0.06067	0.048194	0.208052	0.624155	-0.00553	0.010008	0.592399
overall_ma	STROKE	13	-0.04915	0.039529	0.213682	0.6301817	-0.00436	0.007885	0.591008
INL_RPE_t	Major corc	65	-0.02222	0.01807	0.218816	0.634565	-0.00043	0.002823	0.878106
INL_ELM_t	Atrial fibril	96	-0.01305	0.011303	0.248167	0.6980296	3.22E-05	0.002179	0.988237
overall_ma	Atrial fibril	90	-0.01169	0.010174	0.250491	0.6980296	-0.00137	0.00199	0.494471
INL_ELM_t	Myocardia	49	0.018383	0.016073	0.252735	0.6980296	-0.00088	0.003375	0.795133
ISOS_RPE_	Major corc	65	-0.01587	0.014069	0.259164	0.7046029	0.000254	0.00218	0.907707
VCDR_regı	STROKE	13	-0.04564	0.042599	0.283962	0.7601446	0.007085	0.008488	0.421635
ELM_ISOS_	STROKE	13	0.040383	0.039623	0.308118	0.7786596	-0.00286	0.007902	0.723821
ISOS_RPE_	STROKE	13	-0.04498	0.044735	0.314689	0.7786596	-0.00506	0.009192	0.592878
Disc_diam	Major corc	65	-0.01483	0.014769	0.31523	0.7786596	-0.00231	0.002289	0.316441
VCDR	STROKE	13	-0.04266	0.042581	0.316368	0.7786596	0.008037	0.008485	0.36387
overall_ma	Major corc	65	-0.01677	0.016764	0.31718	0.7786596	0.000637	0.002618	0.808558
INL_RPE_t	STROKE	13	-0.05221	0.052252	0.317729	0.7786596	-0.00109	0.010879	0.922196
INL_ELM_t	Cardiac arı	62	-0.02139	0.021782	0.325994	0.7827588	-0.00015	0.003087	0.961542
ISOS_RPE_	Heart failu	10	-0.04044	0.041539	0.330224	0.7827588	0.019301	0.008751	0.058482
ELM_ISOS_	Cardiac arı	62	0.019274	0.019905	0.332897	0.7827588	0.002108	0.002809	0.45597
VCDR	Hypertens	195	-0.01516	0.015922	0.340933	0.790965	-0.00274	0.001991	0.170059
Disc_diam	Myocardia	49	-0.01315	0.014195	0.354291	0.8111401	-0.00521	0.002884	0.077441
INL_RPE_t	Cardiac arı	62	-0.01865	0.020481	0.362564	0.8115068	0.001752	0.002894	0.547187
mean_of_	Myocardia	49	-0.01298	0.014324	0.365008	0.8115068	-0.00666	0.002849	0.023713
ELM_ISOS_	Cardiac arı	62	0.018521	0.020592	0.368443	0.8115068	0.000897	0.002917	0.759664
ISOS_RPE_	Myocardia	49	-0.01124	0.012905	0.38396	0.8197543	-0.00072	0.002683	0.790736
Disc_diam	STROKE	13	-0.03609	0.04205	0.390734	0.8197543	0.004419	0.00838	0.608482
VCDR_regı	Hypertens	195	-0.0134	0.015803	0.39659	0.8197543	-0.00283	0.001976	0.153489
VCDR_regı	Myocardia	48	-0.0132	0.015643	0.39866	0.8197543	0.000314	0.003262	0.923744
ELM_ISOS_	Myocardia	48	0.012007	0.014258	0.39972	0.8197543	0.004427	0.002851	0.127332
INL_ELM_t	Cardiac arı	62	-0.0177	0.021046	0.400455	0.8197543	0.00044	0.002983	0.883149
mean_of_	STROKE	13	-0.0345	0.041774	0.408914	0.8273370	6.85E-05	0.008325	0.993583

INL_ELM_t	Cardiac arr	59	-0.015	0.018657	0.421541	0.8277105	0.001038	0.002672	0.69904
INL_ELM_t	STROKE	13	-0.03542	0.044372	0.424745	0.8277105	0.000859	0.00924	0.92758
mean_of_	Heart failu	9	-0.03651	0.046062	0.427988	0.8277105	-0.01208	0.01049	0.287254
ELM_ISOS_	Myocardia	48	0.011592	0.014651	0.428808	0.8277105	0.003085	0.003064	0.319249
GCIPL_thic	Heart failu	10	-0.03079	0.039263	0.432883	0.8277105	0.002375	0.009496	0.808849
INL_ELM_t	Myocardia	47	0.01121	0.0148	0.448805	0.8379287	0.004455	0.003014	0.146342
INL_RPE_t	Hypertens	195	0.009839	0.013021	0.44987	0.8379287	-0.00262	0.001626	0.10832
overall_av	Atrial fibril	96	0.008101	0.010788	0.452674	0.8379287	-0.00044	0.002079	0.832848
INL_ELM_t	Atrial fibril	87	-0.00749	0.010319	0.467925	0.8509923	0.001037	0.002057	0.615316
ISOS_RPE_	Hypertens	195	0.011185	0.015464	0.469513	0.8509923	-0.00259	0.001935	0.181723
INL_thickn	Heart failu	9	-0.0378	0.05386	0.482846	0.8563017	-0.01144	0.012067	0.374649
VCDR	Myocardia	48	-0.01079	0.015399	0.483444	0.8563017	-0.00027	0.003211	0.932968
ELM_ISOS_	Cardiac arr	60	0.011965	0.017221	0.487206	0.8563017	0.000754	0.002466	0.760924
ISOS_RPE_	Heart failu	10	-0.02642	0.038563	0.493212	0.858189	0.013256	0.008824	0.171429
RNFL_thicl	Atrial fibril	96	0.006931	0.011078	0.53156	0.9071014	0.003919	0.002097	0.064824
INL_RPE_t	Myocardia	49	0.009074	0.014564	0.53329	0.9071014	-0.00237	0.003041	0.438812
INL_ELM_t	Hypertens	195	0.008494	0.01388	0.540575	0.9071014	-0.0023	0.001737	0.186178
overall_ma	Cardiac arr	62	-0.01069	0.017541	0.542176	0.9071014	0.00164	0.002478	0.510671
ELM_ISOS_	Heart failu	10	-0.02397	0.040378	0.552715	0.9159280	-0.01092	0.009007	0.26006
overall_av	Major corc	59	0.009895	0.016923	0.558749	0.9171914	0.001336	0.002709	0.623692
GCIPL_thic	Cardiac arr	62	0.009428	0.016631	0.570769	0.9281672	-0.00255	0.002334	0.278675
mean_of_	Major corc	65	-0.00818	0.014672	0.577216	0.9299589	-0.00324	0.002274	0.159845
ELM_ISOS_	Major corc	65	-0.00867	0.016302	0.594973	0.9300517	0.002987	0.002519	0.24013
VCDR	Heart failu	10	0.028623	0.055373	0.605215	0.9300517	-0.00351	0.013384	0.799795
GCIPL_thic	Myocardia	49	-0.00771	0.015142	0.610622	0.9300517	0.001486	0.003175	0.641885
ISOS_RPE_	Hypertens	195	0.007062	0.014138	0.617423	0.9300517	-0.00166	0.001773	0.350095
INL_thickn	Hypertens	195	0.00867	0.017388	0.618034	0.9300517	0.000307	0.002185	0.888393
ISOS_RPE_	Hypertens	195	0.007609	0.015461	0.62261	0.9300517	-0.00286	0.001932	0.140582
INL_RPE_t	Myocardia	48	-0.00646	0.013922	0.642548	0.9300517	0.00087	0.002936	0.768245
ELM_ISOS_	Heart failu	10	-0.02228	0.048339	0.644867	0.9300517	-0.01177	0.010974	0.314887
ELM_ISOS_	STROKE	13	0.017804	0.039373	0.65113	0.9300517	-0.00521	0.007853	0.520762
INL_ELM_t	STROKE	13	-0.01876	0.041877	0.654254	0.9300517	-0.0089	0.008299	0.306332
ELM_ISOS_	Atrial fibril	95	0.004319	0.009792	0.659194	0.9300517	-0.00059	0.001893	0.754616
overall_av	Heart failu	10	0.01689	0.038316	0.659351	0.9300517	-0.02195	0.008766	0.036677
INL_ELM_t	STROKE	13	-0.01785	0.041211	0.664913	0.9300517	-0.00512	0.008444	0.556679
INL_RPE_t	Major corc	65	-0.00637	0.015134	0.673823	0.9300517	-0.00323	0.002329	0.170583
Disc_diam	Heart failu	9	-0.01903	0.045879	0.678356	0.9300517	-0.00664	0.011116	0.569178
ELM_ISOS_	Major corc	65	-0.00673	0.01642	0.682057	0.9300517	0.003077	0.002535	0.229507
ELM_ISOS_	Major corc	65	0.00658	0.016414	0.688533	0.9300517	0.001897	0.002553	0.460113
VCDR_regı	Heart failu	10	0.021994	0.055729	0.693092	0.9300517	1.48E-05	0.013528	0.999153
ELM_ISOS_	STROKE	13	-0.01498	0.039364	0.703597	0.9300517	-0.00593	0.00785	0.46622
ISOS_RPE_	STROKE	13	-0.01739	0.04672	0.709772	0.9300517	-0.00922	0.009324	0.343832
RNFL_thicl	Hypertens	195	-0.00514	0.013911	0.71174	0.9300517	0.003283	0.001732	0.059517
ELM_ISOS_	Cardiac arr	62	0.006533	0.017787	0.713411	0.9300517	0.001965	0.002508	0.43649

INL_ELM_t	STROKE	13	-0.01729	0.047531	0.715973	0.9300517	0.002258	0.009879	0.823413
ELM_ISOS_	Hypertens	195	0.005344	0.014716	0.716487	0.9300517	0.000826	0.001849	0.655515
GCIPL_thic	Hypertens	195	-0.00569	0.015753	0.717927	0.9300517	-0.00072	0.001979	0.7169
overall_av	Myocardia	49	0.00532	0.01486	0.720339	0.9300517	0.003168	0.003089	0.310317
ELM_ISOS_	Major corc	65	0.004921	0.01381	0.721592	0.9300517	-9.6E-05	0.00214	0.964495
ELM_ISOS_	Atrial fibril	96	0.003359	0.009831	0.73256	0.9320281	0.0006	0.001895	0.752198
GCIPL_thic	STROKE	12	-0.01549	0.045545	0.733838	0.9320281	0.007961	0.008896	0.391861
INL_RPE_t	STROKE	13	-0.01553	0.048154	0.747079	0.9419697	-0.007	0.009806	0.490258
INL_RPE_t	Hypertens	195	0.00535	0.017402	0.758512	0.9495039	-0.00222	0.002181	0.310686
overall_ma	Myocardia	49	0.00423	0.014348	0.768128	0.9546727	0.003597	0.002969	0.231703
INL_ELM_t	Hypertens	195	-0.00413	0.014427	0.774897	0.9562561	-0.00203	0.001807	0.261912
RNFL_thicl	Cardiac arı	61	0.004845	0.017929	0.786974	0.9603566	0.001862	0.002548	0.467804
INL_ELM_t	Atrial fibril	96	-0.00295	0.011189	0.791978	0.9603566	-0.00051	0.002157	0.8149
INL_RPE_t	Myocardia	48	-0.00369	0.014196	0.794778	0.9603566	0.001589	0.002944	0.592011
ISOS_RPE_	Hypertens	195	0.003678	0.015079	0.807318	0.968782	-0.00345	0.001879	0.067493
VCDR	Atrial fibril	87	-0.00251	0.010923	0.81833	0.9746756	0.000357	0.002107	0.865779
ELM_ISOS_	Atrial fibril	96	0.002197	0.010381	0.83235	0.9746756	-0.00029	0.002001	0.886545
INL_ELM_t	Cardiac arı	62	-0.00415	0.020515	0.839837	0.9746756	-0.00026	0.002908	0.927706
RNFL_thicl	Myocardia	49	0.002575	0.013075	0.84388	0.9746756	0.0017	0.002736	0.537474
INL_RPE_t	Major corc	59	-0.00328	0.016903	0.846215	0.9746756	5.04E-05	0.002592	0.984557
ELM_ISOS_	Atrial fibril	96	-0.00187	0.009866	0.849301	0.9746756	-0.00156	0.001896	0.412042
INL_ELM_t	Hypertens	195	0.00307	0.016928	0.856088	0.9746756	-0.00125	0.002126	0.55583
INL_RPE_t	Hypertens	195	0.002983	0.017218	0.862477	0.9746756	-0.00294	0.002154	0.174396
INL_thickn	Myocardia	49	-0.00306	0.018573	0.869032	0.9746756	0.002197	0.00389	0.57487
INL_RPE_t	Hypertens	195	-0.00242	0.015387	0.875054	0.9746756	-0.0033	0.001919	0.087169
ELM_ISOS_	Hypertens	195	0.002096	0.013484	0.876448	0.9746756	-0.00033	0.001694	0.846317
VCDR_regı	Major corc	64	-0.0026	0.01716	0.879486	0.9746756	0.00026	0.002657	0.922245
ELM_ISOS_	Heart failu	10	0.00556	0.038461	0.88505	0.9746756	-0.00936	0.008802	0.318771
ELM_ISOS_	Hypertens	195	0.00218	0.016857	0.897119	0.9817533	0.000506	0.002118	0.811562
INL_thickn	Cardiac arı	49	-0.00258	0.021244	0.903272	0.9823078	-0.00225	0.002801	0.426693
INL_ELM_t	Major corc	65	-0.00189	0.017824	0.915562	0.9894892	-0.00299	0.002759	0.283168
ISOS_RPE_	STROKE	13	-0.00302	0.045668	0.94731	0.992499	-0.00898	0.009118	0.34594
INL_RPE_t	STROKE	13	-0.00289	0.046671	0.950675	0.992499	-0.00955	0.009284	0.325716
overall_ma	Hypertens	195	-0.00099	0.016173	0.951172	0.992499	-0.0011	0.002031	0.58803
INL_RPE_t	Major corc	57	-0.001	0.017255	0.953931	0.992499	0.000915	0.002628	0.729026
INL_ELM_t	Hypertens	195	0.00079	0.016543	0.961905	0.992499	-0.00163	0.002076	0.434294
INL_ELM_t	Myocardia	49	0.000647	0.014766	0.96503	0.992499	0.002593	0.00308	0.404057
INL_RPE_t	Myocardia	49	0.000553	0.015066	0.970702	0.992499	0.002766	0.00314	0.382801
RNFL_thicl	Major corc	65	-0.00048	0.014576	0.973815	0.992499	0.000723	0.002275	0.751769
ELM_ISOS_	Hypertens	195	0.000436	0.014202	0.97549	0.992499	-0.00029	0.001785	0.868896
VCDR_regı	Atrial fibril	86	-0.00019	0.011324	0.986794	0.992499	-0.00095	0.002138	0.657275
INL_ELM_t	Myocardia	48	0.000157	0.014682	0.991442	0.992499	0.00235	0.003035	0.442721
VCDR	Major corc	64	0.000165	0.017013	0.992269	0.992499	-0.00056	0.002634	0.832065
RNFL_thicl	STROKE	13	-0.00037	0.039615	0.992499	0.992499	-0.0075	0.007902	0.363146

Q_heterog	Q_pval_he	Significance
11.13808	0.266363	Suggestive
107.6895	0.056815	Probable
102.7094	0.276617	Probable
108.615	0.058286	Probable
108.6076	0.128343	Probable
13.04881	0.16041	Suggestive
67.02353	0.27817	Probable
103.8314	0.251468	Probable
71.89914	0.160355	Probable
76.1075	0.055578	Suggestive
107.3492	0.182026	Probable
16.22469	0.062335	Suggestive
70.20191	0.196489	Probable
16.00657	0.066744	Suggestive
68.41665	0.164598	Probable
112.2471	0.056236	Probable
111.9713	0.076956	Probable
106.0924	0.091825	Probable
51.0848	0.353424	Suggestive
116.8131	0.06389	Probable
77.76791	0.072572	Probable
9.297589	0.41027	No evidence
5.871537	0.922419	No evidence
244.1975	0.008425	No evidence
74.88103	0.067144	No evidence
8.406076	0.493797	No evidence
85.98694	0.019244	No evidence
70.40516	0.019213	No evidence
94.5102	0.003836	No evidence
16.47534	0.057595	No evidence
74.20845	0.074294	No evidence
10.34619	0.241564	No evidence
105.6337	0.000343	No evidence
11.42928	0.492531	No evidence
11.92121	0.154754	No evidence
72.17101	0.099843	No evidence
51.92791	0.323438	No evidence
77.89539	0.113702	No evidence
4.929001	0.840453	No evidence
24.98784	0.014881	No evidence
55.79904	0.757646	No evidence
7.744912	0.560043	No evidence

99.35351	0.001396	No evidence
8.75712	0.45999	No evidence
83.62993	0.050347	No evidence
284.2793	2.54E-05	No evidence
69.76515	0.206632	No evidence
6.754094	0.662704	No evidence
252.954	0.002826	No evidence
163.7829	1.5E-05	No evidence
8.352811	0.756987	No evidence
73.55108	0.069114	No evidence
76.13168	0.055368	No evidence
72.12142	0.227256	No evidence
105.7545	0.063226	No evidence
53.57669	0.236652	No evidence
43.75031	0.647513	No evidence
15.53763	0.159183	No evidence
7.669462	0.810398	No evidence
108.4742	0.000437	No evidence
147.5447	0.000449	No evidence
109.8182	0.066533	No evidence
77.72779	0.004238	No evidence
59.5465	0.634517	No evidence
10.40276	0.580672	No evidence
7.728008	0.806008	No evidence
15.14211	0.233757	No evidence
55.55432	0.765	No evidence
10.33922	0.586227	No evidence
93.53446	0.009431	No evidence
20.93532	0.051336	No evidence
115.634	3.05E-05	No evidence
10.62112	0.302576	No evidence
96.05787	0.002797	No evidence
296.5816	2.94E-06	No evidence
52.67866	0.297917	No evidence
104.1036	0.000488	No evidence
54.35695	0.24523	No evidence
103.2255	0.000595	No evidence
46.08283	0.551717	No evidence
6.322785	0.898944	No evidence
291.9107	6.79E-06	No evidence
60.05265	0.095779	No evidence
54.27984	0.216742	No evidence
106.3368	0.000292	No evidence
7.256635	0.840198	No evidence

76.925	0.048841	No evidence
15.05952	0.238198	No evidence
9.29021	0.318409	No evidence
62.1461	0.068455	No evidence
9.582795	0.385303	No evidence
59.60405	0.085879	No evidence
234.0329	0.026153	No evidence
136.8552	0.003209	No evidence
104.9261	0.080901	No evidence
316.406	6.56E-08	No evidence
13.80844	0.086897	No evidence
58.24046	0.125985	No evidence
69.25634	0.16979	No evidence
8.28745	0.505458	No evidence
143.8704	0.000906	No evidence
63.80177	0.063015	No evidence
266.0444	0.000457	No evidence
75.28923	0.103138	No evidence
9.960756	0.353672	No evidence
77.2131	0.046639	No evidence
68.54218	0.236881	No evidence
54.80975	0.786725	No evidence
87.66846	0.02644	No evidence
16.1614	0.063587	No evidence
68.86191	0.025764	No evidence
272.945	0.00016	No evidence
407.1123	3.22E-17	No evidence
316.8204	6.04E-08	No evidence
54.50903	0.210504	No evidence
14.45889	0.106912	No evidence
9.22421	0.68367	No evidence
13.21287	0.353761	No evidence
110.5979	0.11628	No evidence
8.342875	0.499996	No evidence
12.82677	0.381757	No evidence
77.27483	0.123264	No evidence
9.095586	0.334297	No evidence
89.3171	0.02001	No evidence
90.39155	0.016608	No evidence
16.3557	0.059815	No evidence
11.39329	0.495548	No evidence
16.32056	0.176991	No evidence
262.3792	0.000778	No evidence
78.07451	0.069371	No evidence

17.60174	0.128329	No evidence
297.2632	2.59E-06	No evidence
342.1263	2.79E-10	No evidence
65.42795	0.04783	No evidence
57.57078	0.701604	No evidence
112.7202	0.103652	No evidence
13.3798	0.269228	No evidence
17.57203	0.129317	No evidence
405.307	5.2E-17	No evidence
61.06767	0.097562	No evidence
282.1898	3.61E-05	No evidence
76.41839	0.074922	No evidence
149.5158	0.000305	No evidence
57.13691	0.147714	No evidence
305.0913	6.02E-07	No evidence
102.9595	0.102608	No evidence
127.8976	0.013787	No evidence
104.4881	0.000447	No evidence
50.48764	0.375449	No evidence
75.12939	0.064652	No evidence
115.4447	0.075497	No evidence
382.716	1.79E-14	No evidence
398.1946	3.38E-16	No evidence
101.2137	1.14E-05	No evidence
321.8135	2.18E-08	No evidence
245.337	0.007353	No evidence
81.31357	0.060105	No evidence
6.964577	0.640808	No evidence
389.9546	2.85E-15	No evidence
64.43772	0.056651	No evidence
107.2189	0.000579	No evidence
15.56653	0.211906	No evidence
16.44821	0.171559	No evidence
356.1032	1.15E-11	No evidence
73.57141	0.057697	No evidence
366.3773	1E-12	No evidence
63.45733	0.066705	No evidence
66.42368	0.040176	No evidence
70.40251	0.272071	No evidence
273.3194	0.000151	No evidence
102.6713	0.093135	No evidence
60.19972	0.093608	No evidence
79.99613	0.072936	No evidence
9.891062	0.625517	No evidence

Table S7. The Clinical Cohort of cardiovascular disorders.

ID	Group	Age	Gender	BP	ALcohol	Smoke	CCT	RNFL	GCIPL
10001	HC	52	0	0	0	0	557	98	86
10002	HC	53	1	1	0	1	574	106	81
10003	HC	53	1	1	0	1	607	103	81
10004	HC	54	1	1	0	1	445	103	88
10005	HC	55	1	0	0	0	549	100	85
10006	HC	56	0	0	0	1	521	102	78
10007	HC	57	0	0	0	1	549	101	83
10008	HC	58	1	1	1	0	520	95	82
10009	HC	59	1	0	0	0	504	102	86
10010	HC	60	0	1	2	0	509	92	84
10011	HC	61	1	1	0	0	546	116	87
10012	HC	62	1	1	0	2	532	104	89
10013	HC	62	0	1	3	1	593	91	72
10014	HC	63	1	0	0	0	533	100	96
10015	HC	64	0	0	3	0	518	102	88
10016	HC	65	1	1	3	1	527	102	85
10017	HC	66	1	1	0	0	505	99	83
10018	HC	67	1	0	0	0	598	121	88
10019	HC	67	0	1	1	3	449	110	87
10020	HC	68	0	1	1	1	514	99	89
10021	HC	68	1	0	4	0	600	102	92
10022	HC	71	1	0	0	0	525	105	78
10023	HC	72	0	0	0	2	495	99	86
10024	HC	73	1	1	0	0	522	122	89
10025	HC	74	0	1	4	1	554	98	85
10026	HC	75	0	1	2	1	525	97	87
10027	HC	75	1	1	5	2	511	101	82
10028	HC	77	1	0	0	0	522	112	96
10029	HC	78	0	1	0	2	561	90	87
10030	HC	78	0	0	1	0	558	103	89
10031	HC	79	1	0	1	2	567	102	85
10032	HC	80	1	0	0	0	607	107	84
10033	HC	81	0	0	0	2	557	104	86
10034	HC	84	1	1	2	0	567	101	87
10035	HC	86	0	1	0	1	557	104	91
10036	CHD	47	0	0	2	0	519	95	86
10037	CHD	54	0	1	1	1	519	89	88
10038	CHD	55	1	1	1	2	598	95	87
10039	CHD	56	1	1	0	0	490	89	88
10040	CHD	56	0	1	0	2	510	96	92
10041	CHD	59	1	1	0	0	550	88	95
10042	CHD	59	1	1	0	0	498	88	85

10043	CHD	59	1	1	2	0	529	85	81
10044	CHD	61	1	1	0	1	578	97	86
10045	CHD	62	1	1	2	0	557	83	92
10046	CHD	63	0	1	1	0	557	97	85
10047	CHD	64	0	0	0	1	558	99	82
10048	CHD	66	1	0	0	1	558	98	83
10049	CHD	66	1	1	2	0	550	92	79
10050	CHD	67	1	1	0	3	598	96	80
10051	CHD	69	0	0	5	2	519	108	86
10052	CHD	70	1	0	0	0	511	82	88
10053	CHD	70	0	0	3	4	557	86	89
10054	CHD	71	0	1	3	0	592	69	94
10055	CHD	72	1	0	0	1	592	78	88
10056	CHD	73	1	1	1	1	598	69	82
10057	CHD	74	1	0	0	1	549	100	83
10058	CHD	74	1	0	1	0	490	101	75
10059	CHD	79	0	1	3	4	503	82	77
10060	CHD	84	0	1	1	2	483	97	82
10061	HF	60	1	1	0	0	552	98	78
10062	HF	64	1	1	2	0	537	90	73
10063	HF	64	0	1	3	3	552	98	83
10064	HF	65	1	1	0	1	540	92	80
10065	HF	66	1	1	2	0	516	101	79
10066	HF	69	1	0	0	0	540	124	99
10067	HF	73	1	1	0	0	577	98	87
10068	HF	75	0	0	0	4	562	102	97
10069	HF	75	1	1	1	2	549	100	86
10070	HF	81	0	1	1	2	516	101	81
10071	HF	82	0	0	4	1	577	103	93
10072	MI	48	0	1	0	1	517	58	80
10073	MI	52	1	0	0	2	542	91	89
10074	MI	55	1	1	0	0	521	95	78
10075	MI	56	0	1	5	3	511	85	82
10076	MI	58	1	1	0	0	503	77	81
10077	MI	58	1	0	0	3	575	73	93
10078	MI	60	1	1	0	2	588	79	88
10079	MI	61	0	1	0	0	517	117	82
10080	MI	61	0	0	3	0	537	89	92
10081	MI	62	1	1	3	0	559	102	78
10082	MI	64	0	1	0	3	537	88	83
10083	MI	65	1	1	2	0	588	95	82
10084	MI	67	1	1	0	1	477	106	85
10085	MI	68	1	1	0	0	477	71	84
10086	MI	68	0	1	5	4	516	96	86

10087	MI	73	1	1	1	0	538	87	87
10088	MI	74	0	0	1	4	516	91	92
10089	MI	76	0	1	1	3	537	87	83
10090	MI	80	1	0	2	0	467	91	87
10091	MI	81	1	1	0	1	466	97	85
10092	MI	82	0	0	4	0	521	85	89
10093	Stroke	51	0	0	5	0	573	79	92
10094	Stroke	54	1	0	0	0	573	100	90
10095	Stroke	56	0	1	3	1	573	89	82
10096	Stroke	58	0	1	1	3	491	88	84
10097	Stroke	63	1	0	0	0	476	86	86
10098	Stroke	68	1	0	0	4	533	88	87
10099	Stroke	75	0	1	0	0	543	107	82
10100	Stroke	77	1	1	0	0	476	89	84
10101	Stroke	77	1	1	1	2	533	86	85
10102	Stroke	85	1	1	1	0	543	85	81
10103	CA	53	1	1	2	0	558	96	82
10104	CA	57	1	1	0	2	568	95	89
10105	CA	62	1	1	0	0	529	100	79
10106	CA	62	0	1	3	0	529	97	92
10107	CA	65	1	1	0	0	551	102	77
10108	CA	65	1	1	0	0	539	105	86
10109	CA	65	0	1	0	3	550	99	82
10110	CA	65	1	1	1	0	550	94	81
10111	CA	65	1	0	1	3	549	107	90
10112	CA	74	0	1	1	5	543	88	82
10113	CA	81	0	0	2	0	593	99	86
10114	CA	83	0	0	1	0	593	100	88
10115	AF	54	1	1	0	1	534	95	82
10116	AF	58	0	1	5	3	540	82	81
10117	AF	59	1	1	0	1	519	104	80
10118	AF	60	1	0	0	0	478	131	91
10119	AF	61	1	1	0	0	575	108	83
10120	AF	61	0	1	2	0	575	90	85
10121	AF	68	1	1	0	2	480	91	86
10122	AF	68	1	1	1	0	546	100	87
10123	AF	70	0	0	1	4	547	98	96
10124	AF	71	0	1	0	0	584	102	80

INL	OPONL	PR-IS/OS	RPE-BM	Inner	Outer	WBC	RBC	Hb	PLT
40	88	65	23	224	176	7.23	4.23	163	315
42	92	67	21	229	180	6.84	4.65	144	246
43	101	68	20	227	189	9.9	5.5	163	229
46	82	63	26	237	171	5.94	4.24	133	201
52	79	60	28	237	167	6.32	3.98	131	350
36	100	66	26	216	192	5.58	4.29	137	161
39	92	72	23	223	187	7.82	4.79	142	218
41	78	64	22	218	164	5.37	4.07	125	266
40	87	64	20	228	171	5.77	4.59	145	178
40	89	65	26	216	180	8.02	5.32	156	264
38	84	63	22	241	169	5.18	4.85	140	152
36	93	62	27	229	182	8.56	3.75	113	316
50	96	64	23	213	183	8.09	4.99	157	158
42	95	62	24	238	181	6.22	4.34	130	248
39	87	66	21	229	174	8.72	4.6	139	262
44	89	65	22	231	176	7.67	3.76	174	230
45	94	67	24	227	185	7.85	4.19	152	317
42	101	69	22	251	192	6.95	3.6	113	217
40	98	61	21	237	180	8.12	4.82	136	206
35	96	62	24	223	182	5.48	4.94	151	273
42	89	67	26	236	182	4.77	3.68	112	291
36	85	65	24	219	174	9.6	4.81	142	225
40	93	68	23	225	184	6.46	5	156	235
38	88	63	27	249	178	4.65	4.65	111	257
41	80	65	18	224	163	6.55	4.3	158	232
37	90	68	22	221	180	6.88	4.46	131	265
41	92	63	23	224	178	8.13	5.54	161	198
40	86	66	24	248	176	8.83	3.8	122	252
38	79	69	22	215	170	5.54	4.39	137	289
37	94	64	21	229	179	5.35	4.24	134	198
41	88	68	26	228	182	6.08	4.28	140	117
37	79	68	23	228	170	5.05	4.52	135	314
39	89	64	22	229	175	7.85	4.13	147	234
41	93	64	23	229	180	7.62	4.6	112	242
37	95	65	24	232	184	5.63	4.71	151	135
41	90	68	22	222	180	5.29	4.94	148	124
45	93	72	21	222	186	4.67	4.53	129	203
38	79	65	25	220	169	4.69	4.01	148	220
42	89	66	26	219	181	7.87	4.5	126	225
37	95	62	17	225	174	7.38	4.03	139	176
47	95	67	19	230	181	6.04	4.69	130	322
51	102	71	22	224	195	6.48	3.77	121	322

42	79	68	26	208	173	6.03	3.66	139	213
41	84	69	26	224	179	8.65	4.92	113	190
40	88	70	25	215	183	6.58	4.3	146	131
36	94	64	22	218	180	4.21	4.51	139	211
38	93	71	21	219	185	6.07	4.59	132	268
42	78	68	20	223	166	8.8	4.47	133	202
40	88	66	25	211	179	7.1	4.13	144	204
41	84	68	24	217	176	8.8	4.74	167	218
38	89	71	17	232	177	6.51	5.19	143	208
46	95	72	23	216	190	9.72	4.9	133	263
42	94	74	17	217	185	10.24	4.63	148	321
36	90	68	22	199	180	8	4.84	124	196
38	86	69	24	204	179	6.18	4.39	146	527
34	96	69	24	185	189	4.55	4.81	163	213
43	91	73	22	226	186	11.83	5.39	144	156
39	90	75	23	215	188	6.3	4.59	135	215
42	89	73	21	201	183	10.8	4.21	142	260
40	86	69	20	219	175	9.18	4.82	116	180
33	95	65	24	209	184	5.4	3.48	163	356
47	92	62	21	210	175	7.5	5.2	87	242
36	88	66	22	217	176	5.93	2.78	137	294
38	95	69	25	210	189	8.79	4.65	160	259
35	83	70	23	215	176	9.12	5.96	138	226
42	78	63	24	265	165	5.31	4.58	150	145
38	98	62	26	223	186	7.66	5.11	120	266
48	97	61	19	247	177	4.46	4.1	114	175
42	92	65	20	228	177	4.81	4.12	138	205
38	87	62	22	220	171	5.75	4.72	143	278
44	86	63	19	240	168	7.49	4.59	159	341
46	89	68	25	184	182	9.55	5.27	142	171
44	96	69	21	224	186	6.86	4.36	145	231
45	101	68	23	218	192	5.86	4.52	134	238
40	98	72	17	207	187	6.62	4.63	155	177
38	76	75	21	196	172	7.99	4.67	138	180
55	79	68	22	221	169	5.31	4.24	149	286
42	89	62	21	209	172	7.54	5.03	135	182
40	96	65	26	239	187	6.42	4.4	127	265
43	88	69	17	224	174	10.42	4.24	161	375
47	85	72	19	227	176	8.25	5.5	137	239
44	87	74	22	215	183	7.82	4.21	131	228
48	95	68	20	225	183	6.01	4.47	141	144
49	79	67	19	240	165	4.42	4.75	138	318
39	100	73	22	194	195	6.5	4.41	127	227
41	87	74	15	223	176	6.23	3.92	138	293

52	89	69	21	226	179	6.08	4.53	126	319
50	88	74	20	233	182	9.07	3.72	122	325
37	96	64	18	207	178	7.63	4.3	114	137
52	92	73	22	230	187	4.24	3.18	140	157
41	90	72	21	223	183	4.79	4.76	117	146
42	92	75	19	216	186	4.42	4.42	157	214
41	90	63	20	212	173	10.42	5.41	143	214
48	86	59	26	238	171	9.31	3.36	156	160
42	79	68	21	213	168	5.8	4.9	135	310
38	89	69	19	210	177	5.26	4.29	116	157
34	96	68	27	206	191	6.92	3.69	122	367
51	95	69	19	226	183	7.6	4.31	144	201
34	103	72	21	223	196	5.86	4.03	150	157
41	90	62	21	214	173	5.49	4.82	133	162
43	88	64	17	214	169	7.29	4.35	133	254
39	91	62	21	205	174	4.82	4.57	127	267
40	88	63	23	218	174	7.18	4.1	126	269
42	84	62	25	226	171	8.12	4.31	140	275
36	96	67	25	215	188	5.03	4.26	157	191
43	97	73	21	232	191	6.45	4.44	138	257
49	87	68	27	228	182	8.23	4.68	158	218
42	76	63	26	233	165	6.09	5.02	140	121
34	96	63	21	215	180	6.41	3.82	137	212
42	95	62	20	217	177	8.58	4.19	139	211
38	89	63	18	235	170	5.88	4.08	153	292
42	86	65	19	212	170	7.65	4.81	123	221
41	92	62	25	226	179	5.3	3.98	139	262
38	92	65	26	226	183	5.4	4.58	130	416
50	84	64	25	227	173	8.71	4.17	158	167
38	84	68	17	201	169	4.77	5	119	285
37	95	69	24	221	188	8.5	3.93	131	342
41	87	62	28	263	177	7.16	4.58	120	306
43	93	73	20	234	186	5.37	4.06	150	472
40	88	74	24	215	186	7.89	4.67	120	188
39	89	64	21	216	174	6.83	3.82	131	236
42	99	66	22	229	187	8.3	4.21	108	95
36	78	68	18	230	164	3.17	3.42	157	141
41	89	70	26	223	185	7.44	5.29	158	379

ALT	AST	GGT	TBIL	ALB	TP	Crea	Urea	UA	TC
35	53	30	7.4	42.1	75.9	59.8	6.88	336	4.7
9	15	27	17.2	44.9	70.9	75.4	4.8	354	6.18
74	45	50	14.6	46.8	79.9	69	5.53	354	5.7
30	34	42	9.8	45.1	82.6	64.2	7.24	305	6.6
11	14	34	1.7	46	81	106.6	6.98	277	8.6
29	24	28	16.8	46.7	78	77.9	9.88	374	4.5
19	15	26	8.6	44.9	77.3	74.3	5.98	305	3.9
30	25	39	8.9	44.4	79.1	57.8	4.37	293	6.4
84	73	40	10.1	46.7	87	68.8	6.03	343	5.2
20	24	19	14.9	43.9	70.5	79.8	5.7	365	5.5
13	20	25	10.2	42.9	74.1	89.3	5.18	288	4
13	13	19	2	45.9	78.9	102.6	9.74	375	5.1
26	13	45	11	43.5	76.2	82.2	8.99	297	4.8
12	18	19	5.9	43	74.9	59	4.61	296	5.9
12	20	7	3.4	44.9	81.1	61	7.5	232	4.9
21	24	46	12.1	46.5	81.3	98.6	6.69	514	5
15	14	23	5.1	42.9	71.9	70.7	3.75	310	3.2
19	21	25	2.7	38.1	71.4	88.5	8.06	211	3.3
36	25	11	3.9	42.8	75.2	47.4	4.39	181	5.9
14	11	28	8.6	44.6	74.1	70.4	4.3	323	5.5
16	25	13	5	43.4	74.8	65	7.35	208	5.7
12	18	17	16.7	46.2	81.6	82.4	7.6	210	4
21	23	42	9.2	47.9	87.7	76	5.11	254	3.4
49	21	31	8.9	45.4	78.1	60.9	2.92	294	4.4
31	26	45	13.8	44.8	78.7	84.9	6.23	350	5.1
19	18	31	13.7	47.1	80	98	5.6	329	4.05
9	12	18	20	48.3	78	68.4	4	315	6.14
19	18	20	10.4	45.6	70.4	103.2	7.8	300	4.49
28	25	25	6.8	48.7	77.7	53.5	5.58	274	6.1
13	18	38	6.4	41.5	70.9	70.7	5.42	365	6.5
33	47	35	12.7	49.3	77.6	58.6	5.46	284	5.1
9	29	28	10.6	40.9	72.5	90.7	4.48	189	5
15	14	27	11	42.9	79.1	73.8	6.94	292	4.5
52	52	38	6.9	44.2	82.9	55.2	4.61	306	5.5
17	21	28	7	43.3	74.7	86.4	5	315	4.4
20	25	35	3.4	41.4	69.2	65.7	4.02	303	4
21	25	18	16.2	44.8	74.9	57.2	5.5	391	6.6
18	19	18	8.8	47.7	76.8	62.7	9.21	271	7.5
20	21	26	6.2	43.4	75.9	83.6	7.68	307	8.4
19	19	16	6.1	44.5	73.7	63.7	5.09	306	6.7
14	18	13	11.8	46.1	77.2	73.3	5.23	242	6.8
24	18	15	16.4	45.3	75.8	56.2	4.53	237	5.8

31	12	22	12.2	45.9	80.9	52.3	6.12	164	7.3
10	18	22	8.8	42.3	75.4	101.6	6.8	308	6.8
24	15	70	23.7	45.8	73.4	76.1	6.31	390	8.8
30	34	35	7.8	44.4	72	75.5	9.32	474	7.4
25	33	48	8.9	44.7	74.6	41.7	4.32	266	4.7
14	17	16	8.3	43.6	72.9	80	6.68	344	5.3
16	16	53	9.8	47.3	69.5	61	4.77	239	4.6
25	23	71	14.9	46.8	71.2	74.8	5.5	301	4.87
14	24	21	3.4	42.1	70.4	91.1	8.61	345	4.8
28	31	25	7.2	40.6	71.6	66.8	5.82	241	5.2
19	24	65	5.8	48.7	81.1	75.3	5.07	390	5.3
12	16	45	8.7	40.5	74.8	99	8.4	321	6.5
11	17	25	13.8	45	78.8	68.4	7.53	288	5.9
23	17	23	10.6	45.4	70.3	75.7	3.77	397	4.3
23	23	28	20.4	44.6	70.7	68.5	5.4	393	4.1
47	33	29	5.4	49.3	85	67.8	6.11	304	4.6
19	13	17	10.4	47.1	74.5	82.6	7.08	268	5.4
22	24	13	12	40.6	75.1	70.5	4.39	389	4
13	16	37	6.1	45.7	68.9	57.3	4.45	218	5.3
13	21	51	1.7	40	73	102.2	9.28	516	4.1
19	18	25	17.3	43.2	71.5	56.9	4.7	289	5.26
23	22	22	6.9	43.9	77.1	78.7	4.98	299	6.5
16	15	16	4.2	45.6	77.6	78	5.68	284	6.7
27	21	44	12.5	45.4	76.5	73.4	6.07	338	2.3
29	20	17	7.1	44.5	73.6	81.7	6.94	141	7.7
16	26	39	7.8	41.7	71.7	133.4	7.82	400	5.8
27	28	21	33.6	44.9	74.5	81.6	6.65	211	4
25	27	25	7.2	45.1	73.5	60.9	6	326	6.7
38	21	52	10	49.3	80.9	65.6	4.99	371	7
12	19	28	15.8	44.4	68.4	81.2	4.86	535	4.6
26	24	24	15	45	75.6	63	5.29	305	7.3
34	34	15	9.9	46.2	82.9	69.1	5.84	304	5.2
53	36	107	18	48.5	76.1	78.9	9.13	417	8.8
27	24	29	4.6	43.3	71.6	74.8	8.14	246	4.6
10	23	32	11.5	43.6	75.4	79.8	7.56	279	5.5
18	17	65	7.4	43.8	76.3	42.2	3.73	169	5.3
19	19	22	11.7	42.6	74.9	110.1	11.8	472	8.2
40	23	30	8.2	50.8	86.6	66.1	3.26	362	6.6
32	21	18	15.5	46.3	76.1	107.2	6.38	317	5
41	44	36	30.1	43.6	74.3	56	3.48	388	5.9
10	16	21	10.7	47.8	74.3	90.8	5.3	191	4.9
15	21	41	6.9	38.7	69.3	71.8	3.25	269	5.7
11	14	24	15.5	40.1	83	119.5	8.51	391	4.1
14	18	26	6.7	44.6	75.1	57.4	6.06	254	7.1

35	29	26	9.8	47.2	76.5	69.8	5.55	350	6.8
19	14	49	8.9	44	74.3	88.1	5.99	345	6.2
15	15	24	19.2	46.3	72	76.6	5.49	348	3.7
26	25	25	3.3	47.4	74.4	101.6	7.77	308	6.8
11	20	16	9.3	44.3	73.6	56.6	5.17	234	3.9
12	11	25	6.9	42.5	75.2	89.4	6.55	276	5.1
25	19	10	3	41.6	78.8	74.7	7.23	378	5.5
12	19	18	10.6	46.2	76.1	62	4.21	298	4.8
22	13	22	15.4	45.3	76.1	57.6	4.31	320	5.8
23	18	15	6.8	41.2	70.9	55.8	4.9	254	5.1
15	15	10	10.3	47.1	85.4	64.7	6.56	287	7.7
12	26	14	12.3	41.7	70.1	93.3	7.38	306	5.4
16	16	15	9.1	48.8	76.3	71.6	4.69	348	7.5
54	54	170	12.5	42.4	75.1	36.7	4.54	261	4.9
19	27	16	7.1	45.2	74.7	78.2	7.22	391	5.5
21	17	21	10.6	41.1	68.9	77.7	7.9	317	6.1
15	17	17	5.1	37.7	74.6	112.8	12.09	300	5.3
14	20	29	7.8	47.7	73	71.3	6.08	315	5.6
18	17	39	16.4	44	75.5	74.9	4.57	295	5.5
24	20	31	29.6	47.1	78.4	89.7	7.84	336	7.1
44	27	18	16.6	47.7	72.9	96.4	6.5	356	8.4
41	35	36	17.9	45.3	69.1	55.1	3.33	434	3.4
53	48	86	11.9	45.3	75.5	73.2	5.36	278	5.6
12	18	20	13.4	44.1	70.2	78.7	5.32	299	4.2
29	19	27	17.8	42.5	71.8	76.7	7.62	316	8.5
19	24	27	8	46.1	81.3	91.1	12.08	250	5.5
11	15	21	9	45.2	72.7	64.5	3.92	282	6.1
22	16	34	4.4	38.5	68.5	35.1	4.26	231	5.1
53	38	51	11.9	49.2	81.3	81.2	6.95	352	4.9
12	18	36	3.1	41.5	71.4	69.5	6.49	311	7.2
23	22	24	5	44.7	75	52.1	4.11	361	4.8
13	18	20	12.2	45.4	75	85.7	6.23	317	6.9
16	23	39	7.7	42.7	73.6	115.9	7.13	464	6.5
18	23	15	4.3	41.3	77.1	114.9	7.3	187	5.6
17	14	49	8.9	41.5	66	149.1	14.87	311	3.1
36	37	42	4.3	42.7	73.7	50.7	5.89	376	4.8
27	18	16	14.8	44.3	74.9	106.7	6.92	357	6
15	20	9	7	43	68.2	69.8	5.99	266	4.6

TG	HDL_C	LDL_C	LPA	apoA	apoB	Glu	ALP
1.67	1.3	1.02	88.3	1.05	0.62	6.6	88
2.1	1.36	0.92	282.1	1.17	1.07	6.4	118
2.08	1.72	0.83	51.9	1.76	0.8	5.4	109
2.8	1.61	1.91	43.2	0.72	0.83	8	80
1.56	1.06	1.74	234.3	0.96	1.22	5.3	78
1.48	1.42	2.67	457.7	1.41	0.81	6.6	60
1.25	1.29	1.02	90.3	1.32	0.82	8.3	95
1.13	1.32	1.73	111.4	1	0.46	4.5	130
1.09	0.87	1.97	63.9	0.83	0.81	3.9	52
0.62	0.9	0.49	48.2	0.75	0.62	5.4	67
1.53	1.54	2.83	54.3	1.2	0.76	5.9	81
1.43	0.61	2.56	29.5	1.45	0.74	6.6	69
1.2	0.82	1.07	131.8	0.78	0.59	5.3	60
1.16	1.2	2.31	189.8	1.02	0.63	5.5	63
2.15	1.06	0.98	793.5	1.29	0.85	5.6	84
1.05	0.86	1.45	37.9	1.23	0.31	4.8	58
1.04	0.81	2.39	767.1	0.86	0.7	4.5	94
3.03	1.42	2.39	113.8	1.3	0.67	5	59
4	1.12	2.05	25.5	0.94	0.78	5.8	63
1.99	0.9	2.11	58.4	1.06	0.71	5.6	82
0.64	1.79	2.47	100.1	1.4	1.27	5.1	84
1.28	1.3	3.69	54.3	1.15	0.99	4.4	57
0.87	0.9	2.4	61.9	0.98	0.72	5.1	62
1.47	1.01	1.14	50	0.98	0.92	3.3	84
0.56	1.72	2.8	26.8	0.81	0.81	6.3	76
0.68	0.82	3.36	111.7	0.86	0.96	5.5	116
1.6	0.75	2.47	147	1.23	0.69	4.9	83
1.58	0.94	2.74	194.9	1.12	0.77	5.6	112
0.43	1.15	0.61	85	1.23	0.66	6.8	73
0.74	1.45	2.02	130.5	1.19	0.58	5.7	100
1.32	2.14	1.73	47.8	1.63	0.8	6.4	72
1.3	0.72	2.97	23.5	0.74	0.85	5.3	104
0.84	1.71	2.49	51	0.8	0.41	4.4	53
0.93	1.49	0.83	425.9	1.52	1	5.7	105
1.23	1.02	2.56	73.3	1.06	0.48	5.7	53
1.18	1.18	3.07	739.9	1.04	1.3	3.6	105
2.15	1.31	2.31	255.3	1.16	1.57	5.4	78
0.12	1.1	2.98	23.5	1.02	0.89	5.1	77
1.11	1.42	3.45	42.5	1.19	0.69	5.8	63
3.1	0.9	2.39	804.3	0.88	0.72	6.4	64
2.15	0.77	2.39	33.1	1.11	0.9	5.5	69
2.53	1.35	4.05	131.6	1.04	1.05	4.8	55

4.3	0.67	4.11	70.4	1.12	0.83	4.1	61
1.05	1.11	3.1	787	1.32	0.79	5.3	99
2.03	0.61	2.61	383.5	1.01	0.66	6	72
1.03	1.08	3.28	78.7	0.82	0.68	5.3	84
1.54	0.44	3.43	38	0.84	1.02	4.9	55
0.99	1.13	4.46	131.8	1.12	1.43	5.6	92
1.52	0.35	3.14	79.2	0.58	0.93	4.6	93
1.96	1.12	3.97	649.1	0.46	0.8	5	108
0.72	1.14	3.07	41.4	1.07	1.83	5.8	76
1.94	1.58	2.14	78.3	1.02	0.53	4.6	73
3.32	0.93	2.67	52.1	0.46	0.83	5.6	117
0.89	1.04	3.27	72.4	1.72	0.66	6.4	65
0.62	0.32	2.39	78.5	0.8	0.59	4.5	90
0.92	1	3.18	243	0.69	0.92	4.7	84
1.87	1.08	3.78	46.4	0.78	0.82	4.4	71
0.87	1.02	3.69	59.3	0.9	0.5	5.7	84
0.83	0.87	2.39	57.6	0.86	0.59	7	71
1.85	1.03	1.73	49.5	1.4	0.62	5.1	50
2.45	1.32	2.97	36.4	1.1	1.09	5	86
1.94	1.42	2.49	235.7	1.09	0.76	5.2	82
1.62	1.08	2.83	59.6	0.93	0.66	5.2	72
1.46	2.02	2.56	630.5	1.22	0.97	5	87
1.44	0.9	2.07	88.1	0.91	0.71	5.3	98
1.38	1.17	2.31	735.6	1.03	1.55	6.6	60
1.21	1.53	2.98	710.2	1.26	0.98	5.3	102
1	0.9	1.45	441.8	0.65	0.97	4.6	91
0.97	1.39	2.39	30.5	1.12	0.61	5.3	83
1.98	0.98	2.39	250.7	0.88	0.62	5.3	60
0.91	1.25	3.05	175.3	1.11	0.75	5.6	82
1.9	1.15	2.11	80.9	1.47	1.16	5.9	77
1.46	1.02	1.98	39.2	1.3	0.6	6.5	110
0.68	0.98	2.19	253.7	0.94	0.67	5	72
2.14	1.43	2.48	285	1.17	0.73	4.2	81
2.03	1.44	3.93	118.9	1.14	1.12	6.2	64
3.88	1.13	2.78	377	1.08	0.78	5.2	182
1.43	1.08	1.52	49.3	1.37	0.42	7.3	110
1.68	1.14	2.26	232.9	0.52	0.61	7.4	52
1.49	0.7	1.9	69.8	0.75	0.52	5.4	82
1.37	1.67	3.63	289.4	1.12	0.99	5.1	59
1.17	1.08	1.73	312.7	0.54	1.28	5.2	116
1.06	0.8	2.97	209.5	0.94	0.65	5.6	53
1.04	0.81	2.49	176.6	1.2	0.77	4.6	80
0.99	0.92	2.83	222.9	0.86	1.27	5.8	45
1.59	1.08	2.56	392	0.84	1.34	5.1	91

1.74	1.02	2.07	35.6	1.42	1.11	4.5	70
1.83	1.05	2.31	67.2	1.04	1.16	5.8	62
2.89	0.86	2.98	161.3	0.94	1.1	4	66
0.94	0.98	1.45	179.9	0.84	0.89	5.4	72
2.86	1.02	2.39	58.4	1.01	1.09	5.3	78
1.66	1.02	2.39	150.4	0.94	0.73	7	74
0.64	1.4	3.05	353.8	1.29	1.36	6.6	52
2.1	0.51	2.11	78.5	0.58	1.38	4.7	65
1.78	0.67	2.96	58.7	0.69	0.75	5.7	90
1.73	0.98	2.18	502.9	0.94	1.28	6.7	80
1.68	1.41	2.53	136.7	0.86	0.74	7.5	96
1.93	1.34	3.4	123.5	0.86	1.14	3.9	81
0.83	1.08	3.26	104	1.36	0.92	5.5	72
1.46	0.84	2.76	158	0.41	1.15	4.2	127
1.44	1.13	2.24	129.7	0.78	0.92	6	58
1.38	0.95	2.96	127.3	0.81	0.87	6.4	83
1.81	1.32	2.56	373.5	0.89	0.74	4.2	79
1.52	1.14	1.67	298.3	1.07	0.9	3.9	60
1.96	1.58	1.85	25.9	1.28	0.93	9.2	84
1.31	0.93	2.61	92.2	1.53	0.95	4.2	75
1.91	1.53	3.4	32.9	1.43	0.93	5.9	60
1.38	0.67	2.52	60.8	0.79	0.76	5.7	70
1.62	1	2.19	724	0.86	0.65	10	85
1.68	1.1	2.84	670.8	1.56	0.79	5.3	84
2.14	1.02	2.54	65.9	0.82	0.87	3.5	88
2.03	0.87	2.02	88.3	1.05	0.62	4.4	88
1.46	1.03	1.73	282.1	1.17	1.07	8.5	106
1.54	1.04	2.97	51.9	0.76	0.8	9.9	61
1.58	1.13	2.49	243.2	0.79	0.83	5.1	72
1.16	0.89	2.83	234.3	0.96	1.22	6.5	79
1.62	2.05	2.56	457.7	1.41	0.81	4.8	59
2.78	0.98	2.07	90.3	1.12	0.82	5.5	88
0.88	1.17	2.31	111.4	1	0.46	6.4	73
2.79	0.53	2.98	63.9	0.86	0.81	4.1	40
1.9	0.98	1.45	248.2	0.79	0.62	4.9	137
1.63	1.39	2.39	54.3	1.2	0.76	7	82
0.78	0.98	2.39	29.5	0.71	0.74	8.9	91
3.14	1.25	3.05	131.8	0.79	0.59	4.8	74
